# Serotype Screening of *Salmonella enterica* Subspecies I by Intergenic Sequence Ribotyping (ISR): Critical Updates

**DOI:** 10.3390/microorganisms11010097

**Published:** 2022-12-30

**Authors:** Jean Guard, Deana R. Jones, Richard K. Gast, Javier S. Garcia, Michael J. Rothrock

**Affiliations:** U.S. Department of Agriculture, Agricultural Research Service, U.S. National Poultry Research Center, 950 College Station Road, Athens, GA 30605, USA

**Keywords:** *Salmonella enterica*, serotype, whole genome sequencing, homologous recombination, polymerase safety, genomics, salmonellosis, adenylate cyclase, lipopolysaccharide

## Abstract

(1) Background: Foodborne illness from *Salmonella enterica* subspecies I is most associated with approximately 32 out of 1600 serotypes. While whole genome sequencing and other nucleic acid-based methods are preferred for serotyping, they require expertise in bioinformatics and often submission to an external agency. Intergenic Sequence Ribotyping (ISR) assigns serotype to *Salmonella* in coordination with information freely available at the National Center for Biotechnology Information. ISR requires updating because it was developed from 26 genomes while there are now currently 1804 genomes and 1685 plasmids. (2) Methods: Serotypes available for sequencing were analyzed by ISR to confirm primer efficacy and to identify any issues in application. Differences between the 2012 and 2022 ISR database were tabulated, nomenclature edited, and instances of multiple serotypes aligning to a single ISR were examined. (3) Results: The 2022 ISR database has 268 sequences and 40 of these were assigned new NCBI accession numbers that were not previously available. Extending boundaries of sequences resolved *hdfR* cross-alignment and reduced multiplicity of alignment for 37 ISRs. Comparison of gene *cyaA* sequences and some cell surface epitopes provided evidence that homologous recombination was potentially impacting results for this subset. There were 99 sequences that still had no match with an NCBI submission. (4) The 2022 ISR database is available for use as a serotype screening method for *Salmonella enterica* subspecies I. Finding that 36.9% of the sequences in the ISR database still have no match within the NCBI *Salmonella enterica* database suggests that there is more genomic heterogeneity yet to characterize.

## 1. Introduction

Foodborne illness caused by *Salmonella enterica* (*S. enterica*) is a persistent threat to the health of people around the world, and outbreaks are closely monitored within the U.S. [1,2,3]. The genus *Salmonella* has two species, namely *S. bongori* and *S. enterica*. Foodborne pathogens are concentrated in one of the six subspecies (subsp.) namely *S. enterica* subsp. I, which has the synonym of *S. enterica* subsp. *enterica* [4]. There are some instances where other subspecies of *S. enterica* subsp. I cause illness, but overall, they are infrequently encountered as public health issues. The information provided here focuses on *S. enterica* subsp. I taxid:59201 and sometimes broaden searches to all of *Salmonella* in taxid:28901 (Search: *Salmonella enterica*–NLM (last accessed on 18 December 2022 (nih.gov)).

There are approximately 1600 serotypes within *S. enterica* subsp. I. Of these 1600 serotypes, 32 (2.0%) have genomes optimized for clonal expansion, virulence factors, environmental persistence, genetic adaptability, and the ability to be easily transferred between ecological niches associated with humans, animals, and the handling and processing of food [5]. Of the 32 serotypes, approximately twelve are of greater concern because they account for about 90% of foodborne outbreaks. The twelve serotypes, in approximate order of magnitude as evaluated from current information from the Centers for Disease Control (CDC), the Food and Drug Administration (FDA) and the USDA Food Safety and Inspection Service (FSIS) include the following serotypes: *S. enterica* subsp. I serotype Enteritidis (*S*. Enteritidis), *S.* Typhimurium, *S.* Newport, *S.* Javiana, *S.* Heidelberg, *S.* Hadar, *S.* Infantis, *S.* Montevideo, *S.* Muenchen, *S.* Braenderup, *S.* Saintpaul, and *S.* Senftenberg [6,7]. Additionally, included in the list of top twelve serotypes is *S.* 4,[5],12:i:-, which is a serotype that expresses O-antigen and H1 flagellar epitopes; however, it lacks flagellar H2 epitopes due to mutation [8]. There are annual variations in the relative incidence of frequently isolated salmonellae. Another serotype of note is *S.* Kentucky because it is frequently isolated from agricultural environments, but it does not often cause human disease; however, it does harbor antibiotic resistance that can impact in-hospital nosocomial disease [9].

Serotyping of *S. enterica* subsp. I was founded on nearly 70 years of information produced by using a complex panel of monospecific antisera to characterize epitopes on the outer membrane of the bacterial cell. The process is referred to as the Kauffman-White-LeMinor (KWL) scheme [10]. The targeted epitopes are associated with the complex carbohydrate O-antigen repeating unit of lipopolysaccharide (LPS) and two proteins expressed from genes *fliC* and *fljB*. Expressed proteins from these two genes, which undergo phase variation, comprise the major structural component of the flagellar organelle used for motility. These antigenic variants are called H1 and H2 in the KWL scheme. National responses and regulatory actions for the three serotypes are different, and the U.S. poultry industry has eradicated *S.* Gallinarum and *S.* Pullorum due to their threat to the entire poultry industry; in contrast, *S.* Enteritidis remains a constant threat to the safety of the food supply [11,12]. Detection of *S.* Gallinarum or *S.* Pullorum in U.S. poultry flocks necessitates stringent quarantine and eradication measures to protect the economic viability of the egg industry. Detection of *S.* Enteritidis falls under regulatory guidelines and might trigger a traceback investigation or other measures intended to reduce the risk of food contamination [13].

Most of the bioinformatics pipelines for receiving, processing, analyzing, and interpreting whole genome sequences (WGS) are associated with government agencies and both FDA and USDA-FSIS have regulatory responsibilities for the safety of food products in the United States [14]. Regulators use both MLST and WGS to conduct source attribution following outbreaks, and there is an association between MLST and WGS with serotype [15]. Source attribution requires resolving genome sequence to the single nucleotide polymorphism (SNP) and stringent bioinformatics [16,17]. This level of analysis is not needed by companies wanting to keep environments associated with producing food free of *Salmonella*. Instead, companies need streamlined information on the presence of *Salmonella*, on the presence of regulated serotypes such as *S.* Enteritidis, and if serotype populations fluctuate throughout the year. The ability of ISR to distinguish between the closely related serotypes *S.* Gallinarum and *S.* Pullorum provides an example of how ISR can be used in field studies for initial screening of larger sample numbers and then informing additional genomic analyses of selected strains [18].

The Centers for Disease Control developed SeqSero2 for epidemiological investigations of outbreaks in humans, and it associates serotype to WGS [19]. Agencies across the government collaborate with each other, confirm serotype designations with the National Veterinary Services Laboratory (NVSL), and consult with other researchers and public health departments, on issues involving *Salmonella* contamination of food sources (Participants|PulseNet USA|CDC). Large government supported databases are invaluable resources for epidemiological investigations, and they can also be used to evaluate world-wide trends by coordinating analyses with other international databases [20,21]. Companies producing food have expressed concerns about submitting samples to government-based pipelines beyond regulatory requirements because there is potential liability associated with the duty of responsibility to report and a loss of data ownership [22]. Thus, domestic and international agricultural companies are inhibited from using MLST or WGS in a manner that makes full use of their technological power.

For the reasons cited above, and especially to encourage routine screening for the presence *Salmonella enterica* within any operation producing food and food products, Intergenic Sequence Ribotyping (ISR) was developed. The initial development of ISR focused on distinguishing non-motile *S. enterica* subsp. I *S.* Gallinarum and *S.* Pullorum, which lack both H1 and H2 antigens, from rare variants of *S.* Enteritidis that did not express either variant [23]. Previous research found that the *dkgB*-linked ISR region was the most useful for investigating poultry-associated *Salmonella* [23]. It is a PCR-based method developed further for the purpose of screening for contamination and assigning *S. enterica* subsp. I serotype names that have potential for causing foodborne illness [24]. ISR is not designed to make a definitive identification of serotype but instead functions best as a quality control measure. Since cultures are the starting point for analysis, companies can make later submissions if *Salmonella* serotypes appear to be present that might be of concern from either a regulatory viewpoint or from a general concern that products are free of *Salmonella.*

Following is an abbreviated description of the major steps for performing ISR, and further details for processing samples are described in Materials and Methods:

(i) After purification of DNA from cultures suspected to be *Salmonella*, amplifying primers ISR F1 and ISR R1, described in detail in Section 2.4, are used to target sequence spanning part of the 23S ribosomal gene *rrlH* and part of the gene encoding 2,5-diketo-D-gluconate reductase B (*yafB* in *S.* Typhimurium reference strain NC_003197.2; *dkgB* in *S.* Enteritidis reference strain NC_011294.1). The amplicon product will include sequence from the end of the *rrlH* gene, sequence that includes all the 5S ribosomal gene *rrfH* and its 5′ and 3′ flanks, tRNA-*asp,* and part of the *dkgB* gene. Within the reference strain for the genus of *Salmonella enterica*, namely *S.* Typhimurium LT2 (NC_003197.2), the ISR amplicon region with primers is 1444 nt and is located between 294,123 and 295,567 bp [25].
(ii) Sequence is obtained from the amplicon product by using primers ISRs1_F8 and ISRs2_R42, which are located internal to the 5′ and 3′ ends of the amplicon, in separate PCR reactions. Forward and reverse orientations are advised for best resolution. Reactions are then submitted or processed in-house to obtain sequences. If submitted, the client receives the sequence by private link.
(iii) The client then uses commercially available bioinformatics packages to trim ambiguous nucleotides, and trimmed sequences are batch aligned to the ISR database; alternatively, text recognition software can be used if bioinformatics software is not available.
(iv) Trimmed sequence can also be compared by BLAST to available genomes at NCBI. Parameters for aligning sequence to the most likely *S. enterica* subsp. I named serotype are 100% query coverage and 100% identity with no ambiguities.

The ISR database was first developed when there were 26 completed chromosomal *S. enterica* genomes available at NCBI. The ISR database was expanded beyond that of NCBI by combining it with a sequencing project analyzing strains submitted from many sources and coordinating it with a DNA hybridization AOAC approved method for assigning serotyping used in the EU [26]. By the end of 2012, there were 220 ISR sequences available upon request, and another 24 were added between 2012 to 2020. The NCBI database has grown since 2012 to include a list of 1804 chromosomal and 1419 plasmid completed genomes for *S. enterica* subsp. I (taxid: 59201) (last date accessed 21 September 2022). Ten years later after its initial development, it is time to review the ISR database, expand it to include more serotypes accessioned at NCBI, identify issues with interpretation of data, and to identify any problems in application.

## 2. Materials and Methods

### 2.1. Determining the Size of the Ncbi Database in 2022

ISR accessioning of the NCBI database uses only completed genomes due to assembly issues involving redundancies within ribosomal gene sequences. At site Genome–NCBI–NLM (nih.gov), 1804 genomes are listed after filtering for completeness. The number of genomes published per year can also be estimated. When conducting microbe BLAST searches for all subsp. of *S. enterica* (taxid:28901), 3938 genomes are listed, which include 1685 plasmids (Nucleotide BLAST: Search nucleotide databases using a nucleotide query (nih.gov)). BLAST search for complete genomes of *S. enterica* subsp. I (taxid:59201) lists 3327 genomes including 1419 plasmids. Therefore, the range of completed chromosomal genomes at NCBI for *S. enterica* subsp. I is between 1804 to 1908.

### 2.2. Bioinformatics Software and Analytics

There are several sources of suitable software. For the analyses here, Geneious Prime^®^ 1 January 2022 Build 15 March 2022 11:43 was used throughout. NCBI also has applicable bioinformatics, annotations, search engines, BLAST analysis algorithms, and other bioanalytic tools (National Center for Biotechnology Information (nih.gov)). NCBI is the source for *S. enterica* complete genomes.

### 2.3. Culture and Initial DNA Extraction

To begin analysis, 200 isolates of *S. enterica* subsp. I were grown on Brilliant Green (BG) agar (Acumedia; Neogen Corporation, Lansing, MI, USA) from stock frozen in glycerol and maintained at −80 °C at the U.S. National Poultry Research Center (USNPRC) in Athens, GA, USA. All isolates had been stored for at least 2 years and were chosen to maximize serotype variability. However, some serotypes were duplicated to analyze variation in results. Cultures on BG plates were stored at 4 °C after culturing, and then shipped per regulations to RSI Poultry Veterinary Consulting (DeSoto, KS, USA). At RSI, *Salmonella* isolates were grown in Tryptic Soy Broth for 18–24 h at 37 °C. One (1) mL of broth culture was harvested and processed for DNA extraction. DNA extraction was performed using the PureLink™ Genomic DNA Mini kit (Invitrogen Cat#K1820-02). DNA was eluted in 260 μL of PCR water. DNA was then spotted onto Whatman™ FTA Cards (GE Healthcare Bio-Sciences Corp., Piscataway, NJ, USA) for storage [27]. A 15-day DNA quality control ISR PCR run was performed on 40 samples to check for the ability to repeat results.

### 2.4. Preparation of Primers

Handling of all primers and DNA was done within a Mystaire CleanPrep Station. PCR Primers were ordered from IDT (accessed 13 January 2021 (www.idtdna.com)), and parameters were 25nmole DNA oligos with standard desalting. Primers were diluted in PCR pure water to obtain a 100 pmol/μL concentrated solution. The concentrate was diluted to a working concentration of 10 pmol/μL in 9 μL of PCR water. The concentrate and working solutions were frozen at −20 °C until used. Amplifying primers ISR_F1 and ISR_R1 were used in the first phase to make sure DNA from the *dkgB* region was amplified. Product size could vary as much as 250 bp, and bands in gels are typically seen around 1400 bp markers. Sequencing primers, used separately to obtain forward and reverse sequence, were ISRs1_F8 and ISRs2_R42. Primer sequences were:
>ISR F1GCCAATGGCA CTGCCCGGTA(20 nt)>ISR R1TACCGTGCGC TTTCGCCCAG(20 nt)>ISRs1_F8AGGCCGGGTG TGTAAGCGCA(20 nt)>ISRs2_R42CGGAACGGAC GGGACTCGA(19 nt)

### 2.5. Pcr Amplification of DNA Samples

Master mix was DreamTaq Hot Start (ThermoFisher), and 23 μL were aliquoted into 0.2 mL PCR tubes (ThermoFisher, Suwanee, GA, USA). DNA samples were extracted from Whatman FTA card spots as previously described XX. Extracted DNA samples and negative sample controls, 2 μL, were added to the aliquots of master mix. Samples were processed in a Techne Prime Thermal Cycler (ThermoFisher) using the following parameters: (i) initial denaturation at 94 °C for 2 min; (ii) 35 cycles of 94 °C for 30 s, 64 °C for 30 s, and 72 °C for 2 min; (iii) final extension at 72 °C for 5 min; (iv) hold at 4 °C. Amplified samples, 15 μL, were purified using DNA Clean & Concentrator-5™ (Zymo Research, Irvine, CA, USA) according to the manufacturer’s directions.

### 2.6. Electrophoresis

Gel electrophoresis was performed in a 1/10 dilution of the PCR amplicon was as a quality control measure to assess successful amplification of a single band. Materials used for electrophoresis were Agarose I™ biotechnology grade (ThermoFisher), 0.5 mL GelRed^®^ Nucleic Acid Stain (ThermoFisher), 100 bp DNA Molecular Weight Marker (Invitrogen, Waltham, MA, USA), 4X Gel loading buffer (BPB) (ThermoFisher), and distilled water molecular grade. GelRed stain was added to 1% warm agarose prepared in TBE, and 25 mL of agarose was loaded into the gel tray chamber with 1X TBE buffer. Each amplified DNA sample, 2 μL, was mixed with 7 μL loading dye on parafilm and then loaded into gel wells. One well was loaded with 5 μL of DNA molecular weight markers. The run was conducted at a constant 100 V for at least 30 min, and then observed under UV light (254 nm) to visualize the expected band size of approximately 1400 bp.

### 2.7. Sample Preparation for Sequencing

Purified PCR product, 5 μL, was combined separately with 5 μL of 10 pmol sequencing primers ISRs1_F8 and ISRs2_R42 in either 0.2 mL tubes or in 96-well plates. Premixed samples were submitted to Eurofins for Sanger sequencing (accessed 27 June 2022 (https://eurofinsgenomics.com/en/products/dna-sequencing/all-sequencing-options/)). Samples were submitted at ambient temperature on the same day as PCR product purification. Use of a company’s services for sequencing does not constitute endorsement by the U.S. Department of Agriculture.

### 2.8. Analysis of Sequence

Two hundred (200) strains from a variety of serotypes were processed by ISR to try to (i) encounter any problem that a user might experience, (ii) address those issues, and (iii) offer solutions and advice. File management is an issue, and users should consider how they will accession both strains and files associated with strains. Accessioning by processing date with the format YYMMDD works well. The user needs to determine what metadata are important to their operation. Finally, the information should be stored in a secure location, initial data should be saved as a master file that undergoes no further processing, and personnel with access to the data should understand the parameters of interpretation. Raw sequence files could come in an a *.abi format, which can be directly imported by bioinformatics software. Alternatively, some sequencing companies might supply plain text files for importation.

After importing and securing raw data files, the next step in analysis is to copy raw data files to a separate file for progressive trimming of both 5′ and 3′ ends. Using Geneious software (version 2022.1.1), 35 nucleotides (nt) were trimmed off both ends and the option of removing all ambiguities was selected. Sequences that were shorter than 500 nt after trimming were individually evaluated, and most were discarded for poor quality as defined by internal ambiguous results depicted in sequence as “N”. After confirming the quality of trimmed sequences, all sequences were aligned with ISR database sequences to observe if both forward (F) and reverse (R) reactions had substantial overlap. The best quality result is when both F and R sequences span the entire ISR post-trimming. If sequence in only one direction aligns with an ISR, then it is appropriate to accept results if the length of sequence covers the entire ISR. If F and R sequences align to different ISRs, then that sample will require more scrutiny, as will be discussed under troubleshooting results. Users are encouraged to submit novel sequences not included in the updated ISR to the communicating author for further analysis and possible linkage to a known serotype.

## 3. Results

### 3.1. Characteristics of the 2022 ISR Database as Compared to 2012

The previous ISR database last made available upon request had 242 individual sequences, and the current one has 268 sequences; thus, there are 26 new entries. The 2022 database was blasted against the NCBI *Salmonella enterica* subsp. I (taxid:59201) as a FASTA formatted file for immediate application by users (last date accessed 21 September 2022) (Supplement File S1). Within the 2022 update, the average length for ISR-C sequences was 438.2 nt, standard deviation was 79.25 nt, and the range was 257 to 556 nt. As will be discussed, the average length for extended ISR sequences (ISR-X) was 1302.9 nt, standard deviation was 158.59 nt, and the range was 898 to 1499 nt. Table 1 includes results from the first database aligned with current information at NCBI. Parameters for defining a conventional ISR (ISR-C) alignment were 100% Query Coverage (QC) and 100% identity with no ambiguities. In the updated ISR list, phenotypic designations such as “monoflagellated” or “possibly Java” were removed because ISRs are DNA based. However, O-antigen Group B immunogroups (IG), such as IG 1,4,[5],12:i:-, were retained as they have become distinguishable from related serotypes at the genomic level.

### 3.2. Repeatibility of 2012 and 2022 Database Sequence Alignments

Of the 242 sequences in the 2012 database, 169 had no change (69.8%). There were 40 previously reported sequences that aligned with new NCBI accession numbers in the 2022 database. Thus, 209 sequences of the 242 in the 2012 database (86.4%) repeated had no substantial change in 2022. This percentage compares favorably to MLST analysis of serotype [16]. Finding that ISR sequences within the 2012 database were eventually matched to a NCBI accession possibly reflects that the USDA laboratory received samples from more varied environments and sources as compared to submissions sent to NCBI. Overall, 100% of the 2012 database is contained within the 2022 database, and differences between the two are catalogued in Table 1.

### 3.3. Assessing Confidence in Assigning Serotype to an ISR Sequence

Table 1 includes an assessment of the confidence with which serotype is associated with an ISR. The confidence groups are as follows:

(A) Three or more strains align with a single serotype,

Ab) Greater than ten strains align with a predominant serotype with completed genomes, and alignment with additional serotypes accounts for no more than 20% of the total. For all genomes at any stage of completion, *S.* Enteritidis and *S*. Typhimurium together include 60,478 genomes (32.7%) of the 184,731 *Salmonella enterica* subsp. I genomes (synonymous with *Salmonella enterica* serovar *enterica*) at NCBI, whereas another 96 serotypes comprise the remainder of the dataset and range from 1 to 12,076 submitted genomes (Salmonella enterica subsp. enterica–NCBI–NLM (nih.gov): last date accessed 6 December 2022). Thus, the “Ab” confidence rating accounts for database size of complete genomes available for searching.

(B) Fewer than 3 strains align with a single serotype,
(C) Two serotypes, distinguishable by a simplified KWL scheme, align with the same ISR,
(D) Three or more serotypes align with a single ISR, requiring additional analysis by KWL, MLST, or WGS.

Previous determinations of serotype by alternative methods, such as DNA hybridization or submissions with completed KWL immunotyping, were kept within the 2022 database. Strains not available for further analysis are indicated in Table 1 by “not available (na)”, and entries with this designation may have unique sequences without a NCBI accession or any other knowledge of serotype.

Table 1 shows data for 268 ISR results classified by specificity of alignment and an assessment of confidence. An example of how to read results is ISR 147. It aligned with 4 *S. enterica* serotypes, namely *S.* Typhimurium, *S.* Enteritidis, *S.* Hissar, and *S.* Albert. Using the first 2 letters to indicate the respective serotype, column H reports 127Ty:1En:1Hi:1Al. The formula is interpreted as ISR-X 147 aligned perfectly to 127 strains of Typhimurium and to 1 strain each of the other 3 serotypes. Representative NCBI accession numbers respective to the listing of strains aligned is provided in an adjacent column. The confidence assessment for this ISR is thus assigned as Ab, because a typical strain of Typhimurium is about 40 times more likely to be encountered than one of the rarer serotypes. It is important for users to recognize that the NCBI database is skewed in numerical representation to those serotypes of most concern to public health. Increasing submissions of a serotype to NCBI often correlates with its relative importance for impacting human health.

### 3.4. Evaluating Multiplicity of Serotype Alignment to a Single ISR

Multiplicity of serotype alignment to a single ISR is important because one such instance, specifically ISR 37, is a known example of homologous recombination impacting serotype variability [28]. To see if there was further evidence of homologous recombination impacting ISR results, all results that had alignments to multiple serotypes with NCBI accessions are shown in Table 2. The adenylate cyclase (*cyaA*) sequence for each serotype was downloaded, aligned, and examined for having 100% query coverage (QC) and 100% alignment identity (ID) with no ambiguities. Adenylate cyclase was chosen as a secondary site in the genome to evaluate because it is a large housekeeping gene that infrequently generates single nucleotide polymorphisms (SNPs), and it is required for full metabolic potential and virulence of *S. enterica* subsp. I. Additionally, listed, respective to the order within the multiple alignment, is the KWL O-antigen grouping for each serotype. Results are that *cyaA* sequence and O-antigen grouping can differ; thus, chances are that homologous recombination events are impacting serotype variability and resulting in unlikely pairings. These results also suggest that submission errors to NCBI due to mixtures of serotypes in the same sample are not substantially impacting results because processing of data would be likely showing an unacceptable degree of nucleotide ambiguity.

The 2012 database used strict parameters to define an ISR sequence. The convention was to use the first nucleotide after the end of the *rrlH* 23S ribosomal gene to the nucleotide that preceded tRNA-*asp* as the ISR sequence for BLAST searches. [24]. However, additional sequence is generated during the same sequencing reactions at no extra cost. Extending the boundaries of the conventional ISR to include unambiguous sequence that was previously trimmed increased specificity for assigning serotype in some cases by decreasing multiple alignments. Extended sequences are labeled with an “X” after the ISR number in the 2022 database, whereas the conventional length sequences are labeled “C” (Supplement File S1). For the 37 instances of multiple alignments shown in Table 2 (13.8% of the 2022 database), 6 were resolved to one serotype and 21 were resolved to 2 serotypes using ISR-X. Ten (10) ISRs were not improved by using ISR-X and had 3 or more serotype alignments. Of the 21 that were resolved to 2 serotypes, one O-antigen antisera from the KWL scheme would differentiate 12 of them, whereas 9 shared the same O-antigen epitopes [10]. These results indicate that 93% of ISRs could be assigned a serotype name using a single O-antigen to differentiate some double alignments. As with MLST and WGS, there are always outliers that require further analysis or multiple approaches to best assign a strain to a serotype or closest evolutionary group [16,17]. The recent description of a *S.* Lubbock-*S.* Mbandaka hybrid identified by WGS was also identified by ISR as being unusual; in addition, some *S.* Typhimurium strains appeared to align with this hybrid, which is “(37)X” in Supplement File S1 (Table 2).

Thirty-one (31) serotypes (11.6% of the 2022 database) in Table 2 could not be resolved to a single serotype by using an extended ISR region, and thus they aligned with two or more serotypes. The most extreme example is *S.* Typhimurium, which appears 9 times in combinations with other serotypes (Table 2). In contrast, *S.* Enteritidis appears twice in Table 2, and both times together with *S.* Typhimurium. This result supports the theory that *S.* Typhimurium retains its position as one of the top 3 persistent cause of foodborne salmonellosis because it has an exceptional ability to undergo homologous recombination [29]. In contrast, *S.* Enteritidis is especially evolved to colonize and persist in modern food commodities and might not accept donor DNA efficiently because its genome is at a peak of optimization [30]. The next most frequently occurring serotype within Table 2 is *S.* Newport, which appears 6 times. If frequency of ISR appearance in multiple serotypes is an indication of donor capability, then *S.* Newport also appears to be a serotype that is highly competent at undergoing homologous recombination.

There are serotype pairings that appear multiple times in Table 2. Examples are *S.* Newport and *S.* Bardo (ISRs 87 and 136), *S.* Saintpaul and *S.* Stanleyville (ISRs 92 and 141), a different pairing of *S.* Saintpaul and *S.* Stanleyville (ISRs 141 and 184), and *S.* Newport and *S.* Abaetetuba (199 and 222). These pairings resulted from ISRs that differed either by SNPs or by length of ISR-X. It is possible there is some preference for homologous recombination to occur for some pairs. Of note is that *S.* Newport and *S.* Bardo are primarily differentiated by bacteriophage content and that serotypes *S.* Senftenberg and *S.* Dessau are not differentiated by *cyaA* sequence or the KWL typing scheme (10). Thus, it is possible that some pairings indicate variants within a single serotype, and thus they should not be given individual names. The Pasteur KWL reference on *S. enterica* serotypes provides many examples of adjustments to interpretation of serotype [10]. In addition, MLST may be preferred as an alternative to the KWL scheme for grouping Salmonella for commonly encountered groups [20].

### 3.5. Plasmid Association of Isr Sequence

One of the more perplexing results that differentiated the 2012 and 2022 databases was finding 5 ISR sequences (1.9% of the 2022 database) that cross-aligned with a ribosomal gene region other than *dkgB*, namely *hdfR*. We took the 258 nt ISR-C 210 sequence and blasted it separately against all of *Salmonella enterica* (taxid:28901) and associated plasmids. The sequence was highly conserved within *S. enterica* subsp. I genomes, and 901 genomes had identities > 98% with query coverage of 100%. Five (5) plasmid alignments were reported and were from *S. enterica* subsp. I. One unnamed plasmid from *S.* Typhimurium strain SJTUF10484 (CP047533.1) had a striking alignment to ISR 210 with a query coverage of 100%, identity of 98.6%, a maximum score of 449, and an Evalue of 1 × 10^−125^. This large plasmid (96,002 bp) had several core genes, such as *cyaA*, *hemD*, and several LPS genes. Another plasmid from *S.* Senftenberg (LN86894.1) had a query coverage of 100%, percent identity of 98.04%, a maximum score of 355, and an Evalue of 3 × 10^−97^; however, it had only 200/204 identities as compared to 253/258 for the *S.* Typhimurium plasmid. There were 3 other alignments of much poorer similarity, 2 more from *S.* Senftenberg and 1 from *S.* Infantis. Thus, the plasmid with the ribosomal region associated with *hdfR* appears to be a rare find, as it was the only one with such a high-quality alignment.

To see if there were other Enterobacteriaceae with plasmids that were similar to the 96,002 bp plasmid CP047533, its genome was blasted against all other plasmids at NCBI, except that *S. enterica* was excluded. Out of 475 alignments, 3 plasmid genomes were between 90 to 100 kb and had scores suggesting shared similarity. These three plasmids were as follows:

(1) *Citrobacter* sp. TSA-1 plasmid unnamed 2/CP053575.1 (QC 87%/identity 89.20%/max score 16502).
(2) *Enterobacter ludwigii* pEN-119/CP017280.1 (QC 71%/identity 85.58%/max score 9483).
(3) *Kosakonia cowanii* p888-76-2/CP01944 (QC 68%/identity 84.35%/max score 9326).

This result suggests that DNA associated with the sequence of ISR 210 has potential to cross genus and species boundaries by homologous recombination, and that some unusual plasmids might be involved [31]. Finding an extrachromosomal element with potential for transmitting an ISR related sequence within the Enterobacteriaceae and that also has an association with serotype of *S. enterica* subsp. I is an intriguing but poorly understood result due to the rarity of the find. It perhaps bolsters the concept that *S.* Typhimurium is especially proficient at donating DNA amongst the Enterobacteriaceae.

## 4. Conclusions

The NCBI database of completed *Salmonella enterica* subsp. I genomes is exponentially larger today than it was in 2012, and it now includes approximately 3327 completed genomes including 1419 plasmids accessed for these analyses. Overall, the 2012 ISR database transitioned to the larger NCBI database of accessioned *Salmonella enterica* serotypes intact. The most challenging aspect encountered was the alignment of multiple serotypes with a single ISR sequence for 31 of 268 total sequences (11.9%). While extending the strict boundaries of the conventional ISR sequence reduced multiplicity of alignment, the phenomenon might provide insight into the evolution of *S. enterica* subsp. I that poses persistent challenges for protecting the safety of the food supply and the health of people and animals.

We suggest, based on evidence, that ISR sequencing is detecting instances of homologous recombination. Alternative explanations about the alignment of multiple serotypes to a single sequence are refutable. For example, a mixture of DNA from different strains is detectable during sequencing and should show conflicting associations between serotype and expected submission because of sequence differences in housekeeping genes located around the genome. Another alternative explanation to homologous recombination would be that the ISR database is more limited than expected for screening serotype. We have shown here that, of the 242 sequences available in 2012, 209 (86.3%) were not impacted by anything more than having NCBI release an associated accession number. Of the 268 sequences in the 2022 database, 237 out of 268 (88.4%) could be resolved to aligning with a single serotype if the ISR was extended, and 231 (86.2%) were resolved using the conventional ISR boundaries of 2012.

The 2012 database had 40 ISR sequences that were not matched with NCBI accessions until ten year later, which supports that ISR was identifying circulating serotypes faster than submissions of whole genomes were completed within the national database. The 2022 ISR database still contains 23 unique sequences that have not been matched to any database. These results support that another use of ISR is to select new strains for analysis by whole genome sequencing, which would help to avoid redundancy and provide a larger picture of evolution occurring in an important foodborne pathogen. It is important to note that ISR is a democratized assay, requiring no reporting of results or reference to its use [32]. Small in-house laboratories capable of conducting PCR and approved to culture BS level 2 pathogens such as *Salmonella* can use ISR for serotyping of *Salmonella enterica* subsp. I. Democratization of a simpler typing method that has been evaluated in reference to well-curated and publicly accessible information such as that at NCBI can help to put the ability to follow evolutionary trends of *Salmonella enterica* subsp. I occurring in association with food products in more hands.

## Figures and Tables

**Table 1:** Changes to the Salmonella enterica subspecies I Intergenic sequence ribotyping (ISR) database for screening serotype: 2012 versus 2022.

ISR	2022 ISR Serotype Alignments	2022 Change	Alignment Results	Representative NCBI Accession Numbers	Confidence Rank
1	Schwarzengrund	NCBI match	1of1	CP074340	B
2	Thompson	no change	Strain not available	NoNCBImatch	na
3	Cerro	no change	30f3	CP012833	A
4	Senftenberg	NCBI match	13of13	CP047424	A
5	Oranienburg Typhimurium	no change	4Or:1Ty	CP033344_CP029029	C
6	Typhimurium-IG	NCBI match	7 IG-1,4,[5],12:i:- & 4 IG-4,[5],12:i:-	NC_021820.1	A
7	Infantis	no change	1 of1	CP052777	B
8	Muenchen	NCBI match	30f3	CP077691	A
9	Enteritidis	no change	Strain not available	NoNCBImatch	na
10	Emek	no change	Strain not available	NoNCBImatch	na
11	Panama	NCBI match	2of3	CP012346	B
12	Oranienburg	no change	Strain not available	NoNCBImatch	na
13	Schwarzengrund_Bredeney_Give	no change	11Sc:5Br:1Gi:1other	CP085812_CP082691_CP019174	D
14	Bovismorbificans	NCBI match	30f3	CP073715	A
15	Gallinarum-pullorum	NCBI match	1of1	CP074215	B
16	Choleraesuis	NCBI match	1of1	CP074231	B
17	Choleraesuis	no change	Strain not available	NoNCBImatch	na
18	Paratyphi-B	NCBI match	2of2	CP074611	B
19	Dublin	no change	Strain not available	NoNCBImatch	na
20	Paratyphi-B	NCBI match	1of1	CP074222	B
21	Typhimurium	no change	2of2	CP040458	B
22	Paratyphi-B	NCBI match	1of1	CP074225	B
23	Yovokome	no change	1of1	CP019418	B
24	Newport	no change	2of2	CP025232	B
25	Tennessee_Montevideo	requires ISR-X	8Te:1Mo:1Ty	CP007505_CP030029	C
26	Fresno	no change	Strain not available	NoNCBImatch	na
27	Worthington	no change	5of5	CP039509	A
28	Anatum	no change	Strain not available	NoNCBImatch	na
29	Derby	no change	1of1	CP026609	B
30	Infantis	no change	Strain not available	NoNCBImatch	na
31	Paratyphi-B	NCBI match	1of1	CP074221	B
32	Derby	no change	3of3	CP022494	A
33	Derby_California	NCBI match	5De:1Ca	CP082627_CP028900	C
34	Newport	no change	1of1	CP015924	B
35	Kentucky	no change	28of28	CP026327	A
36	Typhimurium	no change	94Ty	LT795114	A
37	Mbandaka_Lubbock_Typhimurium	NCBI match	6Mb:2Lu:1Ty	CP033343_CP032814_CP011365	D
38	Montevideo	no change	5of5	CP017978	A
39	Montevideo	no change	10of10	CP020912	A
40	Montevideo	no change	28of28	CP032816	A
41	Newport	no change	4of4	CP015923	A
42	Abony	no change	1of1	CP007534	B
43	Corvallis	requires ISR-X	1 of1	CP051307	B
44	Senftenberg	no change	8of8	CP016837	A
45	Isangi	no change	Strain not available	NoNCBImatch	na
46	Ohio	no change	3of3	CP030024	A
47	Infantis	no change	Strain not available	NoNCBImatch	na
48	Cerro	no change	Strain not available	NoNCBImatch	na
49	Blockley	NCBI match	3of3	CP043662	A
50	Johannesburg	NCBI match	1of1	CP074325	B
51	Genovar_12350n	no change	Strain not available	NoNCBImatch	na
52	Miami	no change	Strain not available	NoNCBImatch	na
53	Saintpaul	no change	Strain not available	NoNCBImatch	na
54	Albany	no change	13of13	CP036165	A
55	Braenderup	no change	5of5	CP022490	A
56	Rissen	no change	Strain not available	NoNCBImatch	na
57	Kedougou	no change	Strain not available	NoNCBImatch	na
58	Enteritidis	no change	3of3	CP007598	A
59	Paratyphi-B	no change	1of1	CP020492	B
60	Muenster_Typhimurium	NCBI match	8M:1T	CP019198_CP074302	C
61	Muenchen	NCBI match	3Mu	CP022658	A
62	Enteritidis	NCBI match	1of1	CP045956	B
63	Alachua	no change	Strain not available	NoNCBImatch	na
64	Litchfield	NCBI match	1of1	CP082600	B
65	Kiambu	no change	Strain not available	NoNCBImatch	na
66	Anatum_Hayindogo	NCBI match	29An:1Ko:1Ha:1Ma:1Be	CP007584_CP017719	Ab
67	Meleagridis	no change	Strain not available	NoNCBImatch	na
68	Molade	no change	Strain not available	NoNCBImatch	na
69	Bareilly	no change	Strain not available	NoNCBImatch	na
70	Newport	NCBI match	1 of1	CP075033	B
71	Soerenga	NCBI match	1of1	CP074317	B
72	Norwich	no change	Strain not available	NoNCBImatch	na
73	Gera_Genovar_3109	no change	Strain not available	NoNCBImatch	na
74	Hartford	NCBI match	2of2	CP074274	B
75	Cerro	no change	Strain not available	NoNCBImatch	na
76	Cerro	no change	Strain not available	NoNCBImatch	na
77	Amsterdam	no change	Strain not available	NoNCBImatch	na
78	Taksony	no change	1of1	LR134146	B
79	Oranienburg	no change	Strain not available	NoNCBImatch	na
80	IG_6,8_Genovar_1678	no change	Strain not available	NoNCBImatch	na
81	Uganda	NCBI match	5of5	CP051398	A
82	Mississippi	no change	Strain not available	NoNCBImatch	na
83	Litchfield	no change	Strain not available	NoNCBImatch	na
84	Paratyphi-B	no change	1 of1	LR134233	B
85	Newport	no change	Strain not available	NoNCBImatch	na
86	Hadar	NCBI match	1of1	CP082396	B
87	Newport_Bardo	no change	2Ne:1Ba	CP016010_CP019404	C
88	Rissen	NCBI match	1of3	CP043509	B
89	Livingstone	no change	Strain not available	NoNCBImatch	na
90	IG_rough_Genovar_9261	no change	Strain not available	NoNCBImatch	na
91	Bareilly	no change	Strain not available	NoNCBImatch	na
92	Saintpaul_Stanleyville	NCBI match	1Sa:1St	CP017727_CP034716	C
93	Babelberg	no change	Strain not available	NoNCBImatch	na
94	Montevideo	NCBI match	1of1	CP074322	B
95	Oranienburg	no change	Strain not available	NoNCBImatch	na
96	Idikan	no change	Strain not available	NoNCBImatch	na
97	Manchester	NCBI match	1 of1	CP019414	B
98	Give	no change	2of2	LS483463	B
99	Brandenburg_Eastbourne_Reading_SanDiego	NCBI match	6Br:2Ea:1Re:1Sa:1other	CP030002_CP075115_CP093134_CP075039	D
100	Cubana	no change	Strain not available	NoNCBImatch	na
101	Pomona	no change	1of1	CP019186	B
102	Orion	no change	Strain not available	NoNCBImatch	na
103	Adelaide	no change	Strain not available	NoNCBImatch	na
104	Liverpool	no change	Strain not available	NoNCBImatch	na
105	Ouakam	no change	1of1	CP022116	B
106	Gallinarum	NCBI match	1of1	CP088142	B
107	Muenchen	no change	Strain not available	NoNCBImatch	na
108	Lindenburg	no change	Strain not available	NoNCBImatch	na
109	Typhimurium	no change	Strain not available	NoNCBImatch	na
110	Alabama	no change	Strain not available	NoNCBImatch	na
111	Rubislaw	NCBI match	1of1	CP074294	B
112	Kiambu	NCBI match	1of1	CP082587	B
113	Agbeni	no change	Strain not available	NoNCBImatch	na
114	Typhimurium	no change	Strain not available	NoNCBImatch	na
115	Derby	no change	Strain not available	NoNCBImatch	na
116	Nima	no change	Strain not available	NoNCBImatch	na
117	Baranquilla	no change	Strain not available	NoNCBImatch	na
118	Javiana	NCBI match	1of1	CP074206	B
119	Agona_Borreze_Muenchen	NCBI match	32Ag:1Bo:1Mu	NC_011149_CP019407_CP082684	Ab
120	Havana	NCBI match	1of1	CP074203	B
121	Anatum	no change	1of1	CP007211	B
122	Bareilly_Virchow_Saintpaul_Typhimurium	NCBI match	30Ba:2Vi:1Sa:1Ty:2other	CP045757_CP045945_CP023166_CP020565	Ab
123	Berta	no change	3of3	CP030005	A
124	Choleraesuis_Gallinarum	NCBI match	5Ch:1Ga	NC_006905_CP088134	C
125	Cubana	no change	2of2	NC_021818	B
126	Dublin	no change	5of5	NC_011205	A
127	Enteritidis_Newlands_Javiana_Typhimurium	NCBI match	125En:1Ne:1Ty:1Ja	NC_011294_CP082916_CP074314_CP019383	Ab
128	Gallinarum-gallinarum	no change	3of3	NC_011274	A
129	Gallinarum-pullorum	no change	6of6	NC_022221	A
130	Hadar_Anatum_Nchanga	NCBI match	11Ha:1An:1Nc	CP038595_CP074323_CP082370	D
131	Heidelberg_Crossness_IG-4,[5],12:i:-	NCBI match	36H:2 IG_4:5:12:i:-: 1Cr	NC_021812_CP019408_CP082588	Ab
132	Infantis	no change	54of54	CP019202	A
133	Javiana	NCBI match	4of4	NC_020307	A
134	Johannesburg	NCBI match	4of4	CP019411	A
135	Kentucky	no change	15of15	CP022500	A
136	Newport_Derby	NCBI match	37Ne:1De	NC_021902_CP075036	Ab
137	Newport	no change	Strain not available	NoNCBImatch	na
138	Paratyphi-A	no change	5of5	NC_006511	A
139	Javiana	NCBI match	1of2	CP085052	B
140	Paratyphi-C	no change	1of1	NC_021125	B
141	Saintpaul _Stanleyville	no change	10f1	CP045954_CP017723	B
142	Schwarzengrund	no change	1of1	NC_011094	B
143	Stanley	no change	1of1	LS483434	B
144	Thompson	no change	11Th	NC_022525	A
145	Typhi_Typhimurium	NCBI match	137:2other:1Ty	NC_003198_CP085809	Ab
146	Typhimurium_DT2	no change	3of3	NC_022544	A
147	Typhimurium_Enteritidis_Hissar_Albert	NCBI match	127Ty:1Al:1En:1Hi	NC_003197_CP044188_CP018657_CP088138	Ab
148	Unique	no change	Strain not available	NoNCBImatch	na
149	Unique	no change	Strain not available	NoNCBImatch	na
150	Unique	no change	Strain not available	NoNCBImatch	na
151	Brandenburg	no change	Strain not available	NoNCBImatch	na
152	Unique	no change	Strain not available	NoNCBImatch	na
153	Unique	no change	Strain not available	NoNCBImatch	na
154	Unique	no change	Strain not available	NoNCBImatch	na
155	Unique	no change	Strain not available	NoNCBImatch	na
156	Unique	no change	Strain not available	NoNCBImatch	na
157	Unique	no change	Strain not available	NoNCBImatch	na
158	Enteritidis	NCBI match	1of1	CP075019	B
159	Unique	no change	Strain not available	NoNCBImatch	na
160	Unique	no change	Strain not available	NoNCBImatch	na
161	Enteritidis	no change	2of2	CP009091	B
162	Infantis	no change	Strain not available	NoNCBImatch	na
163	Stanley	no change	1of1	CP036167	B
164	Give	no change	Strain not available	NoNCBImatch	na
165	Hartford	NCBI match	1of1	CP074660	B
166	Ouakam	no change	Strain not available	NoNCBImatch	na
167	Inverness	no change	2of2	CP019181	B
168	Reading	NCBI match	8of8	CP093132	A
169	Gaminara	NCBI match	1of1	CP030288	B
170	Meleagridis	no change	Strain not available	NoNCBImatch	na
171	Unique	no change	Strain not available	NoNCBImatch	na
172	Unique	no change	Strain not available	NoNCBImatch	na
173	Dublin	NCBI match	12of12	CP032449	A
174	Muenchen_Newport	NCBI match	4Mu:1Ne	CP051389_CP016014	C
175	Enteritidis	no change	1of1	CP018633	B
176	Indiana	no change	26In	CP022450	A
177	Derby	no change	Strain not available	NoNCBImatch	na
178	Sendai_Saintpaul	no change	Strain not available	NoNCBImatch	na
179	Rubislaw	no change	3of3	CP019192	A
180	Reading	no change	Strain not available	NoNCBImatch	na
181	Saintpaul	NCBI match	1of4	CP053055	B
182	Unique	no change	Strain not available	NoNCBImatch	na
183	Miami	no change	Strain not available	NoNCBImatch	na
184	Saintpaul _Stanleyville	NCBI match	1Sa:1St	CP017727_CP034716	C
185	Paratyphi_A	no change	1of1	CP009559	B
186	Nottingham	no change	Strain not available	NoNCBImatch	na
187	Typhimurium	no change	1of1	NC_021814	B
188	Typhimurium	no change	4of4	NC_016860	A
189	Typhimurium	no change	Strain not available	NoNCBImatch	na
190	Gaminara	no change	Strain not available	NoNCBImatch	na
191	Schwarzengrund	no change	Strain not available	NoNCBImatch	na
192	Enteritidis	NCBI match	1En	CP009083	B
193	Kentucky	NCBI match	1of1	CP082602	B
194	Mbandaka	no change	Strain not available	NoNCBImatch	na
195	Falkensee	no change	Strain not available	NoNCBImatch	na
196	Typhimurium	NCBI match	7of7	CP082526	A
197	Heidelberg	no change	10of10	CP012921	A
198	Infantis	no change	1of1	LS483479	B
199	Newport_Abaetetuba	NCBI match	1Ne:1Ab	CP016357_CP074211	C
200	Moscow	no change	1of1	CP019415	B
201	Typhimurium	no change	5of5	CP011428	A
202	Blegdam	NCBI match	1of1	CP019406	B
203	Wandsworth	no change	1of1	CP019417	B
204	Hillingdon	no change	1of1	CP019410	B
205	Newport_Derby	NCBI match	38Ne:1De	NC_021902_CP075036	Ab
206	Krefeld	no change	1of1	CP019413	B
207	Macclesfield	no change	1of1	CP022117	B
208	Meleagridis	NCBI match	1 of1	CP018642	B
209	Miami	no change	Strain not available	NoNCBImatch	na
210	Fresno_Javiana	requires ISR-X	1Fr:1Ja	CP032444_CP074283	C
211	Heidelberg	no change	1of1	LS483494	B
212	Kentucky	NCBI match	1of1	CP082582	B
213	Tennessee_Montevideo _Typhimurium	requires ISR-X	7Tn:1Ty:1Mu	CP007505_CP030029_CP034232	D
214	Kentucky	no change	Strain not available	NoNCBImatch	na
215	Orion	no change	Strain not available	NoNCBImatch	na
216	Unique	no change	Strain not available	NoNCBImatch	na
217	Reading	no change	Strain not available	NoNCBImatch	na
218	Unique	no change	Strain not available	NoNCBImatch	na
219	Unique	no change	Strain not available	NoNCBImatch	na
220	Newport	NCBI match	2of2	CP025232	B
221	Onderstepoort	no change	1of1	CP022034	B
222	Abaetetuba_Newport	NCBI match	1Ab:1Ne	CP007532_CP074207	C
223	Antsalova	no change	1of1	CP019116	B
224	Apapa	no change	1of1	CP019403	B
225	Djakarta	no change	1of1	CP019409	B
226	Hvittingfoss	no change	1of1	CP022503	B
227	Quebec	no change	1of1	CP022019	B
228	Sloterdijk	no change	1of1	CP012349	B
229	Waycross	no change	1of1	CP022138	B
230	India	no change	1of1	CP022015	B
231	Alachua	no change	Strain not available	NoNCBImatch	na
232	Kentucky	no change	Strain not available	NoNCBImatch	na
233	Haifa	no change	Strain not available	NoNCBImatch	na
234	London_Concord	NCBI match	7Lo:2Co	CP060132_CP028196	C
235	Unique	no change	Strain not available	NoNCBIMatch	na
236	Paratyphi-B	NCBI match	1of2	CP074668	B
237	Saintpaul	no change	2of2	CP023512	B
238	Duisburg	no change	Strain not available	NoNCBImatch	na
239	Pomono	no change	Strain not available	NoNCBImatch	na
240	Unique	no change	Strain not available	NoNCBImatch	na
241	Goldcoast	no change	1of1	LR134158	B
242	Corvallis	requires ISR-X	2of2	CP027677	B
243	Unique	new entry	Strain not available	NoNCBImatch	na
244	Unique	new entry	Strain not available	NoNCBImatch	na
245	Unique	new entry	Strain not available	NoNCBImatch	na
246	Unique	new entry	Strain not available	NoNCBImatch	na
247	Senftenberg_Dessau	new entry	2Se:1De	CP047424_CP038593	C
248	Sundsvall	new entry	1of1	LS483457	B
249	Bredeney_Give	new entry	2Br:1Gi	CP007533_CP019174	C
250	Chester	new entry	1of1	CP019178	B
251	Poona	new entry	2of2	CP019189	B
252	Heidelberg	new entry	1of1	CP051358	B
253	Bergen	new entry	1of1	CP019405	B
254	Manhattan	new entry	1of1	CP022497	B
255	Inverness	new entry	1of1	CP075132	B
256	Othmarschen	new entry	1of1	CP066260	B
257	Choleraesuis-var-Kunzendorf	new entry	3Ch	CP075031	B
258	Nitra	new entry	1of1	CP019416	B
259	Mikawasima	new entry	1of1	CP034713	B
260	Sandiego	new entry	1of1	CP075040	B
261	Mbandaka	new entry	1of1	CP019183	B
262	Reading	new entry	2of2	CP051307	B
263	Tennessee_Gaminara	new entry	1Ga:1Tn	CP075010_CP024165	C
264	Milwaukee	new entry	1of1	CP030175	B
265	Paratyphi-B_Typhimurium	new entry	2Ty:1Pa	CP024619_NC_010102	C
266	Java	new entry	1of1	LT571437	B
267	Koessen	new entry	1of1	CP019412	B
268	Meleagridis	new entry	1 of1	CP074321	B

**Table 2:** Association between the ISR, adenylate cyclase (cyaA) sequences, and O-antigen classification for serotypes of *Salmonella enterica* subspecies I that had multiple alignments.

ISR Number	ISR Sequences with Multiple Alignments and Results from Extending the ISR Region (ISR-X)		O-Antigen Classification Respective to Order in Column B (Shading Indicates Identical *cyaA* Genes)			
			O-Group 1	O-Group 2	O-Group 3	O-Group 4
Impact of ISR-X Sequences for Reducing the Number of Multiple Alignments to a Single Serotype						
4	Senftenberg_Dessau	resolved by ISR-X: Senftenberg	1,3,19 (E4)	1,3,19 (E4)	na	na
5	Oranienburg_Typhimurium	CP033344_CP029029	7 (C1)	4 (B)	na	na
11	Panama_Koessen	resolved by ISR-X: Panama	9 (D1)	2 (A)	na	na
13	Schwarzengrund_Bredeney_Give	CP085812_CP082691_CP019174	4 (B)	4 (B)	3,10 (E1)	na
14	Bovismorbificans_Chester	resolved by ISR-X: Bovismorbificans	8 (C2-C3)	1,3,19 (E4)	na	na
25	Tennessee_Montevideo	CP007505_CP034232	7 (C1)	7 (C1)	na	na
33	Derby_California	CP082627_CP028900	4 (B)	4 (B)	na	na
37	Mbandaka_Lubbock_Typhimurium	CP033343_CP032814_CP0113365	7 (C1)	7 (C1)	4 (B)	na
No 50	Poona_Johannesburg	resolved by ISR-X: Johannesburg	13 (G)	40 (R)	na	na
60	Muenster_Typhimurium	CP019198_CP074302	3,10 (E1)	4 (B)	na	na
61	Muenchen_Heidelberg	resolved by ISR-X: Muenchen	8 (C2-C3)	4 (B)	na	na
66	Anatum_Hayindogo	CP007584_CP017719	3,10 (E1)	1,3,19 (E4)	na	na
87	Newport_Bardo^2^	CP016010_CP019404	8 (C2-C3)	8 (C2-C3)	na	na
92	Saintpaul_Stanleyville	CP017727_CP034716	4 (B)	4 (B)	na	na
97	Manchester_Othmarschen	resolved by ISR-X: Manchester	8 (C2-C3)	7 (C1)	na	na
99	Brandenburg_Eastbourne_Reading_SanDiego	CP030002_CP075115_CP093134_CP075039	4 (B)	9 (D1)	4 (B)	4 (B)
119	Agona_Borreze_Muenchen	NC_011149_CP019407_CP082684	5 (B)	54	8 (C2-C3)	na
122	Bareilly_Typhimurium_Saintpaul_ Virchow	CP045757_CP036168_CP023166_CP045945	7 (C1)	4 (B)	4 (B)	7 (C1)
124	Choleraesuis_Gallinarum	NC_006905_CP088134	7 (C1)	9 (D1)	na	na
127	Enteritidis_Newlands_Javiana_Typhimurium	NC_011294_CP082916_CP074314_CP019383	9 (D1)	9 (D1)	4 (B)	4 (B)
130	Hadar_Anatum_Nchanga	CP038595_CP082370_CP074323	8 (C2-C3)	3,10 (E1)	3,10 (E1)	na
131	Heidelberg_Crossness_IG-4,[5],12:I:-	NC_021812_CP019408_CP082588	4 (B)	67	4 (B)	na
141	Saintpaul_Stanleyville	CP045954_CP017723	4 (B)	4 (B)	na	na
145	Typhi_Typhimurium	NC_003198_CP085809	9 (D1)	4 (B)	na	na
147	Typhimurium_Enteritidis_Hissar_Albert	NC_003197_CP044188_CP018657_CP088138	4 (B)	9 (D1)	7 (C1)	4 (B)
174	Muenchen_Newport	CP051389_CP016014	8 (C2-C3)	8 (C2-C3)	na	na
184	Saintpaul_Stanleyville	CP017727_CP034716	4 (B)	4 (B)	na	na
199	Newport_Abaetetuba	CP016357_CP074211	8 (C2-C3)	11 (F)	na	na
205	Newport_Derby	NC_021902_CP075036	8 (C2-C3)	4 (B)	na	na
210	Fresno_Javiana	CP032444_CP074283	9,46 (D2)	9 (D1)	na	na
213	Tennessee_Montevideo_Typhimurium	CP007505_CP034232_CP030029	7 (C1)	7 (C1)	4 (B)	na
222	Abaetetuba_Newport	CP007532_CP074207	11 (F)	8 (C2-C3)	na	na
234	London_Concord	CP060132_CP028196	3,10 (E1)	7 (C1)	na	na
247	Dessau_Seftenberg	CP047424_CP038593	1,3,19 (E4)	1,3,19 (E4)	na	na
249	Bredeney_Give	CP007533_CP019174	4 (B)	3,10 (E1)	na	na
263	Tennessee_Gaminara	CP075010_CP024165	7 (C1)	16 (I)	na	na
265	Paratyphi-B_Typhimurium	NC_010102_CP024619	4 (B)	4 (B)	na	na

## Data Availability

Contact M.R. (Michael.rothrock@usda.gov) for requesting the ISR database at the U.S. National Poultry Research Center following the retirement of J.G.

## Supplementary Materials

The following supporting information can be downloaded at: https://www.mdpi.com/article/10.3390/microorganisms11010097/s1, Supplement File S1 is the 2022 ISR database with 268 sequences. To convert the PDF to a plain text FASTA formatted file, copy and paste the entire file to Word using the text only option. Then, save the Word file as plain text (Notepad). This file can be uploaded directly to the NCBI microbe BLAST site available at: Nucleotide BLAST: Search nucleotide databases using a nucleotide query (nih.gov) accessed 19 December 2022).
