# Supplementary material for: Serotype Screening of Salmonella enterica Subspecies I by Intergenic Sequence Ribotyping (ISR): Critical Updates
Source: Microorganisms. 2022 Dec 30;11(1):97. doi: 10.3390/microorganisms11010097 (PMC9863722; doi:10.3390/microorganisms11010097)
Supplement: Supplementary file 1 [file microorganisms-11-00097-s001.zip › microorganisms-1965006-file S1.pdf]

>(1)C Schwarzengrund_CP074340

ATGTTTTGGC GGTTGAGAGA TGATTTTCAG CACTGATTCA GATTACATCA GAACGTAAAA 60

ACGGTCTGAT AACAGAATTT GCCTGGCGGC ACTAGCGCGG TGGTCCCACC TGACCCCATG 120

CCGAACTCAG AAGTGAAACG CCGTAGCGCC GATGGTAGTG TGGGGTCTCC CCATGCGAGA 180

GTAGGGAACT GCCAGGCATC AAATTAAGCA GTAAGCCGGT CATAAACCGG TGGTTGTAAA 240

AGAATCCGGT GGAGCGG 257

>(2)C Thompson_NoNCBImatch

ATGTTTTGGC GATTGAGAAG ACGATTTTCA GCACTGATTC AGATTACATC AGAACGTAAA 60

AACGGTCTGA TAACAGAATT TGCCTGGCGG CCGTAGCGCG GTGGTCCCAC CTGACCCCAT 120

GCCGAACTCA GAAGTGAAAC GCCGTAGCGC CGATGGTAGT GTGGGGTCTC CCCATGCGAG 180

AGTAGGGAAC TGCCAGGCAT CAATTTAAGC AGTAAACTGA TGTAAAAATC AGTGATTGTA 240

AAAGAATTCG GTGGAGCGG 259

>(3)C Cerro_CP012833

ATGTTTTGGC GGATGAGAGA AGATTTTCAG CACTGATTCA GAGTTGAGTA CGCAATAATT 60

TGCGCAGCAG CAAGGCGGCA AGCGAAGGAA AGGAAGGAGC ATACAGAAGT ATGTGACTGA 120

CTTTACGAGC GCAGGCAACG CCGCTGATGC GATAAAGAAT TGCGTACTGA GCACAAAAGA 180

ATTTGCCTGG CGGCACTAGC GCGGTGGTCC CACCTGACCC CATGCCGAAC TCAGAAGTGA 240

AACGCCGTAG CGCCGATGGT AGTGTGGGGT CTCCCCATGC GAGAGTAGGG AACTGCCAGG 300

CATCAAATTA AGCAGTAAGC CGGTCATAGA CTGGTGGTTG TAAAAGAATT CGGTGGAGCG 360

G 361

>(4)X Senftenberg_CP032194

CTTGCCCCGA GATGAGTTCT CCCTGAGACT TTAGGTCTCC TGAAGGAACG TTGAAGACGA 60

CGACGTTGAT AGGCCGGGTG TGTAAGCGCA GCGATGCGTT GAGCTAACCG GTACTAATGA 120

ACCGTGAGGC TTAACCTTAC AACGCCGAAG ATGTTTTGGC GGATGAGAGA TGATTTTCAG 180

CACTGATTCA GAGTTGAGTA CGCAATAATT TGCGCAGCAG CAAGGCGGCA AGCGAAGGAA 240

AGGAAGGAGC ATACAGAAGT ATGTGACTGA GTTTACGAGC GCAGGCAACG CCGCTGATGC 300

GATAAAGAAT TGCGTACAGA GCATAAAAGA ATTTGCCTGG CGGCCGTAGC GCGGTGGTCC 360

CACCTGACCC CATGCCGAAC TCAGAAGTGA AACGCCGTAG CGCCGATGGT AGTGTGGGGT 420

CTCCCCATGC GAGAGTAGGG AACTGCCAGG CATCAAATTA AGCAGTAAAC TGATGTAAAA 480

ATCAGTGGTT GTAAAAGAAT TCGGTGGAGC GGTAGTTCAG TTGGTTAGAA TACCTGCCTG 540

TCACGCAGGG GGTCGCGGGT TCGAGTCCCG TCCGTTCCGC CACTTATTAA GAGCCCTGAG 600

CATTAGCTCA GGGCTTTTTC TTTGCCTGTC ATTTAATTAT TGCTCAATTC GCAAAAATCC 660

TCTGCATTTC TCGCTCTTTT TCTGCAAAGC GCTGAAGGTC ATAATCCCCT CAGTTAACGA 720

ACATAGTGAT TAAAAAGAGG AATTCTATGA CTATCCCTGC ATTCGGTTTA GGTACTTTTC 780

GCCTGAAAGA CGACGTGGTT ATCGCTTCTG TGAAAACGGC GCTTGAACTG GGCTACCGTG 840

CTGTCGACAC TGCGCAAATC TATGATAATG AAGCGGCTGT TGGACAGGCG ATTGCTGAGA 900

GCGGCGTACC GCGTAATGAG CTGTACATCA CCACCAAGAT CTGGATTGAA AACCTTAGCA 960

AAGACAAGCT AATCCCAAGC CTGAAAGAGA GCCTGAAAAA ACTGCGTACC GATTATGTCG 1020

ATCTGACGCT GATCCACTGG CCATCTCCGG GTGATGCTGT GTCTGTCGAA GAATTTATGC 1080

AGGCATTGCT GGAAGCTAAA AAACAAGGGC TGACTCGCGA AATTGGCATT TCAAACTTCA 1140

CATTCCGTTG ATGAA 1155

>(5)X Oranienburg_Typhimurium_CP03344_CP029029

GGAAGAGATA AGTGCTGAAA GCATCTAAGC ACGAAACTTG CCCCGAGATG AGTTCTCCCT 60

GAGACTTTAG GTCTCCTGAA GGAACGTTGA AGACGACGAC GTTGATAGGC CGGGTGTGTA 120

AGCGCAGCGA TGCGTTGAGC TAACCGGTAC TAATGAACCG TGAGGCTTAA CCTTACAACG 180

CCGAAGATGT TTTGGCGGTT GAGAAGACAA CAATTTTCAG CACTGATTCA GAGTTGAGTA 240

CGCAATAATT TGCGCAGCAG CAAGGCGGCA AGCGAAGGAA AGGAAGGAGC ATACAGAAGT 300

ATGTGACTGA CTTTACGAGC GCAGGCAACG CCGCTGATGC GATAAAGAAT TGCGTACAGA 360

GCACAAAAGA ATTTGCCTGG CGGCAGTAGC GCGGTGGTCC CACCTGACCC CATGCCGAAC 420

TCAGAAGTGA AACGCCGTAG CGCCGATGGT AGTGTGGGGT CTCCCCATGC GAGAGTAGGG 480

AACTGCCAGG CATCAAATTA AGCAGTAAGC CGGTCATAAA CCGGTGGTTG TAAAAGAATT 540

CGGTGGAGCG GTAGTTCAGT TGGTTAGAAT ACCTGCCTGT CACGCAGGGG GTCGCGGGTT 600

CGAGTCCCGT CCGTTCCGCC ACTTATTAAG AGCCCTGAGC ATTAGCTCAG GGCTTTTTGT 660

CTATGGAAAA CCCCCAGCTA GGCTGGGGGT TCCGGAAAGC TTTCAGCTTT AAGCCAGTTA 720

TTAAAACCCC TTTTGATTTG TTAAAACATC TTGCGGTCTG GCAACTGCAA AAGTTCAACA 780

AGAAATCAAA AGGGGGTCCC AATGGGGGAC GAAAAGAGCT TAGCGCACAC CCGATGGAAC 840

TGCAAATATC ACATAGTTTT CGCGCCCAAA TACCGAAGAC AAGCGTTCTA TGGAGAGAAG 900

CGTAGGGCAG TAGGCAGCAT ATTAAGAAAA TTGTGTGAAT GGAAAAACGT ACGAATTCTG 960

GAAGCGGAAT GTTGTGCAGA TCATATTCAC ATGCTTCTGG AGATCCCGCC GAAGATGAGT 1020

GTGTCGAGCT TCATGGGATA TCTGAAGGGT AAAAGTAGTC TGATGCTTTA CGAGCAGTTT 1080

GGGGATCTAA AATTCAAATA CAGGAACAGG GAGTTCTGGT GCAGAGG 1127

>(6)C Typhimurium-IG-NC_021820

ATGTTTTGGC GGATTGAGAA GACAACAATT TTCAGCTCAG ATTCAGAGTC CGAAGGATTT 60

TACGCTGAGA CAAGGCGGCA AACGCAGCGA CGGAAGGAGC ATACAGAAGT ATGTGACTGA 120

CGCTCGCAAG AGCAGCCAAC GCCGTATCAG TGTAAAAGAC ACAGAACGGA GCACAAAGAA 180

TTTGCCTGGC GGCACTAGCG CGGTGGTCCC ACCTGACCCC ATGCCGAACT CAGAAGTGAA 240

ACGCCGTAGC GCCGATGGTA GTGTGGGGTC TCCCCATGCG AGAGTAGGGA ACTGCCAGGC 300

ATCAAATTAG AAGTATGGCA TTAACTTTCG GGGCAGTTCA CTACTCGACA GAGAGTAGGA 360

CAAAAGCGAA AGCTTTTGAA CGTTGCGAAG CAACGGCCCG AAGGGTGAAT CACGAAGTGA 420

TTCATAAACT GCCAGGCATC AAATTAAGCA GTAAACTGAT GTAAAAATCA GTGGTTGTAA 480

AAGAATTCGG TGGAGCGG 498

>(7)C Infantis_NoNCBImatch

ATGTTTTGGC GGTTGAGAAG ACGATTTTCA GCACTGATTC AGAGTTGAGT ACGCAATAAT 60

TTGCGCAGCA GCAAGGCGGC AAGCGAAGGA AAGGAAGGAG CATACAGAAG TATGTGACTG 120

AGTTTACGAG CGCAGGCAAC GCCGCTGATG CGATAAAGAA TTGCGTACAG AGCATAAAAG 180

AATTTGCCTG GCGGCAGTAG CGCGGTGGTC CCACCTGACC CCATGCCGAA CTCAGAAGTG 240

AAACGCCGTA GCGCCGATGG TAGTGTGGGG TCTCCCCATG CGAGAGTAGG GAACTGCCAG 300

GCATCAAATT AGAAGTATGG CATTAACTTT CGGGGCAGTT CACTACTCGA CAGAGAGTAG 360

GACAAAAGCG AAAGCTTTTG AACGTTGCGA AGCAACGGCC CGAAGGGGGA ATCACGAAGT 420

GATTCATAAG CTGCCAGGCA TCAAATTGCA GTAAACCGGG ATGAAAATCC GGATTGTGAC 480

AAAGAAATTC GGTGGAGCGG 500

>(8)C Muenchen_CP077691

ATGTTTTGGC GGTTGAGAGA CGATTTTCAG CACTGATTCC GGATTTTCGG GAACGAAAGA 60

TAAAAGATTC GTTTCCGGCA ACAGAATTTG CCTGGCGGCA CTAGCGCGGT GGTCCCACCT 120

GACCCCATGC CGAACTCAGA AGTGAAACGC CGTAGCGCCG ATGGTAGTGT GGGGTCTCCC 180

CATGCGAGAG TAGGGAACTG CCAGGCATCA AATTAGAAGT ATGGCATTAA CTTTCGGGGC 240

AGTTCACTAC TCGACAGAGA GTAGGACAAA AGCGAAAGCT TTTGAACGTT GCGAAGCAAC 300

GGCCCGAAGG GTGAATCACG AAGTGATTCA TAAACTGCCA GGCATCAAAT TGCAGTAAAC 360

CGGGATGAAA ATCCGGGTTG TGACAAAGAA ATTCGGTGGA GCGG 404

>(9)C Enteritidis_NoNCBImatch

ATGTTTTGGC GGATGAGAGA CGATTTTCAG CAGTGATTCA GAGTTGAGTA CGCAATAATT 60

TGCGCAGCAG CAAGGCGGCA AGCGAAGGAA AGGAAGGAGC ATACAGAAGT ATGTGACTGA 120

GTTTACGAGC GCAGGCAACG CCGCTGATGC GATAAAGAAT TGCGTACAGA GCACAAAGAA 180

TTTGCCTGGC GGCAGTAGCG CGGTGGTCCC ACCTGACCCC ATGCCGAACT CAGAAGTGAA 240

ACGCCGTAGC GCCGATGGTA GTGTGGGGTC TCCCCATGCG AGAGTAGGGA ACTGCCAGGC 300

ATCAAATTAA GCAGTAAGCC GGTCATAGAC TGGTGGTTGT AAAAAAATTC GGTGGAGCGG 360

>(10)C Emek_NoNCBImatch

ATGTTTTGGC GGATTGAGAA GACAACAATT TTCAGCTCAG ATTCAGAGTC CGAAGGATTT 60

TATGCTGAGA CAAGGCGGCA AACGCAGCGA CGGAAGGAGC ATACAGAAGT ATGTGACTGA 120

CGCTCGCAAG AGCAGCCAAC GCCGTATCAG TGTAAAAGAC ACAGGACAGA GCACAAAGAA 180

TTTGCCTGGC GGCACTAGCG CGGTGGTCCC ACCTGACCCC ATGCCGAACT CAGAAGTGAA 240

ACGCCGTAGC GCCGATGGTA GTGTGGGGTC TCCCCATGCG AGAGTAGGGA ACTGCCAGGC 300

ATCAAATTAG AAGTATGGCA TTAACTTTCG GGGCAGTTCA CTACTCGACA GAGAGTAGGA 360

CAAAAGCGAA AGCTTTTGAA CGTTGCGAAG CAACGGCCCG AAGGGTGAAT CACGAAGTGA 420

TTCATAAACT GCCAGGCATC AAATTGCAGT AAACCGGGAT GAAAATCCGG GTTGTGACAA 480

AGAAATTCGG TGGAGCGG 498

>(11)X Panama_CP012346

AACTTGCCCC GAGATGAGTT CTCCCTGAGA CTTTAGGTCT CCTGAAGGAA CGTTGAAGAC 60

GACAACGTTG ATAGGCCGGG TGTGTAAGCG CAGCGATGCG TTGAGCTAAC CGGTACTAAT 120

GAACCGTGAG GCTTAACCTT ACAACGCCGA AGATGTTTTG GCGGATGAGA GACGATTTTC 180

AGCACTGATT CAGAGTTGAG TACGCAATAA TTTGCGCAGC AGCAAGGCGG CAAGCGAAGG 240

AAAGGAAGGA GCATACAGAA GTATGTGACT GAGTTTACGA GCGCAGGCAA CGCCGCTGAT 300

GCGATAAAGA ATTGCGTACT GAGCATAAAA GAATTTGCCT GGCGGCACTA GCGCGGTGGT 360

CCCACCTGAC CCCATGCCGA ACTCAGAAGT GAAACGCCGT AGCGCCGATG GTAGTGTGGG 420

GTCTCCCCAT GCGAGAGTAG GGAACTGCCA GGCATCAATT TAAGCAGTAA ACTGATGTAA 480

AAATCAGTGG TTGTAAAAGA ATTCGGTGGA GCGGTAGTTC AGTTGGTTAG AATACCTGCC 540

TGTCACGCAG GGGGTCGCGG GTTCGAGTCC CGTCCGTTCC GCCACTTATT AAGAGCCCTG 600

AGCATTAGCT CAGGGCTTTT TCTTTGCCTG TCATTTAATT ATTGCTCAAT TCGCAAAAAT 660

CCTCTGCATT TTTCGCTCTT TTTCTACAAA GCGCTGAAGG TCATAATCCC CTCAGTTAAC 720

GAACATAGTG ATTAAAAAGA GGAATTCTAT GACTATCCCT GCATTCGGTT TAGGTACTTT 780

TCGCCTGAAA GACGACGTGG TTATCGCTTC TGTGAAAACG GCGCTTGAAC TGGGCTACCG 840

TACTGTCGAC ACTGCGCAGA TCTATGATAA TGAAGCGGCT GTTGGACAGG CGATTGCTGA 900

GAGCGGCGTA CCACGTAGCG AGCTGTACAT CACCACCAAG ATCTGGATTG AAAACCTTAG 960

CAAAGACAAA CTAATCCCAA GCCTGACAGA GAGCCTGAAA AAACTGCGTA CCGATTATGT 1020

CGATCTGACG CTGATCCACT GGCCATCTCC GGGTGATGCC GTGTCTGTCA AAAATTATGC 1080

TGGCATTGCT GGAAGCTAAA AACAAGGCCT GACTCGCGAA ATGGCAT 1127

>(12)C Oranienburg_NoNCBImatch

ATGTTTTGGC GGTTGAGAGA CGATTTTCAG CACTGATTCA GAGTTGAGTA CGCAATAATT 60

TGCGCAGCAG CAAGGCGGCA AGCGAAGGAA AGGAAGGAGC ATACAGAAGT ATGTGACTGA 120

CTTTACGAGC GCAGGCAACG CCGCTGATGC GATAAAGAAT TGCGTACTGA GCATAAAAGA 180

ATTTGCCTGG CGGCAACAGC GCGGTGGTCC CACCTGACCC CATGCCGAAC TCAGAAGTGA 240

AACGCCGTAG CGCCGATGGT AGTGTGGGGT CTCCCCATGC GAGAGTAGGG AACTGCCAGG 300

CATCAAATTA AGCAGTAAGC CGGTCATAAA CCGGTGGTTG TAAAAGAATT CGGTGGAGCG 360

G 361

>(13)X Schwarzengrund_Bredeney_Give_CP085812_CP082691_CP019174

CATCGCTCAA CGGATAAAAG GTACTCCGGG GATAACAGGC TGATACCGCC CAAGAGTTCA 60

TATCGACGGC GGTGTTTGGC ACCTCGATGT CGGCTCATCA CATCCTGGGG CTGAAGTAGG 120

TCCCAAGGGT ATGGCTGTTC GCCATTTAAA GTGGTACGCG AGCTGGGTTT AGAACGTCGT 180

GAGACAGTTC GGTCCCTATC TGCCGTGGGC GCTGGAGAAC TGAGGGGGGC TGCTCCTAGT 240

ACGAGAGGAC CGGAGTGGAC GCATCACTGG TGTTCGGGTT GTCATGCCAA TGGCACTGCC 300

CGGTAGCTAA ATGCGGAAGA GATAAGTGCT GAAAGCATCT AAGCACGAAA CTTGCCCCGA 360

GATGAGTTCT CCCTGAGACT TAGAGTCTCC TGAAGGAACG TTGAAGACGA CGACGTTGAT 420

AGGCCGGGTG TGTAAGCGCA GCGATGCGTT GAGCTAACCG GTACTAATGA ACCGTGAGGC 480

TTAACCTTAC AACGCCGAAG ATGTTTTGGC GGTTGAGAAG ACAACAATTT TCAGCTCAGA 540

TTCAGAGTTG AGTACGCAAT AATTTGCGCA GCAGCAAGGC GGCAAGCGAA GGAAAGGAAG 600

GAGCATACAG AAGTATGTGA CTGACTTTAC GAGCGCAGGC AACGCCGCTG ATGCGATAAA 660

GAATTGCGTA CTGAGCATAA AAGAATTTGC CTGGCGGCAC TAGCGCGGTG GTCCCACCTG 720

ACCCCATGCC GAACTCAGAA GTGAAACGCC GTAGCGCCGA TGGTAGTGTG GGGTCTCCCC 780

ATGCGAGAGT AGGGAACTGC CAGGCATCAA ATTAAGCAGT AAGCCGGTCA TAAACCGGTG 840

GTTGTAAAAG AATCCGGTGG AGCGGTAGTT CAGTTGGTTA GAATACCTGC CTGTCACGCA 900

GGGGGTCGCG GGTTCGAGTC CCGTCCGTTC CGCCACTTAT TAAGAGCCCT GAGCATTAGC 960

TCAGGGCTTT TTCTTTGCCT GTCATTTAAT TATTGCTCAA TTCGCAAAAA TCCTCTGCAT 1020

TTCTCGCTCT TTTTCTACAA AGCGCTGAAG GTCATAATCC CCTCAGTTAA CGAACATAGT 1080

GATTAAAAAG AGGAATTCTA TGACTATCCC TGCATTCGGT TTAGGTACTT TTCGCCTGAA 1140

AGACGACGTG GTTATCGCTT CTGTGAAAAC GGCGCTTGAA CTGGGCTACC GTGCTGTCGA 1200

CACTGCGCAA ATCTATGATA ATGAAGTTGC TGTTGGACAG GCGATTGCTG AGAGCGGCGT 1260

ACCGCGTAAT GAGCTGTACA TCACCACTAA GATCTGGATT GAAAACCTTA GCAAAGACAA 1320

GCTGATCCCA AGCCTGAAAG AGAGCCTGAA AAAACTGCGT ACCGA 1365

>(14)X Bovismorbificans_CP073715

CGGAAGAGAT AAGTGCTGAA AGCATCTAAG CACGAAACTT GCCCCGAGAT GAGTTCTCCC 60

TGAGACTTTA GGTCTCCTGA AGGAACGTTG AAGACGACGA CGTTGATAGG CCGGGTGTGT 120

AAGCGCAGCG ATGCGTTGAG CTAACCGGTA CTAATGAACC GTGAGGCTTA ACCTTACAAC 180

GCCGAAGATG TTTTGGCGGT TGAGAGATGA TTTTCAGCAC TGATTCCGGA TTTTCGGGAA 240

CGAAAGATTT TACGCTGAGG CAAGGCGGCA AATGAAGGAA AGGAAGGAGC ATACAGAAGT 300

ATGTGACTGA CTTTACGAAT GCAGCCAACG CAGCATCGGT GTAAAAGATT CGTTTCCGGC 360

AACAGAATTT GCCTGGCGGC CGTAGCGCGG TGGTCCCACC TGACCCCATG CCGAACTCAG 420

AAGTGAAACG CCGTAGCGCC GATGGTAGTG TGGGGTCTCC CCATGCGAGA GTAGGGAACT 480

GCCAGGCATC AATTTAAGCA GTAAACTGAT GTAAAAATCA GTGGTTGTAA AAAAATTCGG 540

TGGAGCGGTA GTTCAGTTGG TTAGAATACC TGCCTGTCAC GCAGGGGGTC GCGGGTTCGA 600

GTCCCGTCCG TTCCGCCACT TATTAAGAGC CCTGAGCATT AGCTCAGGGC TTTTTCTTTG 660

CCTGTCATTT AATTATTGCT CAATTCGCAA AAATCCTCTG CATTTCTCGC TCTTTTTCTA 720

CAAAGCGCTG AAGGTCATAA TCCCCTCAGT TAACGAACAT AGTGATTAAA AAGAGGAATT 780

CTATGACTAT CCCTGCATTC GGTTTAGGTA CTTTTCGCCT GAAAGACGAC GTGGTTATCG 840

CTTCTGTTAA AGCGGCGCTT GAACTAGGCT ACCGTGCTGT CGACACTGCG CAAATCTATG 900

ATAATGAAGC GGCTGTTGGA CAGGCGATTG CTGAGAGCGG CGTACCGCGT AATGAGCTGT 960

ACATCACCAC CAAGATCTGG ATTGAAAACC TTAGCAAAGA CAAGCTAATC CCAAGCCTGA 1020

AAGAGAGCCT GAAAAAACTG CGTACCGATT ATGTCGATCT GACACTGATC CACTGGCCAT 1080

CTCCGGGTGA TGCCGTGCCT GTCGAAGAAT TTATGCAGGC TTGCTGGAAG CTAAAAACAA 1140

GGCTGACTCG CGAATTGGCT CTCAACTTCA CATTCCGTGA TGGAAAGGTA TTGCCGCGGG 1200

GGCGCAGACA TATCGCACCA ACAGATGAGC TTCTCCGATT GCAATC 1246

>(15)C Gallinarum_pullorum_CP074215

ATGTTTTGGC GGATGAGAGA CGATTTTCAG CAGTGATTCA GAGTTGAGTA CGCAATAATT 60

TGCGCAGCAG CAAGGCGGCA AGCGAAGGAA AGGAAGGAGC ATACAGAAGT ATGTGACTGA 120

GTTTACGAGC GCAGGCAACG CCGCTGATGC GATAAAGAAT TTGCGTACAG AGCACAAAGA 180

ATTTGCCTGG CGGCAGTAGC GCGGTGGTCC CACCTGACCC CATGCCGAAC TCAGAAGTGA 240

AACGCCGTAG CGCCGATGGT AGTGTGGGGT CTCCCCATGC GAGAGTAGGG AACTGCCAGG 300

CATCAAATAA AACGAAGGGC CCTGTCGGAA GACAGGGCCT TTTGTTTTAT CTGTAGTCTG 360

TCGGTGAGCA CTCTCCTGAG GAGGAGAAAT CCGCCGGGAG CGGATTTGAA CGTTGCGCAG 420

CAACGGCCCG TAGGGTGGCG GGCAGGACGC CCGCCATAAA CTGCCAGGCA TCAAATTGCA 480

GTAAACCGGG ATGAAAATCC GGGTTGTGAC AAAGAAATTC GGTGGAGCGG 530

>(16)C Choleraesuis_CP074231

ATGTTTTGGC GGTTGAGAGA TGATTTTCAG CACTGATTCC GGATTTTCGG GAACGAAAGA 60

TTTTACGCTG AGGCAAGGCG GCAAATGAAG GAAAGGAAGG AGCATACTGA AGTATGTGAC 120

TGACTTTGCG AATGCAGCCA ACGCAGCATC GGTGTAAAAG ATTCGTTTCT GGCAACAGAA 180

TTTGCCTGGC GGCCGTAGCG CGGTGGTCCC ACCTGACCCC ATGCCGAACT CAGAAGTGAA 240

ACGCCGTAGC GCCGATGGTA GTGTGGGGTC TCCCCATGCG AGAGTAGGGA ACTGCCAGGC 300

ATCAAATTAA GCAGTAAACT GATGTAAAAG TCAGTGGTTG TAAAAGAATT CGGTGGAGCG 360

G 361

>(17)C Choleraesuis_NoNCBImatch

ATGTTTTGGC GGATGAGAGA CGATTTTCAG CACTGATTCA GAGTTGAGTA CGCAATAATT 60

TGCGCAGCAG CAAGGCGGCA AGCGAAGGAA AGGCAGGAGC ATACAGAAGT ATGTGACTGA 120

GTTTACGAGC GCAGGCAACG CCGCTGATGC GATAAAGAAT TGCGTACAGA GCACAAAGAA 180

TTTGCCTGGC GGCACTAGCG CGGTGGTCCC ACCTGACCCC ATGCCGAACT CAGAAGTGAA 240

ACGCCGTAGC GCCGATGGTA GTGTGGGGTC TCCCCATGCG AGAGTAGGGA ACTGCCAGGC 300

ATCAAATTAG AAGTATGGCA TTAACTTTCG GGGCAGTTCA CTACTCGACA GAGAGTAGGA 360

CAAAAGCGAA AGCTTTTGAA CGTTGCGAAG CAACGGCCCG AAGGGTGAAT CACGAAGTGA 420

TTCATAAACT GCCAGGCATC AAATTAAGCA GTAAACTGAT GTAAAAATCA GTGGTTGTAA 480

AAGAATTCGG TGGAGCGG 498

>(18)C Paratyphi_B_CP074611

ATGTTTTGGC GGTTGAGAAG ACGATTTTCA GCAGTGATTC AGAGTTGAGT ACGCAATAAT 60

TTGCGCAGCA GCAAGGCGGC AAGCGAAGGA AAGGAAGGAG CATACAGAAG TATGTGACTG 120

AGTTTACGAG CGCAGGCAAC GCCGCTGATG CGATAAAGAA TTGCGTACAG AGCACAAAGA 180

ATTTGCCTGG CGGCACTAGC GCGGTGGTCC CACCTGACCC CATGCCGAAC TCAGAAGTGA 240

AACGCCGTAG CGCCGATGGT AGTGTGGGGT CTCCCCATGC GAGAGTAGGG AACTGCCAGG 300

CATCAAATTA GAAGTATGGC ATTAACTTTC GGGGCAGTTC ACTACTCGAC AGAGAGTAGG 360

ACAAAAGCGA AAGCTTTTGA ACGTTGCGAA GCAACGGCCC GAAGGGTGAA TCACGAAGTG 420

ATTCATAAAC TGCCAGGCAT CAAATTAAGC AGTAAACTGA TGTAAAAATC AGTGGTTGTA 480

AAAGAATTCG GTGGAGCGG 499

>(19)C Dublin_NoNCBImatch

ATGTTTTGGC GGATGAGAGA CGATTTTCAG CACTGATTCA GAGTTGAGTA CGCAATAATT 60

TGCGCAGCAG CAAGGCGGCA AGCGAAGGAA AGGAAGGAGC ATACAGAAGT ATGTGACTGA 120

GTTTACGAGC GCAGGCAACG CAGCGGATGC GATAAAGAAT TGCGTACAGA GCACAAAGAA 180

TTTGCCTGGC GGCACTAGCG CGGTGGTCCC ACCTGACCCC ATGCCGAACT CAGAAGTGAA 240

ACGCCGTAGC GCCGATGGTA GTGTGGGGTC TCCCCATGCG AGAGTAGGGA ACTGCCAGGC 300

ATCAAATTAG AAGTATGGCA TTAACTTTCG GGGCAGTTCA CTACTCGACA GAGAGTAGGA 360

CAAAAGCGAA AGCTTTTGAA CGTTGCGAAG CAACGGCCCG AAGGGTGAAT CACGAAGTGA 420

TTCATAAACT GCCAGGCATC AAATTGCAGT AAACCGGGAT GAAAATCCGG GTTGTGACAA 480

AGAAATTCGG TGGAGCGG 498

>(20)C Paratyphi_B_CP074222

ATGTTTTGGC GGATGAGAGA CGATTTTCAG CACTGATTCA GAGTTGAGTA CGCAATAATT 60

TACGCAGCAG CAAGGCGGCA AGCGAAGGAA AGGAAGGAGC ATACAGAAGT ATGTGACTGA 120

GTTTACGAGC GCAGGCAACG CAGCGGATGC GATAAAGAAT TGCGTACAGA GCACAAAGAA 180

TTTGCCTGGC GGCACTAGCG CGGTGGTCCC ACCTGACCCC ATGCCGAACT CAGAAGTGAA 240

ACGCCGTAGC GCCGATGGTA GTGTGGGGTC TCCCCATGCG AGAGTAGGGA ACTGCCAGGC 300

ATCAAATTAG AAGTATGGCA TTAACTTTCG GGGCAGTTCA CTACTCGACA GAGAGTAGGA 360

CAAAAGCGAA AGCTTTTGAA CGTTGCGAAG CAACGGCCCG AAGGGTGAAT CACGAAGTGA 420

TTCATAAACT GCCAGGCATC AAATTGCAGT AAACCGGGAT GAAAATCCGG GTTGTGACAA 480

AGAAATTCGG TGGAGCGG 498

>(21)C Typhimurium_CP040458

ATGTTTTGGC GGTTGAGAGA CGATTTTCAG CACTGATTCA GATTACATCA GAACGTAAAA 60

ACGGTCTGAT AACAGAATTT GCCTGGCGGC ACTAGCGCGG TGGTCCCACC TGACCCCATG 120

CCGAACTCAG AAGTGAAACG CCGTAGCGCC GATGGTAGTG TGGGGTCTCC CCATGCGAGA 180

GTAGGGAACT GCCAGGCATC AAATTAGAAG TATGGCATTA ACTTTCGGGG CAGTTCACTA 240

CTCGACAGAG AGTAGGACAA AAGCGAAAGC TTTTGAACGT TGCGAAGCAA CGGCCCGAAG 300

GGTGAATCAC GAAGTGATTC ATAAACTGCC AGGCATCAAA TTAAGCAGTA AACTGATGTA 360

AAAATCAGTG GTTGTAAAAG AATTCGGTGG AGCGG 395

>(22)C Paratyphi-B_CP074225

ATGTTTTGGC GGTTGAGAGA AGATTTTCAG CACGATTCAG AGTTGAGTAC GCAATAATTT 60

GCGCAGCAGC AAGCGAAGGA AAGGAAGGAG CATACAGAAG TATGTGACTG ACTTTACGAG 120

CGCAGGCAAC GCCGCTGATG CGATAAAGAA TTGCGTACTG AGCACAAAGA ATTTGCCTGG 180

CGGCACTAGC GCGGTGGTCC CACCTGACCC CATGCCGAAC TCAGAAGTGA AACGCCGTAG 240

CGCCGATGGT AGTGTGGGGT CTCCCCATGC GAGAGTAGGG AACTGCCAGG CATCAAATTA 300

GAAGTATGGC ATTAACTTTC GGGGCAGTTC ACTACTCGAC AGAGAGTAGG ACAAAAGCGA 360

AAGCTTTTGA ACGTTGCGAA GCAACGGCCC GAAGGGTGAA TCACGAAGTG ATTCATAAAC 420

TGCCAGGCAT CAAATTAAGC AGTAAACTGA TGTAAAAATC AGTGGTTGTA AAAGAATTCG 480

GTGGAGCGG 489

>(23)C Yovokome_CP019418

ATGTTTTGGC GGTTGAGAAG ACGATTTTCA GCACTGATTC AGATTACATC AGAACGTAAA 60

AACGGTCTGA TAACAGAATT TGCCTGGCGG CACTAGCGCG GTGGTCCCAC CTGACCCCAT 120

GCCGAACTCA GAAGTGAAAC GCCGTAGCGC CGATGGTAGT GTGGGGTCTC CCCATGCGAG 180

AGTAGGGAAC TGCCAGGCAT CAAATTAGAA GTATGGCATT AACTTTCGGG GCAGTTCACT 240

ACTCGACAGA GAGTAGGACA AAAGCGAAAG CTTTTGAACG TTGCGAAGCA ACGGCCCGAA 300

GGGTGAATCA CGAAGTGATT CATAAACTGC CAGGCATCAA ATTAAGCAGT AAACTGATGT 360

AAAAATCAGT GGTTGTAAAA GAATTCGGTG GAGCGG 396

>(24)C Newport_CP025232

CTGTTTTGGC GGATGAGAGA CGATTTTCAG CTCAGATTCA GATTACATCA GAACGTAAAA 60

ACGGTCTGAT AACAGAATTT GCCTGGCGGC ACTAGCGCGG TGGTCCCACC TGACCCCATG 120

CCGAACTCAG AAGTGAAACG CCGTAGCGCC GATGGTAGTG TGGGGTCTCC CCATGCGAGA 180

GTAGGGAACT GCCAGGCATC AAATTAGAAG TATGGCATTA ACTTTCGGGG CAGTTCACTA 240

CTCGACAGAG AGTAGGACAA AAGCGAAAGC TTTTGAACGT TGCGAAGCAA CGGCCCGAAG 300

GGTGAATCAC GAAGTGATTC ATAAACTGCC AGGCATCAAA TTAAGCAGTA AACTGATGTA 360

AAAATCAGTG GTTGTAAAAG AATTCGGTGG AGCGG 395

>(25)X Tennessee_Montevideo_CP007505_CP030029

CGGAAGAGAT AAGTGCTGAA AGCATCTAAG CACGAAACTT GCCCCGAGAT GAGTTCTCCC 60

TGAGACTTAG AGTCTCCTGA AGGAACGTTG AAGACGACGA CGTTGATAGG CCGGGTGTGT 120

AAGCGCAGCG ATGCGTTGAG CTAACCGGTA CTAATGAACC GTGAGGCTTA ACCTTACAAC 180

GCCGAAGATG TTTTGGCGGA TGAGAGACGA TTTTCAGCAC TGATTCAGAT TACATCAGAA 240

CGTAAAAACG GTCTGATAAC AGAATTTGCC TGGCGGCCGT AGCGCGGTGG TCCCACCTGA 300

CCCCATGCCG AACTCAGAAG TGAAACGCCG TAGCGCCGAT GGTAGTGTGG GGTCTCCCCA 360

TGCGAGAGTA GGGAACTGCC AGGCATCAAA TTAAGCAGTA AACTGATGTA AAAATCAGTG 420

GTTGTAAAAG AATTCGGTGG AGCGGTAGTT CAGTTGGTTA GAATACCTGC CTGTCACGCA 480

GGGGGTCGCG GGTTCGAGTC CCGTCCGTTC CGCCACTTAT TAAGAGCCCT GAGCATTAGC 540

TCAGGGCTTT TTCTTTGCAT GTCATTTAAT TATTGCTCAA TTCGCAAAAA TCCTCTGCAT 600

TTCTCGCTCT TTTTCTGCAA AGCGCTGAAG GTCATAATCC CCTCAGTTAA CGAACATAGT 660

GATTAAAAAG AGGAATTCTA TGACTATCCC TGCATTCGGT TTAGGTACTT TTCGCCTGAA 720

AGACGACGTG GTTATCGCTT CTGTTAAAAC GGCGCTTGAA CTGGGCTACC GTGCTGTCGA 780

CACTGCACAG ATCTATGATA ATGAAGCGGC TGTTGGACAG GCGATTGCTG AAAGCGGTGT 840

ACCACGTAAT GAGCTGTACA TCACCACCAA GATCTGGATT GAAAACCTTA GCAAAGACAA 900

GCTAATCCCA AGCCTGAAAG AGAGCCTGAA AAAACTGCGT ACCGATTATG TCGATCTGAC 960

GCTGATCCAC TGGCCATCTC CGGGTGATGC CGTGTCTGTC GAAGAATTTA TGCAGGCATT 1020

GCTGGAAGCT AAAAAACAAG GGCTGACTCG CGAAATTGGC ATTTCAAACT TCACCATTCC 1080

GTTGATGGAA AAGGCTATTG CCGCGGTGGG CGCAGACCAT ATCGCCACCA ACCAGATTGA 1140

GCTATCTCCG TATCTGCAGA ATCGT 1165

>(26)C Fresno_noNCBImatch

ATGTTTTGGC GGATGAGAAG ACGATTTTCA GCTCAGATTC AGAGTCCGAA GGATTTTACG 60

CTGAGACAAG GCGGCAAACG CAGCGACGGA AGGAGCATAC AGAAGTATGT GACTGACGCT 120

CGCAAGAGCA GCCAACGCCG TATCAGTGTA AAAGACACAG GACAGAGCAC AAAGAATTTG 180

CCTGGCGGCA CTAGCGCGGT GGTCCCACCT GACCCCATGC CGAACTCAGA AGTGAAACGC 240

CGTAGCGCCG ATGGTAGTGT GGGGTCTCCC CATGCGAGAG TAGGGAACTG CCAGGCATCA 300

AATTAGAAGT ATGGCATTAA CTTTCGGGGC AGTTCACTAC TCGACAGAGA GTAGGACAAA 360

AGCGAAAGCT TTTGAACGTT GCGAAGCAAC GGCCCGAAGG GTGAATCACG AAGTGATTCA 420

TAAACTGCCA GGCATCAAAT TAAGCAGTAA GCCGGTCATA GATCGGTGGT TGTAAAAGAA 480

TTCGGTGGAG CGG 493

>(27)C Worthington_CP039509

ATGTTTTGGC GGATGAGAAG ACGATTTTCA GCACTGATTC CGGATTTTCG GGAACGAAAG 60

ATAAAAGATT CGTTTCCGGC AACAGAATTT GCCTGGCGGC ACTAGCGCGG TGGTCCCACC 120

TGACCCCATG CCGAACTCAG AAGTGAAACG CCGTAGCGCC GATGGTAGTG TGGGGTCTCC 180

CCATGCGAGA GTAGGGAACT GCCAGGCATC AAATTAAGCA GTAAACTGAT GTAAAAATCA 240

GTGGTTGTAA AAGAATTCGG TGGAGCGG 268

>(28)C Anatum_noNCBImatch

ATGTTTTGGC GGATTGAGAA GACAACAATT TTCAGCTCAG ATTCAGAGTC CGAAGGATTT 60

TACGCTGAGA CAAGGCGGCA AACGCAGCGA CGGAAGGAGC ATACAGAAGT ATGTGACTGA 120

CGCTCGCAAG AGCAGCCAAC GCCGTATCAG TGTAAAAGAC ACAGGACAGA GCACAAAGAA 180

TTTGCCTGGC GGCACTAGCG CGGTGGTCCC ACCTGACCCC ATGCCGAACT CAGAAGTGAA 240

ACGCCGTAGC GCCGATGGTA GTGTGGGGTC TCCCCATGCG AGAGTAGGGA ACTGCCAGGC 300

ATCAAATTAG AAGTATGGCA TTAACTTTCG GGGCAGTTCA CTACTCGACA GAGAGTAGGA 360

CAAAAGCGAA AGCTTTTGAA CGTTGCGAAG CAACGGCCCG AAGGGTGAAT CACGAAGTGA 420

TTCATAAACT GCCAGGCATC AAATTAAGCA GTAAACTTGA TGTAAAAATC AGTGGTTGTA 480

AAAGAATTCG GTGGAGCGG 499

>(29)C Derby_CP026609

ATGTTTTGGC GGATGAGAGA CGATTTTCAG CAGTGATTCA GAGTTGAGTA CGCAATGATT 60

TGCGCAGCAG CAAGGCGGCA AGCGAAGGAA AGGAAGGAGC ATACTGAAGT ATGTGACTGA 120

CTTTACGAGC GCAGGCAACG CCGCTGATGC GATAAAGAAT TGCGTACAGA GCACAAAGAA 180

TTTGCCTGGC GGCCGTAGCG CGGTGGTCCC ACCTGACCCC ATGCCGAACT CAGAAGTGAA 240

ACGCCGTAGC GCCGATGGTA GTGTGGGGTC TCCCCATGCG AGAGTAGGGA ACTGCCAGGC 300

ATCAAATTAG AAGTATGGCA TTAACTTTCG GGGCAGTTCA CTACTCGACA GAGAGTAGGA 360

CAAAAGCGAA AGCTTTTGAA CGTTGCGAAG CAACGGCCCG AAGGGTGAAT CACGAAGTGA 420

TTCATAAACT GCCAGGCATC AAATTAAGCA GTAAACTGAT GTAAAAATCA GTGGTTGTAA 480

AAGAATTCGG TGGAGCGG 498

>(30)C Infantis_noNCBImatch

ATGTTTTGGC GGTTGAGAGA TGATTTTCAG CACTGATTCA GAGTTGAGTA CGCAATAATT 60

TGCGCAGCAG CAAGGCGGCA AGCGAAGGAA AGGAAGGAGC ATACAGAAGT ATGTGACTGA 120

CTTTACGAGC GCAGGCAACG CCGCTGATGC GATAAAGAAT TGCGTACTGA GCATAAAAGA 180

ATTTGCCTGG CGGCACTAGC GCGGTGGTCC CACCTGACCC CATGCCGAAC TCAGAAGTGA 240

AACGCCGTAG CGCCGATGGT AGTGTGGGGT CTCCCCATGC GAGAGTAGGG AACTGCCAGG 300

CATCAAATTA GAAGTATGGC ATTAACTTTC GGGGCAGTTC ACTACTCGAC AGAGAGTAGG 360

ACAAAAGCGA AAGCTTTTGA ACGTTGCGAA GCAACGGCCC GAAGGGTGAA TCACGAAGTG 420

ATTCATAAAC TGCCAGGCAT CAATTTAAGC AGTAAACTGA TGTAAAAATC AGTGGTTGTA 480

AAAGAATTCG GTGGAGCGG 499

>(31)C Paratyphi-B_CP074221

ATGTTTTGGC GGATGAGAGA CGATTTTCAG CACTGATTCA GAGTTGAGTA CGCAATAATT 60

TGCGCAGCAG CAAGGCGGCA AGCGAAGGAA AGGAAGGAGC ATACAGAAGT ATGTGACTGA 120

GTTTACGAGC GCAGGCAACG CCGCTGATGC GATAAAGAAT TGCGTACTGA GCATAAAAGA 180

ATTTGCCTGG CGGCCGTAGC GCGGTGGTCC CACCTGACCC CATGCCGAAC TCAGAAGTGA 240

AACGCCGTAG CGCCGATGGT AGTGTGGGGT CTCCCCATGC GAGAGTAGGG AACTGCCAGG 300

CATCAAATTA GAAGTATGGC ATTAACTTTC GGGGCAGTTC ACTACTCGAC AGAGAGTAGG 360

ACAAAAGCGA AAGCTTTTGA ACGTTGCGAA GCAACGGCCC GAAGGGTGAA TCACGAAGTG 420

ATTCATAAAC TGCCAGGCAT CAAATTAAGC AGTAAACTGA TGTAAAAATC AGTGGTTGTA 480

AAAGAATTCG GTGGAGCGG 499

>(32)C Derby_CP022494

ATGTTTTGGC GGATGAGAGA CGATTTTCAG CACTGATTCA GAGTTGAGTA CGCAATAATT 60

TGCGCAGCAG CAAGGCGGCA AGCGAAGGAA AGGAAGGAGC ATACAGAAGT ATGTGACTGA 120

CTTTACGAGC GCAGGCAACG CCGCTGATGC GATAAAGAAT TGCGTACAGA GCACAAAGAA 180

TTTGCCTGGC GGCCGTAGCG CGGTGGTCCC ACCTGACCCC ATGCCGAACT CAGAAGTGAA 240

ACGCCGTAGC GCCGATGGTA GTGTGGGGTC TCCCCATGCG AGAGTAGGGA ACTGCCAGGC 300

ATCAAATTAG AAGTATGGCA TTAACTTTCG GGGCAGTTCA CTACTCGACA GAGAGTAGGA 360

CAAAAGCGAA AGCTTTTGAA CGTTGCGAAG CAACGGCCCG AAGGGTGAAT CACGAAGTGA 420

TTCATAAACT GCCAGGCATC AAATTAAGCA GTAAACTGAT GTAAAAATCA GTGGTTGTAA 480

AAGAATTCGG TGGAGCGG 498

>(33)X Derby_California_CP08267_CP028900

TGCGGAAGAG ATAAGTGCTG AAAGCATCTA AGCACGAAAC TTGCCCCGAG ATGAGTTCTC 60

CCTGAGACTT AGAGTCTCCT GAAGGAACGT TGAAGACGAC GACGTTGATA GGCCGGGTGT 120

GTAAGCGCAG CGATGCGTTG AGCTAACCGG TACTAATGAA CCGTGAGGCT TAACCTTACA 180

ACGCCGAAGA TGTTTTGGCG GTTGAGAGAC GATTTTCAGC AGTGATTCAG AGTTGAGTAC 240

GCAATAATTT GCGCAGCAGC AAGGCGGCAA GCGAAGGAAA GGAAGGAGCA TACAGAAGTA 300

TGTGACTGAC TTTACGAGCG CAGGCAACGC CGCTGATGCG ATAAAGAATT GCGTACAGAG 360

CACAAAGAAT TTGCCTGGCG GCAGTAGCGC GGTGGTCCCA CCTGACCCCA TGCCGAACTC 420

AGAAGTGAAA CGCCGTAGCG CCGATGGTAG TGTGGGGTCT CCCCATGCGA GAGTAGGGAA 480

CTGCCAGGCA TCAAATTAGA AGTATGGCAT TAACTTTCGG GGCAGTTCAC TACTCGACAG 540

AGAGTAGGAC AAAAGCGAAA GCTTTTGAAC GTTGCGAAGC AACGGCCCGA AGGGTGAATC 600

ACGAAGTGAT TCATAAACTG CCAGGCATCA AATTGCAGTA AACCGGGATG AAAATCCGGG 660

TTGTGACAAA GAAATTCGGT GGAGCGGTAG TTCAGTTGGT TAGAATACCT GCCTGTCACG 720

CAGGGGGTCG CGGGTTCGAG TCCCGTCCGT TCCGCCACTT ATTAAGAGCC CTGAGCATTA 780

GCTCAGGGCT TTTTCTTTGC ATGTCATTTA ATTATTGCTC AATTCGCAAA AATCCTCTGC 840

ATTTCTCGCT CTTTTTCTGC AAAGCGCTGA AGGTCATAAT CCCCTCAGTT AACGAACATA 900

GTGATTAAAA AGAGGAATTC TATGACTATC CCTGCATTCG GTTTAGGTAC TTTTCGCCTG 960

AAAGACGACG TGGTTATCGC TTCTGTTAAA ACGGCGCTTG AACTAGGCTA CCGTGCTGTC 1020

GACACTGCGC AAATCTATGA TAATGAAGCG GCTGTTGGAC AGGCGATTGC TGAGAGCGGC 1080

GTACCGCGTA ATGAGCTGTA CATCACCACC AAGATCTGGA TTGAAAACCT TAGCAAAGAC 1140

AGCTAATCCC AGCCTGAAAG AGAGCCTGAA AAACTGCGTA CCGATATGTC GATCTGAG 1198

>(34)C Newport_CP015924

ATGTTTTGGC GGATGAGAGA AGATTTTCAG CACTGATTCA GAGTTGAGTA CGCAATAATT 60

TGCGCAGCAG CAAGGCGGCA AGCGAAGGAA AGGAAGGAGC ATACAGAAGT ATGTGACTGA 120

CTTTACGAGC GCAGGCAACG CCGCTGATGC GATAAAGAAT TGCGTACAGA GCACAAAAGA 180

ATTTGCCTGG CGGCACTAGC GCGGTGGTCC CACCTGACCC CATGCCGAAC TCAGAAGTGA 240

AACGCCGTAG CGCCGATGGT AGTGTGGGGT CTCCCCATGC GAGAGTAGGG AACTGCCAGG 300

CATCAAATTA GAAGTATGGC ATTAACTTTC GGGGCAGTTC ACTACTCGAC AGAGAGTAGG 360

ACAAAAGCGA AAGCTTTTGA ACGTTGCGAA GCAACGGCCC GAAGGGTGAA TCACGAAGTG 420

ATTCATAAAC TGCCAGGCAT CAAATTGCAG TAAACCGGGA TGAAAATCCG GGTTGTGACA 480

AAGAAATTCG GTGGAGCGG 499

>(35)C Kentucky_CP026327

ATGTTTTGGC GGATGAGAGA AGATTTTCAG CACTGATTCA GAGTTGAGTA CGCAATAATT 60

TGCGCAGCAG CAAGGCGGCA AGCGAAGGAA AGGAAGGAGC ATACAGAAGT ATGTGACTGA 120

GTTTACGAGC GCAGGCAACG CCGCTGATGC GATAAAGAAT TGCGTACTGA GCACAAAAGA 180

ATTTGCCTGG CGGCACTAGC GCGGTGGTCC CACCTGACCC CATGCCGAAC TCAGAAGTGA 240

AACGCCGTAG CGCCGATGGT AGTGTGGGGT CTCCCCATGC GAGAGTAGGG AACTGCCAGG 300

CATCAAATTA GAAGTATGGC ATTAACTTTC GGGGCAGTTC ACTACTCGAC AGAGAGTAGG 360

ACAAAAGCGA AAGCTTTTGA ACGTTGCGAA GCAACGGCCC GAAGGGTGAA TCACGAAGTG 420

ATTCATAAAC TGCCAGGCAT CAAATTGCAG TAAACCGGGA TGAAAATCCG GGTTGTGACA 480

AAGAAATTCG GTGGAGCGG 499

>(36)C Paratyphi-B_CP074221

ATGTTTTGGC GGTTAAGAGA AGATTTTCAG CACTGATTCA GATTACATCA GAACGTAAAA 60

ACGGTCTGAT AACAGAATTT GCCTGGCGGC ACTAGCGCGG TGGTCCCACC TGACCCCATG 120

CCGAACTCAG AAGTGAAACG CCGTAGCGCC GATGGTAGTG TGGGGTCTCC CCATGCGAGA 180

GTAGGGAACT GCCAGGCATC AAATTAGAAG TATGGCATTA ACTTTCGGGG CAGTTCACTA 240

CTCGACAGAG AGTAGGACAA AAGCGAAAGC TTTTGAACGT TGCGAAGCAA CGGCCCGAAG 300

GGTGAATCAC GAAGTGATTC ATAAACTGCC AGGCATCAAA TTAAGCAGTA AACTGATGTA 360

AAAATCAGTG GTTGTAAAAG AATTCGGTGG AGCGG 395

>(37)X Mbandaka_Lubbock_Typhimurium_CP033343_CP032814_CP011365

AGAGATAAGT GCTGAAAGCA TCTAAGCACG AAACTTGCCC CGAGATGAGT TCTCCCTGAG 60

ACTTAGAGTC TCCTGAAGGA ACGTTGAAGA CGACGACGTT GATAGGCCGG GTGTGTAAGC 120

GCAGCGATGC GTTGAGCTAA CCGGTACTAA TGAACCGTGA GGCTTAACCT TACAACGCCG 180

AAGATGTTTT GGCGGTTGAG AGAAGATTTT CAGCACTGAT TCAGAGTTGA GTATGCAATA 240

ATTTGCGCAG CAGCAAGGCG GCAAGCGAAG GAAAGGAAGG AGCATACAGA AGTATGTGAC 300

TGACTTTACG AGCGCAGGCA ACGCCGCTGA TGCGATAAAG AATTGCGTAC AGAGCACAAA 360

AGAATTTGCC TGGCGGCCGT AGCGCGGTGG TCCCACCTGA CCCCATGCCG AACTCAGAAG 420

TGAAACGCCG TAGCGCCGAT GGTAGTGTGG GGTCTCCCCA TGCGAGAGTA GGGAACTGCC 480

AGGCATCAAA TTAGAAGTAT GGCATTAACT TTCGGGGCAG TTCACTACTC GACAGAGAGT 540

AGAACAAAAG CGAAAGCTTT TGAACGTTGC GAAGCAACGG CCCGAAGGGT GAATCACGAA 600

GTGATTCATA AACTGCCAGG CATCAAATTG CAGTAAACCG GGATGAAAAT CCGGGTTGTG 660

ACAAAGAAAT TCGGTGGAGC GGTAGTTCAG TTGGTTAGAA TACCTGCCTG TCACGCAGGG 720

GGTCGCGGGT TCGAGTCCCG TCCGTTCCGC CACTTATTAA GAGCCCTGAG CATTAGCTCA 780

GGGCTTTTTC TTTGCATGTC ATTTAATTAT TGCTCAATTC GCAAAAATCC TCTGCATGTC 840

TCGCTCTTTT TCTGCAAAGC GCTGAAGGTC ATAATCCCCT CAGTTAACGA ACATAGTGAT 900

TAAAAAGAGG AATTCTATGA CTATCCCTGC ATTCGGTTTA GGTACTTTTC GCCTGAAAGA 960

CGACGTGGTT ATCGCTTCTG TGAAAACGGC GCTTGAACTG GGCTACCGTG CTGTCGACAC 1020

TGCGCAAATC TATGATAATG AAGCGGCTGT TGGACAGGCG ATTGCTGAGA GCGGTGTACC 1080

GCGTAATGAG CTGTACATCA CCACCAAGAT CTGGATTGAA AACCTTAGCA AAGACAAGCT 1140

AATCCCAAGC CTG 1153

>(38)C Montevideo_CP017978

ATGTTTTGGC GGATTGAGAG ATGATTTTCA GCACTGATTC AGAGTTGAGT ACGCAATAAT 60

TTGCGCAGCA GCAAGGCGGC AAGCGAAGGA AAGGAAGGAG CATACAGAAG TATGTGACTG 120

ACTTTACGAG CGCAGGCAAC GCCGCTGATG CGATAAAGAA TTGCGTACTG AGCATAAAAG 180

AATTTGCCTG GCGGCAGTAG CGCGGTGGTC CCACCTGACC CCATGCCGAA CTCAGAAGTG 240

AAACGCCGTA GCGCCGATGG TAGTGTGGGG TCTCCCCATG CGAGAGTAGG GAACTGCCAG 300

GCATCAAATT AAGCAGTAAG CCGGTCATAA ACCGGTGGTT GTAAAAGAAT TCGGTGGAGC 360

GG 362

>(39)C Montevideo_CP020912

ATGTTTTGGC GGATGAGAGA CGATTTTCAG CACTGATTCA GAGTTGAGTA CGCAATAATT 60

TGCGCAGCAG CAAGGCGGCA AGCGAAGGAA AGGAAGGAGC ATACAGAAGT ATGTGACTGA 120

GTTTACGAGC GCGGGCAACG CCGCTGATGC GATAAAGAAT TGCGTACTGA GCATAAAAGA 180

ATTTGCCTGG CGGCACTAGC GCGGTGGTCC CACCTGACCC CATGCCGAAC TCAGAAGTGA 240

AACGCCGTAG CGCCGATGGT AGTGTGGGGT CTCCCCATGC GAGAGTAGGG AACTGCCAGG 300

CATCAAATTA AGCAGTAAGC CGGTCATAGA CTGGTGGTTG TAAAAAAATT CGGTGGAGCG 360

G 361

>(40)C Montevideo_CP032816

ATGTTTTGGC GGACTGAGAG ACGATTTTCA GCACTGATTC AGAGTTGAGT ACGCAATAAT 60

TTGCGCAGCA GCAAGGCGGC AAGCGAAGGA AAGGAAGGAG CATACAGAAG TATGTGACTG 120

ACTTTACGAG CGCAGGCAAC GCCGCTGATG CGATAAAGAA TTGCGTACTG AGCATAAAAG 180

AATTTGCCTG GCGGCAGTAG CGCGGTGGTC CCACCTGACC CCATGCCGAA CTCAGAAGTG 240

AAACGCCGTA GCGCCGATGG TAGTGTGGGG TCTCCCCATG CGAGAGTAGG GAACTGCCAG 300

GCATCAAATT AAGCAGTAAG CCGGTCATAA ACCGGTGGTT GTAAAAGAAT TCGGTGGAGC 360

GG 362

>(41)C Newport_CP015923

ATGTTTTGGC GGATGAGAGA AGATTTTCAG CACTGATTCA GAGTTGAGTA CGCAATAATT 60

TGCGCAGCAG CAAGGCGGCA AGCGAAGGAA AGGAAGGAGC ATACAGAAGT ATGTGACTGA 120

CTTTACGAGC GCAGGCAACG CCGCTGATGC GATAAAGAAT TGCGTACAGA GCACAAAGAA 180

TTTGCCTGGC GGCACTAGCG CGGTGGTCCC ACCTGACCCC ATGCCGAACT CAGAAGTGAA 240

ACGCCGTAGC GCCGATGGTA GTGTGGGGTC TCCCCATGCG AGAGTAGGGA ACTGCCAGGC 300

ATCAAATTAG AAGTATGGCA TTAACTTTCG GGGCAGTTCA CTACTCGACA GAGAGTAGGA 360

CAAAAGCGAA AGCTTTTGAA CGTTGCGAAG CAACGGCCCG AAGGGTGAAT CACGAAGTGA 420

TTCATAAACT GCCAGGCATC AAATTGCAGT AAACCGGGAT GAAAATCCGG GTTGTGACAA 480

AGAAATTCGG TGGAGCGG 498

>(42)C Abony_CP007534

ATGTTTTGGC GGATGAGAGA CGATTTTCAG CACTGATTCC GGATTTTCGG GAACGAAAGA 60

TTTTACGCTG AGGCAAGGCG GCAAATGAAG GAAAGGAAGG AGCATACAGA AGTATGTGAC 120

TGACTTTGCG AATGCAGCCA ACGCAGCATC GGTGTAAAAG ATTCGTTTCC GGTAACAGAA 180

TTTGCCTGGC GGCACTAGCG CGGTGGTCCC ACCTGACCCC ATGCCGAACT CAGAAGTGAA 240

ACGCCGTAGC GCCGATGGTA GTGTGGGGTC TCCCCATGCG AGAGTAGGGA ACTGCCAGGC 300

ATCAAATTAG AAGTATGGCA TTAACTTTCG GGGCAGTTCA CTACTCGACA GAGAGTAGGA 360

CAAAAGCGAA AGCTTTTGAA CGTTGCGAAG CAACGGCCCG AAGGGTGAAT CACGAAGTGA 420

TTCATAAACT GCCAGGCATC AAATTGCAGT AAACCGGGAT GAAAATCCGG GTTGTGACAA 480

AGAAATTCGG TGGAGCGG 498

>(43)X Corvallis_CP051307

TGCGGAAGAG ATAAGTGCTG AAAGCATCTA AGCACGAAAC TTGCCCCGAG ATGAGTTCTC 60

CCTGAGACTT AGAGTCTCCT GAAGGAACGT TGAAGACGAC GACGTTGATA GGCCGGGTGT 120

GTAAGCGCAG CGATGCGTTG AGCTAACCGG TACTAATGAA CCGTGAGGCT TAACCTTACA 180

ACGCCGAAGA TGTTTTGGCG GAAGAGAGAC GATTTTCAGC ACTGATTCAG ATTACATCAG 240

AACGTAAAAA CGGTCTGATA ACAGAATTTG CCTGGCGGCA CTAGCGCGGT GGTCCCACCT 300

GACCCCATGC CGAACTCAGA AGTGAAACGC CGTAGCGCCG ATGGTAGTGT GGGGTCTCCC 360

CATGCGAGAG TAGGGAACTG CCAGGCATCA AATTAAGCAG TAAACTGATG TAAAAATCAG 420

TGGTTGTAAA AGAATTCGGT GGAGCGGTAG TTCAGTTGGT TAGAATACCT GCCTGTCACG 480

CAGGGGGTCG CGGGTTCGAG TCCCGTCCGT TCCGCCACTT ATTAAGAGCC CTGAGCATTA 540

GCTCAGGGCT TTTTCTTTGC CTGCGATTTA ATTATTGCTC AATTCGCAAA AATCCTCTGC 600

ATTTCTCGCT CTTTTTCTAC AAAGCGCTGA AGGTCATAAT CCCCTCAGTT AACGAACATA 660

GTGATTAAAA AGAGGAATTC TATGACTATC CCTGCATTCG GTTTAGGTAC TTTTCGCCTG 720

AAAGACGACG TGGTTATCGC TTCTGTTAAA ACGGCGCTTG AACTAGGCTA CCGTGCTGTC 780

GACACTGCGC AAATCTATGA TAATGAAGCG GCTGTTGGAC AGGCGATTGC TGAGAGCGGC 840

GTACCGCGTA ATGAGCTGTA CATCACCACC AAGATCTGGA TTGAAAACCT TAGCAAAGAC 900

AAGCTAATCC CAAGCCTGAA AGAGAGCCTG AAAAAACTGC GTACCGATTA TGTCGATCTG 960

ACGCTGATCC ACTGGCCATC TCCGGGTGAT GCCGTGTCTG TCGAAGAATT TATGCAGGCA 1020

TTGCTGGAAG CTAAAAAACA AGGGCTGACT CGCGAAATTG GCATCTCAAA CTTCACTATT 1080

CCGTTGATGG AAAAGGCTAT TGCCGCGGTG GGCGCAGACA TATCGCCACC AACCAGATTG 1140

AGCTATCTCC GATCTGCAGA ATCGTAAGGG GGTGACTGGG AAAAAGGCGC 1190

>(44)C Senftenberg_CP016837

ATGTTTTGGC GGATGAGAGA CGATTTTCAG CACTGATTCC GGATTTTCGG GAACGAAAGA 60

TTTTACGCTG AGGCAAGGCG GCAAATGAAG GAAAGGAAGG AGCATACAGA AGTATGTGAC 120

TGACTTTACG AATGCAGCCA ACGCAGCATC GGTGTAAAAG ATTCGTTTCC GGCAACAGAA 180

TTTGCCTGGC GGCACTAGCG CGGTGGTCCC ACCTGACCCC ATGCCGAACT CAGAAGTGAA 240

ACGCCGTAGC GCCGATGGTA GTGTGGGGTC TCCCCATGCG AGAGTAGGGA ACTGCCAGGC 300

ATCAAATTAG AAGTATGGCA TTAACTTTCG GGGCAGTTCA CTACTCGACA GAGAGTAGGA 360

CAAAAGCGAA AGCTTTTGAA CGTTGCGAAG CAACGGCCCG AAGGGTGAAT CACGAAGTGA 420

TTCATAAACT GCCAGGCATC AAATTGCAGT AAACCGGGAT GAAAATCCGG GTTGTGACAA 480

AGAAATTCGG TGGAGCGG 498

>(45)C Isangi_noNCBImatch

ATGTTTTGGC GGAAGAGAGA CGATTTTCAG CACTGATTCA GATTACATCA GAACGTAAAA 60

ACGGTCTGAT AACAGAATTT GCCTGGCGGC AGTAGCGCGG TGGTCCCACC TGACCCCATG 120

CCGAACTCAG AAGTGAAACG CCGTAGCGCC GATGGTAGTG TGGGGTCTCC CCATGCGAGA 180

GTAGGGAACT GCCAGGCATC AAATTAAGCA GTAAACTGAT GTAAAAATCA GTGGTTGTAA 240

AAGAATTCGG TGGAGCGG 258

>(46)C Ohio_CP030024

ATGTTTTGGC GGATTGAGAG GACAACAATT TTCAGCTCAG ATTCAGAGTC CGAAGGATTT 60

TACGCTGAGA CAAGGCGGCA AACGCAGCGA CGGAAGGAGC ATACAGAAGT ATGTGACTGA 120

CGCCCGCAAG AGCAGCCAAC GCCGTATCAG TGTAAAAGAC ACAGGACAGA GCACAAAGAA 180

TTTGCCTGGC GGCACTAGCG CGGTGGTCCC ACCTGACCCC ATGCCGAACT CAGAAGTGAA 240

ACGCCGTAGC GCCGATGGTA GTGTGGGGTC TCCCCATGCG AGAGTAGGGA ACTGCCAGGC 300

ATCAAATTAA GCAGTAAACT GATGTAAAAA TCAGTGGTTG TAAAAGAATT CGGTGGAGCG 360

G 361

>(47)C Infantis_noNCBImatch

ATGTTTTGGC GGATTGAGAG ATGATTTTCA GCACTGATTC AGAGTTGAGT ACGCAATAAT 60

TTGCGCAGCA GCAAGGCGGC AAGCGAAGGA AAGGAAGGAG CATACAGAAG TATGTGACTG 120

ACTTTACGAG CGCAGGCAAC GCCGCTGATG CGATAAAGAA TTGCGTACTG AGCATAAAAG 180

AATTTGCCTG GCGGCAGTAG CGCGGTGGTC CCACCTGACC CCATGCCGAA CTCAGAAGTG 240

AAACGCCGTA GCGCCGATGG TAGTGTGGGG TCTCCCCATG CGAGAGTAGG GAACTGCCAG 300

GCATCAAATT AGAAGTATGG CATTAACTTT CGGGGCAGTT CACTACTCGA CAGAGAGTAG 360

GACAAAAGCG AAAGCTTTTG AACGTTGCGA AGCAACGGCC CGAAGGGGGA ATCACGAAGT 420

GATTCATAAG CTGCCAGGCA TCAAATTGCA GTAAACCGGG ATGAAAATCC GGATTGTGAC 480

AAAGAAATTC GGTGGAGCGG 500

>(48)C Cerro_noNCBImatch

ATGTTTTGGC GGATGAGAAG ACAACAATTT TCAGCACTGA TTCCGGATTT TCGGGAACGA 60

AAGATTTTAC GCTGAGGCAA GGCGGCAAAT GAAGGAAAGG AAGGAGCATA CAGAAGTATG 120

TGACTGACTT TACGAATGCA GCCAACGCAG CATCGGTGTA AAAGATTCGT TTCTGGCAAC 180

AGAATTTGCC TGGCGGCACT AGCGCGGTGG TCCCACCTGA CCCCATGCCG AACTCAGAAG 240

TGAAACGCCG TAGCGCCGAT GGTAGTGTGG GGTCTCCCCA TGCGAGAGTA GGGAACTGCC 300

AGGCATCAAA TTAGAAGTAT GGCATTAACT TTCGGGGCAG TTCACTACTC GACAGAGAGT 360

AGGACAAAAG CGAAAGCTTT TGAACGTTGC GAAGCAACGG CCCGAAGGGC GAATCACGAA 420

GTGATTCATA AACTGCCAGG CATCAAATTA AGCAGTAAAC TGATGTAAAA ATCAGTGGTT 480

GTAAAAGAAT TCGGTGGAGC GG 502

>(49)C Blockley_CP043662

ATGTTTTGGC GGATGAGAGA CGATTTTCAG CACTGATTCA GAGTTGAGTA CGCAATAATT 60

TGCGCAGCAG CAAGGCGGCA AGCGAAGGAA AGGAAGGAGC ATACAGAAGT ATGTGACTGA 120

GTTTACGAGC GCAGGCAACG CCGCTGATGC GATAAAGAAT TGCGTACAGA GCACAAAAGA 180

ATTTGCCTGG CGGCACTAGC GCGGTGGTCC CACCTGACCC CATGCCGAAC TCAGAAGTGA 240

AACGCCGTAG CGCCGATGGT AGTGTGGGGT CTCCCCATGC GAGAGTAGGG AACTGCCAGG 300

CATCAAATTA GAAGTATGGC ATTAACTTTC GGGGCAGTTC ACTACTCGAC AGAGAGTAGG 360

ACAAAAGCGA AAGCTTTTGA ACGTTGCGAA GCAACGGCCC GAAGGGTGAA TCACGAAGTG 420

ATTCATAAAC TGCCAGGCAT CAAATTAAGC AGTAAACTGA TGTAAAAATC AGTGATTGTA 480

AAAGAATTCG GTGGAGCGG 499

>(50)X Johannesburg_CP074325

CTTGCCCCGA GATGAGTTCT CCCTGAGACT TAGAGTCTCC TGAAGGAACG TTGAAGACGA 60

CGACGTTGAT AGGCCGGGTG TGTAAGCGCA GCGATGCGTT GAGCTAACCG GTACTAATGA 120

ACCGTGAGGC TTAACCTTAC AACGCCGAAG ATGTTTTGGC GGATGAGAGA CGATTTTCAG 180

CACTGATTCA GAGTTGAGTA CGCAATAATT TGCGCAGCAG CAAGGCGGCA AGCGAAGGAA 240

AGGAAGGAGC ATACAGAAGT ATGTGACTGA GTTTACGAGC GCAGGCAACG CCGCTGATGC 300

GATAAAGAAT TGCGTACTGA GCATAAAAGA ATTTGCCTGG CGGCACTAGC GCGGTGGTCC 360

CACCTGACCC CATGCCGAAC TCAGAAGTGA AACGCCGTAG CGCCGATGGT AGTGTGGGGT 420

CTCCCCATGC GAGAGTAGGG AACTGCCAGG CATCAAATTG CAGTAAACCG GGATGAAAAT 480

CCGGGTTGTG ACAAAGAAAT TCGGTGGAGC GGTAGTTCAG TTGGTTAGAA TACCTGCCTG 540

TCACGCAGGG GGTCGCGGGT TCGAGTCCCG TCCGTTCCGC CACTTATTAA GAGCCCTGAG 600

CATTAGTTCA GGGCTTTTTC TTTGCCTGTC ATTTAATTAT TGCTCAATTC GCAAAAATCC 660

TCTGCATTTC TCGCTCTTTT TCTACAAAGC GCTGAAGGTC ATAATCCCCT CAGTTAACGA 720

ACATAGTGAT TAAAAAGAGG AATTCTATGA CTATCCCTGC ATTCGGTTTA GGTACTTTTC 780

GCCTGAAAGA CGACGTGGTT ATCGCTTCTG TGAAAACGGC GCTTGAACTG GGCTACCGTG 840

CTGTCGACAC TGCGCAAATC TATGATAATG AAGTTGCTGT TGGACAGGCG ATTGCTGAGA 900

GCGGCGTACC GCGTAATGAG CTGTACATCA CCACTAAGAT CTGGATTGAA AACCTTAGCA 960

AAGACAAGCT GATCCCAAGC CTGAAAGAGA GCCTGAAAAA ACTGCGTACC GATTATGTCG 1020

ATCTGACGCT GATCCACTGG CCATCTCCGG GTGATGCCGT GTCTGTCGAA GAATTTATGC 1080

AGGCATTGCT TGAAGCTAAA AACAAGGGCT GACTCGCGAA TTGGCATTTC AACTTCACAT 1140

TCCGTTGATG GAAAAGCTAT TGCCGCGGGG GCGTAGACAT ATCGCACCAC CAGATGAGCT 1200

TC 1202

>(51)C Genovar_12350N_noNCBImatch

ATGTTTTGGC GGTTGAGAGA CGATTTTCAG CACTGATTCC GGATTTTCGG GAACGAAAGA 60

TAAAAGATTC GTTTCCGGCA ACAGAATTTG CCTGGCGGCA CTAGCGCGGT GGTCCCACCT 120

GACCCCATGC CGAACTCAGA AGTGAAACGC CGTAGCGCCG ATGGTAGTGT GGGGTCTCCC 180

CATGCGAGAG TAGGGAACTG CCAGGCATCA AATTAGAAGT ATGGCATTAA CTTTCGGGGC 240

AGTTCACTAC TCGACAGAGA GTAGGACAAA AGCGAAAGCT TTTGAACGTT GCGAAGCAAC 300

GGCCCGAAGG GTGAATCACG AAGTGATTCA TAAACTGCCA GGCATCAAAT TAAGCAGTAA 360

GCCGGTCATA AACTGGTGGT TGTAAAAAAA TTCGGTGGAG CGG 403

>(52)C Miami_noNCBImatch

ATGTTTTGGC GGATGAGAGA CGATTTTCAG CACTGATTCA GAGTTGAGTA CGCAATAATT 60

TGCGCAGCAG CAAGGCGGCA AGCGAAGGAA AGGAAGGAGC ATACAGAAGT ATGTGACTGA 120

CTTTGCGAGC GCAGGCAACG CCGCTGATGT GATAAAGAAT TGCGTACTGA GCACAAAGAA 180

TTTGCCTGGC GGCACTAGCG CGGTGGTCCC ACCTGACCCC ATGCCGAACT CAGAAGTGAA 240

ACGCCGTAGC GCCGATGGTA GTGTGGGGTC TCCCCATGCG AGAGTAGGGA ACTGCCAGGC 300

ATCAAATTAG AAGTATGGCA TTAACTTTCG GGGCAGTTCA CTACTCGACA GAGAGTAGGA 360

CAAAAGCGAA AGCTTTTGAA CGTTGCGAAG CAACGGCCCG AAGGGTGAAT CACGAAGTGA 420

TTCATAAACT GCCAGGCATC AAATTAAGCA GTAAGCCGGT CATAAACTGG TGGTTGTAAA 480

AAAATTCGGT GGAGCGG 497

>(53)C Saintpaul_noNCBImatch

ATGTTTTGGC GGTTAAGAGA AGATTTTCAG CACTGATTCA GAGTTGAGTA CGCAATAATT 60

TGCGCAGCAG CGAGGCGGCA AGCGAAGGAA AGGAAGGAGC ATACAGAAGT ATGTGACTGA 120

CTTTACGAGC GCAGGCAACG CCGCTGATGC GATAAAGAAT TGCGTACAGA GCACAAAGAA 180

TTTGCCTGGC GGCACTAGCG CGGTGGTCCC ACCTGACCCC ATGCCGAACT CAGAAGTGAA 240

ACGCCGTAGC GCCGATGGTA GTGTGGGGTC TCCCCATGCG AGAGTAGGGA ACTGCCAGGC 300

ATCAAATTAG AAGTATGGCA TTAACTTTCG GGGCAGTTCA CTACTCGACA GAGAGTAGGA 360

CAAAAGCGAA AGCTTTTGAA CGTTGCGAAG CAACGGCCCG AAGGGTGAAT CACGAAGTGA 420

TTCATAAACT GCCAGGCATC AGATTAAGCA GTAAACTGAT GTAAAAATCA GTGGTTGTAA 480

AAGAATTCGG TGGAGCGG 498

>(54)C Albany_CP036165

ATGTTTTGGC GGATTGAGAA GACAACAATT TTCAGCTCAG ATTCAGAGTC CGAAGGATTT 60

TACGCTGAGA CAAGGCGGCA AACGCAGCGA CGGAAGGAGC ATACAGAAGT ATGTGACTGA 120

CGCCCGCAAG AGCAGCCAAC GCCGTATCAG TGTAAAAGAC ACAGGACAGA GCACAAAGAA 180

TTTGCCTGGC GGCACTAGCG CGGTGGTCCC ACCTGACCCC ATGCCGAACT CAGAAGTGAA 240

ACGCCGTAGC GCCGATGGTA GTGTGGGGTC TCCCCATGCG AGAGTAGGGA ACTGCCAGGC 300

ATCAAATTAG AAGTATGGCA TTAACTTTCG GGGCAGTTCA CTACTCGACA GAGAGTAGGA 360

CAAAAGCGAA AGCTTTTGAA CGTTGCGAAG CAACGGCCCG AAGGGTGAAT CACGAAGTGA 420

TTCATAAACT GCCAGGCATC AAATTGCAGT AAACCGGGAT GAAAATCCGG ATTGTGACAA 480

AGAAATTCGG TGGAGCGG 498

>(55)C Braenderup_CP022490

CTGTTTTGGC GGATTGAGAA GACAACAATT TTCAGCTCAG ATTCAGAGTC CGAAGGATTT 60

TACGCTGAGA CAAGGCGGCA AACGCAGCGA CGGAAGGAGC ATACAGAAGT ATGTGACTGA 120

CGCTCGCAAG AGCAGCCAAC GCGGTATCAG TGTAAAAGAC ACAGGACAGA GCACAAAGAA 180

TTTGCCTGGC GGCACTAGCG CGGTGGTCCC ACCTGACCCC ATGCCGAACT CAGAAGTGAA 240

ACGCCGTAGC GCCGATGGTA GTGTGGGGTC TCCCCATGCG AGAGTAGGGA ACTGCCAGGC 300

ATCAAATTAG AAGTATGGCA TTAACTTTCG GGGCAGTTCA CTACTCGACA GAGAGTAGGA 360

CAAAAGCGAA AGCTTTTGAA CGTTGCGAAG CAACGGCCCG AAGGATGAAT CACGAAGTGA 420

TTCATAAACT GCCAGGCATC AAATTAAGCA GTAAACTGAT GTAAAAATCA GTGGTTGTAA 480

AAGAATTCGG TGGAGCGG 498

>(56)C Rissen_noNCBImatch

ATGTTTTGGC GGTTGAGAGA TGATTTTCAG CACTGATTCA GAGTTGAGTA CGCAATAATT 60

TGCGCAGCGG CAAGGCGGCA AGCGAAGGAA AGGAAGGAGC ATACAGAAGT ATGTGACTGA 120

CTTTACGAGC GCAGGTAACG CCGCTGATGC GATAAAGAAT TGCGCACTGA GCACAAAGAA 180

TTTGCCTGGC GGCCGTAGCG CGGTGGTCCC ACCTGACCCC ATGCCGAACT CAGAAGTGAA 240

ACGCCGTAGC GCCGATGGTA GTGTGGGGTC TCCCCATGCG AGAGTAGGGA ACTGCCAGGC 300

ATCAAATTAG AAGTATGGCA TTAACTTTCG GGGCAGTTCA CTACTCGACA GAGAGTAGGA 360

CAAAAGCGAA AGCTTTTGAA CGTTGCGAAG CAACGGCCCG AAGGGTGAAT CACGAAGTGA 420

TTCATAAACT GCCAGGCATC AAATTAAGCA GTAAACTGAT GTAAAAATCA GTGGTTGTAA 480

AAGAATTCGG TGGAGCGG 498

>(57)C Kedougou_noNCBImatch

ATGTTTTGGC GGATGAGAGA CGATTTTCAG CACTGATTCA GAGTTGAGTA CGCAATAATT 60

TGCGCAGCAG CAAGGCGGCA AGCGAAGGAA AGGAAGGAGC ATACAGAAGT ATGTGACTGA 120

CTTTACGAGC GCGGGCAACG CCGCTGATGC GATAAAGAAT TGCGTACTGA GCACAAAAGA 180

ATTTGCCTGG CGGCCGTAGC GCGGTGGTCC CACCTGACCC CATGCCGAAC TCAGAAGTGA 240

AACGCCGTAG CGCCGATGGT AGTGTGGGGT CTCCCCATGC GAGAGTAGGG AACTGCCAGG 300

CATCAAATTA GAAGTATGGC ATTAACTTTC GGGGCAGTTC ACTACTCGAC AGAGAGTAGG 360

ACAAAAGCGA AAGCTTTTGA ACGTTGCGAA GCAACGGCCC GAAGGGTGAA TCACGAAGTG 420

ATTCATAAAC TGCCAGGCAT CAAATTAAGC AGTAAACTGA TGTAAAAATC AGTGGTTGTA 480

AAAGAATTCG GTGGAGCGG 499

>(58)C Enteritidis_CP007598

ATGTTTTGGC GGATGAGAAG ACGATTTTCA GCAGTGATTC AGAGTTGAGT ACGCAATAAT 60

TTGCGCAGCA GCAAGGCGGC AAGCGAAGGA AAGGAAGGAG CATACAGAAG TATGTGACTG 120

AGTTTACGAG CGCAGGCAAC GCCGCTGATG CGATAAAGAA TTGCGTACAG AGCACAAAGA 180

ATTTGCCTGG CGGCAGTAGC GCGGTGGTCC CACCTGACCC CATGCCGAAC TCAGAAGTGA 240

AACGCCGTAG CGCCGATGGT AGTGTGGGGT CTCCCCATGC GAGAGTAGGG AACTGCCAGG 300

CATCAAATTA GAAGTATGGC ATTAACTTTC GGGGCAGTTC ACTACTCGAC AGAGAGTAGG 360

ACAAAAGCGA AAGCTTTTGA ACGTTGCGAA GCAACGGCCC GAAGGGTGAA TCACGAAGTG 420

ATTCATAAAC TGCCAGGCAT CAAATTGCAG TAAACCGGGA TGAAAATCCG GGTTGTGACA 480

AAGAAATTCG GTGGAGCGG 499

>(59)C Paratyphi-B_CP020492

ATGTTTTGGC GGATTGAGAA GACGAACAAT TTTCAGCTCA GATTCAGAGT CCGAAGGATT 60

TTACGCTGAG ACAAGGCGGC AAACGCAGCG ACGGAAGGAG CATACAGAAG TATGTGACTG 120

ACGCTCGCAA GAGCAGCCAA CGCCGTATCA GTGTAAAAGA CACAGGACAG AGCACAAAGA 180

ATTTGCCTGG CGGCACTAGC GCGGTGGTCC CACCTGACCC CATGCCGAAC TCAGAAGTGA 240

AACGCCGTAG CGCCGATGGT AGTGTGGGGT CTCCCCATGC GAGAGTAGGG AACTGCCAGG 300

CATCAAATAG AATGAAAGGC CCTGTCGGAA GACTGGGCCT TTTGTTTTAT CTGTAGTCTG 360

CCGGTGAGCA CTCTCCTGAG GAGGAGAAAT CCGCCGGGAG CGGATTTGAA CGTTGCGCAG 420

CAACGGCCCG GAGGGTGGCG GGCAGGACGC CCGCCATAAA CTGCCAGGCA TCAAATTAAG 480

CAGTAAGCCG GTCATAGATC GGTGGTTGTA AAAGAATTCG GTGGAGCGG 529

>(60)X Muenster_Typhimurium_CP019198_CP074302

TGCGGAAGAG ATAAGTGCTG AAAGCATCTA AGCACGAAAC TTGCCCCGAG ATGAGTTCTC 60

CCTGAGACTT AGAGTCTCCT GAAGGAACGT TGAAGACGAC GACGTTGATA GGCCGGGTGT 120

GTAAGCGCAG CGATGCGTTG AGCTAACCGG TACTAATGAA CCGTGAGGCT TAACCTTACA 180

ACGCCGAAGA TGTTTTGGCG GTTGAGAAGA CAACAATTTT CAGCTCAGAT TCAGATTACA 240

TCAGAACGTA AAAACGGTCT GATAACAGAA TTTGCCTGGC GGCACTAGCG CGGTGGTCCC 300

ACCTGACCCC ATGCCGAACT CAGAAGTGAA ACGCCGTAGC GCCGATGGTA GTGTGGGGTC 360

TCCCCATGCG AGAGTAGGGA ACTGCCAGGC ATCAATTTAA GCAGTAAACT GATGTAAAAA 420

TCAGTGGTTG TAAAAGAATT CGGTGGAGCG GTAGTTCAGT TGGTTAGAAT ACCTGCCTGT 480

CACGCAGGGG GTCGCGGGTT CGAGTCCCGT CCGTTCCGCC ACTTATTAAG AGCCCTGAGC 540

ATTAGCTCAG GGCTTTTTCT TTGCCTGTCA TTTAATTATT GCTCAATTCG CAAAAATCCT 600

CTGCATTTCT CGCTCTTTTT CTACAAAGCG CTGAAGGTCA TAATCCCCTC AGTTAACGAA 660

CATAGTGATT AAAAAGAGGA GTTCTATGAC TATCCCTGCA TTCGGTTTAG GTACTTTTCG 720

CCTGAAAGAC GACGTGGTTA TCGCTTCTGT GAAAACGGCG CTTGAACTGG GCTACCGTGC 780

TGTCGACACT GCGCAAATCT ATGATAATGA AGTTGCTGTT GGACAGGCGA TTGCTGAGAG 840

CGGCGTACCG CGTAATGAGC TGTACATCAC CACTAAGATC TGGATTGAAA ACCTTAGCAA 900

AGACAAGCTG ATCCCAAGCC TGAAAGAGAG CCTGAAAAAA CTGCGTACCG ATTATGTCGA 960

TCTGACGCTG ATCCACTGGC CATCTCCGGG TGATGCCGTG TCTGTCGAAG AATTTATGCA 1020

GGCATTGCTT GAAGCTAAAA AACAAGGGCT GACTCGCGAA ATTGGCATTT CAAACTTCAC 1080

CATTCCGTTG ATGGAAAAGG CTATTGCCGC GGTGGGCGTA GACAATATCG CCACCAACCA 1140

GATTGAGCTA TCTCCGTATC TGCAGAATCG 1170

>(61)X Muenchen_CP022658

GGAAGAGATA AGTGCTGAAA GCATCTAAGC ACGAAACTTG CCCCGAGATG AGTTCTCCCT 60

GAGACTTTAG GTCTCCTGAA GGAACGTTGA AGACGACGAC GTTGATAGGC CGGGTGTGTA 120

AGCGCAGCGA TGCGTTGAGC TAACCGGTAC TAATGAACCG TGAGGCTTAA CCTTACAACG 180

CCGAAGATGT TTTGGCGGTT GAGAGACGAT TTTCAGCACT GATTCAGATT ACATCAGAAC 240

GTAAAAACGG TCTGATAACA GAATTTGCCT GGCGGCACTA GCGCGGTGGT CCCACCTGAC 300

CCCATGCCGA ACTCAGAAGT GAAACGCCGT AGCGCCGATG GTAGTGTGGG GTCTCCCCAT 360

GCGAGAGTAG GGAACTGCCA GGCATCAAAT TAGAAGTATG GCATTAACTT TCGGGGCAGT 420

TCACTACTCG ACAGAGAGTA GGACAAAAGC GAAAGCTTTT GAACGTTGCG AAGCAACGGC 480

CCGAAGGGTG AATCACGAAG TGATTCATAA ACTGCCAGGC ATCAAATTGC AGTAAACCGG 540

GATGAAAATC CGGGTTGTGA CAAAGAAATT CGGTGGAGCG GTAGTTCAGT TGGTTAGAAT 600

ACCTGCCTGT CACGCAGGGG GGCGCGGGTT CGAGTCCCGT CCGTTCCGCC ACTTATTAAG 660

AGCCCTGAGC ATTAGCTCAG GGCTTTTTCT TTGCCTGCGA TTTAATTATT GCTCAATTCG 720

CAAAAATCCT CTGCATTTCT CGCTCTTTTT CTACAAAGCG CTGAAGGTCA TAATCCCCTC 780

AGTTAACGAA CATAGTGATT AAAAAGAGGA ATTCTATGAC TATCCCTGCA TTCGGTTTAG 840

GTACTTTTCG CCTGAAAGAC GACGTGGTTA TCGCTTCTGT TAAAACGGCG CTTGAACTGG 900

GCTACCGTGC TGTCGACACT GCGCAAATCT ATGATAATGA AGCGGCTGTT GGACAGGCGA 960

TTGCTGAGAG CGGTGTACCG CGTAATGAGC TGTATATCAC CACCAAGATC TGGATTGAAA 1020

ACCTTAGCAA AGACAAGCTA ATCCCAAGCC TGAAAGAGAG CCTGAAAAAA CTGCGTACCG 1080

ATTATGTCGA TCTGACGCTG ATCCACTGGC CATCTCCGGG TGATGCCGTG TCTGTCGAAG 1140

AATTATGCAG GCTTGCTTGA AGCTAAAAAC AAGGGCTGAC TCGCGAAATG GCATTTCAAC 1200

TTCACATTCC TTGATGGAAA AGCTTTGCC 1229

>(62)C Enteritidis_CP045956

ATGTTTTGGC GGATGAGAAG ACGATTTTCA GCACTGATTC AGATTACATC AGAACGTAAA 60

AACGGTCTGA TAACAGAATT TGCCTGGCGG CACTAGCGCG GTGGTCCCAC CTGACCCCAT 120

GCCGAACTCA GAAGTGAAAC GCCGTAGCGC CGATGGTAGT GTGGGGTCTC CCCATGCGAG 180

AGTAGGGAAC TGCCAGGCAT CAAATTAGAA GTATGGCATT AACTTTCGGG GCAGTTCACT 240

ACTCGACAGA GAGTAGGACA AAAGCGAAAG CTTTTGAACG TTGCGAAGCA ACGGCCCGAA 300

GGGTGAATCA CGAAGTGATT CATAAACTGC CAGGCATCAA ATTAAGCAGT AAACTGATGT 360

AAAAATCAGT GGTTGTAAAA GAATTCGGTG GAGCGG 396

>(63)C Alachua_noNCBImatch

ATGTTTTGGC GGATTGAGAA GACAACAATT TTCAGCTCAG ATTCAGAGTC CGAAGGATTT 60

TACGCTGAGA CAAGGCGGCA AACGCAGCGA CGGAAGGAGC ATACAGAAGT ATGTGACTGA 120

CGCTCGCAAG AGCAGCCAAC GCCGTATCAG TGTAAAAGAC AAAGGACAGA GCACAAAGAA 180

TTTGCCTGGC GGCACTAGCG CGGTGGTCCC ACCTGACCCC ATGCCGAACT CAGAAGTGAA 240

ACGCCGTAGC GCCGATGGTA GTGTGGGGTC TCCCCATGCG AGAGTAGGGA ACTGCCAGGC 300

ATCAAATTAA GCAGTAAGCC GGTCATAGAC TGGTGGTTGT AAAAGAATTC GGTGGAGCGG 360

>(64)C Litchfield_CP082600

ATGTTTTGGC GGATTGAGAA GACAACAATT TTCAGCTCAG ATTCAGAGTC CGAAGGATTT 60

TACGCTGAGA CAAGGCGGCA AACGCAGCGA CGGAAGGAGC ATACAGAAGT ATGTGACTGA 120

CGCCCGCAAG AGCAGCCAAC GCCGTATCAG TGTAAAAGAC ACAGGACAGA GCACAAAGAA 180

TTTGCCTGGC GGCACTAGCG CGGTGGTCCC ACCTGACCCC ATGCCGAACT CAGAAGTGAA 240

ACGCCGTAGC GCCGATGGTA GTGTGGGGTC TCCCCATGCG AGAGTAGGGA ACTGCCAGGC 300

ATCAAATTAG AAGTATGGCA TTAACTTTCG GGGCAGTTCA CTACTCGACA GAGAGTAGGA 360

CAAAAGCGAA AGCTTTTGAA CGTTGCGAAG CAACGGCCCG AAGGGTGAAT CACGAAGTGA 420

TTCATAAACT GCCAGGCATC AAATTAAGCA GTAAACTGAT GTAAAAATCA GTGGTTGTAA 480

AAGAATTCGG TGGAGCGG 498

>(65)C Kiambu_noNCBImatch

ATGTTTTGGC GGATGAGAAG ACGATTTTCA GCACTGATTC CGGATTTTCG GGAACGAAAG 60

ATTTTACGCT GAGGCAAGGC GGCAAATGAA GGAAAGGAAG GAGCATACAG AAGTATGTGA 120

CTGACTTTAC GAATGCAGCC AACGCAGCAT CGGTGTAAAA GATTCGTTTC TGGCAACAGA 180

ATTTGCCTGG CGGCACTAGC GCGGTGGTCC CACCTGACCC CATGCCGAAC TCAGAAGTGA 240

AACGCCGTAG CGCCGATGGT AGTGTGGGGT CTCCCCATGC GAGAGTAGGG AACTGCTAGG 300

CATCAAATTA GAAGTATGGC ATTAACTTTC GGGGCAGTTC ACTACTCGAC AGAGAGTAGG 360

ACAAAAGCGA AAGCTTTTGA ACGTTGCGAA GCAACGGCCC GAAGGGTGAA TCACGAAGTG 420

ATTCATAAAC TGCCAGGCAT CAAATTAAGC AGTAAACTGA TGTAAAAATC AGTGGTTGTA 480

AAAGAATTCG GTGGAGCGG 499

>(66)X Anatum_Hayindogo_CP007584_CP017719

GAAGAGATAA GTGCTGAAAG CATCTAAGCA CGAAACTTGC CCCGAGATGA GTTCTCCCTG 60

AGACTTAGAG TCTCCTGAAG GAACGTTGAA GACGACGACG TTGATAGGCC GGGTGTGTAA 120

GCGCAGCGAT GCGTTGAGCT AACCGGTACT AATGAACCGT GAGGCTTAAC CTTACAACGC 180

CGAAGATGTT TTGGCGGATT GAGAAGACAA CAATTTTCAG CTCAGATTCA GAGTCCGAAG 240

GATTTTACGC TGAGACAAGG CGGCAAACGC AGCGACGGAA GGAGCATACA GAAGTATGTG 300

ACTGACGCTC GCAAGAGCAG CCAACGCCGT ATCAGTGTAA AAGACACAGG ACAGAGCACA 360

AAGAATTTGC CTGGCGGCAC TAGCGCGGTG GTCCCACCTG ACCCCATGCC GAACTCAGAA 420

GTGAAACGCC GTAGCGCCGA TGGTAGTGTG GGGTCTCCCC ATGCGAGAGT AGGGAACTGC 480

CAGGCATCAA ATTAGAAGTA TGGCATTAAC TTTCGGGGCA GTTCACTACT CGACAGAGAG 540

TAGGACAAAA GCGAAAGCTT TTGAACGTTG CGAAGCAACG GCCCGAAGGG TGAATCACGA 600

AGTGATTCAT AAACTGCCAG GCATCAAATT AAGCAGTAAA CTGATGTAAA AATCAGTGGT 660

TGTAAAAGAA TTCGGTGGAG CGGTAGTTCA GTTGGTTAGA ATACCTGCCT GTCACGCAGG 720

GGGTCGCGGG TTCGAGTCCC GTCCGTTCCG CCACTTATTA AGAGCCCTGA GCATTAGCTC 780

AGGGCTTTTT CTTTGCATGT CATTTAATTA TTGCTCAATT CGCAAAAATC CTCTGCATTT 840

CTCGCTCTTT TTCTGCAAAG CGCTGAAGGT CATAATCCCC TCAGTTAACG AACATAGTGA 900

TTAAAAAGAG GAATTCTATG ACTATCCCTG CATTCGGTTT AGGTACTTTT CGCCTGAAAG 960

ACGACGTGGT TATCGCTTCT GTGAAAACGG CGCTTGAACT GGGCTACCGT GCTGTCGACA 1020

CTGCGCAAAT CTATGATAAT GAAGCGGCTG TTGGACAGGC GATTACTGAG AGCGGCGTAC 1080

CACGTAGCGA GCTGTACATC ACCACCAAGA TCTGGATTGA A 1121

>(67)C Meleagridis_noNCBImatch

CCGCTCCACC GAATTTCTTT GTCACAACCC GGATTTTCAT CCCGGTTTAC TGCAATTTGA 60

TGCCTGGCAG TTTATGAATC ACTTCGTGAT TCACCCTTCG GGCCGTTGCT TCGCAACGTT 120

CAAAAGCTTT CGCTTTTGTC CTACTCTCTG TCGAGTAGTG AACTGCCCCG AAAGTTAATG 180

CCATACTTCT AATTTGATGC CTGGCAGTTC CCTACTCTCG CATGGGGAGA CCCCACACTA 240

CCATCGGCGC TACGGCGTTT CACTTCTGAG TTCGGCATGG GGTCAGGTGG GACCACCGCG 300

CTAGTGCCGC CAGGCAAATT CTTTGTGCTC TGTACGCAAT TCTTTATCGC ATCAGCGGCG 360

TTGCCTGCGC TCGTAAAGTC AGTCACATAC TTCTGTATGC TCCTTCCTTT CCTTCGCTTG 420

CCGCCTTGCT GCTGCGCAAA TTATTGCGTA CTCAACTCTG AATCACTGCT GAAAATCGTC 480

TCTCATCCGC CAAAACAT 498

>(68)C Molade_noNCBImatch

ATGTTTTGGC GGATGAGAAG ACGATTTTCA GCTCAGATTC AGATTACATC AGAACGTAAA 60

AACGGTCTGA TAACAGAATT TGCCTGGCGG CCGTAGCGCG GTGGTCCCAC CTGACCCCAT 120

GCCGAACTCA GAAGTGAAAC GCCGTAGCGC CGATGGTAGT GTGGGGTCTC CCCATGCGAG 180

AGTAGGGAAC TGCCAGGCAT CAAATTGCAG TAAACCAGGA TGAAAATCCG GGTTGTGACA 240

AAGAAATTCG GTGGAGCGG 259

>(69)C Bareilly_noNCBImatch

ATGTTTTGGC GGATGAGAGA CGATTTTCAG CACTGATTCA GAGTTGAGTA CGCAATAATT 60

TGCGCAGCAG CAAGGCGGCA AGCGAAGGAA AGGCAGGAGC ATACAGAAGT ATGTGACTGA 120

GTTTACGAGC GCAGGCAACG CCGCTGATGC GATAAAGAAT TGCGTACAGA GCACAAAAGA 180

ATTTGCCTGG CGGCACTAGC GCGGTGGTCC CACCTGACCC CATGCCGAAC TCAGAAGTGA 240

AACGCCGTAG CGCCGATGGT AGTGTGGGGT CTCCCCATGC GAGAGTAGGG AACTGCCAGG 300

CATCAAATTA GAAGTATGGC ATTAACTTTC GGGGCAGTTC ACTACTCGAC AGAGAGTAGG 360

ACAAAAGCGA AAGCTTTTGA ACGTTGCGAA GCAACGGCCC GAAGGGTGAA TCACGAAGTG 420

ATTCATAAAC TGCCAGGCAT CAATTTAAGC AGTAAACTGA TGTAAAAATC AGTGATTGTA 480

AAAGAATTCG GTGGAGCGG 499

>(70)C Newport_CP075033

ATGTTTTGGC GGATTGAGAA GACAACAATT TTCAGCTCAG ATTCAGAGTC CGAAGGATTT 60

TACGCTGAGA CAAGGCGGCA AACGCAGCGA CGGAAGGAGC ATACAGAAGT ATGTGACTGA 120

CGCCCGCAAG AGCAGCCAAC GCCGTATCAG TGTAAAAGAC ACAGGACAGA GCACAAAGAA 180

TTTGCCTGGC GGCACTAGCG CGGTGGTCCC ACCTGACCCC ATGCCGAACT CAGAAGTGAA 240

ACGCCGTAGC GCCGATGGTA GTGTGGGGTC TCCCCATGCG AGAGTAGGGA ACTGCCAGGC 300

ATCAAATTAG AAGTATGGCA TTAACTTTCG GGGCAGTTCA CTACTCGACA GAGAGTAGGA 360

CAAAAGCGAA AGCTTTTGAA CGTTGCGAAG CAACGGCCCG AAGGGTGAAT CACGAAGTGA 420

TTCATAAACT GCCAGGCATC AAATTGCAGT AAACCGGGAT GAAAATCCGG GTTGTGACAA 480

AGAAATTCGG TGGAGCGG 498

>(71)C Soerenga_CP074317

ATGTTTTGGC GGATGAGAGA CGATTTTCAG CACTGATTCC GGATTTTCGG GAACGAAAGA 60

TTTTACGCTG AGGCAAGGCG GCAAATGAAG GAAAGGAAGG AGCATACAGA AGTATGTGAC 120

TGACTTTGCG AATGCAGCCA ACGCAGCATC GGTGTAAAAG ATTCGTTTCC GGCAACAGAA 180

TTTGCCTGGC GGCACTAGCG CGGTGGTCCC ACCTGACCCC ATGCCGAACT CAGAAGTGAA 240

ACGCCGTAGC GCCGATGGTA GTGTGGGGTC TCCCCATGCG AGAGTAGGGA ACTGCCAGGC 300

ATCAAATTAG AAGTATGGCA TTAACTTTCG GGGCAGTTCA CTACTCGACA GAGAGTAGGA 360

CAAAAGCGAA AGCTTTTGAA CGTTGCGAAG CAACGGCCCG AAGGGTGAAT CACGAAGTGA 420

TTCATAAACT GCCAGGCATC AAATTAAGCA GTAAACTGAT GTAAAAATCA GTGGTTGTAA 480

AAGAATTCGG TGGAGCGG 498

>(72)C Norwich_noNCBImatch

ATGTTTTGGC GGTTGAGAGA TGATTTTCAG CACTGATTCC GGATTTTCGG GAACGAAAGA 60

TAAAAGATTC GTTTCCGGCA ACAGAATTTG CCTGGCGGCA CTAGCGCGGT GGTCCCACCT 120

GACCCCATGC CGAACTCAGA AGTGAAACGC CGTAGCGCCG ATGGTAGTGT GGGGTCTCCC 180

CATGCGAGAG TAGGGAACTG CCAGGCATCA AATTAAGCAG TAAACTGATG TAAAAATCAG 240

TGGTTGTAAA AGAATTCGGT GGAGCGG 267

>(73)C Gera_Genovar_3109_noNCBImatch

ATGTTTTGGC GGATGAGAGA TAATTTTCAG CACTGATTCA GATTACATCA GAACGTAAAA 60

ACGGTCTGAT AACAGAATTT GCCTGGCGGC ACTAGCGCGG TGGTCCCACC TGACCCCATG 120

CCGAACTCAG AAGTGAAACG CCGTAGCGCC GATGGTAGTG TGGGGTCTCC CCATGCGAGA 180

GTAGGGAACT GCCAGGCATC AAATTAGAAG TATGGCATTA ACTTTCGGGG CAGTTCACTA 240

CTCGACAGAG AGTAGGACAA AAGCGAAAGC TTTTGAACGT TGCGAAGCAA CGGCCCGAAG 300

GGTGAATCAC GAAGTGATTC ATAAACTGCC AGGCATCAAA TTAAGCAGTA AACTGATGTA 360

AAAATCAGTG GTTGTAAAAG AATTCGGTGG AGCGG 395

>(74)C Hartford_CP074274

ATGTTTTGGC GGTTGAGAAG ACGAACAATT TTCAGCTCAG ATTCAGAGTC CGAAGGATTT 60

TACGCTGAGA CAAGGCGGCA AACGCAGCGA CGGAAGGAGC ATACAGAAGT ATGTGACTGA 120

CGCTCGCAAG AGCAGCCAAC GCCGTATCAG TGTAAAAGAC ACAGGACAGA GCACAAAGAA 180

TTTGCCTGGC GGCACTAGCG CGGTGGTCCC ACCTGACCCC ATGCCGAACT CAGAAGTGAA 240

ACGCCGTAGC GCCGATGGTA GTGTGGGGTC TCCCCATGCG AGAGTAGGGA ACTGCCAGGC 300

ATCAAATTAA GCAGTAAACT GATGTAAAAA TCAGTGGTTG TAAAAGAATT CGGTGGAGCG 360

G 361

>(75)C Cerro_NoNCBImatch

ATGTTTTGGC GGATGAGAGA AGATTTTCAG CACTGATTCA GAGTTGAGTA CGCAATAATT 60

TGCGCAGCAG CAAGGCGGCA AGCGAAGGAA AGGAAGGAGC ATACAGAAGT ATGTGACTGA 120

CTTTACGAGC GCAGGCAACG CCGCTGATGC GATAAAGAAT TGCGTACTGA GCACAAAAGA 180

ATTTGCCTGG CGGCAGTAGC GCGGTGGTCC CACCTGACCC CATGCCGAAC TCAGAAGTGA 240

AACGCCGTAG CGCCGATGGT AGTGTGGGGT CTCCCCATGC GAGAGTAGGG AACTGCCAGG 300

CATCAAATTA AGCAGTAAGC CGGTCATAGA CTGGTGGTTG TAAAAGAATT CGGTGGAGCG 360

G 361

>(76)C Cerro_NoNCBImatch

ATGTTTTGGC GGATGAGAAG ACGATTTTCA GCACTGATTC CGGATTTTCG GGAACGAAAG 60

ATTTTACGCT GAGGCAAGGC TGCAAATGAA GGAAAGGAAG GAGCATACAG AAGTATGTGA 120

CTGACTTTAC GAATGCAGCC AACGCAGCAT CGGTGTAAAA GATTCGTTTC TGGCAACAGA 180

ATTTGCCTGG CGGCACTAGC GCGGTGGTCC CACCTGACCC CATGCCGAAC TCAGAAGTGA 240

AACGCCGTAG CGCCGATGGT AGTGTGGGGT CTCCCCATGC GAGAGTAGGG AACTGCCAGG 300

CATCAAATTA GAAGTATGGC ATTAACTTTC GGGGCAGTTC ACTACTCGAC AGAGAGTAGG 360

ACAAAAGCGA AAGCTTTTGA ACGTTGCGAA GCAACGGCCC GAAGGGTGAA TCACGAAGTG 420

ATTCATAAAC TGCCAGGCAT CAAATTAAGC AGTAAACTGA TGTAAAAATC AGTGGTTGTA 480

AAAGAATTCG GTGGAGCGG 499

>(77)C Amsterdam_NoNCBImatch

CTGTTTTGGC GGTGAGAGAC GATTTTCAGC ACTGATTCAG AGTTGAGTAC GCAATAATTT 60

GCGCAGCAGC AAGGCGGCAA GCGAAGGAAA GGAAGGAGCA TACAGAAGTA TGTGACTGAG 120

TTTACGAGCG CAGGCAACGC CGCTGATGCG ATAAAGAATT GCGTACAGAG CACAAAGAAT 180

TTGCCTGGCG GCCGTAGCGC GGTGGTCCCA CCTGACCCCA TGCCGAACTC AGAAGTGAAA 240

CGCCGTAGCG CCGATGGTAG TGTGGGGTCT CCCCATGCGA GAGTAGGGAA CTGCCAGGCA 300

TCAAATTAGA AGTATGGCAT TAACTTTCGG GGCAGTTCAC TACTCGACAG AGAGTAGGAC 360

AAAAGCGAAA GCTTTTGAAC GTTGCGAAGC AACGGCCCGA AGGGTGAATC ACGAAGTGAT 420

TCATAAACTG CCAGGCATTA AATTGCAGTA AACCGGGATG AAAATCCGGG TTGTGACAAA 480

GAAATTCGGT GGAGCGG 497

>(78)C Taksony_LR134146

ATGTTTTGGC GGATGAGAGA CGATTTTCAG CACTGATTCA GAGTTGAGTA CGCAATAATT 60

TGCGCAGCAG CAAGGCGGCA AGCGAAGAAA AGGAAGGAGC ATACAGAAGT ATGTGACTGA 120

CTTTACGAGC GCAGGCAACG CCGCTGATGC GATAAAGAAT TGCGTACTGA GCACAAAAGA 180

ATTTGCCTGG CGGCACTAGC GCGGTGGTCC CACCTGACCC CATGCCGAAC TCAGAAGTGA 240

AACGCCGTAG CGCCGATGGT AGTGTGGGGT CTCCCCATGC GAGAGTAGGG AACTGCCAGG 300

CATCAAATTA GAAGTATGGC ATTAACTTTC GGGGCAGTTC ACTACTCGAC AGAGAGTAGG 360

ACAAAAGCGA AAGCTTTTGA ACGTTGCGAA GCAACGGCCC GAAGGGTGAA TCACGAAGTG 420

ATTCATAAAC TGCCAGGCAT CAAATTGCAG TAAACCGGGA TGAAAATCCG GGTTGTGACA 480

AAGAAATTCG GTGGAGCGG 499

>(79)C Oranienburg_NoNCBImatch

ATGTTTTGGC GGTTGAGAAG ACAACAATTT TCAGCTCAGA TTCAGAGTCC GAAGGATTTT 60

ACGCTGAGAC AAGGCGGCAA ACGCAGCGAC GGAAGGAGCA TACAGAAGTA TGTGACTGAC 120

GCTCGGAAGA GCAGCCAACG CCGTATCAGT GTAAAAGACA CAGGACAGAG CACAAAGAAT 180

TTGCCTGGCG GCACTAGCGC GGTGGTCCCA CCTGACCCCA TGCCGAACTC AGAAGTGAAA 240

CGCCGTAGCG CCGATGGTAG TGTGGGGTCT CCCCATGCGA GAGTAGGGAA CTGCCAGGCA 300

TCAAATTAGA AGTATGGCAT TAACTTTCGG GGCAGTTCAC TACTCGACAG AGAGTAGGAC 360

AAAAGCGAAA GCTTTTGAAC GTTGCGAAGC AACGGCCCGA AGGGTGAATC ACGAAGTGAT 420

TCATAAACTG CCAGGCATCA AATTGCAGTA AACCGGGATG AAAATCCGGG TTGTGACAAA 480

GAAATTCGGT GGAGCGG 497

>(80)C IG_6,8_Genovar_1678_NoNCBImatch

ATGTTTTGGC GGATGAGAGA CGATTTTCAG CACTGATTCA GAGTTGAGTA CGCAATAATT 60

TGCGCAGCAG CAAGGCGGCA AGCGAAGGAA AGGAAGGAGC ATACAGAAGT ATGTGACTGA 120

CTTTACGAGC GCAGGCAACG CCGCTGATGC GATAAAGAAT TGCGTACAGA GCACAAAGAA 180

TTTGCCTGGC GGCACTAGCG CGGTGGTCCC ACCTGACCCC ATGCCGAACT CAGAAGTGAA 240

ACGCCGTAGC GCCGATGGTA GTGTGGGGTC TCCCCATGCG AGAGTAGGGA ACTGCCAGGC 300

ATCAAATTAG AAGTATGGCA TTAATTTTCG GGGCAGTTCA CTACTCGACA GAGAGTAGGA 360

CAAAAGCGAA AGCTTTTGAA CGTTGCGAAG CAACGGCCCG AAGGGTGAAT CACGAAGTGA 420

TTCATAAACT GCCAGGCATC AAATTGCAGT AAACCGGGAT GAAAATCCGG GTTGTGACAA 480

AGAAATTCGG TGGAGCGG 498

>(81)C Uganda_CP051398

ATGTTTTGGC GGATGAGAAG ACGAACAATT TTCAGCACTG ATTCCGGATT TTCGGGAACG 60

AAAGATTTTA CGCTGAGGCA AGGCGGCAAA TGAAGGAAAG GAAGGAGCAT ACAGAAGTAT 120

GTGACTGACT TTACGAATGC AGCCAACGCA GCATCGGTGT AAAAGATTCG TTTCCGGCAA 180

CAGAATTTGC CTGGCGGCAC TAGCGCGGTG GTCCCACCTG ACCCCATGCC GAACTCAGAA 240

GTGAAACGCC GTAGCGCCGA TGGTAGTGTG GGGTCTCCCC ATGCGAGAGT AGGGAACTGC 300

CAGGCATCAA ATTAAGCAGT AAACTGATGT AAAAATCAGT GGTTGTAAAA GAATTCGGTG 360

GAGCGG 366

>(82)C Mississippi_NoNCBImatch

ATGTTTTGGC GAAAGAGAAG ACAACAATTT TCAGCACTGA TTCAGAGTCC GAAGGATTTT 60

ACGCTGAGAC AAGGCGGCAA ACGCAGCGAC GGAAGGAGCA TACAGAAGTA TGTGACTGAC 120

GCCCGCAAGA GCAGCCAACG CCGTATCAGT GTAAAAGACA CAGGACAGAG CACAAAGAAT 180

TTGCCTGGCG GCACTAGCGC GGTGGTCCCA CCTGACCCCA TGCCGAACTC AGAAGTGAAA 240

CGCCGTAGCG CCGATGGTAG TGTGGGGTCT CCCCATGCGA GAGTAGGGAA CTGCCAGGCA 300

TCAAATTGCA GTAAACCGGG ATGAAAATCC GGGTGGTGAC AAAGAAATTC GGTGGAGCGG 360

>(83)C Litchfield_NoNCBImatch

ATGTTTTGGC GGATTGAGAA GACAACAATT TTCAGCTCAG ATTCAGAGTC CGAAGGATTT 60

TACGCTGAGA CAAGGCGGCA AACGCAGCGA CGGAAGGAGC ATACAGAAGT ATGTGACTGA 120

CGCTCGCAAG AGCAGCCAAC GCGGTATCAG TGTAAAAGAC ACAGGACAGA GCACAAAGAA 180

TTTGCCTGGC GGCACTAGCG CGGTGGTCCC ACCTGACCCC ATGCCGAACT CAGAAGTGAA 240

ACGCCGTAGC GCCGATGGTA GTGTGGGGTC TCCCCATGCG AGAGTAGGGA ACTGCCAGGC 300

ATCAAATTAG AAGTATGGCA TTAACTTTCG GGGCAGTTCA CTACTCGACA GAGAGTAGGA 360

CAAAAGCGAA AGCTTTTGAA CGTTGCGAAG CAACGGCCCG AAGGGTGAAT CACGAAGTGA 420

TTCATAAACT GCCAGGCATC AAATTAAGCA GTAAACTGAT GTAAAAATCA GTGGTTGTAA 480

AAGAATTCGG TGGAGCGG 498

>(84)C Paratyphi_B_LR134233

ATGTTTTGGC GGACTGAGAA GACGAACAAT TTTCAGCACT GATTCCGGAT TTTCGGGAAC 60

GAAAGATTTT ACGCTGAGGC AAGGCGGCAA ATGAAGGAAA GGAAGGAGCA TACAGAAGTA 120

TGTGACTGAC TTTACGAATG CAGCCAACGC AGCATCGGTG TAAAAGATTC GTTTCTGGCA 180

ACAGAATTTG CCTGGCGGCA CTAGCGCGGT GGTCCCACCT GACCCCATGC CGAACTCAGA 240

AGTGAAACGC CGTAGCGCCG ATGGTAGTGT GGGGTCTCCC CATGCGAGAG TAGGGAACTG 300

CCAGGCATCA AATTAAGCAG TAAACTGATG TAAAAATCAG TGGTTGTAAA AGAATTCGGT 360

GGAGCGG 367

>(85)C Newport_NoNCBImatch

ATGTTTTGGC GGTTGAGAGA CGATTTTCAG CACTGATTCA GAGTTGAGTA CGCAATAATT 60

TGCGCAGCAG CAAGGCGGCA AGCGAAGGAA AGGAAGGAGC ATACAGAAGT ATGTGACTGA 120

CTTTACGAGC GCAGGCAACG CCGCTGATGC GATAAAGAAT TGCGTACAGA GCACAAAGAA 180

TTTGCCTGGC GGCACTAGCG CGGTGGTCCC ACCTGACCCC ATGCCGAACT CAGAAGTGAA 240

ACGCCGTAGC GCCGATGGTA GTGTGGGGTC TCCCCATGCG AGAGTAGGGA ACTGCCAGGC 300

ATCAAATTAG AAGTATGGCA TTAACTTTCG GGGCAGTTCA CTACTCGACA GAGAGTAGGA 360

CAAAAGCGAA AGCTTTTGAA CGTTGCGAAG CAACGGCCCG AAGGGTGAAT CACGAAGTGA 420

TTCATAAACT GCCAGGCATC AAATTGCAGT AAACCGGGAT GAAAATCCGG GTTGTGACAA 480

AGAAATTCGG TGGAGCGG 498

>(86)C Hadar_CP082396

ATGTTTTGGC GGTTGAGAGA CGATTTTCAG CTCAGATTCA GAGTCCGAAG GATTTTACGC 60

TGAGACAAGG CGGCAAACGC AGCGACGGAA GGAGCATACA GAAGTATGTG ACTGACGCTC 120

GCAAGAGCAG CCAACGCCGT ATCAGTGTAA AAGACACAGG ACAGAGCACA AAGAATTTGC 180

CTGGCGGCAC TAGCGCGGTG GTCCCACCTG ACCCCATGCC GAACTCAGAA GTGAAACGCC 240

GTAGCGCCGA TGGTAGTGTG GGGTCTCCCC ATGCGAGAGT AGGGAACTGC CAGGCATCAA 300

ATTAGAAGTA TGGCATTAAC TTTCGGGGCA GTTCACTACT CGACAGAGAG TAGGACAAAA 360

GCGAAAGCTT TTGAACGTTG CGAAGCAACG GCCCGAAGGG TGAATCACGA AGTGATTCAT 420

AAACTGCCAG GCATCAAATT AAGCAGTAAA CTGATGTAAA AATCAGTGGT TGTAAAAGAA 480

TTCGGTGGAG CGG 493

>(87)X Newport_Bardo_CP016010_CP019404

GGAAGAGATA AGTGCTGAAA GCATCTAAGC ACGAAACTTG CCCCGAGATG AGTTCTCCCT 60

GAGACTTAGA GTCTCCTGAA GGAACGTTGA AGACGACGAC GTTGATAGGC CGGGTGTGTA 120

AGCGCAGCGA TGCGTTGAGC TAACCGGTAC TAATGAACCG TGAGGCTTAA CCTTACAACG 180

CCGAAGATGT TTTGGCGGAT GAGAGAAGAT TTTCAGCACT GATTCAGAGT TGAGTACGCA 240

ATAATTTGCG CAGCAGCAAG GCGGCAAGCG AAGGAAAGGA AGGAGCATAC AGAAGTATGT 300

GACTGAGTTT ACGAGCGCAG GCAACGCCGC TGATGCGATA AAGAATTGCG TACAGAGCAC 360

AAAGAATTTG CCTGGCGGCA CTAGCGCGGT GGTCCCACCT GACCCCATGC CGAACTCAGA 420

AGTGAAACGC CGTAGCGCCG ATGGTAGTGT GGGGTCTCCC CATGCGAGAG TAGGGAACTG 480

CCAGGCATCA AATTAGAAGT ATGGCATTAA CTTTCGGGGC AGTTCACTAC TCGACAGAGA 540

GTAGGACAAA AGCGAAAGCT TTTGAACGTT GCGAAGCAAC GGCCCGAAGG GTGAATCACG 600

AAGTGATTCA TAAACTGCCA GGCATCAAAT TGCAGTAAAC CGGGATGAAA ATCCGGGTTG 660

TGACAAAGAA ATTCGGTGGA GCGGTAGTTC AGTTGGTTAG AATACCTGCC TGTCACGCAG 720

GGGGTCGCGG GTTCGAGTCC CGTCCGTTCC GCCACTTATT AAGAGCCCTG AGCATTAGCT 780

CAGGGCTTTT TCTTTGCCTG CGATTTAATT ATTGCTCAAT TCGCAAAAAT CCTCTGCATT 840

TCTCGCTCTT TTTCTACAAA GCGCTGAAGG TCATAATCCC CTCAGTTAAC GAACATAGTG 900

ATTAAAAAGA GGAATTCTAT GACTATCCCT GCATTCGGTT TAGGTACTGT TCGCCTGAAA 960

GACGACGTGG TTATCGCTTC TGTGAAAACG GCGCTTGAAC TGGGCTACCG TGCTGTCGAC 1020

ACTGCGCAAA TCTATGATAA TGAAGCGGCT GTTGGACAGG CGATTACTGA GAGCGGCGTA 1080

CCACGTAGCG AGCTGTACAT CACCACCAAG ATCTGGATTG AAAACCTTAG CAAAGACAGC 1140

TAATCCCAGC CTGAAAGAGA CCTGAAAAAC TGCGTACCGA TATGTCGATC TGACCCTGAT 1200

CACTGGCATC TCCGGTGATG CTGGGCTGTC GAAAATTATG CAGCATGCTG GAGCTAAAAA 1260

CAAGGCTGAT CGC 1273

>(88)C Rissen_CP043509

ATGTTTTGGC GGATGAGAAG ACGATTTTCA GCACTGATTC AGAGTTGAGT ACGCAATAAT 60

TTGCGCAGCA GCAAGGCGGC AAGCGAAGGA AAGGAAGGAG CATACAGAAG TATGTGACTG 120

AGTTTACGAG CGCAGGCAAC GCCGCTGATG CGATAAAGAA TTGCGTACTG AGCACAAAAG 180

AATTTGCCTG GCGGCCGTAG CGCGGTGGTC CCACCTGACC CCATGCCGAA CTCAGAAGTG 240

AAACGCCGTA GCGCCGATGG TAGTGTGGGG TTTCCCCATG CGAGAGTAGG GAACTGCCAG 300

GCATCAAATT AGAAGTATGG CATTAACTTT CGGGGCAGTT CACTACTCGA CAGAGAGTAG 360

GACAAAAGCG AAAGCTTTTG AACGTTGCGA AGCAACGGCC CGAAGGGTGA ATCACGAAGT 420

GATTCATAAA CTGCCAGGCA TCAAATTAAG CAGTAAACTG ATGTAAAAAT CAGTGGTTGT 480

AAAAGAATTC GGTGGAGCGG 500

>(89)C Livingstone_NoNCBImatch

ATGTTTTGGC GGTTGAGAGA TGATTTTCAG CACTGATTCA GAGTTGAGTA CGCAATAATT 60

TGCGCAGCAG CAAGGCGGCA AGCGAAGGAA AGGAAGGAGC ATACAGAAGT ATGTGACTGA 120

GTTTACGAGC GCAGGCAACG CCGCTGATGC GATAAAGAAT TGCGTACAGA GCACAAAGAA 180

TTTGCCTGGC GGCACTAGCG CGGTGGTCCC ACCTGACCCC ATACCGAACT CAGAAGTGAA 240

ACGCCGTAGC GCCGATGGTA GTGTGGGGTC TCCCCATGCG AGAGTAGGGA ACTGCCAGGC 300

ATCAAATTAG AAGTATGGCA TTAACTTTCG GGGCAGTTCA CTACTCGACA GAGAGTAGGA 360

CAAAAGCGAA AGCTTTTGAA CGTTGCGAAG CAACGGCCCG AAGGGTGAAT CACGAAGTGA 420

TTCATAAACT GCCAGGCATC AAATTGCAGT AAACTGATGT AAAAATCAGT GGTTGTAAAA 480

GAATTCGGTG GAGCGG 496

>(90)C IG_rough_Genovar_9261_NoNCBImatch

ATGTTTTGGC GGATGAGAGA TGATTTTCAG CACTGATTCC GGATTTTCGG GAACGAAAGA 60

TTTTACGCTG AGGCAAGGCG GCAAATGAAG GAAAGGAAGG AGCATACAGA AGTATGTGAC 120

TGACTTTGCG AATGCAGCCA ACGCAGCATC GGTGTAAAAG ATTCGTTTCC GGCAACAGAA 180

TTTGCCTGGC GGCACTAGCG CGGTGGTCCC ACCTGACCCC ATGCCGAACT CAGAAGTGAA 240

ACGCCGTAGC GCCGATGGTA GTGTGGGGTC TCCCCATGCG AGAGTAGGGA ACTGCCAGGC 300

ATCAAATTAG AAGTATGGCA TTAACTTTCG GGGCAGTTCA CTACTCGACA GAGAGTAGAA 360

CAAAAGCGAA AGCTTTTGAA CGTTGCGAAG CAACGGCCCG AAGGGTGAAT CACGAAGTGA 420

TTCATAAACT GCCAGGCATC AAATTGCAGT AAACCGGGAT GAAAATCCGG GTTGTGACAA 480

AGAAATTCGG TGGAGCGG 498

>(91)C Bareilly_NoNCBImatch

ATGTTTTGGC GGATGAGAGA CGATTTTCAG CACTGATTCA GAGTTGAGTA CGCAATAATT 60

TGCGCAGCAG CAAGGCGGCA AGCGAAGGAA AGGCAGGAGC ATACAGAAGT ATGTGACTGA 120

CTTTACGAGC GCAGGCAACG CCGCTGATGC GATAAAGAAT TGCGTACAGA GCACAAAAGA 180

ATTTGCCTGG CGGCACTAGC GCGGTGGTCC CACCTGACCC CATGCCGAAC TCAGAAGTGA 240

AACGCCGTAG CGCCGATGGT AGTGTGGGGT CTCCCCATGC GAGAGTAGGG AACTGCCAGG 300

CATCAAATTA GAAGTATGGC ATTAACTTTC GGGGCAGTTC ACTACTCGAC AGAGAGTAGG 360

ACAAAAGCGA AAGCTTTTGA ACGTTGCGAA GCAACGGCCC GAAGGGTGAA TCACGAAGTG 420

ATTCATAAAC TGCCAGGCAT CAATTTAAGC AGTAAACTGA TGTAAAAATC AGTGATTGTA 480

AAAGAATTCG GTGGAGCGG 499

>(92)X Saintpaul_Stanleyville_CP017727_CP034716

GAAGAGATAA GTGCTGAAAG CATCTAAGCA CGAAACTTGC CCCGAGATGA GTTCTCCCTG 60

AGACTTAGAG TCTCCTGAAG GAACGTTGAA GACGACGACG TTGATAGGCC GGGTGTGTAA 120

GCGCAGCGAT GCGTTGAGCT AACCGGTACT AATGAACCGT GAGGCTTAAC CTTACAACGC 180

CGAAGCTGTT TTGGCGGATG AGAGACGATT TTCAGCACTG ATTCCGGATT TTCGGGAACG 240

AAAGATTTTA CGCTGAGGCA AGGCGGCAAA TGAAGGAAAG GAAGGAGCAT ACAGAAGTAT 300

GTGACTGACT TTGCGAATGC AGCCAACGCA GCATCGGTGT AAAAGATTCG TTTCCGGTAA 360

CAGAATTTGC CTGGCGGCAC TAGCGCGGTG GTCCCACCTG ACCCCATGCC GAACTCAGAA 420

GTGAAACGCC GTAGCGCCGA TGGTAGTGTG GGGTCTCCCC ATGCGAGAGT AGGGAACTGC 480

CAGGCATCAA ATTAAGCAGT AAACTGATGT AAAAATCAGT GGTTGTAAAA GAATTCGGTG 540

GAGCGGTAGT TCAGTTGGTT AGAATACCTG CCTGTCACGC AGGGGGTCGC GGGTTCGAGT 600

CCCGTCCGTT CCGCCACTTA TTAAGAGCCC TGAGCATTAG CTCAGGGCTT TTTCTTTGCA 660

TGTCATTTAA TTATTGCTCA ATTCGCAAAA ATCCTCTGCA TTTCTCGCTC TTTTTCTGCA 720

AAGCGCTGAA GGTCATAATC CCCTCAGTTA ACGAACATAG TGATTAAAAA GAGGAATTCT 780

ATGACTATCC CTGCATTCGG TTTAGGTACT TTTCGCCTGA AAGACGACGT GGTTATCGCT 840

TCTGTTAAAA CGGCGCTTGA ACTGGGCTAC CGTGCTGTCG ACACTGCACA GATCTATGAT 900

AATGAAGCGG CTGTTGGACA GGCGATTGCT GAGAGCGGCG TACCGCGTAG CGAGCTGTAC 960

ATCACCACCA AGATCTGGAT TGAAAACCTT AGCAAAGACA AGCTAATCCC AAGCCTGAAA 1020

GAGAGCCTGA AAAAACTGCG TACCGAATAT GTCGATCTGA CGCTGATCCA CTGGCCATCT 1080

CCGGGTGATG CCGTGTCTGT CGAAGAATTT ATGCAGGCAT TGCTGGAAGC TAAAAACAAG 1140

GGCTGACTCG CGAAATTGGC ATTTCAACTT CACCATTCCG TTGATGAAAA AGCTATTGCC 1200

GCGGGGGCGC AGACATATCG CACCACCAGA TGAGCTTTCT CCGATCTCAG ATCTAAGGGT 1260

GAGTGGGCGA 1270

>(93)C Babelberg_NoNCBImatch

ATGTTTTGGC GGTGAGAGAC GATTTTCAGC ACTGATTCAG AGTTGAGTAC GCAATAATTT 60

GCGCAGCAGC AAGGCGGCAA GCGAAGGAAA GGAAGGAGCA TACAGAAGTA TGTGACTGAG 120

TTTACGAGCG CAGGCAACGC CGCTGATGCG ATAAAGAATT GCGTACAGAG CACAAAGAAT 180

TTGCCTGGCG GCACTAGCGC GGTGGTCCCA CCTGACCCCA TGCCGAACTC AGAAGTGAAA 240

CGCCGTAGCG CCGATGGTAG TGTGGGGTCT CCCCATGCGA GAGTAGGGAA CTGCCAGGCA 300

TCAAATTAGA AGTATGGCAT TAACTTTCGG GGCAGTTCAC TACTCGACAG AGAGTAGGAC 360

AAAAGCGAAA GCTTTTGAAC GTTGCGAAGC AACGGCCCGA AGGGTGAATC ACGAAGTGAT 420

TCATAAACTG CCAGGCATCA AATTGCAGTA AACCGGGATG AAAATCCGGG TGGTGACAAA 480

GAAATTCGGT GGAGCGG 497

>(94)C Montevideo_CP074322

ATGTTTTGGC GGTTGAGAGA CGATTTTCAG CACTGATTCA GAGTTGAGTA CGCAATAATT 60

TGCGCAGCAG CAAGGCGGCA AGCGAAGGAA AGGAAGGAGC ATACAGAAGT ATGTGACTGA 120

CTTTACGAGC GCAGGCAACG CCGCTGATGC GATAAAGAAT TGCGTACAGA GCACAAAGAA 180

TTTGCCTGGC GGCACTAGCG CGGTGGTCCC ACCTGACCCC ATGCCGAACT CAGAAGTGAA 240

ACGCCGTAGC GCCGATGGTA GTGTGGGGTC TCCCCATGCG AGAGTAGGGA ACTGCCAGGC 300

ATCAAATTAG AAGTATGGCA TTAACTTTCG GGGCAGTTCA CTACTCGACA GAGAGTAGGA 360

CAAAAGCGAA AGCTTTTGAA CGTTGCGAAG CAACGGCCCG AAGGGTGAAT CACGAAGTGA 420

TTTATAAACT GCCAGGCATC AAATTAAGCA GTAAACTGAT GTAAAAATCA GTGGTTGTAA 480

AAGAATTCGG TGGAGCGG 498

>(95)C Oranienburg_NoNCBImatch

ATGTTTTGGC GGATTGAGAG ATGATTTTCA GCACTGATTC AGAGTTGAGT ACGCAATAAT 60

TTGCGCAGCA GCAAGGCGGC AAGCGAAGGA AAGGAAGGAG CATACAGAAG TATGTGACTG 120

ACTTTACGAG CGCAGGCAAC GCCGCTGATG CGATAAAGAA TTGCGTACTG AGCATAAAAG 180

AATTTGCCTG GCGGCAGTAG CGCGGTGGTC CCACCTGACC CCATGCCGAA CTCAGAAGTG 240

AAACGCCGTA GCGCCGATGG TAGTGTGGGG TCTCCCCATG CGAGAGTAGG GAACTGCCAG 300

GCATCAAATT AAGCAGTAAG CCGGTCATAA AACCGGTGGT TGTAAAAGAA TTCGGTGGAG 360

CGG 363

>(96)C Idikan_NoNCBImatch

ATGTTTTGGC GGTTGAGAGA AGATTTTCAG CACTGATTCC GGATTTTCGG GAACGAAAGA 60

TTTTACGCTG AGGCAAGGCG GCAAATGAAG GAAAGGAAGG AGCATACAGA AGTATGTGAC 120

TGACTTTGCG AATGCAGCCA ACGCAGCATC GGTGTAAAAG ATTCGTTTCC GGTAACAGAA 180

TTTGCCTGGC GGCACTAGCG CGGTGGTCCC ACCTGACCCC ATGCCGAACT CAGAAGTGAA 240

ACGCCGTAGC GCCGATGGTA GTGTGGGGTC TCCCCATGCG AGAGTAGGGA ACTGCCAGGC 300

ATCAAATTAG AAGTATGGCA TTAACTTTCG GGGCAGTTCA CTACTCGACA GAGAGTAGGA 360

CAAAAGCGAA AGCTTTTGAA CGTTGCGAAG CAACGGCCCG AAGGGTGAAT CACGAAGTGA 420

TTCATAAACT GCCAGGCATC AAATTAAGCA GTAAACTGAT GTAAAAATCA GTGGTTGTAA 480

AAGAATTCGG TGGAGCGG 498

>(97)X Manchester_CP019414

CCGGTAGCTA AATGCGGAAG AGATAAGTGC TGAAAGCATC TAAGCACGAA ACTTGCCCCG 60

AGATGAGTTC TCCCTGAGAC TTTAGGTCTC CTGAAGGAAC GTTGAAGACG ACGACGTTGA 120

TAGGCCGGGT GTGTAAGCGC AGCGATGCGT TGAGCTAACC GGTACTAATG AACCGTGAGG 180

CTTAACCTTA CAACGCCGAA GATGTTTTGG CGGATGAGAG ATGATTTTCA GCACTGATTC 240

AGAGTTGAGT ACGCAATAAT TTGCGCAGCA GCAAGGCGGC AAGCGAAGGA AAGGAAGGAG 300

CATACAGAAG TATGTGACTG AGTTTACGAG CGCAGGCAAC GCCGCTGATG CGATAAAGAA 360

TTGCGTACAG AGCACAAAGA ATTTGCCTGG CGGCACTAGC GCGGTGGTCC CACCTGACCC 420

CATGCCGAAC TCAGAAGTGA AACGCCGTAG CGCCGATGGT AGTGTGGGGT CTCCCCATGC 480

GAGAGTAGGG AACTGCCAGG CATCAAATTA GAAGTATGGC ATTAACTTTC GGGGCAGTTC 540

ACTACTCGAC AGAGAGTAGG ACAAAAGCGA AAGCTTTTGA ACGTTGCGAA GCAACGGCCC 600

GAAGGGTGAA TCACGAAGTG ATTCATAAAC TGCCAGGCAT CAAATTAAGC AGTAAACTGA 660

TGTAAAAATC AGTGGTTGTA AAAGAATTCG GTGGAGCGGT AGTTCAGTTG GTTAGAATAC 720

CTGCCTGTCA CGCAGGGGGT CGCGGGTTCG AGTCCCGTCC GTTCCGCCAC TTATTAAGAG 780

CCCTGAGCAT TAGCTCAGGG CTTTTTCTTT GCCTGTCATT TAATTATTGC TCAATTCGCA 840

AAAATCCTCT GCATTTCTCG CTCTTTTTCT GCAAAGCGCT GAAGGTCATA ATCCCCTCAG 900

TTAACGAACA TAGTGATTAA AAAGAGGAAT TCTATGACTA TCCCTGCATT CGGTTTAGGT 960

ACTTTTCGCC TGAAAGACGA CGTGGTTATC GCTTCTGTTA AAACGGCGCT TGAACTGGGC 1020

TACCGTGCTG TCGACACTGC GCAAATCTAT GATAATGAAG CGGCTGTTGG ACAGGCGATT 1080

GCTGAGAGCG GCGTACCGCG TAATGAGCTG TACATCACCA CCAAGATCTG GATT 1134

>(98)C Give_LS483463

ATGTTTTGGC GGATTGAGAG ATGATTTTCA GCACTGATTC AGATTACATC AGAACGTAAA   60

AACGGTCTGA TAACAGAATT TGCCTGGCGG CAGTAGCGCG GTGGTCCCAC CTGACCCCAT 120

GCCGAACTCA GAAGTGAAAC GCCGTAGCGC CGATGGTAGT GTGGGGTCTC CCCATGCGAG 180

AGTAGGGAAC TGCCAGGCAT CAAATTAGAA GTATGGCATT AACTTTCGGG GCAGTTCACT 240

ACTCGACAGA GAGTAGGACA AAAGCGAAAG CTTTTGAACG TTGCGAAGCA ACGGCCCGAA 300

GGGTGAATCA CGAAGTGATT CATAAACTGC CAGGCATCAA ATTAAGCAGT AAGCCGGTCA 360

TAAACCGGTG GTTGTAAAAG AATTCGGTGG AGCGG 395

>(99)X_Brandenburg_Eastbourne_Reading_Sandiego_CP030002_CP075115_CP093134_CP075039

TCGGTACGCA GTTTTTTCAG GCTCTCTTTC AGGCTTGGGA TCAGCTTGTC TTTGCTAAGG 60

TTTTCAATCC AGATCTTAGT GGTGATGTAC AGCTCATTAC GCGGTACGCC GCTCTCAGCA 120

ATCGCCTGTC CAACAGCAAC TTCATTATCA TAGATTTGCG CAGTGTCGAC AGCACGGTAG 180

CCCAGTTCAA GCGCCGTTTT CACAGAAGCG ATAACCACGT CGTCTTTCAG GCGAAAAGTA 240

CCTAAACCGA ATGCAGGGAT AGTCATAGAA TTCCTCTTTT TAATCACTAT GTTCGTTAAC 300

TGAGGGGATT ATGACCTTCA GCGCTTTGTA GAAAAAGAGC GAGAAATGCA GAGGATTTTT 360

GCGAATTGAG CAATAATTAA ATGACAGGCA AAGAAAAAGC CCTGAGCTAA TGCTCAGGGC 420

TCTTAATAAG TGGCGGAACG GACGGGACTC GAACCCGCGA CCCCCTGCGT GACAGGCAGG 480

TATTCTAACC AACTGAACTA CCGCTCCACC GAATTTTTTT ACAACCACCA GTTTATGACC 540

GGCTTACTGC TTAATTTGAT GCCTGGCAGT TTATCAATCA CTTCGTGATT CACCCTTCGG 600

GCCGTTGCTT CGCAACGTTC AAAAGCTTTC GCTTTTGTCC TACTCTCTGT CGAGTAGTGA 660

ACTGCCCCGA AAGTTAATGC CATACTTCTA ATTTGATGCC TGGCAGTTCC CTACTCTCGC 720

ATGGGGAGAC CCCACACTAC CATCGGCGCT ACGGCGTTTC ACTTCTGAGT TCGGCATGGG 780

GTCAGGTGGG ACCACCGCGC TAGTGCCGCC AGGCAAATTC TGTTGCCAGA AACGAATCTC 840

TTATCTTTCG TTCCCGAAAA TCCGGAATCA GTGCTGAAAA TCGTCTCTCA ATCCGCCAAA 900

ACATCTTCGG CGTTGTAAGG TTAAGCCTCA CGGTTCATTA GTACCGGTTA GCTCAACGCA 960

TCGCTGCGCT TACACACCCG GCCTATCAAC GTCGTCGTCT TCAACGTTCC TTCAGGAGAC 1020

TCTAAGTCTC AGGGAGAACT CATCTCGGGG CAAGTTTCGT GCTTAGATGC TTTCAGCACT 1080

TATCTCTTCC GCATTTAGCT ACCGGGCAGT GCCATTGGCA TGACAACCCG AACACCAGTG 1140

ATGCGTCCAC TCCGGTCCTC TCGTACTAGG AGCAGCCCCC CTCAGTTCTC CAGCGCCCAC 1200

GGCAGATAGG GACCGAACTG TCTCACGACG TTCTAAACCC AGCTCGCGTA CCACTTTAAA 1260

TGGCGAACAG CCATACCCTT GGGACCTACT TCAGCCCCAG GATGTGATGA GCCGACATCG 1320

AGGTGCCAAA CACCGCCGTC GATATGAACT CTTGGGCGGT ATCAGCCTGT TATCCCCGGA 1380

GTACCTTTTA TCCGTTGAGC GATG 1404

>(100)C Cubana_NoNCBImatch

ATGTTTTGGC GGTTGAGAGA CGATTTTCAG CAGTGATTCA GAGTTGAGTA CGCAATGATT 60

TGCGCAGCAG CAAGGCGGCA AGCGAAGGAA AGGAAGGAGC ATACTGAAGT ATGTGACTGA 120

CTTTACGAGC GCAGGCAACG CCGCTGATGC GATAAAGAAT TGCGTACAGA GCACAAAGAA 180

TTTGCCTGGC GGCCGTAGCG CGGTGGTCCC ACCTGACCCC ATGCCGAACT CAGAAGTGAA 240

ACGCCGTAGC GCCGATGGTA GTGTGGGGTC TCCCCATGCG AGAGTAGGGA ACTGCCAGGC 300

ATCAAATTAG AAGTATGGCA TTAACTTTCG GGGCAGTTCA CTACTCGACA GAGAGTAGGA 360

CAAAAGCGAA AGCTTTTGAA CGTTGCGAAG CAACGGCCCG AAGGGTGAAT CACGAAGTGA 420

TTCATAAACT GCCAGGCATC AAATTAAGCA GTAAACTGAT GTAAAAATCA GTGGTTGTAA 480

AAGAATTCGG TGGAGCGG 498

>(101)C Pomona_CP019186

ATGTTTTGGC GGATGAGAGA TGATTTTCAG CGCTGATTCA GATTACATCA GAACGTAAAA 60

ACGGTCTGAT AACAGAATTT GCCTGGCGGC ACTAGCGCGG TGGTCCCACC TGACCCCATG 120

CCGAACTCAG AAGTGAAACG CCGTAGCGCC GATGGTAGTG TGGGGTCTCC CCATGCGAGA 180

GTAGGGAACT GCCAGGCATC AAATTAGAAG TATGGCATTA ACTTTCGGGG CAGTTCACTA 240

CTCGACAGAG AGTAGGACAA AAGCGAAAGC TTTTGAACGT TGCGAAGCAA CGGCCCGAAG 300

GGTGAATCAC GAAGTGATTC ATAAACTGCC AGGCATCAAA TTAAGCAGTA AGCCGGTCAT 360

AAACTGGTGG TTGTAAAAAA ATTCGGTGGA GCGG 394

>(102)C Orion_NoNCBImatch

ATGTTTTGGC GGATGAGAAG ACGATTTTCA GCAGTGATTC AGAGTTGAGT ACGCAATAAT 60

TTGCGCAGCA GCAAGGCGGC AAGCGAAGGA AAGGAAGGAG CATACAGAAG TATGTGACTG 120

AGTTTACGAG CGCAGGCAAC GCCGCTGATG CGATAAAGAA TTGCGTACAG AGCACAAAGA 180

ATTTGCCTGG CGGCACTAGC GCGGTGGTCC CACCTGACCC CATGCCGAAC TCAGAAGTGA 240

AACGCCGTAG CGCCGATGGT AGTGTGGGGT CTCCCCATGC GAGAGTAGGG AACTGCCAGG 300

CATCAAATTA AGCAGTAAGC CGGTCATAGA CTGGTGGTTG TAAAAGAATT CGGTGGAGCG 360

G 361

>(103)C Adelaide_NoNCBImatch

ATGTTTTGGC GGATGAGAGA CGATTTTCAG CACTGATTCA GAGTTGAGTA CGCAATAATT 60

TGCGCAGCAG CAAGGCGGCA AGCGAAGGAA AGGAAGGAGC ATACAGAAGT ATGTGACTGA 120

GTTTACGAGC GCAGGCAACG CCGCTGATGC GATAAAGAAT TGCGTACAGA GCACAAAGAA 180

TTTGCCTGGC GGCACTAGCG CGGTGGTCCC ACCTGACCCC ATGCCGAACT CAGAAGTGAA 240

ACGCCGTAGC GCCGATGGTA GTGTGGGGTC TCCCCATGCG AGAGTAGGGA ACTGCCAGGC 300

ATCAAATTAG AAGTATGGCA TTAACTTTCG AGGCAGTTCA CTACTCGACA GAGAGTAGGA 360

CAAAAGCGAA AGCTTTTGAA CGTTGCGAAG CAACGGCCCG AAGGGGGAAT CACGAAGTGA 420

TTCATAAACT GCCAGGCATC AATTTAAGCA GTAAACTGAT GTAAAAATCA GTGATTGTAA 480

AAGAAATTCG GTGGAGCGG 499

>(104)C Liverpool_NoNCBImatch

ATGTTTTGGC GGATGAGAGA CGATTTTCAG CACTGATTCC GGATTTTCGG GAACGAAAGA 60

TTTTACGCTG AGGCAAGGCG GAAAATGAAG GAAAGGAAGG AGCATACAGA AGTATGTGAC 120

TGACTTTGCG AATGCAGCCA ACGCAGCATC GGTGTAAAAG ATTCGTTTCC GGCAACAGAA 180

TTTGCCTGGC GGCACTAGCG CGGTGGTCCC ACCTGACCCC ATGCCGAACT CAGAAGTGAA 240

ACGCCGTAGC GCCGATGGTA GTGTGGGGTC TCCCCATGCG AGAGTAGGGA ACTGCCAGGC 300

ATCAAATTAA GCAGTAAACT GATGTAAAAA TCAGTGGTTG TAAAAGAATT CGGTGGAGCG 360

G 361

>(105)C Ouakam_CP022116

ATGTTTTGGC GGATTGAGAA GACAACAATT TTCAGCTCAG ATTCAGAGTC CGAAGGATTT 60

TACGCTGAGA CAAGGCGGCA AACGCAGCGA CGGAAGGAGC ATACAGAAGT ATGTGACTGA 120

CGCCCGCAAG AGCAGCCAAC GCCGTATCAG TGTAAAAGAC ACAGGACAGA GCACAAAGAA 180

TTTGCCTGGC GGCACTAGCG CGGTGGTCCC ACCTGACCCC ATGCCGAACT CAGAAGTGAA 240

ACGCCGTAGC GCCGATGGTA GTGTGGGGTC TCCCCATGCG AGAGTAGGGA ACTGCCAGGC 300

ATCAAATTAG AAGTATGGCA TTAACTTTCG GGGCAGTTCA CTACTCGACA GAGAGTAGGA 360

CAAAAGCGAA AGCTTTTGAA CGTTGCGAAG CAACGGCCCG AAGGGTGAAT CACGAAGTGA 420

TTCATAAACT GCCAGGCATC AAATTAAGCA GTAAACTGAT GTAAAAATCA GTGGTTGTAA 480

AAGAAATTCG GTGGAGCGG 499

>(106)C Gallinarum_gallinarum_CP088142

ATGTTTTGGC GGATGAGAAG ACGATTTTCA GCAGTGATTC AGAGTTGAGT ACGCAATAAT 60

TTGCGCAGCA GCAAGGCGGC AAGCGAAGGA AAGGAAGGAG CATACAGAAG TATGTGACTG 120

AGTTTACGAG CGCAGGCAAC GCCGCTGATG CGATAAAGAA TTGCGTACAG AGCACAAAGA 180

ATTTGCCTGG CGGCACTAGC GCGGTGGTCC CACCTGACCC CATGCCGAAC TCAGAAGTGA 240

AACGCCGTAG CGCCGATGGT AGTGTGGGGT CTCCCCATGC GAGAGTAGGG AACTGCCAGG 300

CATCAAATTA GAAGTATGGC ATTAACTTTC GGGGCAGTTC ACTACTCGAC AGAGAGTAGG 360

ACAAAAGCGA AAGCTTTTGA ACGTTGCGAA GCAACGGCCC GAAGGGTGAA TCACGAAGTG 420

ATTCATAAAC TGCCAGGCAT CAAATTGCAG TAAACCGGGA TGAAAATCCG GGTTGTGACA 480

AAGAAATTCG GTGGAGCGG 499

>(107)C Muenchen_NoNCBImatch

ATGTTTTGGC GGTTGAGAAG ACGATTTTCA GCACTGATTC AGATTACATC AGAACGTAAA 60

AACGGTCTGA TAACAGAATT TGCCTGGCGG CACTAGCGCG GTGGTCCCAC CTGACCCCAT 120

GCCGAACTCA GAAGTGAAAC GCCGTAGCGC CGATGGTAGT GTGGGGTCTC CCCATGCGAG 180

AGTAGGGAAC TGCCAGGCAT CAAATTAGAA GTATGGCATT AACTTTCGGG GCAGTTCACT 240

ACTCGACAGA GAGTAGGACA AAAGCGAAAG CTTTTGAACG TTGCGAAGCA ACGGCCCGAA 300

GGGTGAATCA CGAAGTGATT CATAAACTGC CAGGCATCAA ATTGCAGTAA ACCGGGATGA 360

AAATCCGGGT TGTGACAAAG AAATTCGGTG GAGCGG 396

>(108)C Lindenburg_NoNCBImatch

ATGTTTTGGC GTTGAGAGAC GATTTTCAGA ACTGATTCAG AGTCCGAAGG ATTTTACGCT 60

GAGACAAGGC GGCAAACGCA GCGACGGAAG GAGCATACAG AAGTATGTGA CTGACGCTCG 120

CAAGAGCAGC CAACGCCGTA TCAGTGTAAA AGACACAGGA CAGAGCACAA AGAATTTGCC 180

TGGCGGCACT AGCGCGGTGG TCCCACCTGA CCCCATGCCG AACTCAGAAG TGAAACGCCG 240

TAGCGCCGAT GGTAGTGTGG GGTCTCCCCA TGCGAGAGTA GGGAACTGCC AGGCATCAAA 300

TTAGAAGTAT GGCATTAACT TTCGGGGCAG TTCACTACTC GACAGAGAGT AGGACAAAAG 360

CGAAAGCTTT TGAACGTTGC GAAGCAACGG CCCGAAGGGT GAATCACGAA GTGATTCATA 420

AACTGCCAGG CATCAAATTG CAGTAAACCG GATGAAAAT CCGGATTGTG ACAAAGAAAT 480

TCGGTGGAGC GG 492

>(109)C Typhimurium_NoNCBImatch

ATGTTTTGGC GGTTAAGAGA AGATTTTCAG CACTGATTCC GGATTTTCGG GAACGAAAGA 60

TTTTACGCTG AGGCAAGGCG GCAAATGAAG GAAAGGAAGG AGCATACAGA AGTATGTGAC 120

TGACTTTGCG AATGCAGCCA ACGCAGCATC GGTGTAAAAG ATTCGTTTCT GGCAACAGAA 180

TTTGCCTGGC GGCACTAGCG CGGTGGTCCC ACCTGACCCC ATGCCGAACT CAGAAGTGAA 240

ACGCCGTAGC GCCGATGGTA GTGTGGGGTC TCCCCATGCG AGAGTAGGGA ACTGCCAGGC 300

ATCAAATTAG AAGTATGGCA TTAACTTTCG GGCAGTTCA CTACTCGACA GAGAGTAGGA 360

CAAAAGCGAA AGCTTTTGAA CGTTGCGAAG CAACGGCCCG AAGGGTGAAT CACGAAGTGA 420

TTCATAAACT GCCAGGCATC AAATTAAGCA GTAAACTGAT GTAAAAATCA GTGGTTGTAA 480

AAGAATTCGG TGGAGCGG 498

>(110)C Alabama_NoNCBImatch

ATGTTTTGGC GGATGAGAAG ACGAACAATT TTCAGTACTG ATTCAGAGTT GAGTACGCAA 60

TAATTTGCGC AGCAGCAAGG CGGCAAGCGA AGGAAAGGAA GGAGCATACA GAAGTATGTG 120

ACTGAGTTTA CGAGCGCAGG CAACGCCGCT GATGCGATAA AGAATTGCGT ACAGAGCACA 180

AAGAATTTGC CTGGCGGCAC TAGCGCGGTG GTCCCACCTG ACCCCATGCC GAACTCAGAA 240

GTGAAACGCC GTAGCGCCGA TGGTAGTGTG GGGTCTCCCC ATGCGAGAGT AGGGAACTGC 300

CAGGCATCAA ATTAGAAGTA TGACATTAAC TTTCGGGGCA GTTCACTACT CGACAGAGAG 360

TAGGACAAAA GCGAAAGCTT TTGAACGTTG CGAAGCAACG GCCCGAAGGG TGAATCACGA 420

AGTGATTCAT AAACTGCCAG GCATCAAATT ATAAGCAGTA AACTGATGTA AAAATCAGTG 480

GTTGTAAAAG AATTCGGTGG AGCGG 505

>(111)C Rubislaw_CP074294

ATGTTTTGGC GGTTGAGAAG ACAGATTTTC AGCACTGATT CAGATTACAT CAGAACGTAA 60

AAACGGTCTG ATAACAGAAT TTGCCTGGCG GCAGTAGCGC GGTGGTCCCA CCTGACCCCA 120

TGCCGAACTC AGAAGTGAAA CGCCGTAGCG CCGATGGTAG TGTGGGGTCT CCCCATGCGA 180

GAGTAGGGAA CTGCCAGGCA TCAATTTAAG CAGTAAACTG ATGTAAAAAT CAGTGGTTGT 240

AAAAGAATTC GGTGGAGCGG 260

>(112)C Kiambu_CP082587

ATGTTTTGGC GGATGAGAAG ACGATTTTCA GCACTGATTC CGGATTTTCG GGAACGAAAG 60

ATTTTACGCT GAGGCAAGGC GGCAAATGAA GGAAAGGAAG GAGCATACAG AAGTATGTGA 120

CTGACTTTAC GAATGCAGCC AACGCAGCAT CGGTGTAAAA GATTCGTTTC TGGCAACAGA 180

ATTTGCCTGG CGGCACTAGC GCGGTGGTCC CACCTGACCC CATGCCGAAC TCAGAAGTGA 240

AACGCCGTAG CGCCGATGGT AGTGTGGGGT CTCCCCATGC GAGAGTAGGG AACTGCCAGG 300

CATCAAATTA GAAGTATGGC ATTAACTTTC GGGGCAGTTC ACTACTCGAC AGAGAGTAGG 360

ACAAAAGCGA AAGCTTTTGA ACGTTGCGAA GCAACGGCCC GAAGGGTGAA TCACGAAGTG 420

ATTCATAAAC TGCCAGGCAT CAAATTAAGC AGTAAACTGA TGTAAAAATC AGTGGTTGTA 480

AAAGAATTCG GTGGAGCGG 499

>(113)C Agbeni_NoNCBImatch

ATGTTTTGGC GGATGAGAAG ACAGATTTTC AGCACTGATT CAGAGTTGAG TACGCAATAA 60

TTTGCGCAGC AGCAAGGCGG CAAGCGAAGG AAAGAAAGGA GCATACTGAA GTATGTGACT 120

GACTTTACGA GCGCAGGCAA CGCCGCTGAT GCGATAAAGA ATTGCGTACA GAGCACAAAG 180

AATTTGCCTG GCGGCCGTAG CGCGGTGGTC CCACCTGACC CCATGCCGAA CTCAGAAGTG 240

AAACGCCGTA GCGCCGATGG TAGTGTGGGG TCTCCCCATG CGAGAGTAGG GAACTGCCAG 300

GCATCAAATT AGAAGTATGG CATTAACTTT CGGGGCAGTT CACTACTCGA CAGAGAGTAG 360

GACAAAAGCG AAAGCTTTTG AACGTTGCGA AGCAACGGCC CGAAGGGTGA ATCACGGAGT 420

GATTCATAAA CTGCCAGGCA TCAAATTAAG CAGTAAACTG ATGTAAAAGT CAGTGGTTGT 480

AAAAGAATTC GGTGGAGCGG 500

>(114)C Typhimurium_NoNCBImatch

ATGTTTTGGC GGATTGAGAA GACAACAATT TTCAGCTCAG ATTCAGAGTC CGAAGGATTT 60

TACGCTGAGA CAAGGCGGCA AACGCAGCGA CGGAAGGAGC ATACAGAAGT ATGTGACTGA 120

CGCTCGCAAG AGCAGCCAAC GCCGTATCAG TGTAAAAGAC ACAGGACGGA GCACAAAGAA 180

TTTGCCTGGC GGCCGTAGCG CGGTGGTCCC ACCTGACCCC ATGCCGAACT CAGAAGTGAA 240

ACGCCGTAGC GCCGATGGTA GTGTGGGGTC TCCCCATGCG AGAGTAGGGA ACTGCCAGGC 300

ATCAAATTAG AAGTATGGCA TTAACTTTCG GGGCAGTTCA CTACTCGACA GAGAGTAGGA 360

CAAAAGCGAA AGCTTTTGAA CGTTGCGAAG CAACGGCCCG AAGGGTGAAT CACGAAGTGA 420

TTCATAAACT GCCAGGCATC AAATTAAGCA GTAAACTGAT GTAAAAATCA GTGGTTGTAA 480

AAGAATTCGG TGGAGCGG 498

>(115)C Derby_NoNCBImatch

ATGTTTTGGC GGATTGAGAA GACAACAATT TTCAGCTCAG ATTCAGAGTC CGAAGGATTT 60

TACGCTGAGA CAAGGCGGCA AACGCAGCGA CGGAAGGAGC ATACAGAAGT ATGTGACTGA 120

CGCTCGCAAG AGCAGCCAAC GCCGTATCAG TGTAAAAGAC ACAGGACAGA GCACAAAGAA 180

TTTGCCTGGC GGCAGTAGCG CGGTGGTCCC ACCTGACCCC ATGCCGAACT CAGAAGTGAA 240

ACGCCGTAGC GCCGATGGTA GTGTGGGGTC TCCCCATGCG AGAGTAGGGA ACTGCCAGGC 300

ATCAAATTAG AAGTATGGCA TTAACTTTCG GGGCAGTTCA CTACTCGACA GAGAGTAGGA 360

CAAAAGCGAA AGCTTTTGAA CGTTGCGAAG CAACGGCCCG AAGGGTGAAT CACGAAGTGA 420

TTCATAAACT GCCAGGCATC AAATTGCAGT AAACCAGGAT GAAAATCCGG GTTGTGACAA 480

AGAAATTCGG TGGAGCGG 498

>(116)C Nima_NoNCBImatch

ATGTTTTGGC GGATTGAGAG AAGATTTTCA GCTCAGATTC AGAGTCCGAA GGATTTTACG 60

CTGAGACAAG GCGGCAAACG CAGCGACGGA AGGAGCATAC AGAAGTATGT GACTGACGTT 120

CGCAAGAGCA GCCAACGCCG TATCAGTGTA AAAGACACAG GACAGAGCAC AAAGAATTTG 180

CCTGGCGGCA CTAGCGCGGT GGTCCCACCT GACCCCATGC CGAACTCAGA AGTGAAACGC 240

CGTAGCGCCG ATGGTAGTGT GGGGTCTCCC CATGCGAGAG TAGGGAACTG CCAGGCATCA 300

AATTAGAAGT ATGGCATTAA CTTTCGGGGC AGTTCACTAC TCGACAGAGA GTAGGACAAA 360

AGCGAAAGCT TTTGAACGTT GCGAAGCAAC GGCCCGAAGG GTGAATCACG AAGTGATTCA 420

TAAACTGCCA GGCATCAAAT TAAGCAGTAA GCCGGTCATA AACTGGTGGT TGTAAAAAAA 480

TTCGGTGGAG CGG 493

>(117) Baranquilla_NoNCBImatch

ATGTTTTGGC GGTTGAGAAG ACAACAATTT TCAGCTCAGA TTCAGAGTCC GAAGGATTTT 60

ACGCTGAGAC AAGGCGGCAA ACGCGGCGAC GGAAGGAGCA TACAGAAGTA TGTGACTGAC 120

GTTCGCAAGA GCAGCCAACG CCGTATCAGC GTAAAAGACA CAGGACAGAG CACAAAGAAT 180

TTGCCTGGCG GCACTAGCGC GGTGGTCCCA CCTGACCCCA TGCCGAACTC AGAAGTGAAA 240

CGCCGTAGCG CCGATGGTAG TGTGGGGTCT CCCCATGCGA GAGTAGGGAA CTGCCAGGCA 300

TCAATTTAAG CAGTAAACTG ATGTAAAAAT CAGTGGTTGT AAAAGAATTC GGTGGAGCGG 360

>(118)C Javiana_CP074206

ATGTTTTGGC GGACTGAGAA GACGAACAAT TTTCAGCTTT GATTCAGAGT TGAGTACGCA 60

ATAATTTGCG CAGCAGCAAG GCGGCAAGCG AAGGAAAGGA AGGAGCATAC AGAAGTATGT 120

GACTGACTTT ACGAGCGCAG GCAACGCCGC TGATGCGATA AAGAATTGCG TACTGAGCAT 180

AAAAGAATTT GCCTGGCGGC AGTAGCGCGG TGGTCCCACC TGACCCCATG CCGAACTCAG 240

AAGTGAAACG CCGTAGCGCC GATGGTAGTG TGGGGTCTCC CCATGCGAGA GTAGGGAACT 300

GCCAGGCATC AAATTAAGCA GTAAGCCGGT CATAAACCGG TGGTTGTAAA AGAATCCGGT 360

GGAGCGG 367

>(119)X_Agona_Borreze_Muenchen_NC_011149_CP019407_CP082684

CATCGCTCAA CGGATAAAAG GTACTCCGGG GATAACAGGC TGATACCGCC CAAGAGTTCA 60

TATCGACGGC GGTGTTTGGC ACCTCGATGT CGGCTCATCA CATCCTGGGG CTGAAGTAGG 120

TCCCAAGGGT ATGGCTGTTC GCCATTTAAA GTGGTACGCG AGCTGGGTTT AGAACGTCGT 180

GAGACAGTTC GGTCCCTATC TGCCGTGGGC GCTGGAGAAC TGAGGGGGGC TGCTCCTAGT 240

ACGAGAGGAC CGGAGTGGAC GCATCACTGG TGTTCGGGTT GTCATGCCAA TGGCACTGCC 300

CGGTAGCTAA ATGCGGAAGA GATAAGTGCT GAAAGCATCT AAGCACGAAA CTTGCCCCGA 360

GATGAGTTCT CCCTGAGACT TAGAGTCTCC TGAAGGAACG TTGAAGACGA CGACGTTGAT 420

AGGCCGGGTG TGTAAGCGCA GCGATGCGTT GAGCTAACCG GTACTAATGA ACCGTGAGGC 480

TTAACCTTAC AACGCCGAAG ATGTTTTGGC GGATTGAGAA GACAACAATT TTCAGCTCAG 540

ATTCAGAGTC CGAAGGATTT TACGCTGAGA CAAGGCGGCA AACGCAGCGA CGGAAGGAGC 600

ATACAGAAGT ATGTGACTGA CGCTCGCAAG AGCAGCCAAC GCCGTATCAG TGTAAAAGAC 660

ACAGGACAGA GCACAAAGAA TTTGCCTGGC GGCAGTAGCG CGGTGGTCCC ACCTGACCCC 720

ATGCCGAACT CAGAAGTGAA ACGCCGTAGC GCCGATGGTA GTGTGGGGTC TCCCCATGCG 780

AGAGTAGGGA ACTGCCAGGC ATCAAATTAG AAGTATGGCA TTAACTTTCG AGGCAGTTCA 840

CTACTCGACA GAGAGTAGGA CAAAAGCGAA AGCTTTTGAA TGTTGCGAAG CAACGGCCCG 900

AAGGGTGAAT CACGAAGTGA TTCATAAACT GCCAGGCATC AAATTGCAGT AAACCAGGAT 960

GAAAATCCGG GTTGTGACAA AGAAATTCGG TGGAGCGGTA GTTCAGTTGG TTAGAATACC 1020

TGCCTGTCAC GCAGGGGGTC GCGGGTTCGA GTCCCGTCCG TTCCGCCACT TATTAAGAGC 1080

CCTGAGCATT AGCTCAGGGC TTTTTCTTTG CATGTCATTT AATTATTGCT CAATTCGCAA 1140

AAATCCTCTG CATTTCTCGC TCTTTTTCTG CAAAGCGCTG AAGGTCATAA TCCCCTCAGT 1200

TAACGAACAT AGTGATTAAA AAGAGGAATT CTATGACTAT CCCTGCATTC GGTTTAGGTA 1260

CTTTTCGCCT GAAAGACGAC GTGGTTATCG CTTCTGTTAA AACGGCGCTT GAACTAGGCT 1320

ACCGTGCTGT CGACACTGCG CAAATCTATG ATAATGAAGC GGCTGTTGGA CAGGCGATTG 1380

CTGAGAGCGG CGTACCGCGT AATGAGCTGT ACATCACCAC CAAGATCTGG ATTGAAAATC 1440

TTAGCAAAGA CAAGCTAATC CCAAGCCTGA AAGAGAGCCT GAAAAAACTG CGTACCGA 1498

>(120)C Havana_CP074203

ATGTTTTGGC GGATGAGAGA CGATTTTCAG CACTGATTCA GAGTTGAGTA CGCAATAATT 60

TGCGCAGCAG CAAGGCGGCA AGCGAAGGAA AGGAAGGAGC ATACAGAAGT ATGTGACTGA 120

GTTTACGAGC GCAGGCAACG CCGCTGATGC GATAAAGAAT TGCGTACAGA GCACAAAGAA 180

TTTGCCTGGC GGCCGTAGCG CGGTGGTCCC ACCTGACCCC ATGCCGAACT CAGAAGTGAA 240

ACGCCGTAGC GCCGATGGTA GTGTGGGGTC TCCCCATGCG AGAGTAGGGA ACTGCCAGGC 300

ATCAAATTAG AAGTATGGCA TTAACTTTCG GGGCAGTTCA CTACTCGACA GAGAGCAGGA 360

CAAAAGCGAA AGCTTTTGAA CGTTGCGAAG CAACGGCCCG AAGGGTGAAT CACGAAGTGA 420

TTCATAAACT GCCAGGCATC AAATTAAGCA GTAAACTGAT GTAAAAATCA GTGGTTGTAA 480

AAGAATTCGG TGGAGCGG 498

>(121)C Anatum_CP007211

ATGTTTTGGC GGATTGAGAA GACAACAATT TTCAGCTCAG ATTCAGAGTC CGAAGGATTT 60

TACGCTGAGA CAAGGCGGCA AACGCAGCGA CGGAAGGAGC ATACAGAAGT ATGTGACTGA 120

CGCTCGCAAG AGCAGCCAAC GCCGTATCAG TGTAAAAGAC ACAGGACAGA GCACAAAGAA 180

TTTGCCTGGC GGCACTAGCG CGGTGGTCCC ACCTGACCCC ATGCCGAACT CAGAAGTGAA 240

ACGCCGTAGC GCCGATGGTA GTGTGGGGTC TCCCCATGCG AGAGTAGGGA ACTGCCAGGC 300

ATCAAATTAG AAGTATGGCA TTAACTTTCG GGGCAGTTCA CTACTCGACA GAGAGTAGGA 360

CAAAAGCGAA AGCTTTTGAA CGTTGCGAAG CAACGGCCCG AAGAGTGAAT CACGAAGTGA 420

TTCATAAACT GCCAGGCATC AAATTAAGCA GTAAACTGAT GTAAAAATCA GTGGTTGTAA 480

AAGAATTCGG TGGAGCGG 498

>(122)C Bareilly_Typhimurium_Saintpaul_Virchow_CP045757_CP045945_CP023166_CP020565

ATGTTTTGGC GGATGAGAGA CGATTTTCAG CACTGATTCA GAGTTGAGTA CGCAATAATT 60

TGCGCAGCAG CAAGGCGGCA AGCGAAGGAA AGGAAGGAGC ATACAGAAGT ATGTGACTGA 120

GTTTACGAGC GCAGGCAACG CCGCTGATGC GATAAAGAAT TGCGTACAGA GCACAAAAGA 180

ATTTGCCTGG CGGCAGTAGC GCGGTGGTCC CACCTGACCC CATGCCGAAC TCAGAAGTGA 240

AACGCCGTAG CGCCGATGGT AGTGTGGGGT CTCCCCATGC GAGAGTAGGG AACTGCCAGG 300

CATCAAATTA GAAGTATGGC ATTAACTTTC GGGGCAGTTC ACTACTCGAC AGAGAGTAGG 360

ACAAAAGCGA AAGCTTTTGA ACGTTGCGAA GCAACGGCCC GAAGGGTGAA TCACGAAGTG 420

ATTCATAAAC TGCCAGGCAT CAAATTGCAG TAAACCGGGA TGAAAATCCG GGTTGTGACA 480

AAGAAATTCG GTGGAGCGG 499

>(123)C Berta_CP030005

ATGTTTTGGC GGATGAGAGA AGATTTTCAG CACTGATTCA GAGTTGAGTA CGCAATAATT 60

TGCGCAGCAG CAAGGCGGCA AGCGAAGGAA AGGAAGGAGC ATACAGAAGT ATGTGACTGA 120

GTTTACGAGC GCAGGCAACG CCGCTGATGC GATAAAGAAT TGCGTACAGA GCACAAAGAA 180

TTTGCCTGGC GGCAGTAGCG CGGTGGTCCC ACCTGACCCC ATGCCGAACT CAGAAGTGAA 240

ACGCCGTAGC GCCGATGGTA GTGTGGGGTC TCCCCATGCG AGAGTAGGGA ACTGCCAGGC 300

ATCAAATTAG AAGTATGGCA TTAACTTTCG GGGCAGTTCA CTACTCGACA GAGAGTAGGA 360

CAAAAGCGAA AGCTTTTGAA CGTTGCGAAG CAACGGCCCG AAGGGTGAAT CACGAAGTGA 420

TTCATAAACT GCCAGGCATC AAATTGCAGT AAACCGGGAT GAAAATCCGG GTTGTGACAA 480

AGAAATTCGG TGGAGCGG 498

>(124)X Choleraesuis_Gallinarum_NC_006905_CP088134

GCGGAAGAGA TAAGTGCTGA AAGCATCTAA GCACGAAACT TGCCCCGAGA TGAGTTCTCC 60

CTGAGACTTA GAGTCTCCTG AAGGAACGTT GAAGACGACG ACGTTGATAG GCCGGGTGTG 120

TAAGCGCAGC GATGCGTTGA GCTAACCGGT ACTAATGAAC CGTGAGGCTT AACCTTACAA 180

CGCCGAAGAT GTTTTGGCGG ATGAGAGACA ATTTTCAGCA CTGATTCAGA GTTGAGTACG 240

CAATAATTTG CGCAGCAGCA AGGCGGCAAG CGAAGGAAAG GAAGGAGCAT ACAGAAGTAT 300

GTGACTGACT TTACGAGCGC GGGCAACGCC GCTGATGCGA TAAAGAATTG CGTACTGAGC 360

ACAAAAGAAT TTGCCTGGCG GCACTAGCGC GGTGGTCCCA CCTGACCCCA TGCCGAACTC 420

AGAAGTGAAA CGCCGTAGCG CCGATGGTAG TGTGGGGTCT CCCCATGCGA GAGTAGGGAA 480

CTGCCAGGCA TCAAATTAGA AGTATGGTAT TAACTTTCGG GGCAGTTCAC TACTCGACAG 540

AGAGTAGGAC AAAAGCGAAA GCTTTTGAAC GTTGCGAAGC AACGGCCCGA AGGGTGAATC 600

ACGAAGTGAT TCATAAACTG CCAGGCATCA AATTGCAGTA AACCGGGATG AAAATCCGGA 660

TTGTGACAAA GAAATTCGGT GGAGCGGTAG TTCAGTTGGT TAGAATACCT GCCTGTCACG 720

CAGGGGGTCG CGGGTTCGAG TCCCGTCCGT TCCGCCACTT ATTAAGAGCC CTGAGCATTA 780

GCTCAGGGCT TTTTCTTTGC CTGTCATTTA ATTATTGCTC AATTCGCAAA AATCCTCTGC 840

ATTTCTCGCT CTTTTTCTAC AAAGCGCTGA AGGTCATAAT TCCCTCAGCT AACGAACATA 900

GTGATTAAAA AGAGGAATTC TATGACTATC CCTGCATTCG GTTTAGGTAC TTTTCGCCTG 960

AAAGACGACG TGGTTATCGC TTCTGTTAAA ACGGCGCTTG AACTGGGCTA CCGTGCTGTC 1020

GACACTGCAC AGATCTATGA TAATGAAGCG GCTGTTGGAC AGGCGATTGC TGAAAGCGGT 1080

GTACCACGTA ATGAGCTGTA CATCACCACC AAGATCTGGA TTGAAAACCT TAGCAAAGA 1139

>(125)C Cubana_NC_021818

ATGTTTTGGC GGTTGAGAGA AGATTTTCAG CACTGATTCA GATTACATCA GAACGTAAAA 60

ACGGTCTGAT AACAGAATTT GCCTGGCGGC ACTAGCACGG TGGTCCCACC TGACCCCATG 120

CCGAACTCAG AAGTGAAACG CCGTAGCGCC GATGGTAGTG TGGGGTCTCC CCATGCGAGA 180

GTAGGGAACT GCCAGGCATC AAATTAGAAG TATGGCATTA ACTTTCGGGG CAGTTCACTA 240

CTCGACAGAG AGTAGGACAA AAGCGAAAGC TTTTGAACGT TGCGAAGCAA CGGCCCGAAG 300

GGTGAATCAC GAAGTGATTC ATAAACTGCC AGGCATCAAA TTAAGCAGTA AACTGATGTA 360

AAAATCAGTG GTTGTAAAAG AATTCGGTGG AGCGG 395

>(126)C Dublin_NC_011205

ATGTTTTGGC GGATGAGAAG ACGATTTTCA GCAGTGATTC AGAGTTGAGT ACGCAATAAT 60

TTGCGCAGCA GCAAGGCGGC AAGCGAAGGA AAGGAAGGAG CATACAGAAG TATGTGACTG 120

AGTTTACGAG CGCAGGCAAC GCCGCTGATG CGATAAAGAA TTGCGTACAG AGCACAAAGA 180

ATTTGCCTGG CGGCACTAGC GCGGTGGTCC CACCTGACCC CATGCCGAAC TCAGAAGTGA 240

AACGCTGTAG CGCCGATGGT AGTGTGGGGT CTCCCCATGC GAGAGTAGGG AACTGCCAGG 300

CATCAAATTA GAAGTATGGC ATTAACTTTC GGGGCAGTTC ACTACTCGAC AGAGAGTAGG 360

ACAAAAGCGA AAGCTTTTGA ACGTTGCGAA GCAACGGCCC GAAGGGTGAA TCACGAAGTG 420

ATTCATAAAC TGCCAGGCAT CAAATTGCAG TAAACCGGGA TGAAAATCCG GGTTGTGACA 480

AAGAAATTCG GTGGAGCGG 499

>(127)X Enteritidis_Newlands_Javiana_Typhimurium_NC_011294_CP019416_CP074314_CP019383

GAAGAGATAA GTGCTGAAAG CATCTAAGCA CGAAACTTGC CCCGAGATGA GTTCTCCCTG 60

AGACTTAGAG TCTCCTGAAG GAACGTTGAA GACGACGACG TTGATAGGCC GGGTGTGTAA 120

GCGCAGCGAT GCGTTGAGCT AACCGGTACT AATGAACCGT GAGGCTTAAC CTTACAACGC 180

CGAAGATGTT TTGGCGGATG AGAAGACGAT TTTCAGCAGT GATTCAGAGT TGAGTACGCA 240

ATACTTTGCG CAGCAGCAAG GCGGCAAGCG AAGGAAAGGA AGGAGCATAC AGAAGTATGT 300

GACTGAGTTT ACGAGCGCAG GCAACGCCGC TGATGCGATA AAGAATTGCG TACAGAGCAC 360

AAAGAATTTG CCTGGCGGCA CTAGCGCGGT GGTCCCACCT GACCCCATGC CGAACTCAGA 420

AGTGAAACGC CGTAGCGCCG ATGGTAGTGT GGGGTCTCCC CATGCGAGAG TAGGGAACTG 480

CCAGGCATCA AATTAGAAGT ATGGCATTAA CTTTCGGGGC AGTTCACTAC TCGACAGAGA 540

GTAGGACAAA AGCGAAAGCT TTTGAACGTT GCGAAGCAAC GGCCCGAAGG GTGAATCACG 600

AAGTGATTCA TAAACTGCCA GGCATCAAAT TGCAGTAAAC CGGGATGAAA ATCCGGGTTG 660

TGACAAAGAA ATTCGGTGGA GCGGTAGTTC AGTTGGTTAG AATACCTGCC TGTCACGCAG 720

GGGGTCGCGG GTTCGAGTCC CGTCCGTTCC GCCACTTATT AAGAGCCCTG AGCATTAGCT 780

CAGGGCTTTT TCTTTGCATA TCATTTAATT ATTGCTCAAT TCGCAAAAAT CCTCTGCATT 840

TCTCGCTCTT TTTCTACAAA GCGCTGAAGG TCATAATCCC CTCAGTTAAC GAACATAGTG 900

ATTAAAAAGA GGAATTCTAT GACTATCCCT GCATTCGGTT TAGGTACTTT TCGCCTGAAA 960

GACGACGTGG TTATCGCTTC TGTGAAAACG GCGCTTGAAC TGGGCTACCG TGCTGTCGAC 1020

ACTGCGCAAA TCTATGATAA TGAAGCGGCT GTTGGACAGG CGATTGCTGA GAGCGGCGTA 1080

CCGCGTAATG AGCTGTACAT CACCACCAAG ATCTGGATTG AAAACCTTAG CAAAGACAAG 1140

CTAATCCCAG CCTGAAAGAG AGCCTGAAAA ACTGCGTACC GATATGTCGA TCTGACGCTG 1200

ATCACTGGCC 1210

>(128)C Gallinarum-gallinarum_NC_011274

ATGTTTTGGC GGATGAGAGA AGATTTTCAG CAGTGATTCA GAGTTGAGTA CGCAATACTT 60

TGCGCAGCAG CAAGGCGGCA AGCGAAGGAA AGGAAGGAGC ATACAGAAGT ATGTGACTGA 120

GTTTACGAGC GCAGGCAACG CCGCTGATGC GATAAAGAAT TGCGTACAGA GCACAAAGAA 180

TTTGCCTGGC GGCAGTAGCG CGGTGGTCCC ACCTGACCCC ATGCCGAACT CAGAAGTGAA 240

ACGCCGTAGC GCCGATGGTA GTGTGGGGTC TCCCCATGCG AGAGTAGGGA ACTGCCAGGC 300

ATCAAATTAG AAGTATGGCA TTAACTTTCG GGGCAGTTCA CTACTCGACA GAGAGTAGGA 360

CAAAAGCGAA AGCTTTTGAA CGTTGCGCAG CAACGGCCCG AAGGGTGAAT CACGAAGTGA 420

TTCATAAACT GCCAGGCATC AAATTGCAGT AAACCGGGAT GAAAATCCGG GTTGTGACAA 480

AGAAATTCGG TGGAGCGG 498

>(129)X Gallinarum-pullorum_NC_022221

CGGAAGAGAT AAGTGCTGAA AGCATCTAAG CACGAAACTT GCCCCGAGAT GAGCTCTCCC 60

TGAGACTTAG AGTCTCCTGA AGGAACGTTG AAGACGACGA CGTTGATAGG CCGGGTGTGT 120

AAGCGCAGCG ATGCGTTGAG CTAACCGGTA CTAATGAACC GTGAGGCTTA ACCTTACAAC 180

GCCGAAGATG TTTTGGCGGA TGAGAGACGA TTTTCAGCAG TGATTCAGAG TTGAGTACGC 240

AATAATTTGC GCAGCAGCAA GGCGGCAAGC GAAGGAAAGG AAGGAGCATA CAGAAGTATG 300

TGACTGAGTT TACGAGCGCA GGCAACGCCG CTGATGCGAT AAAGAATTGC GTACAGAGCA 360

CAAAGAATTT GCCTGGCGGC AGTAGCGCGG TGGTCCCACC TGACCCCATG CCGAACTCAG 420

AAGTGAAACG CCGTAGCGCC GATGGTAGTG TGGGGTCTCC CCATGCGAGA GTAGGGAACT 480

GCCAGGCATC AAATTAAGCA GTAAACTGAT GTAAAAATCA GTGGTTGTAA AAGAATTCGG 540

TGGAGCGGTA GTTCAGTTGG TTAGAATACC TGCCTGTCAC GCAGGGGGTC GCGGGTTCGA 600

GTCCCGTCCG TTCCGCCACT TATTAAGAGC CCTGAGCATT AGCTCAGGGC TTTTTCTTTG 660

CCTGTCATTT AATTATTGCT CAATTCGCAA AAATCCTCTG CATTTCTCGC TCTTTTTCTG 720

CAAAGCGCTG AAGGTCATAA TCCCCTCAGT TAACGAACAT AGTGATTAAA AAGAGGAATT 780

CTATGACTAT CCCTGCATTC GGTTTAGGTA CTTTTCGCCT GAAAGACGAC GTGGTTATCG 840

CTTCTGTGAA AACGGCGCTT GAACTGGGCT ACCGTGCTGT CGACACTGCG CAAATCTATG 900

ATAATGAAGC GGCTGTTGGA CAGGCGATTG CTGAGAGCGG CGTACCACGT AGCGAGCTGT 960

ACATCACCAC CAAGGTCTGG ATTGAAAACC TTAGCAAAGA CAAGCTAATC CCAAGCCTGA 1020

AAGAGAGCCT GAAAAACTGC GTACCGATTA TGTCGATCTG ACGCTGATCC ACTGGCCATC 1080

TCCGGGTGAT GCCGTGTCTG TCGAAAAATT ATGCAGGCAT TGCTGGAAGC TAAAAACAAG 1140

GGCTGACTCG CGAAATGGCA TCT 1163

>(130)X Hadar_Anatum_Nchanga_CP038595_CP074323_CP082370

TCGGTACGCA GTTTTTTCAG GCTCTCTTTC AGGCTTGGGA TTAGCTTGTC TTTGCTAAGG 60

TTTTCAATCC AGATCTTGGT GGTGATGTAC AGCTCATTAC GCGGTACGCC GCTCTCAGCA 120

ATCGCCTGTC CAACAGCCGC TTCATTATCA TAGATTTGCG CAGTGTCGAC AGCACGGTAG 180

CCCAGTTCAA GCGCCGTTTT AACAGAAGCG ATAACCACGT CGTCTTTCAG GCGAAAAGTA 240

CCTAAACCGA ATGCAGGGAT AATCATAGAA TTCCTCTTTT TAATCACTAT GTTCGTTAAC 300

TGAGGGGATT ATGACCTTCA GCGCTTTGCA GAAAAAGAGC GAGAAATGCA GAGGATTTTT 360

GCTAATTGAG CAATAATTAA ATGACATGCA AAGAAAAAGC CCTGAGCTAA TGCTCAGGGC 420

TCTTAATAAG TGGCGGAACG GACGGGACTC GAACCCGCGA CCCCCTGCGT GACAGGCAGG 480

TATTCTAACC AACTGAACTA CCGCTCCACC GAATTTCTTT GTCACAACCC GGATTTTCAT 540

CCCGGTTTAC TGCAATTTGA TGCCTGGCAG TTTATGAATC ACTTCGTGAT TCACCCTTCT 600

GGCCGTTGCT TCGCAACGTT CAAAAGCTTT CGCTTTTGTC CTACTCTCTG TCGAGTAGTG 660

AACTGCCCCG AAAGTTAATG CCATACTTCT AATTTGATGC CTGGCAGTTC CCTACTCTCG 720

CATGGGGAGA CCCCACACTA CCATCGGCGC TACGGCGTTT CACTTCTGAG TTCGGCATGG 780

GGTCAGGTGG GACCACCGCG CTAGTGCCGC CAGGCAAATT CTTTGTGCTC CGTCCTGTGT 840

CTTTTACACT GATACGGCGT TGGCTGCTCT TGCGAGCGTC AGTCACATAC TTCTGTATGC 900

TCCTTCCGTC GCTGCGTTTG CCGCCTTGTC TCAGCGTAAA ATCCTTCGGA CTCTGAATCT 960

GAGCTGAAAA TTGTTGTCTT CTCAATCCGC CAAAACATCT TCGGCGTTGT AAGGTTAAGC 1020

CTCACGGTTC ATTAGTACCG GTTAGCTCAA CGCATCGCTG CGCTTACACA CCCGGCCTAT 1080

CAACGTCGTC GTCTTCAACG TTCCTTCAGG AGACTCTAAG TCTCAGGGAG AACTCATCTC 1140

GGGGCAAGTT TCGTGCTTAG ATGCTTTCAG CACTTATCTC TTCCGCATTT AGCTACCGGG 1200

CAGTGCCATT GGCATGACAA CCCGAACACC AGTGATGCGT CCACTCCGGT CCTCTCGTAC 1260

TAGGAGCAGC CCCCCTCAGT TCTCCAGCGC CCACGGCAGA TAGGGACCGA ACTGTCTCAC 1320

GACGTTCTAA ACCCAGCTCG CGTACCACTT TAAATGGCGA ACAGCCATAC CCTTGGGACC 1380

TACTTCAGCC CCAGGATGTG ATGAGCCGAC ATCGAGGTGC CAAACACCGC CGTCGATATG 1440

AACTCTTGGG CGGTATCAGC CTGTTATCCC CGGAGTACCT TTTATCCGTT GAGCGATG 1498

>(131)C Heidelberg_Crossness_IG-4,[5],12:i:-_NC_021812_CP019408_CP082588

ATGTTTTGGC GGATTGAGAA GACAACAATT TTCAGCTCAG ATTCAGAGTC CGAAGGATTT 60

TACGCTGAGA CAAGGCGGCA AACGCAGCGA CGGAAGGAGC ATACAGAAGT ATGTGACTGA 120

CGCCCGCAAG AGCAGCCAAC GCCGTATCAG TGTAAAAGAC ACAGGACAGA GCACAAAGAA 180

TTTGCCTGGC GGCAGTAGCG CGGTGGTCCC ACCTGACCCC ATGCCGAACT CAGAAGTGAA 240

ACGCCGTAGC GCCGATGGTA GTGTGGGGTC TCCCCATGCG AGAGTAGGGA ACTGCCAGGC 300

ATCAAATTAG AAGTATGGCA TTAACTTTCG GGGCAGTTCA CTACTCGACA GAGAGTAGGA 360

CAAAAGCGAA AGCTTTTGAA CGTTGCGAAG CAACGGCCCG AAGGGTGAAT CACGAAGTGA 420

TTCATAAACT GCCAGGCATC AAATTGCAGT AAACCGGGAT GAAAATCCGG GTTGTGACAA 480

AGAAATTCGG TGGAGCGG 498

>(132)C Infantis_CP019202

ATGTTTTGGC GGTTGAGAAG ACGATTTTCA GCACTGATTC AGAGTTGAGT ACGCAATAAT 60

TTGCGCAGCA GCAAGGCGGC AAGCGAAGGA AAGGAAGGAG CATACAGAAG TATGTGACTG 120

AGTTTACGAG CGCAGGCAAC GCCGCTGATG CGATAAAGAA TTGCGTACAG AGCATAAAAG 180

AATTTGCCTG GCGGCACTAG CGCGGTGGTC CCACCTGACC CCATGCCGAA CTCAGAAGTG 240

AAACGCCGTA GCGCCGATGG TAGTGTGGGG TCTCCCCATG CGAGAGTAGG GAACTGCCAG 300

GCATCAAATT AGAAGTATGG CATTAACTTT CGGGGCAGTT CACTACTCGA CAGAGAGTAG 360

GACAAAAGCG AAAGCTTTTG AACGTTGCGA AGCAACGGCC CGAAGGGGGA ATCACGAAGT 420

GATTCATAAG CTGCCAGGCA TCAAATTGCA GTAAACCGGG ATGAAAATCC GGATTGTGAC 480

AAAGAAATTC GGTGGAGCGG 500

>(133)C Javiana_NC_020307

ATGTTTTGGC GGACTGAGAA GACGAACAAT TTTCAGCTTT GATTCAGAGT TGAGTACGCA 60

ATAATTTGCG CAGCAGCAAG GCGGCAAGCG AAGGAAAGGA AGGAGCATAC AGAAGTATGT 120

GACTGACTTT ACGAGCGCAG GCAACGCCGC TGATGCGATA AAGAATTGCG TACTGAGCAT 180

AAAAGAATTT GCCTGGCGGC ACTAGCGCGG TGGTCCCACC TGACCCCATG CCGAACTCAG 240

AAGTGAAACG CCGTAGCGCC GATGGTAGTG TGGGGTCTCC CCATGCGAGA GTAGGGAACT 300

GCCAGGCATC AAATTAAGCA GTAAGCCGGT CATAAACCGG TGGTTGTAAA AGAATCCGGT 360

GGAGCGG 367

>(134)C Johannesburg_CP019411

ATGTTTTGGC GGTTGAGAGA TGATTTTCAG CACTGATTCA GATTACATCA GAACGTAAAA 60

ACGGTCTGAT AACAGAATTT GCCTGGCGGC ACTAGCGCGG TGGTCCCACC TGACCCCATG 120

CCGAACTCAG AAGTGAAACG CCGTAGCGCC GATGGTAGTG TGGGGTCTCC CCATGCGAGA 180

GTAGGGAACT GCCAGGCATC AAATTGCAGT AAACCGGGAT GAAAATCCGG GTTGTGACAA 240

AGAAATTCGG TGGAGCGG 258

>(135)C Kentucky_CP022500

ATGTTTTGGC GTTGAGAGAC GATTTTCAGC ACTGATTCAG AGTCCGAAGG ATTTTACGCT 60

GAGACAAGGC GGCAAACGCA GCGACGGAAG GAGCATACAG AAGTATGTGA CTGACGCTCG 120

CAAGAGCAGC CAACGCCGTA TCAGTGTAAA AGACACAGGA CAGAGCACAA AGAATTTGCC 180

TGGCGGCACT AGCGCGGTGG TCCCACCTGA CCCCATGCCG AACTCAGAAG TGAAACGCCG 240

TAGCGCCGAT GGTAGTGTGG GGTCTCCCCA TGCGAGAGTA GGGAACTGCC AGGCATCAAA 300

TTAGAAGTAT GGCATTAACT TTCGGGGCAG TTCACTACTC GACAGAGAGT AGGACAAAAG 360

CGAAAGCTTT TGAACGTTGC GAAGCAACGG CCCGAAGGGT GAATCACGAA GTGATTCATA 420

AACTGCCAGG CATCAAATTG CAGTAAACCG GGATGAAAAT CCGGATTGTG ACAAAGAAAT 480

TCGGTGGAGC GG 492

>(136)X Newport_Derby_NC_021902_CP075036

CTTGCCCCGA GATGAGTTCT CCCTGAGACT TAGAGTCTCC TGAAGGAACG TTGAAGACGA 60

CGACGTTGAT AGGCCGGGTG TGTAAGCGCA GCGATGCGTT GAGCTAACCG GTACTAATGA 120

ACCGTGAGGC TTAACCTTAC AACGCCGAAG ATGTTTTGGC GGATGAGAGA CGATTTTCAG 180

CACTGATTCC GGATTTTCGG GAACGAAAGA TTTTACGCTG AGGCAAGGCG GCAAATGAAG 240

GAAAGGAAGG AGCATACAGA AGTATGTGAC TGACTTTGCG AATGCAGCCA ACGCAGCATC 300

GGTGTAAAAG ATTCGTTTCC GGTAACAGAA TTTGCCTGGC GGCACTAGCG CGGTGGTCCC 360

ACCTGACCCC ATGCCGAACT CAGAAGTGAA ACGCCGTAGC GCCGATGGTA GTGTGGGGTC 420

TCCCCATGCG AGAGTAGGGA ACTGCCAGGC ATCAAATTAG AAGTATGGCA TTAACTTTCG 480

GGGCAGTTCA CTACTCGACA GAGAGTAGGA CAAAAGCGAA AGCTTTTGAA CGTTGCGAAG 540

CAACGGCCCG AAGGGTGAAT CACGAAGTGA TTCATAAACT GCCAGGCATC AAATTAAGCA 600

GTAAACTGAT GTAAAAATCA GTGGTTGTAA AAGAATTCGG TGGAGCGGTA GTTCAGTTGG 660

TTAGAATACC TGCCTGTCAC GCAGGGGGTC GCGGGTTCGA GTCCCGTCCG TTCCGCCACT 720

TATTAAGAGC CCTGAGCATT AGCTCAGGGC TTTTTCTTTG CCTGCGATTT AATTATTGCT 780

CAATTCGCAA AAATCCTCTG CATTTCTCGC TCTTTTTCTA CGAAGCGCTG AAGGTCATAA 840

TCCCCTCAGT TAACGAACAT AGTGATTAGA AAGAGGAATT CTATGACTAT CCCTGCATTC 900

GGTTTAGGTA CTTGTCGCCT GACAGACGAC GTGGTTATCG CTTCTGTGAA AACGGCGCTT 960

GAACTGAGCT ACCGTGCTGT CGA 983

>(137)C Newport_NoNCBImatch

ATGTTTTGGC GGATGAGAGA CGATTTTCAG CTCAGATTCA GAGTCCGAAG GATTTTACGC 60

TGAGACAAGG CGGCAAACGC AGCGACGGAA GGAGCATACA GAAGTATGTG ACTGACGCTC 120

GCAAGAGCAG CCAACGCCGT ATCAGTGTAA AAGATTCGGT ACAGAGCACA AAGAATTTGC 180

CTGGCGGCAC TAGCGCGGTG GTCCCACCTG ACCCCATGCC GAACTCAGAA GTGAAACGCC 240

GTAGCGCCGA TGGTAGTGTG GGGTCTCCCC ATGCGAGAGT AGGGAACTGC CAGGCATCAA 300

ATTAGAAGTA TGGCATTAAC TTTCGGGGCA GTTCACTACT CGACAGAGAG TAGGACAAAA 360

GCGAAAGCTT TTGAACGTTG CGAAGCAACG GCCCGAAGGG TGAATCACGA AGTGATTCAT 420

AAACTGCCAG GCATCAAATT AAGCAGTAAA CTGATGTAAA AATCAGTGGT TGTAAAAGAA 480

TTCGGTGGAG CGG 493

>(138)C Paratyphi-A_NC_006511

ATGTTTTGGC GGATGAGAGA CGATTTTCAG CACTGATTCC GGATTTTCGG GAACGAAAGA 60

TTTTACGCTG AGGCAAGGCG GCAAATGAAG GAAAGGAAGA AGCATACAGA AGTATGTGAC 120

TGACTTTACG AATGCAGCCA ACGCAGCATC GGTGTAAAAG ATTCGTTTCT GGCAACAGAA 180

TTTGCCTGGC GGCACTAGCG CGGTGGTCCC ACCTGACCCC ATGCCGAACT CAGAAGTGAA 240

ACGCCGTAGC GCCGATGGTA GTGTGGGGTC TCCCCATGCG AGAGTAGGGA ACTGCCAGGC 300

ATCAAATTAG AAGTATGGCA TTAACTTTCG GGGCAGTTCA CTACTCGACA GAGAGTAGGA 360

CAAAAGCGAA AGCTTTTGAA CGTTGCGAAG CAACGGCCCG AAGGGTGAAT CACGAAGTGA 420

TTCATAAACT GCCAGGCATC AAATTAAGCA GTAAACTGAT GTAAAAATCA GTGGTTGTAA 480

AAGAATTCGG TGGAGCGG 498

>(139)X Javiana_CP085052

AAGAGATAAG TGCTGAAAGC ATCTAAGCAC GAAACTTGCC CCGAGATGAG TTCTCCCTGA 60

GACTTTAGGT CTCCTGAAGG AACGTTGAAG ACGACGACGT TGATAGGCCG GGTGTGTAAG 120

CGCAGCGATG CGTTGAGCTA ACCGGTACTA ATGAACCGTG AGGCTTAACC TTACAACGCC 180

GAAGATGTTT TGGCGGATGA GAGACGATTT TCAGCACTGA TTCAGAGTTG AGTACGCAAT 240

AATTTGCGCA GCAGCAAGGC GGCAAGCGAA GGAAAGGAAG GAGCATACAG AAGTATGTGA 300

CTGAGTTTAC GAGCGCAGGC AACGCCGCTG ATGCGATAAA GAATTGCGTA CAGAGCACAA 360

AGAATTTGCC TGGCGGCACT AGCGCGGTGG TCCCACCTGA CCCCATGCCG AACTCAGAAG 420

TGAAACGCCG TAGCGCCGAT GGTAGTGTGG GGTCTCCCCA TGCGAGAGTA GGGAACTGCC 480

AGGCATCAAA TTAGAAGTAT GGCATTAACT TTCGGGGCAG TTCACTACTC GACAGAGAGT 540

AGGACAAAAG CGAAAGCTTT TGAACGTTGC GAAGCAACGG CCCGAAGGGT GAATCACGAA 600

GTGATTCATA AACTGCCAGG CATCAAATTG CAGTAAACCG GGATGAAAAT CCGGGTTGTG 660

ACAAAGAAAT TCGGTGGAGC GGTAGTTCAG TTGGTTAGAA TACCTGCCTG TCACGCAGGG 720

GGTCGCGGGT TCGAGTCCCG TCCGTTCCGC CACTTATTAA GAGCCCTGAG CATTAGCTCA 780

GGGCTTTTTC TTTTCCTGTC ATTTAATTAT TGCTCAATTC GCAAAAATCC TCTGCATTTC 840

TCGCTCTTTT TCTGCAAAGC GCTGAAGGTC ATAATTCCCT CAGTTAACGA ACATAGTGAT 900

TAAAAAGAGG AATTCTATGA CTATCCCTGC ATTCGGTTTA GGTACTTTTC GCCTGAAAGA 960

CGACGTGGTT ATCGCTTCTG TTAAAACGGC GCTTGAACTG GGCTACCGTG CTGTCGACAC 1020

TGCGCAAATC TATGATAATG AAGCGGCTGT TGGACAGGCG ATTGCTGAGA GCGGCGTACC 1080

GCGTAGCGAG CTGTACATCA CCACCAAGAT CTGGATTGAA AACCTTAGCA AAGACAAGCT 1140

AATCCCAAGC CTGAAAGAGA GCCTGAAAAA CTGCGTACCG ATTATGTCGA TCTGACGCTG 1200

ATCCCTGGCC ATCTCCGGGT GATGCCGGTC TGTCAAGAAT T 1241

>(140)C Paratyphi-C_NC_012125

ATGTTTTGGC GGATGAGAGA CGATTTTCAG CTCAGATTCA GATTACATCA GAACGTAAAA 60

ACGGTCTGAT AACAGAATTT GCCTGGTGGC ACTAGCGCGG TGGTCCCACC TGACCCCATG 120

CCGAACTCAG AAGTGAAACG CCGTAGCGCC GATGGTAGTG TGGGGTCTCC CCATGCGAGA 180

GTAGGGAACT GCCAGGCATC AAATTAGAAG TATGGTATTA ACTTTCGAGG CAGTTCACTA 240

CTCGACAGAG AGTAGGACAA AAGCGAAAGC TTTTGAACGT TGCGAAGCAA CGGCCCGAAG 300

GGTGAATCAC GAAGTGATTC ATAAACTGCC AGGCATCAAA TTGCAGTAAA CCGGGATGAA 360

AATCCGGATT GTGACAAAGA AATTCGGTGG AGCGG 395

>(141)C Saintpaul_Stanleyville_CP019206_CP017723

ATGTTTTGGC GGATTGAGAA GACAACAATT TTCAGCTCAG ATTCAGAGTC CGAAGGATTT 60

TACGCTGAGA CAAGGCGGCA AACGCAGCGA CGGAAGGAGC ATACAGAAGT ATGTGACTGA 120

CGCTCGCAAG AGCAGCCAAC GCCGTATCAG TGTAAAAGAC ACAGGACGGA GCACAAAGAA 180

TTTGCCTGGC GGCACTAGCG CGGTGGTCCC ACCTGACCCC ATGCCGAACT CAGAAGTGAA 240

ACGCCGTAGC GCCGATGGTA GTGTGGGGTC TCCCCATGCG AGAGTAGGGA ACTGCCAGGC 300

ATCAAATTAG AAGTATGGCA TTAACTTTCG GGGCAGTTCA CTACTCGACA GAGAGTAGGA 360

CAAAAGCGAA AGCTTTTGAA CGTTGCGAAG CAACGGCCCG AAGGGTGAAT CACGAAGTGA 420

TTCATAAACT GCCAGGCATC AGATTAAGCA GTAAACTGAT GTAAAAATCA GTGGTTGTAA 480

AAGAATTCGG TGGAGCGG 498

>(142)C Schwarzengrund_NC_011094

ATGTTTTGGC GGACTGAGAG ACGATTTTCA GCACTGATTC CGGATTTTCG GGAACGAAAG 60

ATAAAAGATT CGTTTCCGGC AACAGAATTT GCCTGGCGGC ACTAGCGCGG TGGTCCCACC 120

TGACCCCATG CCGAACTCAG AAGTGAAACG CCGTAGCGCC GATGGTAGTG TGGGGTCTCC 180

CCATGCGAGA GTAGGGAACT GCCAGGCATC AAATTAAGCA GTAAGCCGGT CATAAACCGG 240

TGGTTGTAAA AGAATCCGGT GGAGCGG 267

>(143)C Stanley_LS483434

ATGTTTTGGC GGATTGAGAA GACAACAATT TTCAGCTCAG ATTCAGAGTC CGAAGGATTT 60

TACGCTGAGA CAAGGCGGCA AACGCAGCGA CGGAAGGAGT ATACAGAAGT ATGTGACTGA 120

CGCTCGCAAG AGCAGCCAAC GCCGTATCAG TGTAAAAGAC ACAGGACAGA GCACAAAGAA 180

TTTGCCTGGC GGCACTAGCG CGGTGGTCCC ACCTGACCCC ATGCCGAACT CAGAAGTGAA 240

ACGCCGTAGC GCCGATGGTA GTGTGGGGTC TCCCCATGCG AGAGTAGGGA ACTGCCAGGC 300

ATCAAATTAG AAGTATGGCA TTAACTTTCG GGGCAGTTCA CTACTCGACA GAGAGTAGGA 360

CAAAAGCGAA AGCTTTTGAA CGTTGCGAAG CAACGGCCCG AAGGGTGAAT CACGAAGTGA 420

TTCATAAACT GCCAGGCATC AAATTGCAGT AAACCGGGAT GAAAATCCGG GTTGTGACAA 480

AGAAATTCGG TGGAGCGG 498

>(144)C Thompson_NC_022525

ATGTTTTGGC GGATGAGAGA CGATTTTCAG CACTGATTCA GATTACATCA GAACGTAAAA 60

ACGGTCTGAT AACAGAATTT GCCTGGCGGC CGTAGCGCGG TGGTCCCACC TGACCCCATG 120

CCGAACTCAG AAGTGAAACG CCGTAGCGCC GATGGTAGTG TGGGGTCTCC CCATGCGAGA 180

GTAGGGAACT GCCAGGCATC AATTTAAGCA GTAAACTGAT GTAAAAATCA GTGATTGTAA 240

AAGAATTCGG TGGAGCGG 258

>(145)C Typhi_NC_003198

ATGTTTTGGC GGATGAGAGA CGATTTTCAG CACTGATTCC GGATTTTCGG GAACGAAAGA 60

TAAAAGATTC GTTTCCGGCA ACAGAATTTG CCTGGCGGCA CTAGCGCGGT GGTCCCACCT 120

GACCCCATGC CGAACTCAGA AGTGAAACGC CGTAGCGCCG ATGGTAGTGT GGGGTCTCCC 180

CATGCGAGAG TAGGGAACTG CCAGGCATCA AATTAAGCAG TAAATTGATG TAAAAATCAG 240

TGGTTGTAAA AGAATTCGGT GGAGCGG 267

>(146)C Typhimurium-DT2_NC_022544

ATGTTTTGGC GGTTAAGAGA AGATTTTCAG CACTGATTCA GAGTTGAGTA CGCAATAATT 60

TGCGCAGCAG CGAGGCGGCA AGCGAAGGAA AGGAAGGAGC ATACAGAAGT ATGTGACTGA 120

CTTTACGAGC GCAGGCAACG CCGCTGATGC GATAAAGAAT TGCGTACAGA GCACAAAGAA 180

TTTGCCTGGC GGCACTAGCG CGGTGGTCCC ACCTGACCCC ATGCCGAACT CAGAAGTGAA 240

ACGCCGTAGC GCCGATGGTA GTGTGGGGTC TCCCCATGCG AGAGTAGGGA ACTGCCAGGC 300

ATCAAATTAG AAGTATGGCA TTAACTTTCG GGGCAGTTCA CTACTCGACA GAGAGTAGGA 360

CAAAAGCGAA AGCTTTTGAA CGTTGCGAAG CAACGGCCCG AAGGGTGAAT CACGAAGTGA 420

TTCATAAACT GCCAGGCATC AAATTAAGCA GTAAACTGAT GTAAAAATCA GTGGTTGTAA 480

AAGAATTCGG TGGAGCGG 498

>(147)X Typhimurium_Enteritidis_Albert_Hissar_NC_003917_CP044188_CP018657_CP088138

GAAGAGATAA GTGCTGAAAG CATCTAAGCA CGAAACTTGC CCCGAGATGA GTTCTCCCTG 60

AGACTTAGAG TCTCCTGAAG GAACGTTGAA GACGACGACG TTGATAGGCC GGGTGTGTAA 120

GCGCAGCGAT GCGTTGAGCT AACCGGTACT AATGAACCGT GAGGCTTAAC CTTACAACGC 180

CGAAGATGTT TTGGCGGATT GAGAAGACAA CAATTTTCAG CTCAGATTCA GAGTCCGAAG 240

GATTTTACGC TGAGACAAGG CGGCAAACGC AGCGACGGAA GGAGCATACA GAAGTATGTG 300

ACTGACGCTC GCAAGAGCAG CCAACGCCGT ATCAGTGTAA AAGACACAGG ACGGAGCACA 360

AAGAATTTGC CTGGCGGCAC TAGCGCGGTG GTCCCACCTG ACCCCATGCC GAACTCAGAA 420

GTGAAACGCC GTAGCGCCGA TGGTAGTGTG GGGTCTCCCC ATGCGAGAGT AGGGAACTGC 480

CAGGCATCAA ATTAGAAGTA TGGCATTAAC TTTCGGGGCA GTTCACTACT CGACAGAGAG 540

TAGGACAAAA GCGAAAGCTT TTGAACGTTG CGAAGCAACG GCCCGAAGGG TGAATCACGA 600

AGTGATTCAT AAACTGCCAG GCATCAAATT AAGCAGTAAA CTGATGTAAA AATCAGTGGT 660

TGTAAAAGAA TTCGGTGGAG CGGTAGTTCA GTTGGTTAGA ATACCTGCCT GTCACGCAGG 720

GGGTCGCGGG TTCGAGTCCC GTCCGTTCCG CCACTTATTA AGAGCCCTGA GCATTAGCTC 780

AGGGCTTTTT CTTTCCATGT CATTTAATTA TTGCTCAATT CGCAAAAATC CTCTGCATTT 840

CTCGCTCTTT TTCTGCAAAG CGCTGAAGGT CATAATCCCC TCAGTTAACG AACATAGTGA 900

TTAAAAAGAG GAATTCTATG ACTATCCCTG CATTCGGTTT AGGTACTTTT CGCCTGAAAG 960

ACGACGTGGT TATCGCTTCT GTTAAAACGG CGCTTGAACT GGGCTACCGT GCTGTCGACA 1020

CTGCGCAAAT CTATTATAAT GAAGCGGCTG TTGGACAGGC GATTGCTGAG AGCGGCGTAC 1080

CGCGTAATGA GCTGTACATC ACCACCAAGA TCTGGATTGA AAACCTTAGC AAAGACAAGC 1140

TAATCCCAAG CCTGAAAGAG AGTCTGAAAA ACTGCGTACC GATAATGTCG ATCTGAC 1197

>(148)C Unique_NoNCBImatch

ATGTTTTGGC GGATGAGAGA CGATTTTCAG CAGTGATTCA GAGTTGAGTA CGCAATAATT 60

TGCGCAGCAG CAAGGCGGCA AGCGAAGGAA AGGAAGGAGC ATACAGAAGT ATGTGACTGA 120

GTTTACGAGC GCAGGCAACG CCGCTGATGC GATAAAGAAT TGCGTACAGA GCACAAAGAA 180

TTTGCCTGGC GGCAGTAGCG CGGTGGTCCC ACCTGACCCC ATGCCGAACT CAGAAGTGAA 240

ACGCCGTAGC GCCGATGGTA GTGTGGGGTC TCCCCATGCG AGAGTAGGGA ACTGCCAGGC 300

ATCAAATTGC AGTAAACCGG GATGAAAATC CGGGTTGTGA CAAAGAAATT CGGTGGAGCG 360

G 361

>(149)C Unique_NoNCBImatch

ATGTTTTGGC GGTTGAGAAG ACAACAATTT TCAGCTCAGA TTCAGAGTTG AGTACGCAAT 60

AATTTGCGCA GCAGCAAGGC GGCAAGCGAA GGAAAGGAAG GAGCATACAG AAGTATGTGA 120

CTGACTTTAC GAGCGCAGGC AACGCCGCTG ATGCGATAAA GAATTGCGTA CTGAGCATAA 180

AAGAATTTGC TTGGCGGCAC TAGCGCGGTG GTCCCACCTG ACCCCATGCC GAACTCAGAA 240

GTGAAACGCC GTAGCGCCGA TGGTAGTGTG GGGTCTCCCC ATGCGAGAGT AGGGAACTGC 300

CAGGCATCAA ATTAAGCAGT AAGCCGGTCA TAAACCGGTG GTTGTAAAAG AATCCGGTGG 360

AGCGG 365

>(150)C Unique_NoNCBImatch

ATGTTTTGGC GGATGAGAGA AAGATTTTCA GCCTGATACA GATTAAATCA GAACGCAGAA 60

GCGGTCTGAT AAAACAGAAT TTGCCTGGCG GCCTTAGCGC GGTGGTCCCA CCTGACCCCA 120

TGCCGAACTC AGAAGTGAAA CGCCGTAGCG CCGATGGTAG TGTGGGGTCT CCCCATGCGA 180

GAGTAGGGAA CTGCCAGGCA TCAAATTACG AAGAATGGCA CTGATCTTTG AGGCAGTTCA 240

CTACTCGATA AGAGAGTAGG ACAAAAGCGA AAGCTTTTGA ACGTTGCGAA GCAACGGCCC 300

GAAGGGTGAA TCACGAAGTG ATTCATAAAC TGCCAGGCAT CAAATTAAGC AGTAAGCCGG 360

TCATAAAACC GGTGGTTGTA AAAGAATTCG GTGGAGCGG 399

>(151)C Brandenburg_NoNCBImatch

ATGTTTTGGC GGATTGAGAG ACGATTTTCA GCACTGATTC CGGATTTTCG GGAACGAAAG 60

ATAAGAGATT CGTTTCCGGC AACAGAATTT GCCTGGCGGC ACTAGCGCGG TGGTCCCACC 120

TGACCCCATG CCGAACTCAG AAGTGAAACG CCGTAGCGCC GATGGTAGTG TGGGGTCTCC 180

CCATGCGAGA GTAGGGAACT GCCAGGCATC AAATTAGAAG TATGGCATTA ACTTTCGGGG 240

CAGTTCACTA CTCGACAGAG AGTAGGACAA AAGCGAAAGC TTTTGAACGT TGCGAAGCAA 300

CGGCCCGAAG GGTGAATCAC GAAGTGATTG ATAAACTGCC AGGCATCAAA TTAAGCAGTA 360

AGCCGGTCAT AAACTGGTGG TTGTAAAAAA ATTCGGTGGA GCGG 404

>(152)C Unique_NoNCBImatch

ATGCTTTGGC GGATTGAGAG ATGATTTTCA GCACTGATTC AGAGTTGAGT ACGCAATAAT 60

TTGCGCAGCA GCAAGGCGGC AAGCGAAGGA AAGGAAGGAG CATACAGAAG TATGTGACTG 120

AGTTTACGAG CGCAGGCAAC GCCGCTGATG CGATAAAGAA TTGCGTACTG AGCATAAAAG 180

AATTTGCCTG GCGGCACTAG CGCGGTGGTC CCACCTGACC CCATGCCGAA CTCAGAAGTG 240

AAACGCCGTA GCGCCGATGG TAGTGTGGGG TCTCCCCATG CGAGAGTAGG GAACTGCCAG 300

GCATCAAATT AGAAGTATGG CATTAACTTT CGGGGCAGTT CACTACTCGA CAGAGAGTAG 360

GACAAAAGCG AAAGCTTTTG AACGTTGCGA AGCAACGGCC CGAAGGGTGA ATCACGAAGT 420

GATTCATAAA CTGCCAGGCA TCAAATTAAG CAGTAAGCCG GTCATAGACT GGTGGTTGTA 480

AAAGAATTCG GTGGAGCGG 499

>(153)C Unique_NoNCBImatch

ATGTTTTGGC GGATTGAGAA GACAACAATT TTCAGCTCAG ATTCAGAGTC CGAAGGATTT 60

TACGCTGAGA CAAGGCGGCA AACGCAGCGA CGGAAGGAGC ATACAGAAGT ATGTGACTGA 120

CGCCCGCAAG AGCAGCCAAC GCGGTATCAG TGTAAAAGAC ACAGGACAGA GCACAAAGAA 180

TTTGCCTGGC GGCACTAGCG CGGTGGTCCC ACCTGACCCC ATGCCGAACT CAGAAGTGAA 240

ACGCCGTAGC GCCGATGGTA GTGTGGGGTC TCCCCATGCG AGAGTAGGGA ACTGCCAGGC 300

ATCAAATTAG AAGTATGGCA TTAACTTTCG GGGCAGTTCA CTACTCGACA GAGAGTAGGA 360

CAAAAGCGAA AGCTTTTGAA CGTTGCGAAG CAACGGCCCG AAGGGTGAAT CACGAAGTGA 420

TTCATAAACT GCCAGGCATC AAATTAAGCA GTAAACTGAT GTAAAAATCA GTGGTTGTAA 480

AAGAATTCGG TGGAGCGG 498

>(154)C Unique_NoNCBImatch

ATGTTTTGGG GGGCTGAGAA GGAGAGCAAT TTTCAGTTTT GATTCAGAGT TGAGTAGGCA 60

ATAATTTGTG CAGCAGCAAG GGGGCAAGCG AAGGAAAGGA AGGAGCATTC AGAAGTATGT 120

GAGTGGCTTT ACGAGCGCAG GCAACCCCGT TGATGTGATA AAGAATTGGG TGGTGAGCAT 180

AAAAGAATTT CCCGGGGGGC GCTAGCCCGG GGGTCCCCCC GGCCCCCATC CGGAATTCAG 240

AAGGGAAACC CGGTAGCCCC GAGGGTAGGG GGGGTTTTCC CCATGGGAGA GTAGGGAACT 300

CCCAGCCATC AAATTCAGCA GTAAGCCGGT CATATACCGG TGGTTGTAAA AGAATTCGGT 360

GGAGCGG 367

>(155)C Unique_NoNCBImatch

ATGTTTTGGC GGTTGAGAAG ACGATTTTCA GCACTGATTC AGAGTTGAGT ACGCAATAAT 60

TTGCGCAGCA GCAAGGCGGC AAGCGAAGGA AAGGAAGGAG CATACAGAAG TATGTGACTG 120

AGTTTACGAG CGCAGGCAAC GCCGCTGATG CGATAAAGAA TTGCGTACAG AGCATAAAAG 180

AATTTGCCTG GCGGCACTAG CGCGGTGGTC CCACCTGACC CCATGCCGAA CTCAGAAGTG 240

AAACGCCGTA GCGCCGATGG TAGTGTGGGG TCTCCCCATG CGAGAGTAGG GAACTGCCAG 300

GCATCAAATT AGAAGTATGG CATTAACTTT CGGGGCAGTT CACTACTCGA CAGAGAGTAG 360

GACAAAAGCG AAAGCTTTTG AACGTTGCGA AGCAACGGCC CGAAGGGGGA ATCCCGAAGT 420

GATTCATAAG CTGCCAGGCA TCAAATTGCA GTAAACCGGG ATGAAAATCC GGATTGTGAC 480

AAAGAAATTC GGTGGAGCGG 500

>(156)C Unique_NoNCBImatch

ATGTTTTGGC GGTTGAGAAG ACAACAATTT TCAGCACTGA TTCAGAGTTG AGTACGCAAT 60

AATTTGCGCA GCAGCAAGGC GGCAAGCGAA GGAAAGGAAG GAGCATACAG AAGTATGTGA 120

CTGACTTTGC GAGCGCAGGC AACGCCGCTG ATGCGATAAA GAATTGCGTA CTGAGCATAA 180

AAGAATTTGC CTGGCGGCAG TAGCGCGGTG GTCCCACCTG ACCCCATGCC GAACTCAGAA 240

GTGAAACGCC GTAGCGCCGA TGGTAGTGTG GGGTCTCCCC ATGCGAGAGT AGGGAACTGC 300

CAGGCATCAA ATTAAGCAGT AAGCCGGTCA TAAACCGGTG GTTGTAAAAG AATTCGGTGG 360

AGCGG 365

>(157)C Unique_NoNCBImatch

ATGTTTTGGC GGATTGAGAA GACAACAATT TTCAGCTCAG ATTCAGAGTC CGAAGGATTT 60

TACGCTGAGA CAAGGCGGCA AACGCAGCGA CGGGAGGAGC ATACAGAAGT ATGTGACTGA 120

CGCCCGCAAG AGCAGCCAAC GCCGTATCAG TGTAAAAGAC ACAGGACAGA GCACAAAGAA 180

TTTGCCTGGC GGCAGTAGCG CGGTGGTCCC ACCTGACCCC ATGCCGAACT CAGAAGTGAA 240

ACGCCGTAGC GCCGATGGTA GTGTGGGGTC TCCCCATGCG AGAGTAGGGA ACTGCCAGGC 300

ATCAAATTAG AAGTATGGCA TTAACTTTCG GGGCAGTTCA CTACTCGACA GAGAGTAGGA 360

CAAAAGCGAA AGCTTTTGAA CGTTGCGAAG CAACGGCCCG AAGGGTGAAT CACGAAGTGA 420

TTCATAAACT GCCAGGCATC AAATTGCAGT AAACCGGGAT GAAAATCCGG GTTGTGACAA 480

AGAAATTCGG TGGAGCGG 498

>(158)C Enteritidis_CP075019

ATGTTTTGGC GGATGAGAGA CGATTTTCAG CAGTGATTCA GAGTTGAGTA CGCAATACTT 60

TGCGCAGCAG CAAGGCGGCA AGCGAAGGAA AGGAAGGAGC ATACAGAAGT ATGTGACTGA 120

GTTTACGAGC GCAGGCAACG CCGCTGATGC GATAAAGAAT TGCGTACAGA GCACAAAGAA 180

TTTGCCTGGC GGCAGTAGCG CGGTGGTCCC ACCTGACCCC ATGCCGAACT CAGAAGTGAA 240

ACGCCGTAGC GCCGATGGTA GTGTGGGGTC TCCCCATGCG AGAGTAGGGA ACTGCCAGGC 300

ATCAAATTAG AAGTATGGCA TTAACTTTCG GGGCAGTTCA CTACTCGACA GAGAGTAGGA 360

CAAAAGCGAA AGCTTTTGAA CGTTGCGAAG CAACGGCCCG AAGGGTGAAT CACGAAGTGA 420

TTCATAAACT GCCAGGCATC AAATTGCAGT AAACCGGGAT GAAAATCCGG GTTGTGACAA 480

AGAAATTCGG TGGAGCGG 498

>(159)C Unique_NoNCBImatch

ATGTTTTGGC GGTTGAGAGA CGATTTTCAG CACTGATTCA GATTACATCA GAACGTAAAA 60

ACGGTCTGAT AACAGAATTT GCCTGGCGGC ACTAGCGCGG TGGTCCCACC TGACCCCATG 120

CCGAACTCAG AAGTGAAACG CCGTAGCGCC GATGGTAGTG TGGGGTCTCC CCATGCGAGA 180

GTAGGGAACT GCCAGGCATC AAATTAGAAG TATGGCATTA ACTTTCGGGG CAGTTCACTA 240

CTCGACAGAG AGTAGGACAA AAGCGAAAGC TTTTGAACAT TGCGAAGCAA CGGCCCGAAG 300

GGTGAATCAC GAAGTGATTC ATAAACTGCC AGGCATCAAA TTAAGCAGTA AACTGATGTA 360

AAAATCAGTG GTTGTAAAAG AATTCGGTGG AGCGG 395

>(160)C Unique_NoNCBImatch

ATGTTTTGGC GGATTGAGAA GACAACAATT TTCAGCTCAG ATTCAGAGTC CGAAGGATTT 60

TACGCTGAGA CAAGGCGGCA AACGCAGCGA CGGAAGGAGC ATACAGAAGT ATGTGACTGA 120

CGCTCGCAAG AGCAGCCAAC GCCGTATCAG TGTAAAAGAC ACAGGACGGA GCACAAAGAA 180

TTCGCCTGGC GGCACTAGCG CGGTGGTCCC ACCTGACCCC ATGCCGAACT CAGAAGTGAA 240

ACGCCGTAGC GCCGATGGTA GTGTGGGGTC TCCCCATGCG AGAGTAGGGA ACTGCCAGGC 300

ATCAAATTAG AAGTATGGCA TTAACTTTCG GGGCAGTTCA CTACTCGACA GAGAGTAGGA 360

CAAAAGCGAA AGCTTTTGAA CGTTGCGAAG CAACGGCCCG AAGGGTGAAT CACGAAGTGA 420

TTCATAAACT GCCAGGCATC AAATTAAGCA GTAAACTGAT GTAAAAATCA GTGGTTGTAA 480

AAGAATTCGG TGGAGCGG 498

>(161)C Enteritidis_CP009091

ATGTTTTGGC GGATGAGAAG ACGATTTTCA GCAGTGATTC AGAGTTGAGT ACGCAATACT 60

TTGCGCAGCA GCAAGGCGGC AAGCGAAGGA AAGGAAGGAG CATACAGAAG TATGTGACTG 120

AGTTTACGAG CGCAGGCAAC GCCGCTGATG CGATAAAGAA TTGCGTACAG AGCACAAAGA 180

ATTTGCCTGG CGGCAGTAGC GCGGTGGTCC CACCTGACCC CATGCCGAAC TCAGAAGTGA 240

AACGCCGTAG CGCCGATGGT AGTGTGGGGT CTCCCCATGC GAGAGTAGGG AACTGCCAGG 300

CATCAAATTA GAAGTATGGC ATTAACTTTC GGGGCAGTTC ACTACTCGAC AGAGAGTAGG 360

ACAAAAGCGA AAGCTTTTGA ACGTTGCGAA GCAACGGCCC GAAGGGTGAA TCACGAAGTG 420

ATTCATAAAC TGCCAGGCAT CAAATTGCAG TAAACCGGGA TGAAAATCCG GGTTGTGACA 480

AAGAAATTCG GTGGAGCGG 499

>(162)C Infantis_NoNCBImatch

ATGTTTTGGC GGATGAGAGA CGATTTTCAG CACTGATTCA GAGTTGAGTA CGCAATAATT 60

TGCGCAGCGG CAAGCGAAGG AAAGGAAGGA GCATACAGAA GTATGTGACT GAGTTTACGA 120

GCGCAGGCAA CGCCGCTGAT GCGATAAAGA ATTGCGTACA GAGCACAAAG AATTTGCCTG 180

GCGGCACTAG CGCGGTGGTC CCACCTGACC CCATGCCGAA CTCAGAAGTG AAACGCCGTA 240

GCGCCGATGG TAGTGTGGGG TCTCCCCATG CGAGAGTAGG GAACTGCCAG GCATCAAATT 300

GCAGTAAACC GGGATGAAAA TCCGGGTTGT GACAAAGAAA TTCGGTGGAG CGG 353

>(163)C Stanley_CP036167

ATGTTTTGGC GGTTGAGAGA AGATTTTCAG CACTGATTCA GAGTCCGAAG GATTTTACGC 60

TGAGACAAGG CGGCAAACGC AGCGACGGAA GGAGCATACA GAAGTATGTG ACTGACGCTC 120

GCAAGAGCAG CCAACGCCGT ATCAGTGTAA AAGACACAGG ACAGAGCACA AAGAATTTGC 180

CTGGCGGCAC TAGCGCGGTG GTCCCACCTG ACCCCATGCC GAACTCAGAA GTGAAACGCC 240

GTAGCGCCGA TGGTAGTGTG GGGTCTCCCC ATGCGAGAGT AGGGAACTGC CAGGCATCAA 300

ATTAGAAGTA TGGCATTAAC TTTCGGGGCA GTTCACTACT CGACAGAGAG TAGGACAAAA 360

GCGAAAGCTT TTGAACGTTG CGAAGCAACG GCCCGAAGGG TGAATCACGA AGTGATTCAT 420

AAACTGCCAG GCATCAATTT AAGCAGTAAA CTGATGTAAA AATCAGTGAT TGTAAAAGAA 480

TTCGGTGGAG CGG 493

>(164)C Give_NoNCBImatch

ATGTTTTGGC GGTTGAGAGA CGATTTTCAG CACTGATTCA GAGTTGAGTA CGCAATAATT 60

TGCGCAGCAG CAAGGCGGCA AGCGAAGGAA AGGAAGGAGC ATACAGAAGT ATGTGACTGA 120

CTTTACGAGC GCAGGCAACG CCGCTGATGC GATAAAGAAT TGCGTACAGA GCACAAAGAA 180

TTTGCCTGGC GGCACTAGCG CGGTGGTCCC ACCTGACCCC ATGCCGAACT CAGAAGTGAA 240

ACGCCGTAGC GCCGATGGTA GTGTGGGGTC TCCCCATGCG AGAGTAGGGA ACTGCCAGGC 300

ATCAAATTAA GCAGTAAGCC GGTCATAAAC CGGTGGTTGT AAAAGAATCC GGTGGAGCGG 360

>(165)C Hartford_CP074660

ATGTTTTGGC GGTTGAGAGA CGATTTTCAG CACTGATTCA GAGTTGAGTA CGCAATAATT 60

TGCGCAGCAG CAAGGCGGCA AGCGAAGGAA AGGAAGGAGC ATACAGAAGT ATGTGACTGA 120

GTTTACGAGC GCAGGCAACG CCGCTGATGC GATAAAGAAT TGCGTACAGA GCACAAAGAA 180

TTTGCCTGGC GGCACTAGCG CGGTGGTCCC ACCTGACCCC ATGCCGAACT CAGAAGTGAA 240

ACGCCGTAGC GCCGATGGTA GTGTGGGGTC TCCCCATGCG AGAGTAGGGA ACTGCCAGGC 300

ATCAAATTGC AGTAAACCGG GATGAAAATC CGGGTTGTGA CAAAGAAATT CGGTGGAGCG 360

G 361

>(166)C Ouakam_NoNCBImatch

ATGTTTTGGC GGATTGAGAA GACGATTTTC AGCACTGATT CAGAGTTGAG TACGCAATAA 60

TTTGCGCAGC AGCAAGGCGG CAAGCGAAGG AAAGGAAGGA GCATACAGAA GTATGTGACT 120

GACTTTACGA GCGCAGGCAA CGCCGCTGAT GCGATAAAGA ATTGCGTACT GAGCACAAAG 180

AATTTGCCTG GCGGCCGTAG CGCGGTGGTC CCACCTGACC CCATGCCGAA CTCAGAAGTG 240

AAACGCCGTA GCGCCGATGG TAGTGTGGGG TCTCCCCATG CGAGAGTAGG GAACTGCCAG 300

GCATCAAATT GCAGTAAACC GGGATGAAAA TCCGGGTGGT GACAAAGAAA TTCGGTGGAG 360

CGG 363

>(167)C Inverness_CP019181

CTGTTTTGGC GGATGAGAGA CGATTTTCAG CACTGATTCA GAGTTGAGTA CGCAATAATT 60

TGCGCAGCAG CAAGGCGGCA AGCGAAGAAA AGGAAGGAGC ATACAGAAGT ATGTGACTGA 120

GTTTACGAGC GCAGGCAACG CCGCTGATGC GATAAAGAAT TGCGTACAGA GCACAAAGAA 180

TTTGCCTGGC GGCACTAGCG CGGTGGTCCC ACCTGACCCC ATGCCGAACT CAGAAGTGAA 240

ACGCCGTAGC GCCGATGGTA GTGTGGGGTC TCCCCATGCG AGAGTAGGGA ACTGCCAGGC 300

ATCAAATTAG AAGTATGGCA TTAACTTTCG GGGCAGTTCA CTACTCGACA GAGAGTAGGA 360

CAAAAGCGAA AGCTTTTGAA CGTTGCGAAG CAACGGCCCG AAGGGTGAAT CACGAAGTGA 420

TTCATAAACT GCCAGGCATC AAATTAAGCA GTAAACTGAT GTAAAAATCA GTGGTTGTAA 480

AAGAATTCGG TGGAGCGG 498

>(168)C Reading_CP093132

ATGTTTTGGC GGATGAGAAG ACGAACAATT TTCAGCACTG ATTCCGGATT TTCGGGAACG 60

AAAGATAAGA GATTCGTTTC TGGCAACAGA ATTTGCCTGG CGGCACTAGC GCGGTGGTCC 120

CACCTGACCC CATGCCGAAC TCAGAAGTGA AACGCCGTAG CGCCGATGGT AGTGTGGGGT 180

CTCCCCATGC GAGAGTAGGG AACTGCCAGG CATCAAATTA GAAGTATGGC ATTAACTTTC 240

GGGGCAGTTC ACTACTCGAC AGAGAGTAGG ACAAAAGCGA AAGCTTTTGA ACGTTGCGAA 300

GCAACGGCCC GAAGGGTGAA TCACGAAGTG ATTGATAAAC TGCCAGGCAT CAAATTAAGC 360

AGTAAGCCGG TCATAAACTG GTGGTTGTAA AAAAATTCGG TGGAGCGG 408

>(169)X Ganimara_CP030288

CTTGCCCCGA GATGAGTTCT CCCTGAGACT TTAAGTCTCC TGAAGGAACG TTGAAGACGA 60

CGACGTTGAT AGGCCGGGTG TGTAAGCGCA GCGATGCGTT GAGCTAACCG GTACTAATGA 120

ACCGTGAGGC TTAACCTTAC AACGCCGAAG ATGTTTTGGC GGATTGAGAG AAGATTTTCA 180

GCTCAGATTC AGAGTCCGAA GGATTTTACG CTGAGACAAG GCGGCAAACG CAGCGACGGA 240

AGGAGCATAC AGAAGTATGT GACTGACGTT CGCAAGAGCA GCCAACGCCG TATCAGTGTA 300

AAAGACACAG GACAGAGCAC AAAGAATTTG CCTGGCGGCA CTAGCGCGGT GGTCCCACCT 360

GACCCCATGC CGAACTCAGA AGTGAAACGC CGTAGCGCCG ATGGTAGTGT GGGGTCTCCC 420

CATGCGAGAG TAGGGAACTG CCAGGCATCA AATTAGAAGT ATGGCATTAA CTTTCGGGGC 480

AGTTCACTAC TCGACAGAGA GTAGGACAAA AGCGAAAGCT TTTGAACGTT GCGAAGCAAC 540

GGCCCGAAGG GTGAATCACG AAGTGATTCA TAAACTGCCA GGCATCAAAT TAAGCAGTAA 600

GCCGGTCATA GACTGGTGGT TGTAAAAAAA TTCGGTGGAG CGGTAGTTCA GTTGGTTAGA 660

ATACCTGCCT GTCACGCAGG GGGTCGCGGG TTCGAGTCCC GTCCGTTCCG CCACTTATTA 720

AGAGCCCTGA GCATTAGCTC AGGGCTTTTT CTTTGCCTGT CATTTAATTA TTGCTCAATT 780

CGCAAAAATC CTCTGCATTT TTCGCTCTTT TTCTACAAAG CGCTGAAGGT CATAATCCCC 840

TCAGTTAACG AACATAGTGA TTAAAAAGAG GAATTCTATG ACTATCCCTG CATTCGGTTT 900

AGGTACTTTT CGCCTGAAAG ACGACGTGGT TATCGCTTCT GTGAAAACGG CGCTTGAACT 960

GGGCTACCGT GCTGTCGACA CTGCGCAAAT CTATGATAAT GAAGCGGCTG TTGGACAGGC 1020

GATTGCTGAG AGCGGCGTAC CGCGTAATGA GCTGTACATC ACCACCAAGA TCTGGATTGA 1080

AAACCTTAGC AAAGACAGCT GATCCCAGCC TGAAAGAGAG CTGAAAAACT GCGTACCGAT 1140

ATGTCGATCT GACGCTGATC ACTGGCCTCT CCGGTGATGC CGGT 1184

>(170)C Meleagridis_NoNCBImatch

ATGTTTTGGC GGATGAGAGA CGATTTTCAG CAGTGATTCA GAGTTGAGTA CGCAATAATT 60

TGCGCAGCAG CAAGGCGGCA AGCGAAGGAA AGGAAGGAGC ATACAGAAGT ATGTGACTGA 120

GTTTACGAGC GCAGGCAACG CCGCTGATGC GATAAAGAAT TGCGTACAGA GCACAAAGAA 180

TTTGCCTGGC GGCAGTAGCG CGGTGGTCCC ACCTGACCCC ATGCCGAACT CAGAAGTGAA 240

ACGCCGTAGC GCCGATGGTA GTGTGGGGTC TCCCCATGCG AGAGTAGGGA ACTGCCAGGC 300

ATCAAATTAG AAGTATGGCA TTAACTTTCG GGGCAGTTCA CTACTCGACA GAGAGTAGGA 360

CAAAAGCGAA AGCTTTTGAA CGTTGCGAAG CAACGGCCCG AAGGGTGAAT CACGAAGTGA 420

TTCATAAACT GCCAGGCATC AAATTGCAGT AAACCGGGAT GAAAATCCGG GTTGTGACAA 480

AGAAATTCGG TGGAGCGG 498

>(171)C Unique_NoNCBImatch

ATGTTTTGGC GGTTGAGAAG ACAACAATTT TCAGCTCAGA TTCAGAGTCC GAAGGATTTT 60

GCGCTGAGAC AAGGCGGCAA ACGCGGCGAC GGAAGGAGCA TACAGAAGTA TGTGACTGAC 120

GTTCGCAAGA GCAGCCAACG CCGTATCAGC GTAAAAGACA CAGGACAGAG CACAAAGAAT 180

TTGCCTGGCG GCACTAGCGC GGTGGTCCCA CCTGACCCCA TGCCGAACTC AGAAGTGAAA 240

CGCCGTAGCG CCGATGGTAG TGTGGGGTCT CCCCATGCGA GAGTAGGGAA CTGCCAGGCA 300

TCAAATTAGA AGTATGGCAT TAACTTTCGG GGCAGTTCAC TACTCGACAG AGAGTAGAAC 360

AAAAGCGAAA GCTTTTGAAC GTTGCGAAGC AACGGCCCGA AGGGTGAATC ACGAAGTGAT 420

TCATAAACTG CCAGGCATCA AATTGCAGTA AACCGGGATG AAAATCCGGG TTGTGACAAA 480

GAAATTCGGT GGAGCGG 497

>(172)C Unique_NoNCBImatch

ATGTTTTGGC GGTTGAGAGA CGATTTTCAG CACTGATTCC GGATTTTCGG GAACGAAAGA 60

TAAAAGATTC GTTTCCGGCA ACAGAATTTG CCTGGCGGCA CTAGCGCGGT GGTCCCACCT 120

GACCCCATGC CGAACTCAGA AGTGAAACGC CGTAGCGCCG ATGGTAGTGT GGGGTATCCC 180

CATGCGAGAG TAGGGAACTG CCAGGCATCA AATTAGAAGT ATGGCATTAA CTTTCGGGGC 240

AGTTCACTAC TCGACAGAGA GTAGGACAAA AGCGAAAGCT TTTGAACGTT GCGAAGCAAC 300

GGCCCGAAGG GTGAATCACG AAGTGATTCA TAAACTGCCA GGCATCAAAT TGCAGTAAAC 360

CGGGATGAAA ATCCGGGTTG TGACAAAGAA ATTCGGTGGA GCGG 404

>(173)C Dublin_CP032449

ATGTTTTGGC GGATGAGAAG ACGATTTTCA GCAGTGATTC AGAGTTGAGT ACGCAATACT 60

TTGCGCAGCA GCAAGGCGGC AAGCGAAGGA AAGGAAGGAG CATACAGAAG TATGTGACTG 120

AGTTTACGAG CGCAGGCAAC GCCGCTGATG CGATAAAGAA TTGCGTACAG AGCACAAAGA 180

ATTTGCCTGG CGGCACTAGC GCGGTGGTCC CACCTGACCC CATGCCGAAC TCAGAAGTGA 240

AACGCTGTAG CGCCGATGGT AGTGTGGGGT CTCCCCATGC GAGAGTAGGG AACTGCCAGG 300

CATCAAATTA GAAGTATGGC ATTAACTTTC GGGGCAGTTC ACTACTCGAC AGAGAGTAGG 360

ACAAAAGCGA AAGCTTTTGA ACGTTGCGAA GCAACGGCCC GAAGGGTGAA TCACGAAGTG 420

ATTCATAAAC TGCCAGGCAT CAAATTGCAG TAAACCGGGA TGAAAATCCG GGTTGTGACA 480

AAGAAATTCG GTGGAGCGG 499

>(174)X Muenchen_Newport_CP51389_CP016014

GAAGAGATAA GTGCTGAAAG CATCTAAGCA CGAAACTTGC CCCGAGATGA GTTCTCCCTG 60

AGACTTAGAG TCTCCTGAAG GAACGTTGAA GACGACGACG TTGATAGGCC GGGTGTGTAA 120

GCGCAGCGAT GCGTTGAGCT AACCGGTACT AATGAACCGT GAGGCTTAAC CTTACAACGC 180

CGAAGATGTT TTGGCGGATT GAGAAGACAA CAATTTTCAG CTCAGATTCA GAGTCCGAAG 240

GATTTTACGC TGAGACAAGG CGGCAAACGC AGCGACGGAA GGAGCATACA GAAGTATGTG 300

ACTGACGCTC GCAAGAGCAG CCAACGCCGT ATCAGTGTAA AAGACACAGG ACAGAGCACA 360

AAGAATTTGC CTGGCGGCAC TAGCGCGGTG GTCCCACCTG ACCCCATGCC GAACTCAGAA 420

GTGAAACGCC GTAGCGCCGA TGGTAGTGTG GGGTCTCCCC ATGCGAGAGT AGGGAACTGC 480

CAGGCATCAA ATTAGAAGTA TGGCATTAAC TTTCGGGGCA GTTCACTACT CGACAGAGAG 540

TAGGACAAAA GCGAAAGCTT TTGAACGTTG CGAAGCAACG GCCCGAAGGG TGAATCACGA 600

AGTGATTCAT AAACTGCCAG GCATCAAATT GCAGTAAACC GGGATGAAAA TCCGGGTTGT 660

GACAAAGAAA TTCGGTGGAG CGGTAGTTCA GTTGGTTAGA ATACCTGCCT GTCACGCAGG 720

GGGTCGCGGG TTCGAGTCCC GTCCGTTCCG CCACTTATTA AGAGCCCTGA GCATTAGCTC 780

AGGGCTTTTT CTTTGCCTGC GATTTAATTA TTGCTCAATT CGCAAAAATC CTCTGCATTT 840

CTCGCTCTTT TTCTACAAAG CGCTGAAGGT CATAATCCCC TCAGTTAACG AACATAGTGA 900

TTAGAAAGAG GAATTCTATG ACTATCCCTG CATTCGGTTT AGGTACTGTT CGCCTGAAAG 960

ACGACGTGGT TATCGCTTCT GTTAAAACGG CGCTTGAACT GGGCTACCGT GCTGTCGACA 1020

CTGCGCAAAT CTATGATAAT GAAGCGGCTG TTGGACAGGC GATTGCTGAG AGCGGTGTAC 1080

CGCGTAATGA GCTGAATATC ACCACCAAGA TCTGGATTGA AAACCTTAGC AAAGACAAGC 1140

TAATCCCAGG CTGAAAGAGA CCCTGATAAA CTGCGTACCG ATTATGTCGA TCTGACGCTG 1200

ATCACTGGCC ATCTCCGGTG ATGCGTGTTG TCAAACAATT ATGC 1244

>(175)C Enteritidis_CP018633

ATGTTTTGGC GGATGAGAAG ACGATTTTCA GCACTGATTC AGATTACATC AGAACGTAAA 60

AACGGTCTGA TAACAGAATT TGCCTGGCGG CACTAGCGCG GTGGTCCCAC CTGACCCCAT 120

GCCGAACTCA GAAGTGAAAC GCCGTAGCGC CGATGGTAGT GTGGGGTCTC CCCATGCGAG 180

AGTAGGGAAC TGCCAGGCAT CAAATTAGAA GTATGGCATT AACTTTCGGG GCAGTTCACT 240

ACTCGACAGA GAGTAGGACA AAAGCGAAAG CTTTTGAACG TTGCGAAGCA ACGGCCCGAA 300

GGGTGAATCA CGAAGTGATT CATAAACTGC CAGGCATCAA ATTGCAGTAA ACCGGGATGA 360

AAATCCGGGT TGTGACAAAG AAATTCGGTG GAGCGG 396

>(176)C Indiana_CP022450

ATGTTTTGGC GGATGAGAAG ACGATTTTCA GCACTGATTC AGATTACATC AGAACGTAAA 60

AACGGTCTGA TAACAGAATT TGCCTGGCGG CACTAGCGCG GTGGTCCCAC CTGACCCCAT 120

GCCGAACTCA GAAGTGAAAC GCCGTAGCGC CGATGGTAGT GTGGGGTCTC CCCATGCGAG 180

AGTAGGGAAC TGCCAGGCAT CAAATTAGAA GTATGGCATT AACTTTCGGG GCAGTTCACT 240

ACTCGACAGA GAGTAGGACA AAAGCGAAAG CTTTTGAACG TTGCGAAGCA ACGGCCCGAA 300

GGGTGAATCA CGAAGTGATT CATAAACTGC CAGGCATCAA ATTGCAGTAA ACCGGGATGA 360

AAATCCGGAT TGTGACAAAG AAATTCGGTG GAGCGG 396

>(177)C Derby_NoNCBImatch

ATGTTTTGGC GGTTGAGAAG ACAGATTTTC AGCTCAGATT CAGAGTTGAG TACGCAATAA 60

TTTGCGCAGC AGCAAGGCGG CAAGCGAAGG AAAGGAAGGA GCATACAGAA GTATGTGACT 120

GACTTTACGA GCGCAGGCAA CGCCGCTGAT GCGATAAAGA ATTGCGTACT GAGCATAAAA 180

GAATTTGCCT GGCGGCACTA GCGCGGTGGT CCCACCTGAC CCCATGCCGA ACTCAGAAGT 240

GAAACGCCGT AGCGCCGATG GTAGTGTGGG GTCTCCCCAT GCGAGAGTAG GGAACTGCCA 300

GGCATCAAAT TAAGCAGTAA GCCGGTCATA AACTGGTGGT TGTAAAAAAA TTCGGTGGAG 360

CGG 363

>(178)C Sendai_Saintpaul_NoNCBImatch

ATGTTTTGGC GGTTGAGAAG ACGATTTTCA GCACTGATTC CGGATTTTCG GGGACGAAAG 60

ATAAAAGATT CGTTTCCGGC AACAGAATTT GCCTGGCGGC ACTAGCGCGG TGGTCCCACC 120

TGACCCCATG CCGAACTCAG AAGTGAAACG CCGTAGCGCC GATGGTAGTG TGGGGTCTCC 180

CCATGCGAGA GTAGGGAACT GCCAGGCATC AAATTAGAAG TATGGCATTA ACTTTCGGGG 240

CAGTTCACTA CTCGACAGAG AGTAGGACAA AAGCGAAAGC TTTTGAACGT TGCGAAGCAA 300

CGGCCCGAAG GGTGAATCAC GAAGTGATTC ATAAACTGCC AGGCATCAAA TTGCAGTAAA 360

CCGGGATGAA AATCCGGGTT GTGACAAAGA AATTCGGTGG AGCGG 405

>(179)C Rubislaw_CP019192

ATGTTTTGGC GGTTGAGAGA CGATTTTCAG CACTGATTCC GGATTTTCGG GAACGAAAGA 60

TAAGAGATTC GTTTCCGGCA ACAGAATTTG CCTGGCGGCA CTAGCGCGGT GGTCCCACCT 120

GACCCCATGC CGAACTCAGA AGTGAAACGC CGTAGCGCCG ATGGTAGTGT GGGGTCTCCC 180

CATGCGAGAG TAGGGAACTG CCAGGCATCA AATTAGAAGT ATGGCATTAA CTTTCGGGGC 240

AGTTCACTAC TCGACAGAGA GTAGGACAAA AGCGAAAGCT TTTGAACGTT GCGAAGCAAC 300

GGCCCGAAGG GTGAATCACG AAGTGATTCA TAAACTGCCA GGCATCAAAT TAAGCAGTAA 360

GCCGGTCATA AACCGGTGGT TGTAAAAGAA TTCGGTGGAG CGG 403

>(180)C Reading_NoNCBImatch

ATGTTTTGGC GGTTGAGAAG ACAACAATTT TCAGCTCAGA TTCAGAGTCC GAAGGATTTT 60

ACGCTGAGAC AAGGCGGCAA ACGCAGCGAC GGAAGGAGCA TACAGAAGTA TGTGACTGAC 120

GTTCGCAAGA GCAGCCAACG CCGTATCAGT GTAAAAGACA CAGGACAGAG CACAAAGAAT 180

TTGCCTGGCG GCACTAGCGC GGTGGTCCCA CCTGACCCCA TGCCGAACTC AGAAGTGAAA 240

CGCCGTAGCG CCGATGGTAG TGTGGGGTCT CCCCATGCGA GAGTAGGGAA CTGCCAGGCA 300

TCAAATTAGA AGTATGGCAT TAACTTTCGG GGCAGTTCAC TACTCGACAG AGAGTAGGAC 360

AAAAGCGAAA GCTTTTGAAC GTTGCGAAGC AACGGCCCGA AGGGTGAATC ACGAAGTGAT 420

TCATAAACTG CCAGGCATCA AATTGCAGTA AACCGGGATG AAAATCCGGG TTGTGACAAA 480

GAAATTCGGT GGAGCGG 497

>(181)C Saintpaul_CP053055

ATGTTTTGGC GGATTGAGAG AAGATTTTCA GCACTGATTC CGGATTTTCG GGAACGAAAG 60

ATTTTACGCT GAGGCAAGGC GGCAAATGAA GGAAAGGAAG GAGCATACAG AAGTATGTGA 120

CTGACTTTAC GAATGCAGCC AACGCAGCAT CGGTGTAAAA GATTCGTTTC CGGCAACAGA 180

ATTTGCCTGG CGGCCGTAGC GCGGTGGTCC CACCTGACCC CATGCCGAAC TCAGAAGTGA 240

AACGCCGTAG CGCCGATGGT AGTGTGGGGT CTCCCCATGC GAGAGTAGGG AACTGCCAGG 300

CATCAAATTA GAAGTATGGC ATTAACTTTC GGGGCAGTTC ACTACTCGAC AGAGAGTAGG 360

ACAAAAGCGA AAGCTTTTGA ACGTTGCGAA GCAACGGCCC GAAGGGTGAA TCACGAAGTG 420

ATTCATAAAC TGCCAGGCAT CAAATTAAGC AGTAAACTGA TGTAAAAATC AGTGGTTGTA 480

AAAGAATTCG GTGGAGCGG 499

>(182)C Unique_NoNCBImatch

CTGTTTTGGC GGATGAGAGA AGATTTTCAG CCTGATACAG ATTAAATCAG AACGCAGAAG 60

CGGTCTGATA AAACAGAATT TGCCTGGCGG CCGTAGCGCG GTGGTCCCAC CTGACCCCAT 120

GCCGAACTCA GAAGTGAAAC GCCGTAGCGC CGATGGTAGT GTGGGGTATC CCCATGCGAG 180

AGTAGGGAAC TGCCAGGCAT CAAATTAAGC AGTAAGCCGG TCATAAAACC GGTGGTTGTA 240

AAAGAATTCG GTGGAGCGG 259

>(183)C Miami_NoNCBImatch

ATGTTTTGGC GGTTGAGAGA TGATTTTCAG CACTGATTCA GATTACATCA GAACGTAAAA 60

ACGGTCTGAT AACAGAATTT GCCTGGCGGC ACTAGCGCGG TGGTCCCACC TGACCCCATG 120

CCGAACTCAG AAGTGAAACG CCGTAGCGCC GATGGTAGTG TGGGGTCTCC CCATGCGAGA 180

GTAGGGAACT GCCAGGCATC AATTTAAGCA GTAAACTGAT GTAAAAATCA GTGGTTGTAA 240

AAGAATTCGG TGGAGCGG 258

>(184)X Saintpaul_Stanleyville_CP017727_CP034716

GAAGAGATAA GTGCTGAAAG CATCTAAGCA CGAAACTTGC CCCGAGATGA GTTCTCCCTG 60

AGACTTTAAG TCTCCTGAAG GAACGTTGAA GACGACGACG TTGATAGGCC GGGTGTGTAA 120

GCGCAGCGAT GCGTTGAGCT AACCGGTACT AATGAACCGT GAGGCTTAAC CTTACAACGC 180

CGAAGATGTT TTGGCGGATG AGAGACGATT TTCAGCACTG ATTCCGGATT TTCGGGAACG 240

AAAGATTTTA CGCTGAGGCA AGGCGGCAAA TGAAGGAAAG GAAGGAGCAT ACAGAAGTAT 300

GTGACTGACT TTGCGAATGC AGCCAACGCA GCATCGGTGT AAAAGATTCG TTTCCGGTAA 360

CAGAATTTGC CTGGCGGCAC TAGCGCGGTG GTCCCACCTG ACCCCATGCC GAACTCAGAA 420

GTGAAACGCC GTAGCGCCGA TGGTAGTGTG GGGTCTCCCC ATGCGAGAGT AGGGAACTGC 480

CAGGCATCAA ATTAAGCAGT AAACTGATGT AAAAATCAGT GGTTGTAAAA GAATTCGGTG 540

GAGCGGTAGT TCAGTTGGTT AGAATACCTG CCTGTCACGC AGGGGGTCGC GGGTTCGAGT 600

CCCGTCCGTT CCGCCACTTA TTAAGAGCCC TGAGCATTAG CTCAGGGCTT TTTCTTTGCC 660

TGTCATTTAA TTATTGCTCA ATTCGCAAAA ATCCTCTGCA TTTCTCGCTC TTTTTCTGCA 720

AAGCGCTGAA GGTCATAATC CCCTCAGTTA ACGAACATAG TGATTAAAAA GAGGAATTCT 780

ATGACTATCC CTGCATTCGG TTTAGGTACT TTTCGCCTGA AAGACGACGT GGTTATCGCT 840

TCTGTGAAAA CGGCGCTTGA ACTGGGCTAC CGTGCTGTCG ACACTGCGCA AATCTATGAT 900

AATGAAGCGG CTGTTGGACA GGCGATTGCT GAGAGCGGCG TACCGCGTAA TGAGCTGTAC 960

ATCACCACCA AGATCTGGAT TGAAAACCTT AGCAAAGACA AACTAATCCC AAGCCTGCAA 1020

GAGAGCCTGA AAAAACTGCG TACCGATTAT GTCGATCTGA CGCTGATCCA CTGGCCATCT 1080

CCGGGTGATG CCGTGTCTGT CGAAGAATTT ATGCAGGCAT TGCTGGAAGC TAAAAAACAA 1140

GGGCTGACTC GCGAAATTGG CATCTCAAAC TTCACATTCC GTTGATGGAA AAGGTATTGC 1200

CGCGTGGGCG CAGACATATC GCACCAACCA GATGAACTA 1239

>(185)C Paratyphi-A_CP009559

ATGTTTTGGC GGATGAGAGA CGATTTTCAG CACTGATTCC GGATTTTCGG GAACGAAAGA 60

TTTTACGCTG AGGCAAGGCG GCAAATGAAG GAAAGGAAGA AGCATACAGA AGTATGTGAC 120

TGACTTTACG AATGCAGCCA ACGCAGCATC GGTGTAAAAG ATTCGTTTCT GGCAACAGAA 180

TTTGCCTGGC GGCAGTAGCG CGGTGGTCCC ACCTGACCCC ATGCCGAACT CAGAAGTGAA 240

ACGCCGTAGC GCCGATGGTA GTGTGGGGTC TCCCCATGCG AGAGTAGGGA ACTGCCAGGC 300

ATCAAATTAG AAGTATGGCA TTAACTTTCG GGGCAGTTCA CTACTCGACA GAGAGTAGGA 360

CAAAAGCGAA AGCTTTTGAA CGTTGCGAAG CAACGGCCCG AAGGGTGAAT CACGAAGTGA 420

TTCATAAACT GCCAGGCATC AAATTAAGCA GTAAACTGAT GTAAAAATCA GTGGTTGTAA 480

AAGAATTCGG TGGAGCGG 498

>(186)C Nottingham_NoNCBImatch

ATGTTTTGGC GGACTGAGAA GACGAACAAT TTTCAGCTTT GATTCAGAGT TGAGTACGCA 60

ATAATTTGCG CAGCAGCAAG GCGGCAAGCG AAGGAAAGGA AGGAGCATAC AGAAGTATGT 120

GACTGAGTTT ACGAGCGCAG GCAACGCCGC TGATGCGATA AAGAATTGCG TACTGAGCAC 180

AAAAGAATTT GCCTGGCGGC CGTAGCGCGG TGGTCCCACC TGACCCCATG CCGAACTCAG 240

AAGTGAAACG CCGTAGCGCC GATGGTAGTG TGGGGTCTCC CCATGCGAGA GTAGGGAACT 300

GCCAGGCATC AAATTAGAAG TATGGCATTA ACTTTCTGGG CAGTTCACTA CTCGACAGAG 360

AGTAGGACAA AAGCGAAAGC TTTTGAACGT TGCGAAGCAA CGGCCCGAAG GGTGAATCAC 420

GAAGTGATTC ATAAACTGCC AGGCATCAAA TTGCAGTAAA CCGGGATGAA AATCCGGGTT 480

GTGACAAAGA AATTCGGTGG AGCGG 505

>(187)C Typhimurium_NC_021814

ATGTTTTGGC GGTTAAGAGA AGATTTTCAG CACTGATTCC GGATTTTCGG GAACGAAAGA 60

TTTTACGCTG AGGCAAGGCG GCAAGCGAAG GAAAGGAAGG AGCATACAGA AGTATGTGAC 120

TGACTTTACG AGCGCAGGCA ACGCCGCTGA TGCGATAAAG AATTGCGTAC AGAGCACAAA 180

GAATTTGCCT GGCGGCACTA GCGCGGTGGT CCCACCTGAC CCCATGCCGA ACTCAGAAGT 240

GAAACGCCGT AGCGCCGATG GTAGTGTGGG GTCTCCCCAT GCGAGAGTAG GGAACTGCCA 300

GGCATCAAAT TAGAAGTATG GCATTAACTT TCGGGGCAGT TCACTACTCG ACAGAGAGTA 360

GGACAAAAGC GAAAGCTTTT GAACGTTGCG AAGCAACGGC CCGAAGGGTG AATCACGAAG 420

TGATTCATAA ACTGCCAGGC ATCAAATTAA GCAGTAAACT GATGTAAAAA TCAGTGGTTG 480

TAAAAGAATT CGGTGGAGCG G 501

>(188)C Typhimurium_NC_016860

ATGTTTTGGC GGATTGAGAA GACAACAATT TTCAGCTCAG ATTCAGAGTC CGAAGGATTT 60

TACGCTGAGA CAAGGCGGCA AACGCAGCGA CGGAAGGAGC ATACAGAAGT ATGTGACTGA 120

CGCTCGCAAG AGCAGCCAAC GCCGTATCAG TGTAAAAGAC ACAGGACGGA GCACAAAGAA 180

TTTGCCTGGC GGCACTAGCG CGGTGGTCCC ACCTGACCCC ATGCCGAACT CAGAAGTGAA 240

ACGCCGTAGC GCCGATGGTA GTGTGGGGTC TCCCCATGCG AGAGTAGGGA ACTGCCAGGC 300

ATCAAATTAG AAGTATGGCA TTAACTTTCG GGGCAGTTCA CTACTCGACA GAGAGTAGGA 360

CAAAAGCGAA AGCTTTTGAA CGTTGCGAAG CAACGGCCCG AAGGGTGAAT CACGAAGTGA 420

TTCATAAACT GCCAGGCATC AAATTAAGCA GCAAACTGAT GTAAAAATCA GTGGTTGTAA 480

AAGAATTCGG TGGAGCGG 498

>(189)C Typhimurium_NoNCBImatch

ATGTTTTGGC GGATTGAGAA GACAACAATT TTCAGCTCAG ATTCAGAGTC CGAAGGATTT 60

TACGCTGAGA CAAGGCGGCA AACGCAGCGA CGGAAGGAGC ATACAGAAGT ATGTGACTGA 120

CGCTCGCAAG AGCAGCCAAC GCCGTATCAG TGTAAAAGAC ACAGAACGGA GCACAAAGAA 180

TTTGCCTGGC GGCCGTAGCG CGGTGGTCCC ACCTGACCCC ATGCCGAACT CAGAAGTGAA 240

ACGCCGTAGC GCCGATGGTA GTGTGGGGTC TCCCCATGCG AGAGTAGGGA ACTGCCAGGC 300

ATCAAATTAG AAGTATGGCA TTAACTTTCG GGGCAGTTCA CTACTCGACA GAGAGTAGGA 360

CAAAAGCGAA AGCTTTTGAA CGTTGCGAAG CAACGGCCCG AAGGGTGAAT CACGAAGTGA 420

TTCATAAACT GCCAGGCATC AAATTAAGCA GTAAACTGAT GTAAAAATCA GTGGTTGTAA 480

AAGAATTCGG TGGAGCGG 498

>(190)C Gaminara_NoNCBImatch

ATGTTTTGGC GGATTGAGAG AAGATTTTCA GCTCAGATTC AGAGTCCGAA GGATTTTACG 60

CTGAGACAAG GCGGCAAACG CAGCGACGGA AGGAGCATAC AGAAGTATGT GACTGACGTT 120

CGCAAGAGCA GCCAACGCCG TATCAGTGTA AAAGACACAG GACAGAGCAC AAAGAATTTG 180

CCTGGCGGCA CTAGCGCGGT GGTTCCACCT GACCCCATGC CGAACTCAGA AGTGAAACGC 240

CGTAGCGCCG ATGGTAGTGT GGGGTCTCCC CATGCGAGAG TAGGGAACTG CCAGGCATCA 300

AATTAGAAGT ATGGCATTAA CTTTCGGGGC AGTTCACTAC TCGACAGAGA GTAGGACAAA 360

AGCGAAAGCT TTTGAACGTT GCGAAGCAAC GGCCCGAAGG GTGAATCACG AAGTGATTCA 420

TAAACTGCCA GGCATCAAAT TAAGCAGTAA GCCGGTCATA GACTGGTGGT TGTAAAAAAA 480

TTCGGTGGAG CGG 493

>(191)C Schwarzengrund_NoNCBImatch

ATGTTTTGGC GGTTGAGAGA TGATTTTCAG CACTGATTCC GGATTTTCGG GAACGAAAGA 60

TAAAAGATTC GTTTCCGGCA ACAGAATTTG CCTGGCGGCA CTAGCGCGGT GGTCCCACCT 120

GACCCCATGC CGAACTCAGA AGTGAAACGC CGTAGCGCCG ATGGTAGTGT GGGGTCTCCC 180

CATGCGAGAG TAGGGAACTG CCAGGCATCA AATTAAGCAG TAAGCCGGTC ATAAACCGGT 240

GGTTGTAAAA GAATCCGGTG GAGCGG 266

>(192)C Enteritidis_CP009083

ATGTTTTGGC GGATGAGAAG ACGATTTTCA GCAGTGATTC AGAGTTGAGT ACGCAATACT 60

TTGCGCAGCA GCAAGGCGGC AAGCGAAGGA AAGGAAGGAG CATACAGAAG TATGTGACTG 120

AGTTTACGAG CGCAGGCAAC GCCGCTGATG CGATAAAGAA TTGCGTACAG AGCACAAAGA 180

ATTTGCCTGG CGGCACTAGC GCGGTGGTCC CACCTGACCC CATGCCGAAC TCAGAAGTGA 240

AACGCCGTAG CGCCGATGGT AGTGTGGGGT CTCCCCATGC GAGAATGTTG AATAGCAGTT 300

TGGTTCGCAG GCGAACGCCT ATTTACATAG CGCCGTTCAC GGTAGGGAAC TGCCAGGCAT 360

CAAATTAGAA GTATGGCATT AACTTTCGGG GCAGTTCACT ACTCGACAGA GAGTAGGACA 420

AAAGCGAAAG CTTTTGAACG TTGCGAAGCA ACGGCCCGAA GGGTGAATCA CGAAGTGATT 480

CATAAACTGC CAGGCATCAA ATTGCAGTAA ACCGGGATGA AAATCCGGGT TGTGACAAAG 540

AAATTCGGTG GAGCGG 556

>(193)C Kentucky_CP082602

ATGTTTTGGC GTTGAGAGAC GATTTTCAGC ACTGATTCAG ATTACATCAG AACGTAAAAA 60

CGGTCTGATA ACAGAATTTG CCTGGCGGCA CTAGCGCGGT GGTCCCACCT GACCCCATGC 120

CGAACTCAGA AGTGAAACGC CGTAGCGCCG ATGGTAGTGT GGGGTCTCCC CATGCGAGAG 180

TAGGGAACTG CCAGGCATCA AATTAGAAGT ATGGCATTAA CTTTCGGGGC AGTTCACTAC 240

TCGACAGAGA GTAGGACAAA AGCGAAAGCT TTTGAACGTT GCGAAGCAAC GGCCCGAAGG 300

GTGAATCACG AAGTGATTCA TAAACTGCCA GGCATCAAAT TGCAGTAAAC CGGGATGAAA 360

ATCCGGATTG TGACAAAGAA ATTCGGTGGA GCGG 394

>(194)C Mbandaka_NoNCBImatch

ATGTTTTGGC GGTGAGAGAC AATTTTCAGC AGTGATTCAG AGTTGAGTAC GCAATAATTT 60

GCGCAGCAGC AAGGCGGCAA GCGAAGGAAA GGAAGGAGCA TACAGAAGTA TGTGACTGAC 120

TTTACGAGCG CAGGCAACGC CGCTGATGCG ATAAAGAATT GCGTACAGAG CACAAAAGAA 180

TTTGCCTGGC GGCCGTAGCG CGGTGGTCCC ACCTGACCCC ATGCCGAACT CAGAAGTGAA 240

ACGCCGTAGC GCCGATGGTA GTGTGGGGTC TCCCCATGCG AGAGTAGGGA ACTGCCAGGC 300

ATCAAATTAG AAGTATGGCA TTAACTTTCG GGGCAGTTCA CTACTCGACA GAGAGTAGAA 360

CAAAAGCGAA AGCTTTTGAA CGTTGCGAAG CAACGGCCCG AAGGGTGAAT CACGAAGTGA 420

TTCATAAACT GCCAGGCATC AAATTGCAGT AAACCGGGAT GAAAATCCGG GTTGTGACAA 480

AGAAATTCGG TGGAGCGG 498

>(195)C Falkensee_NoNCBImatch

ATGTTTTGGC GGATGAGAGA CGATTTTCAG CACTGATTCC GGATTTTCGG GAACGAAAGA 60

TTTTACGCTG AGGCAAGGCG GCAAATGAAG GAAAGGAAGG AGCATACAGA AGTATGTGAC 120

TGACTTTGCG AATGCAGCCA ACGCAGCATC GGTGTAAAAG ATTCGTTTCC GGTAACAGAA 180

TTTGCCTGGC GGCACTAGCG CGGTGGTCCC ACCTGACCCC ATGCCGAACT CAGAAGTGAA 240

ACGCCGTAGC GCCGATGGTA GTGTGGGGTC TCCCCATGCG AGAGTAGGGA ACTGCCAGGC 300

ATCAAATTAG AAGTATGGCA TTAACTTTCG GGGCAGTTCA CTACTCGACA GAGAGTAGGA 360

CAAAAGCGAA AGCTTTTGAA CGTTGCGAAG CAACGGCCAG AAGGGTGAAT CACGAAGTGA 420

TTCATAAACT GCCAGGCATC AAATTGCAGT AAACTGATGT AAAAATCAGT GGTTGTAAAA 480

GAATTCGGTG GAGCGG 496

>(196)C Typhimurium_CP082526

ATGTTTTGGC GGTTGAGAGA CGATTTTCAG CTCAGATTCA GAGTCCGAAG GATTTTACGC 60

TGAGACAAGG CGGCAAACGC AGCGACGGAA GGAGCATACA GAAGTATGTG ACTGACGCTC 120

GCAAGAGCAG CCAACGCCGT ATCAGTGTAA AAGACACAGG ACGGAGCACA AAGAATTTGC 180

CTGGCGGCAC TAGCGCGGTG GTCCCACCTG ACCCCATGCC GAACTCAGAA GTGAAACGCC 240

GTAGCGCCGA TGGTAGTGTG GGGTCTCCCC ATGCGAGAGT AGGGAACTGC CAGGCATCAA 300

ATTAGAAGTA TGGCATTAAC TTTCGGGGCA GTTCACTACT CGACAGAGAG TAGGACAAAA 360

GCGAAAGCTT TTGAACGTTG CGAAGCAACG GCCCGAAGGG TGAATCACGA AGTGATTCAT 420

AAACTGCCAG GCATCAAATT AAGCAGTAAA CTGATGTAAA AATCAGTGGT TGTAAAAGAA 480

TTCGGTGGAG CGG 493

>(197)C Heidelberg_CP012921

ATGTTTTGGC GGATTGAGAA GACAACAATT TTCAGCTCAG ATTCAGAGTC CGAAGGATTT 60

TACGCTGAGA CAAGGCGGCA AACGCAGCGA CGGAAGGAGC ATACAGAAGT ATGTGACTGA 120

CGCCCGCAAG AGCAGTCAAC GCCGTATCAG TGTAAAAGAC ACAGGACAGA GCACAAAGAA 180

TTTGCCTGGC GGCAGTAGCG CGGTGGTCCC ACCTGACCCC ATGCCGAACT CAGAAGTGAA 240

ACGCCGTAGC GCCGATGGTA GTGTGGGGTC TCCCCATGCG AGAGTAGGGA ACTGCCAGGC 300

ATCAAATTAG AAGTATGGCA TTAACTTTCG GGGCAGTTCA CTACTCGACA GAGAGTAGGA 360

CAAAAGCGAA AGCTTTTGAA CGTTGCGAAG CAACGGCCCG AAGGGTGAAT CACGAAGTGA 420

TTCATAAACT GCCAGGCATC AAATTGCAGT AAACCGGGAT GAAAATCCGG GTTGTGACAA 480

AGAAATTCGG TGGAGCGG 498

>(198)C Infantis LS483479

ATGTTTTGGC GGATGAGAAG ACGATTTTCA GCACTGATTC AGAGTTGAGT ACGCAATAAT 60

TTGCGCAGCA GCAAGGCGGC AAGCGAAGGA AAGGAAGGAG CATACAGAAG TATGTGACTG 120

AGTTTACGAG CGCAGGCAAC GCCGCTGATG CGATAAAGAA TTGCGTACAG AGCACAAAGA 180

ATTTGCCTGG CGGCAGTAGC GCGGTGGTCC CACCTGACCC CATGCCGAAC TCAGAAGTGA 240

AACGCCGTAG CGCCGATGGT AGTGTGGGGT CTCCCCATGC GAGAGTAGGG AACTGCCAGG 300

CATCAAATTA GAAGTATGGC ATTAACTTTC GGGGCAGTTC ACTACTCGAC AGAGAGTAGG 360

ACAAAAGCGA AAGCTTTTGA ACGTTGCGAA GCAACGGCCC GAAGGGTGAA TCACGAAGTG 420

ATTAATAAAC TGCCAGGCAT CAAATTGCAG TAAACCGGGA TGAAAATCCG GGTTGTGACA 480

AAGAAATTCG GTGGAGCGG 499

>(199)C Newport_Abaetetuba_CP016357_CP074211

CCGCTCCACC GAATTCTTTT ACAACCACTG ATTTTTACAT CAGTTTACTG CTTAATTTGA 60

TGCCTGGCAG TTTATGAATC ACTTCGTGAT TCACCCTTCG GGCCGTTGCT TCGCAACGTT 120

CAAAAGCTTT CGCTTTTGTC CTACTCTCTG TCGAGTAGTG AACTGCCCCG AAAGTTAATG 180

CCATACTTCT AATTTGATGC CTGGCAGTTC CCTACTCTCG CATGGGGAGA CCCCACACTA 240

CCATCGGCGC TACGGCGTTT CACTTCTGAG TTCGGCATGG GGTCAGGTGG GACCACCGCG 300

CTACTGCCGC CAGGCAAATT CTGTTACCGG AAACGAATCT TTTACACCGA TGCTGCGTTG 360

GCTGCATTCG CAAAGTCAGT CACATACTTC TGTATGCTCC TTCCTTTCCT TCATTTGCCG 420

CCTTGCCTCA GCGTAAAATC TTTCGTTCCC GAAAATCCGG AATCAGTGCT GAAAATCGTC 480

TCTCATCCGC CAAAACAT 498

>(200)C Moscow_CP019415

ATGTTTTGGC GGATGAGAAG ACGATTTTCA GCAGTGATTC AGAGTTGAGT ACGCAATAAT 60

TTGCGCAGCA GCAAGGCGGC AAGCGAAGGA AAGGAAGGAG CATACAGAAG TATGTGACTG 120

AGTTTACGAG CGCAGGCAAC GCCGCTGATG CGATAAAGAA TTGCGTACAG AGCATAAAAG 180

AATTTGCCTG GCGGCACTAG CGCGGTGGTC CCACCTGACC CCATGCCGAA CTCAGAAGTG 240

AAACGCCGTA GCGCCGATGG TAGTGTGGGG TCTCCCCATG CGAGAGTAGG GAACTGCCAG 300

GCATCAAATT AGAAGTATGG CATTAACTTT CGGGGCAGTT CACTACTCGA CAGAGAGTAG 360

GACAAAAGCG AAAGCTTTTG AACGTTGCGA AGCAACGGCC CGAAGGGTGA ATCACGAAGT 420

GATTCATAAA CTGCCAGGCA TCAAATTGCA GTAAACCGGG ATGAAAATCC GGGTTGTGAC 480

AAAGAAATTC GGTGGAGCGG 500

>(201)C Typhimurium_CP011428

ATGTTTTGGC GGATGAGAAG ACGATTTTCA GCAGTGATTC CGGATTTTCG GGAACGAAAG 60

ATTTTACGCT GAGGCAAGGC GGCAAATGAA GGAAAGGAAG GAGCATACAG AAGTATGTGA 120

CTGACTTTAC GAATGCAGCC AACGCAGCAT CGGTGTAAAA GATTCGTTTC TGGCAACAGA 180

ATTTGCCTGG CGGCACTAGC GCGGTGGTCC CACCTGACCC CATGCCGAAC TCAGAAGTGA 240

AACGCCGTAG CGCCGATGGT AGTGTGGGGT CTCCCCATGC GAGAGTAGGG AACTGCCAGG 300

CATCAAATTA GAAGTATGGC ATTAACTTTC GGGGCAGTTC ACTACTCGAC AGAGAGTAGG 360

ACAAAAGCGA AAGCTTTTGA ACGTTGCGAA GCAACGGCCC GAAGGGTGAA TCACGAAGTG 420

ATTCATAAAC TGCCAGGCAT CAAATTAAGC AGTAAACTGA TGTAAAAATC AGTGGTTGTA 480

AAAGAATTCG GTGGAGCGG 499

>(202)C Blegdam_CP019406

ATGTTTTGGC GGATGAGAAG ACGATTTTCA GCAGTGATTC AGAGTTGAGT ACGCAATACT 60

TTGCGCAGCA GCAAGGCGGC AAGCGAAGGA AAGGAAGGAG CATACAGAAG TATGTGACTG 120

AGTTTACGAG CGCAGGCAAC GCCGCTGATG CGATAAAGAA TTGCGTACAG AGCATAAAAG 180

AATTTGCCTG GCGGCACTAG CGCGGTGGTC CCACCTGACC CCATGCCGAA CTCAGAAGTG 240

AAACGCCGTA GCGCCGATGG TAGTGTGGGG TCTCCCCATG CGAGAGTAGG GAACTGCCAG 300

GCATCAAATT AGAAGTATGG CATTAACTTT CGGGGCAGTT CACTACTCGA CAGAGAGTAG 360

GACAAAAGCG AAAGCTTTTG AACGTTGCGA AGCAACGGCC CGAAGGGTGA ATCACGAAGT 420

GATTCATAAA CTGCCAGGCA TCAAATTGCA GTAAACCGGG ATGAAAATCC GGGTTGTGAC 480

AAAGAAATTC GGTGGAGCGG 500

>(203)C Wandsworth_CP019417

ATGTTTTGGC GGATGAGAAG ACGATTTTCA GCAGTGATTC AGAGTTGAGT ACGCAATAAT 60

TTGCGCAGCA GCAAGGCGGT AAGCGAAGGA AAGGAAGGAG CATACAGAAG TATGTGACTG 120

AGTTTACGAG CGCAGGCAAC GCCGCTGATG CGATAAAGAA TTGCGTACAG AGCACAAAGA 180

ATTTGCCTGG CGGCACTAGC GCGGTGGTCC CACCTGACCC CATGCCGAAC TCAGAAGTGA 240

AACGCCGTAG CGCCGATGGT AGTGTGGGGT CTCCCCATGC GAGAGTAGGG AACTGCCAGG 300

CATCAAATTA GAAGTATGGC ATTAACTTTC GGGGCAGTTC ACTACTCGAC AGAGAGTAGG 360

ACAAAAGCGA AAGCTTTTGA ACGTTGCGAA GCAACGGCCC GAAGGGTGAA TCACGAAGTG 420

ATTCATAAAC TGCCAGGCAT CAAATTGCAG TAAACCGGGA TGAAAATCCG GGTTGTGACA 480

AAGAAATTCG GTGGAGCGG 499

>(204)C Hillingdon_CP019410

ATGTTTTGGC GGTTGAGAGA CGATTTTCAG CACTGATTCA GAGTTGAGTA CGCAATAATT 60

TGCGCAGCAG CAAGGCGGCA AGCGAAGGAA AGGAAGGAGC ATACAGAAGT ATGTGACTGA 120

CTTTACGGGC GCAGGCAACG CCGCTGATGC GATAAAGAAT TGCGTACAGA GCACAAAGAA 180

TTTGCCTGGC GGCACTAGCG CGGTGGTCCC ACCTGACCCC ATGCCGAACT CAGAAGTGAA 240

ACGCCGTAGC GCCGATGGTA GTGTGGGGTC TCCCCATGCG AGAGTAGGGA ACTGCCAGGC 300

ATCAAATTAG AAGTATGGCA TTAACTTTCG GGGCAGTTCA CTACTCGACA GAGAGTAGGA 360

CAAAAGCGAA AGCTTTTGAA CGTTGCGAAG CAAGGGCCTG AAGGGTGAAT CACGAAGTGA 420

TTCATAAACT GCCAGGCATC AAATTAAGCA GTAAACTGAT GTAAAAATCA GTGGTTGTAA 480

AAGAATTCGG TGGAGCGG 498

>(205)X Newport_Derby_NC_021902_CP075036

CTAAGGTTTT CAATCCAGAT CTTGGTGGTG ATGTACAGCT CGCTACGTGG TACGCCGCTC 60

TCAGTAATCG CCTGTCCAAC AGCCGCTTCA TTATCATAGA TTTGCGCAGT GTCGACAGCA 120

CGGTAGCCCA GTTCAAGCGC CGTTTTCACA GAAGCGATAA CCACGTCGTC TTTCAGGCGA 180

AAAGTACCTA AACCGAATGC AGGGATAGTC ATAGAATTCC TCTTTTTAAT CACTATGTTC 240

GTTAACTGAG GGGATTATGA CCTTCAGCGC TTTGTAGAAA AAGAGCGAGA AATGCAGAGG 300

ATTTTTGCGA ATTGAGCAAT AATTAAATCG CAGGCAAAGA AAAAGCCCTG AGCTAATGCT 360

CAGGGCTCTT AATAAGTGGC GGAACGGACG GGACTCGAAC CCGCGACCCC CTGCGTGACA 420

GGCAGGTATT CTAACCAACT GAACTACCGC TCCACCGAAT TCTTTTACAA CCACTGATTT 480

TTACATCAGT TTACTGCTTA ATTTGATGCC TGGCAGTTTA TGAATCACTT CGTGATTCAC 540

CCTTCGGGCC GTTGCTTCGC AACGTTCAAA AGCTTTCGCT TTTGTCCTAC TCTCTGTCGA 600

GTAGTGAACT GCCCCGAAAG TTAATGCCAT ACTTCTAATT TGATGCCTGG CAGTTCCCTA 660

CTCTCGCATG GGGAGACCCC ACACTACCAT CGGCGCTACG GCGTTTCACT TCTGAGTTCG 720

GCATGGGGTC AGGTGGGACC ACCGCGCTAG TGCCGCCAGG CAAATTCTGT TACCGGAAAC 780

GAATCTTTTA CACCGATGCT GCGTTGGCTG CATTCGCAAA GTCAGTCACA TACTTCTGTA 840

TGCTCCTTCC TTTCCTTCAT TTGCCGCCTT GCCTCAGCGT AAAATCTTTC GTTCCCGAAA 900

ATCCGGAATC AGTGCTGAAA ATCGTCTCTC ATCCGCCAAA ACATCTTCGG CGTTGTAAGG 960

TTAAGCCTCA CGGTTCATTA GTACCGGTTA GCTCAACGCA TCGCTGCGCT TACACACCCG 1020

GCCTATCAAC GTCGTCGTCT TCAACGTTCC TTCAGGAGAC TCTAAGTCTC AGGGAGAACT 1080

CATCTCGGGG CAAGTTTCGT GCTTAGATGC TTTCA 1115

>(206)C Krefeld_CP019413

ATGTTTTGGC GGATTGAGAA GACAACAATT TTCAGCTCAG ATTCAGAGTC CGAAGGATTT 60

TACGCTGAGA CAAGGCGGCA AACGCAGCGA CGGAAGGAGC ATACAGAAGT ATGTGACTGA 120

CGCTCGCAAG AGCAGCCAAC GCCGTATCAG TGTAAAAGAC ACAGGACAGA GCACAAAGAA 180

TTTGCCTGGC GGCACTAGCG CGGTGGTCCC ACCTGACCCC ATGCCGAACT CAGAAGTGAA 240

ACGCCGTAGC GCCGATGGTA GTGTGGGGTC TCCCCATGCG AGAGTAGGGA ACTGCCAGGC 300

ATCAAATTAG AAGTATGGCA TTAACTTTCG GGGCAGTTCA CTACTCGACA GAGAGTAGGA 360

CAAAAGCGAA AGCTTTTGAA CGTTGCGAAG CAACGGCCCG AAGGGTGAAT CACGAAGTGA 420

TTCATAAACT GCCAGGCATC AAATTGCAGT AAACCGGGAT GAAAATCCGG ATTGTGACAA 480

AGAAATTCGG TGGAGCGG 498

>(207)C Macclesfield_CP022117

CCGCTCCACC GAATTCTTTT ACAACCACTG ATTTTTACAT CAGTTTACTG CTTAATTTGA 60

TGCCTGGCAG TTTATGAATC ACTTCGTGAT TCACCCTTCG GGCCGTTGCT TCGCAACGTT 120

CAAAAGCTTT CGCTTTTGTC CTACTCTCTG TCGAGTAGTG AACTGCCCCG AAAGTTAATG 180

CCATACTTCT AATTTGATGC CTGGCAGTTC CCTACTCTCG CATGGGGAGA CCCCACACTA 240

CCATCGGCGC TACGGCGTTT CACTTCTGAG TTCGGCATGG GGTCAGGTGG GACCACCGCG 300

CTACGGCCGC CAGGCAAATT CTTTGTGCTC GGTACGCAAT ATTTTATCGC ATCAGCGGCG 360

TTGGCTGCAT TTGCGCTCGT CAGTCACATA CTTCAGTATG CTCCTTCCGA CGCTGCATTT 420

GCCGCCTTGC TGCTGCGCAA ACTATTGCGC ACTGCAATCT GTATCGAAGC TGAAAATCTC 480

TCATCCGCCA AAACAT 496

>(208)X_Enteritidis_CP018642

TCGGTACGCA GTTTTTTCAG GCTCTCTTTC AGGCTTGGGA TTAGCTTGTC TTTGCTAAGG 60

TTTTCAATCC AGATCTTGGT GGTGATGTAC AGCTCATTAC GCGGTACGCC GCTCTCAGCA 120

ATCGCCTGTC CAACAGCCGC TTCATTATCA TAGATTTGCG CAGTGTCGAC AGCACGGTAG 180

CCCAGTTCAA GCGCCGTTTT CACAGAAGCG ATAACCACGT CGTCTTTCAG GCGAAAAGTA 240

CCTAAACCGA ATGCAGGGAT AGTCATAGAA TTCCTCTTTT TAATCACTAT GTTCGTTAAC 300

TGAGGGGATT ATGACCTTCA GCGCTTTGTA GAAAAAGAGC GAGAAATGCA GAGGATTTTT 360

GCGAATTGAG CAATAATTAA ATGATATGCA AAGAAAAAGC CCTGAGCTAA TGCTCAGGGC 420

TCTTAATAAG TGGCGGAACG GACGGGACTC GAACCCGCGA CCCCCTGCGT GACAGGCAGG 480

TATTCTAACC AACTGAACTA CCGCTCCACC GAATTTCTTT GTCACAACCC GGATTTTCAT 540

CCCGGTTTAC TGCAATTTGA TGCCTGGCAG TTTATGAATC ACTTCGTGAT TCACCCTTCG 600

GGCCGTTGCT TCGCAACGTT CAAAAGCTTT CGCTTTTGTC CTACTCTCTG TCGAGTAGTG 660

AACTGCCCCG AAAGTTAATG CCATACTTCT AATTTGATGC CTGGCAGTTC CCTACTCTCG 720

CATGGGGAGA CCCCACACTA CCATCGGCGC TACGGCGTTT CACTTCTGAG TTCGGCATGG 780

GGTCAGGTGG GACCACCGCG CTAGTGCCGC CAGGCAAATT CTTTGTGCTC TGTACGCAAT 840

TCTTTATCGC ATCAGCGGCG TTGCCTGCGC TCGTAAACTC AGTCACATAC TTCTGTATGC 900

TCCTTCCTTT CCTTCGCTTG CCGCCTTGCT GCTGCGCAAA TTATTGCGTA CTCAACTCTG 960

AATCACTGCT GAAAATCGTC TCTCATCCGC CAAAACATCT TCGGCGTTGT AAGGTTAAGC 1020

CTCACGGTTC ATTAGTACCG GTTAGCTCAA CGCATCGCTG CGCTTACACA CCCGGCCTAT 1080

CAACGTCGTC GTCTTCAACG TTCCTTCAGG AGACTCTAAG TCTCAGGGAG AACTCATCTC 1140

GGGGCAAGTT TCGTGCTTAG ATGCTTTCAG CACTTATCTC TTCCGCATTT AGCTACCGGG 1200

CAGTGCCATT GGCATGACAA CCCGAACACC AGTGATGCGT CCACTCCGGT CCTCTCGTAC 1260

TAGGAGCAGC CCCCCTCAGT TCTCCAGCGC CCACGGCAGA TAGGGACCGA ACTGTCTCAC 1320

GACGTTCTAA ACCCAGCTCG CGTACCACTT TAAATGGCGA ACAGCCATAC CCTTGGGACC 1380

TACTTCAGCC CCAGGATGTG ATGAGCCGAC ATCGAGGTGC CAAACACCGC CGTCGATATG 1440

AACTCTTGGG CGGTATCAGC CTGTTATCCC CGGAGTACCT TTTATCCGTT GAGCGATG 1498

>(209)C Miami_NoNCBImatch

ATGTTTTGGC GGTTGAGAGA TGATTTTCAG CACTGATTCC GGATTTTCGG GAACGAAAGA 60

TAAGAGATTC GTTTCCGGCA ACAGAATTTG CCTGGCGGCA CTAGCGCGGT GGTCCCACCT 120

GACCCCATGC CGAACTCAGA AGTGAAACGC CGTAGCGCCG ATGGTAGTGT GGGGTCTCCC 180

CATGCGAGAG TAGGGAACTG CCAGGCATCA AATTAGAAGT ATGGCATTAA CTTTCGGGGC 240

AGTTCACTAC TCGACAGAGA GTAGGACAAA AGCGAAAGCT TTTGAACGTT GCGAAGCAAC 300

GGCCCGAAGG GTGAATCACG AAGTGATTCA TAAACTGCCA GGCATCAAAT TAAGCAGTAA 360

GCCGGTCATA AACTGGTGGT TGTAAAAAAA TTCGGTGGAG CGG 403

>(210)X Fresno_Javiana_CP032444_CP074283

AGAGATAAGT GCTGAAAGCA TCTAAGCACG AAACTTGCCC CGAGATGAGT TCTCCCTGAG 60

ACTTAGAGTC TCCTGAAGGA ACGTTGAAGA CGACGACGTT GATAGGCCGG GTGTGTAAGC 120

GCAGCGATGC GTTGAGCTAA CCGGTACTAA TGAACCGTGA GGCTTAACCT TACAACGCCG 180

AAGATGTTTT GGCGGATGAG AGACGATTTT CAGCACTGAT TCAGATTACA TCAGAACGTA 240

AAAACGGTCT GATAACAGAA TTTGCCTGGC GGCACTAGCG CGGTGGTCCC ACCTGACCCC 300

ATGCCGAACT CAGAAGTGAA ACGCCGTAGC GCCGATGGTA GTGTGGGGTC TCCCCATGCG 360

AGAGTAGGGA ACTGCCAGGC ATCAAATTAA GCAGTAAACT GATGTAAAAA TCAGTGGTTG 420

TAAAAGAATT CGGTGGAGCG GTAGTTCAGT TGGTTAGAAT ACCTGCCTGT CACGCAGGGG 480

GTCGCGGGTT CGAGTCCCGT CCGTTCCGCC ACTTATTAAG AGCCCTGAGC ATTAGCTCAG 540

GGCTTTTTCT TTGCATGTCA TTTAATTATT GCTCAATTCG CAAAAATCCT CTGCATTTCT 600

CGCTCTTTTT CTGCAAAGCG CTGAAGGTCA TAATCCCCTC AGTTGACGAA CATAGTGATT 660

AAAAAGAGGA ATTCTATGAC TATCCCTGCA TTCGGTTTAG GTACTTTTCG CCTGAAAGAC 720

GACGTGGTTA TCGCTTCTGT TAAAACGGCG CTTGAACTGG GCTACCGTGC TGTCGACACT 780

GCACAGATCT ATGATAATGA AGCGGCTGTT GGACAGGCGA TTGCTGAAAG CGGTGTACCA 840

CGTAATGAGC TGTACATCAC CACCAAGATC TGGATTGAAA ACCTTAGCAA AGACAAACTA 900

ATCCCAAGCC TGAAAGAGAG CCTGAAAAAA CTGCGTACCG ATTATGTCGA TCTGACGCTG 960

ATCCACTGGC CATCTCCGGG TGATGCTGTG TCTGTCGAAG AATTTATGCA GGCATTGCTG 1020

GAAGCTAAAA AACAAGGGCT GACTCGCGAA ATTGGCATTT CAAACTTCAC CATTCCGTTG 1080

ATGAAAAAGG CTATTGCCGC GGTGGGCGCA GACCATATCG CCACCAACCA GATTGAGCTA 1140

TCTCCGTATC TGCAGAATC 1159

>(211)C Heidelberg_LS483494

ATGTTTTGGC GGATTGAGAA GACAACAATT TTCAGCTCAG ATTCAGAGTC CGAAGGATTT 60

TACGCTGAGA CAAGGCGGCA AACGCAGCGA CGGAAGGAGC ATACAGAAGT ATGTGACTGA 120

CGCTCGCAAG AGCAGCCAAC GCCGTATCAG TGTAAAAGAC ACAGGACGGA GCACAAAGAA 180

TTTGCCTGGC GGCACTAGCG CGGTGGTCCC ACCTGACCCC ATGCCGAACT CAGAAGTGAA 240

ACGCCGTAGC GCCGATGGTA GTGTGGGGTC TCCCCATGCG AGAGTAGGGA ACTGCCAGGC 300

ATCAAATTAG AAGTATGGCA TTAACTTTCG GGGCAGTTCA CTACTCGACA GAGAGTAGGA 360

CAAAAGCGAA AGCTTTTGAA CGTTGCGAAG CAACGGCCCG AAGGGTGAAT CACGAAGTGA 420

TTCATAAACT GCCAGGCATC AAATTGCAGT AAACCGGGAT GAAAATCCGG GTTGTGACAA 480

AGAAATTCGG TGGAGCGG 498

>(212)C Kentucky_CP082582

ATGTTTTGGC GGATGAGAGA AGATTTTCAG CACTGATTCA GAGTTGAGTA CGCAATAATT 60

TGCGCAGCAG CAAGGCGGCA AGCGAAGGAA AGGAAGGAGC ATACAGAAGT ATGTGACTGA 120

GTTTACGAGC GCAGGCAACG CCGCTGATGC GATAAAGAAT TGCGTACTGA GCATAAAAGA 180

ATTTGCCTGG CGGCAGTAGC GCGGTGGTCC CACCTGACCC CATGCCGAAC TCAGAAGTGA 240

AACGCCGTAG CGCCGATGGT AGTGTGGGGT CTCCCCATGC GAGAGTAGGG AACTGCCAGG 300

CATCAAATTA GAAGTATGGC ATTAACTTTC GGGGCAGTTC ACTACTCGAC AGAGAGTAGG 360

ACAAAAGCGA AAGCTTTTGA ACGTTGCGAA GCAACGGCCC GAAGGGTGAA TCACGAAGTG 420

ATTCATAAAC TGCCAGGCAT CAAATTGCAG TAAACCGGGA TGAAAATCCG GGTTGTGACA 480

AAGAAATTCG GTGGAGCGG 499

>(213)X Tennessee_Montevideo_Typhimurium_CP007505_CP030029_CP034232_

TGCGGAAGAG ATAAGTGCTG AAAGCATCTA AGCACGAAAC TTGCCCCGAG ATGAGTTCTC 60

CCTGAGACTT TAGGTCTCCT GAAGGAACGT TGAAGACGAC GACGTTGATA GGCCGGGTGT 120

GTAAGCGCAG CGATGCGTTG AGCTAACCGG TACTAATGAA CCGTGAGGCT TAACCTTACA 180

ACGCCGAAGA TGTTTTGGCG GATGAGAGAC GATTTTCAGC ACTGATTCAG ATTACATCAG 240

AACGTAAAAA CGGTCTGATA ACAGAATTTG CCTGGCGGCA CTAGCGCGGT GGTCCCACCT 300

GACCCCATGC CGAACTCAGA AGTGAAACGC CGTAGCGCCG ATGGTAGTGT GGGGTCTCCC 360

CATGCGAGAG TAGGGAACTG CCAGGCATCA AATTAAGCAG TAAACTGATG TAAAAGTCAG 420

TGGTTGTAAA AGAATTCGGT GGAGCGGTAG TTCAGTTGGT TAGAATACCT GCCTGTCACG 480

CAGGGGGTCG CGGGTTCGAG TCCCGTCCGT TCCGCCACTT ATTAAGAGCC CTGAGCATTA 540

GCTCAGGGCT TTTTCTTTGC ATGTCATTTA ATTATTGCTC AATTCGCAAA AATCCTCTGC 600

ATTTCTCGCT CTTTTTCTGC AAAGCGCTGA AGGTCATAAT CCCCTCAGTT AACGAACATA 660

GTGATTAAAA AGAGGAATTC TATGACTATC CCTGCATTCG GTTTAGGTAC TTTTCGCCTG 720

AAAGACGACG TGGTTATCGC TTCTGTTAAA ACGGCGCTTG AACTGGGCTA CCGTGCTGTC 780

GACACTGCAC AGATCTATGA TAATGAAGCG GCTGTTGGAC AGGCGATTGC TGAAAGCGGT 840

GTACCACGTA ATGAGCTGTA CATCACCACC AAGATCTGGA TTGAAAACCT TAGCAAAGAC 900

AAGCTAATCC CAAGCCTGAA AGAGAGCCTG AAAAAACTGC GTACCGATTA TGTCGATCTG 960

ACGCTGATCC ACTGGCCATC TCCGGGTGAT GCCGTGTCTG TCGAAGAATT TATGCAGGCA 1020

TTGCTGGAAG CTAAAAAACA AGGGCTGACT CGCGAAATTG GCATTTCAAA CTTCACCATT 1080

CCGTTGATGG AAAAGGCTAT TGCCGCGGTG GGCGCAGACC ATATCGCCAC CAACCAGATT 1140

GAGCTATCTC CGTATCTGCA GATCGTAAGG GTTGATGG 1178

>(214)C Kentucky_NoNCBImatch

ATGTTTTGGC GGATGAGAGA CGATTTTCAG CACTGATTCA GAGTTGAGTA CGCAATAATT 60

TGCGCAGCAG CAAGGCGGCA AGCGAAGGAA AGGAAGGAGC ATACAGAAGT ATGTGACTGA 120

GTTTACGAGC GCAGGCAACG CCGCTGATGC GATAAAGAAT TGCGTACAGA GCACAAAGAA 180

TTTGCCTGGC GGCACTAGCG CGGTGGTCCC ACCTGACCCC ATGCCGAACT CAGAAGTGAA 240

ACGCCGTAGC GCCGATGGTA GTGTGGGGTC TCCCCATGCG AGAGTAGGGA ACTGCCAGGC 300

ATCAAATTAG AAGTATGGCA TTAACTTTCG GGGCAGTTCA CTACTCGACA GAGAGTAGGA 360

CAAAAGCGAA AGCTTTTGAA CGTTGCGAAG CAACGGCCCG AAGGGTGAAT CACGAAGTGA 420

TTCATAAACT GCCAGGCATC AAATTGCAGT AAACCGGGAT GAAAATCCGG ATTGTGACAA 480

AGAAATTCGG TGGAGCGG 498

>(215)C Orion_NoNCBImatch

ATGTTTTGGC GGATGAGAAG ACGATTTTCA GCAGTGATTC AGAGTTGAGT ACGCAATAAT 60

TTGCGCAGCA GCAAGGCGGC AAGCGAAGGA AAGGAAGGAG CATACAGAAG TATGTGACTG 120

AGTTTACGAG CGCAGGCAAC GCCGCTGATG CGATAAAGAA TTGCGTACAG AGCACAAAGA 180

ATTTGCCTGG CGGCCGTAGC GCGGTGGTCC CACCTGACCC CATGCCGAAC TCAGAAGTGA 240

AACGCCGTAG CGCCGATGGT AGTGTGGGGT CTCCCCATGC GAGAGTAGGG AACTGCCAGG 300

CATCAAATTA AGCAGTAAGC CGGTCATAGA CTGGTGGTTG TAAAAGAATT CGGTGGAGCG 360

G 361

>(216)C Unique_NoNCBImatch

CTGTTTTGGC GGATTTGAGA AGACAGATTT TCAGCCTGAT ACAGATAAGA TTAACCGGCG 60

AAAAGCGGGT TAATAAACAG AATTTGCCTG GCGGCTGTAG CGCGGTGGTC CCACCTGACC 120

CCATGCCGAA CTCAGAAGTG AAACGCCGTA GCGCCGATGG TAGTGTGGGG TCTCCCCATG 180

CGAGAGTAGG GAACTGCCAG GCATCAAATA GCAGTAAACC GGGATGAAAA TCCGGGTGGT 240

GACAAAGAAA TTCGGTGGAG CGGT 264

>(217)C Reading_NoNCBImatch

ATGTTTTGGC GGATGAGAGA CGATTTTCAG CACTGATTCA GAGTTGAGTA CGCAATAATT 60

TGCGCAGCAG CAAGGCGGCA AGCGAAGGAA AGGAAGGAGC ATACAGAAGT ATGTGACTGA 120

CTTTACGAGC GCAGGCAACG CCGCTGATGC GATAAAGAAT TGCGTACAGA GCACAAAAGA 180

ATTTGCCTGG CGGCACTAGC GCGGTGGTCC CACCTGACCC CATGCCGAAC TCAGAAGTGA 240

AACGCCGTAG CGCCGATGGT AGTGTGGGGT CTCCCCATGC GAGAGTAGGG AACTGCCAGG 300

CATCAAATTA GAAGTATGGC ATTAACTTTC GGGGCAGTTC ACTACTCGAC AGAGAGTAGG 360

ACAAAAGCGA AAGCTTTTGA ACGTTGCGAA GCAACGGCCC GAAGGGTGAA TCACGAAGTG 420

ATTCATAAAC TGCCAGGCAT CAAATTAAGC AGTAAACTGA TGTAAAAATC AGTGGTTGTA 480

AAAGAATTCG GTGGAGCGG 499

>(218)C Unique_NoNCBImatch

ATGTTTTGGC GGATGAGAGA AGATTTTCAG CACTGATTCA GATTACATCA GAACGTAAAA 60

ACGGTCTGAT AACAGAATTT GCCTGGCGGC ACTAGCGCGG TGGTCCCACC TGACCCCATG 120

CCGAACTCAG AAGTGAAACG CTGTAGCGCC GATGGTAGTG TGGGGTCTCC CCATGCGAGA 180

GTAGGGAACT GCCAGGCATC AAATTAGAAG TATGGCATTA ACTTTCGGGG CAGTTCACTA 240

CTCGACAGAG AGTAGGACAA AAGCGAAAGC TTTTGAACGT TGCGAAGCAA CGGCCCGAAG 300

GGTGAATCAC GAAGTGATTC ATAAACTGCC AGGCATCAAA TTGCAGTAAA CCGGGATGAA 360

AATCCGGGTT GTGACAAAGA AATTCGGTGG AGCGG 395

>(219)C Unique_NoNCBImatch

ATGTTTTGGC GGATTGAGAA GACAACAATT TTCAGCTCAG ATTCAGAGTC CGAAGGATTT 60

TACGCTGAGA CAAGGCGGCA AACGCAGCGA CGGAAGGAGC ATACAGAAGT ATGTGACTGA 120

CGCTCGCAAG AGCAGCCAAC GCCGTATCAG TGTAAAAGAC ACAGGACGGA GCACAAAGAA 180

TTTGCCGGGC GGCACTAGCG CGGTGGTCCC ACCTGACCCC ATGCCGAACT CAGAAGTGAA 240

ACGCCGTAGC GCCGATGGTA GTGTGGGGTC TCCCCATGCG AGAGTAGGGA ACTGCCAGGC 300

ATCAAATTAG AAGTATGGCA TTAACTTTCG GGGCAGTTCA CTACTCGACA GAGAGTAGGA 360

CAAAAGCGAA AGCTTTTGAA CGTTGCGAAG CAACGGCCCG AAGGGTGAAT CACGAAGTGA 420

TTCATAAACT GCCAGGCATC AAATTAAGCA GTAAACTGAT GTAAAAATCA GTGGTTGTAA 480

AAGAATTCGG TGGAGCGG 498

>(220)C Newport_CP025232

ATGTTTTGGC GGATGAGAGA CGATTTTCAG CTCAGATTCA GATTACATCA GAACGTAAAA 60

ACGGTCTGAT AACAGAATTT GCCTGGCGGC ACTAGCGCGG TGGTCCCACC TGACCCCATG 120

CCGAACTCAG AAGTGAAACG CCGTAGCGCC GATGGTAGTG TGGGGTCTCC CCATGCGAGA 180

GTAGGGAACT GCCAGGCATC AAATTAGAAG TATGGCATTA ACTTTCGGGG CAGTTCACTA 240

CTCGACAGAG AGTAGGACAA AAGCGAAAGC TTTTGAACGT TGCGAAGCAA CGGCCCGAAG 300

GGTGAATCAC GAAGTGATTC ATAAACTGCC AGGCATCAAA TTAAGCAGTA AACTGATGTA 360

AAAATCAGTG GTTGTAAAAG AATTCGGTGG AGCGG 395

>(221)C Onderstepoort_CP022034

ATGTTTTGGC GATTGAGAAG ACGATTTTCA GCACTGATTC CGGATTTTCG GGAACGAAAG 60

ATAAAAGATT CGTTTCCGGC AACAGAATTC GCCTGGCGGC ACTAGCGCGG TGGTCCCACC 120

TGACCCCATG CCGAACTCAG AAGTGAAACG CCGTAGCGCC GATGGTAGTG TGGGGTCTCC 180

CCATGCGAGA GTAGGGAACT GCCAGGCATC AAATTAGAAG TATGGCATTA ACTTTCGGGG 240

CAGTTCACTA CTCGACAGAG AGTAGGACAA AAGCGAAAGC TTTTGAACGT TGCGAAGCAA 300

CGGCCCGAAG GGTGAATCAC GAAGTGATTC ATAAACTGCC AGGCATCAAA TTGCAGTAAA 360

CCGGGATGAA AATCCGGGTT GTGACAAAGA AATTCGGTGG AGCGG 405

>(222)C Abaetetuba_Newport_CP007532_CP074207

ATGTTTTGGC GGATGAGAAG ACAGATTTTC AGCACTGATT CAGATTACAT CAGAACGTAA 60

AAACGGTCTG ATAACAGAAT TTGCCTGGCG GCAGTAGCGC GGTGGTCCCA CCTGACCCCA 120

TGCCGAACTC AGAAGTGAAA CGCCGTAGCG CCGATGGTAG TGTGGGGTCT CCCCATGCGA 180

GAGTAGGGAA CTGCCAGGCA TCAAATTGCA GTAAACCGGG ATGAAAATCC GGGTTGTGAC 240

AAAGAAATTC GGTGGAGCGG 260

>(223)C Antsalova_CP019116

ATGTTTTGGC GGATGAGAGA CGATTTTCAG CACTGATTCA GAGTTGAGTA CGCAATAATT 60

TGCGCAGCAG CAAGGCGGCA AGCGAAGGAA AGGAAGGAGC ATACAGAAGT ATGTGACTGA 120

GTTTACGAGC GCGGGCAACG CCGCTGATGC GATAAAGAAT TGCGTACTGA GCATAAAAGA 180

ATTTGCCTGG CGGCACTAGC GCGGTGGTCC CACCTGACCC CATGCCGAAC TCAGAAGTGA 240

AACGCCGTAG CGCCGATGGT AGTGTGGGGT CTCCCCATGC GAGAGTAGGG AACTGCCAGG 300

CATCAAATTG CAGTAAACCA GGATCAAAAT CCGGGTTGTG ACAAAAAAAT TCGGTGGAGC 360

GG 362

>(224)C Apapa_CP019403

ATGTTTTGGC GGTTGAGAGA CGATTTTCAG CACTGATTCA GAGTTGAGTA CGCAATAATT 60

TGCGCAGCAG CAAGGCGGCA AGCGAAGGAA AGGCAGGAGC ATACAGAAGT ATGTGACTGA 120

CTTTACGAGC GCAGGCAACG CCGCTGATGC GATAAAGAAT TGCGTACTGA GCACAAAAGA 180

ATTTGCCTGG CGGCACTAGC GCGGTGGTCC CACCTGACCC CATGCCGAAC TCAGAAGTGA 240

AACGCCGTAG CGCCGATGGT AGTGTGGGGT CTCCCCATGC GAGAGTAGGG AACTGCCAGG 300

CATCAAATTA AGCAGTAAAC TGATGTAAAA ATCAGTGGTT GTAAAAGAAT TCGGTGGAGC 360

GG 362

>(225)C Djakarta_CP019409

ATGTTTTGGC GGATGAGAGA CGATTTTCAG CACTGATTCA GAGTTGAGTA CGCAATAATT 60

TGCGCAGCAG CAAGGCAGCA AGCGAAGGAA AGGAAGGAGC ATACAGAAGT ATGTGACTGA 120

CTTTACGAGC GCAGGCAACG CCGCTGATGC GATAAAGAAT TGCGTACTGA GCACAAAAGA 180

ATTTGCCTGG CGGCACTAGC GCGGTGGTCC CACCTGACCC CATGCCGAAC TCAGAAGTGA 240

AACGCCGTAG CGCCGATGGT AGTGTGGGGT CTCCCCATGC GAGAGTAGGG AACTGCCAGG 300

CATCAAATTA AGCAGTAAGC CGGTCATAAA CCGGTGGTTG TAAAAGAATT CGGTGGAGCG 360

G 361

>(226)C Hvittingfoss_CP022503

CTGTTTTGGC GGATTGAGAA GACAACAATT TTCAGCTCAG ATTCAGAGTC CGAAGGATTT 60

TACGCTGAGA CAAGGCGGCA AACGCAGCGA CGGAAGGAGC ATACAGAAGT ATGTGACTGA 120

CGCCCGCAAG AGCAGCCAAC GCCGTATCAG TGTAAAAGAC ACAGGACAGA GCACAAAGAA 180

TTTGCCTGGC GGCACTAGCG CGGTGGTCCC ACCTGACCCC ATGCCGAACT CAGAAGTGAA 240

ACGCCGTAGC GCCGATGGTA GTGTGGGGTC TCCCCATGCG AGAGTAGGGA ACTGCCAGGC 300

ATCAAATTGC AGTAAACCGG GATGAAAATC CGGGTTGTGA CAAAGAAATT CGGTGGAGCG 360

G 361

>(227)C Quebec_CP022019

ATGTTTTGGC GGTTGAGAGA CGATTTTCAG CACTGATTCA GATTACATCA GAACGTAAAA 60

ACGGTCTGAT AACAGAATTT GCCTGGCGGC ACTAGCGCGG TGGTCCCACC TGACCCCATG 120

CCGAACTCAG AAGTGAAACG CCGTAGCGCC GATGGTAGTG TGGGGTCTCC CCATGCGAGA 180

GTAGGGAACT GCCAGGCATC AATTTAAGCA GTAAACTGAT GTAAAAATCA GTGGTTGTAA 240

AAGAATTCGG TGGAGCGG 258

>(228)C Sloterdijk_CP012349

ATGTTTTGGC GGATTGAGAG ACGATTTTCA GCACTGATTC AGAGTTGAGT ACGCAATAAT 60

TTGCGCAGCA GCAAGGCGGC AAGCGAAGGA AAGGAAGGAG CATACAGAAG TATGTGACTG 120

AGTTTACGAG CGCAGGCAAC GCCGCTGATG CGATAAAGAA TTGCGTACAG AGCACAAAGA 180

ATTTGCCTGG CGGCACTAGC GCGGTGGTCC CACCTGACCC CATGCCGAAC TCAGAAGTGA 240

AACGCCGTAG CGCCGATGGT AGTGTGGGGT CTCCCCATGC GAGAGTAGGG AACTGCCAGG 300

CATCAAATTA AGCAGTAAAC TGATGTAAAA ATCAGTGGTT GTAAAAGAAT TCGGTGGAGC 360

GG 362

>(229)C Waycross_CP022138

CTGTTTTGGC GGACTGAGAA GACGAACAAT TTTCAGCACT GATTCCGGAT TTTCGGGAAC 60

GAAAGATTTT ACGCTGAGGC AAGGCGGCAA ATGAAGGAAA GGAAGGAGCA TACAGAAGTA 120

TGTGACTGAC TTTACGAATG CAGCCAACGC AGCATCGGTG TAAAAGATTC GTTTCTGGCA 180

ACAGAATTTG CCTGGCGGCA GTAGCGCGGT GGTCCCACCT GACCCCATGC CGAACTCAGA 240

AGTGAAACGC CGTAGCGCCG ATGGTAGTGT GGGGTCTCCC CATGCGAGAG TAGGGAACTG 300

CCAGGCATCA AATTGCAGTA AACCGGGATG AAAATCCGGG TTGTGACAAA GAAATTCGGT 360

GGAGCGG 367

>(230)C India_CP022015

ATGTTTTGGC GGATGAGAAG ACGATTTTCA GCACTGATTC AGAGTTGAGT ACGCAATAAT 60

TTGCGCAGCA GCAAGGCGGC AAGCGAAGGA AAGGAAGGAG CATACAGAAG TATGTGACTG 120

AGTTTACGAG CGCAGGCAAC GCCGCTGATG CGATAAAGAA TTGCGTACTG AGCATAAAAG 180

AATTTGCCTG GCGGCACTAG CGCGGTGGTC CCACCTGACC CCATGCCGAA CTCAGAAGTG 240

AAACGCCGTA GCGCCGATGG TAGTGTGGGG TCTCCCCATG CGAGAGTAGG GAACTGCCAG 300

GCATCAATTT AAGCAGTAAA CTGATGTAAA AATCAGTGGT TGTAAAAGAA TTCGGTGGAG 360

CGG 363

>(231)C Alachua_NoNCBImatch

ATGTTTTGGC GGATGAGAAG ACGATTTTCA GCACTGATTC AGAGTTGAGT ACGCAATAAT 60

TTGCGCAGCA GCAAGGCGGC AAGCGAAGGA AAGGAAGGAG CATACAGAAG TATGTGACTG 120

AGTTTACGAG CGCAGGCAAC GCCGCTGATG CGATAAAGAA TTGCGTACAG AGCACAAAGA 180

ATTTGCCTGG CGGCACTAGC GCGGTGGTCC CACCTGACCC CATGCCGAAC TCAGAAGTGA 240

AACGCCGTAG CGCCGATGGT AGTGTGGGGT CTCCCCATGC GAGAGTAGGG AACTGCCAGG 300

CATCAAATTA AGCAGTAAGC CGGTCATAGA CTGGTGGTTG TAAAAGAATT CGGTGGAGCG 360

G 361

>(232)C Kentucky_NoNCBImatch

ATGTTTTGGC GGATGAGAGA CGATTTTCAG CACTGATTCA GATTACATCA GAACGTAAAA 60

ACGGTCTGAT AACAGAATTT GCCTGGCGGC ACTAGCGCGG TGGTCCCACC TGACCCCATG 120

CCGAACTCAG AAGTGAAACG CCGTAGCGCC GATGGTAGTG TGGGGTCTCC CCATGCGAGA 180

GTAGGGAACT GCCAGGCATC AAATTAGAAG TATGGCATTA ACTTTCGGGG CAGTTCACTA 240

CTCGACAGAG AGTAGGACAA AAGCGAAAGC TTTTGAACGT TGCGAAGCAA CGGCCCGAAG 300

GGTGAATCAC GAAGTGATTC ATAAACTGCC AGGCATCAAA TTGCAGTAAA CCGGGATGAA 360

AATCCGGATT GTGACAAAGA AATTCGGTGG AGCGG 395

>(233)C Haifa_NoNCBImatch

ATGTTTTGGC GGTTGAGAGA AGATTTTCAG CACTGATTCC GGATTTTCGG GAACGAAAGA 60

TTTTACGCTG AGGCAAGGCG GCAAATGAAG GAAAGGAAGG AGCATACAGA AGTATGTGAC 120

TGACTTTACG AATGCAGCCA ACGCAGCATC GGTGTAAAAG ATTCGTTTCC GGCAACAGAA 180

TTTGCCTGGC GGCCGTAGCG CGGTGGTCCC ACCTGACCCC ATGCCGAACT CAGAAGTGAA 240

ACGCCGTAGC GCCGATGGTA GTGTGGGGTC TCCCCATGCG AGAGTAGGGA ACTGCCAGGC 300

ATCAAATTAG AAGTATGGCA TTAACTTTCG GGGCAGTTCA CTACTCGACA GAGAGTAGGA 360

CAAAAGCGAA AGCTTTTGAA CGTTGCGAAG CAACGGCCCG AAGGGTGAAT CACGAAGTGA 420

TTCATAAACT GCCAGGCATC AAATTAAGCA GTAAACTGAT GTAAAAATCA GTGGTTGTAA 480

AAGAATTCGG TGGAGCGG 498

>(234)C London_Concord_CP060132_CP028196

CTGTTTTGGC GGATTGAGAA GACAACAATT TTCAGCTCAG ATTCAGAGTC CGAAGGATTT 60

TACGCTGAGA CAAGGCGGCA AACGCAGCGA CGGAAGGAGC ATACAGAAGT ATGTGACTGA 120

CGCTCGCAAG AGCAGCCAAC GCCGTATCAG TGTAAAAGAC ACAGGACAGA GCACAAAGAA 180

TTTGCCTGGC GGCACTAGCG CGGTGGTCCC ACCTGACCCC ATGCCGAACT CAGAAGTGAA 240

ACGCCGTAGC GCCGATGGTA GTGTGGGGTC TCCCCATGCG AGAGTAGGGA ACTGCCAGGC 300

ATCAAATTAG AAGTATGGCA TTAACTTTCG GGGCAGTTCA CTACTCGACA GAGAGTAGGA 360

CAAAAGCGAA AGCTTTTGAA CGTTGCGAAG CAACGGCCCG AAGGATGAAT CACGAAGTGA 420

TTCATAAACT GCCAGGCATC AAATTAAGCA GTAAACTGAT GTAAAAATCA GTGGTTGTAA 480

AAGAATTCGG TGGAGCGG 498

>(235)C Unique_NoNCBIMatch

ATGTTTTGGC GGATGAGAGA CGATTTTCAG CACTGATTCA GAGTTGAGTA CGCAATAATT 60

TGCGCAGCAG CAAGGCGGCA AGCGAAGGAA AGGAAGGAGC ATACAGAAGT ATGTGACTGA 120

CTTTACGAGC GCGGGCAACG CCGCTGATGC GATAAAGAAT TGCGTACAGA GCACAAAAGA 180

ATTTGCCTGG CGGCCGTAGC GCGGTGGTCC CACCTGACCC CATGCCGAAC TCAGAAGTGA 240

AACGCCGTAG CGCCGATGGT AGTGTGGGGT CTCCCCATGC GAGAGTAGGG AACTGCCAGG 300

CATCAAATTA AGCAGTAAAC TGATGTAAAA ATCAGTGGTT GTAAAAGAAT TCGGTGGAGC 360

GG 362

>(236)C Paratyphi_B_CP074668

ATGTTTTGGC GGATTGAGAA GACAACAATT TTCAGCTCAG ATTCAGAGTC CGAAGGATTT 60

TACGCTGAGA CAAGGCGGCA AACGCAGCGA CGGAAGGAGC ATACAGAAGT ATGTGACTGA 120

CGCTCGCAAG AGCAGCCAAC GCCGTATCAG TGTAAAAGAC ACAGGACAGA GCACAAAGAA 180

TTTGCCTGGC GGCAGTAGCG CGGTGGTCCC ACCTGACCCC ATGCCGAACT CAGAAGTGAA 240

ACGCCGTAGC GCCGATGGTA GTGTGGGGTC TCCCCATGCG AGAGTAGGGA ACTGCCAGGC 300

ATCAAATTAG AAGTATGGCA TTAACTTTCG GGGCAGTTCA CTACTCGACA GAGAGTAGGA 360

CAAAAGCGAA AGCTTTTGAA CGTTGCGAAG CAAGGGCCCG AAGGGTGAAT CACGAAGTGA 420

TTCATAAACT GCCAAGCATC AAATTAAGCA GTAAACTGAT GTAAAAATCA GTGGTTGTAA 480

AAGAATTCGG TGGAGCGG 498

>(237)C Saintpaul_CP023512

ATGTTTTGGC GGATGAGAGA CGATTTTCAG CACTGATTCC GGATTTTCGG GGACGAAAGA 60

TAAAAGATTC GTTTCCGGCA ACAGAATTTG CCTGGCGGCA CTAGCGCGGT GGTCCCACCT 120

GACCCCATGC CGAACTCAGA AGTGAAACGC CGTAGCGCCG ATGGTAGTGT GGGGTCTCCC 180

CATGCGAGAG TAGGGAACTG CCAGGCATCA AATTAGAAGT ATGGCATTAA CTTTCGGGGC 240

AGTTCACTAC TCGACAGAGA GTAGGACAAA AGCGAAAGCT TTTGAACGTT GCGAAGCAAC 300

GGCCCGAAGG GTGAATCACG AAGTGATTCA TAAACTGCCA GGCATCAAAT TGCAGTAAAC 360

CGGGATGAAA ATCCGGGTTG TGACAAAGAA ATTCGGTGGA GCGG 404

>(238)C Duisburg _NoNCBImatch

ATGTTTTGGC GGTTGAGAAG ACGATTTTCA GCACTGATTC AGAGTTGAGT ACGCAATAAT 60

TTGCGCAGCA GCAAGGCGGC AAGCGAAGGA AAGGAAGGAG CATACAGAAG TATGTGACTG 120

AGTTTACGAG CGCAGGCAAC GCCGCTGATG CGATAAAGAA TTGCGTACAG AGCACAAAGA 180

ATTTGCCTGG CGGCAGTAGC GCGGTGGTCC CACCTGACCC CATGCCGAAC TCAGAAGTGA 240

AACGCCGTAG CGCCGATGGT AGTGTGGGGT CTCCCCATGC GAGAGTAGGG AACTGCCAGG 300

CATCAAATTA GAAGTATGGC ATTAACTTTC GGGGCAGTTC ACTACTCGAC AGAGAGTAGG 360

ACAAAAGCGA AAGCTTTTGA ACGTTGCGAA GCAACGGCCC GAAGGGTGAA TCACGAAGTG 420

ATTCATAAAC TGCCAGGCAT TAAATTGCAG TAAACCGGGA TGAAAATCCG GGTTGTGACA 480

AAGAAATTCG GTGGAGCGG 499

>(239)C Pomono_NoNCBImatch

ATGTTTTGGC GGACTGAGAG ACGATTTTCA GCACTGATTC CGGATTTTCG GGAACGAAAG 60

ATAAGAGATT CGTTTCCGGC AACAGAATTT GCCTGGCGGC ACTAGCGCGG TGGTCCCACC 120

TGACCCCATG CCGAACTCAG AAGTGAAACG CCGTAGCGCC GATGGTAGTG TGGGGTCTCC 180

CCATGCGAGA GTAGGGAACT GCCAGGCATC AAATTAGAAG TATGGCATTA ACTTTCGGGG 240

CAGTTCACTA CTCGACAGAG AGTAGGACAA AAGCGAAAGC TTTTGAACGT TGCGAAGCAA 300

CGGCCCGAAG GGTGAATCAC GAAGTGATTC ATAAACTGCC AGGCATCAAA TTAAGCAGTA 360

AGCCGGTCAT AAACTGGTGG TTGTAAAAAA ATTCGGTGGA ACGG 404

>(240)C Unique_NoNCBImatch

GTGTTTTGGC GGTTGAGAAG ACGATTTTCA GCACTGATTC CGGATTTTCG GGAACGAAAG 60

ATTTTACGCT GAGGCAAGGC GGCAAATGAA GGAAAGGAAG GAGCATACAG AAGTATGTGA 120

CTGACTTTAC GAATGCAGCC AACGCAGCAT CGGTGTAAAA GATTCGTTTC CGGCAACAGA 180

ATTTGCCTGG CGGCAGTAGC GCGGTGGTCC CACCTGACCC CATGCCGAAC TCAGAAGTGA 240

AACGCCGTAG CGCCGATGGT AGTGTGGGGT CTCCCCATGC GAGAGTAGGG AACTGCCAGG 300

CATCAAATTG CAGTAAACCG GGATGAAAAT CCGGGTTGTG ACAAAGAAAT TCGGTGGAGC 360

GG 362

>(241)C Goldcoast_LR134158

ATGTTTTGGC GGATTGAGAA GACAACAATT TTCAGCTCAG ATTCAGAGTC CGAAGGATTT 60

TACGCTGAGA CAAGGCGGCA AACGCAGCGA CGGAAGGAGC ATACAGAAGT ATGTGACTGA 120

CGCTCGCAAG AGCAGCCAAC GCCGTATCAG TGTAAAAGAC ACAGGACAGA GCACAAAGAA 180

TTTGCCTGGC GGCACTAGCG CGGTGGTCCC ACCTGACCCC ATGCCGAACT CAGAAGTGAA 240

ACGCCGTAGC GCCGATGGTA GTGTGGGGTC TCCCCATGCG AGAGTAGGGA ACTGCCAGGC 300

ATCAAATTAG AAGTATGGCA TTAACTTTCG GGGCAGTTCA CTACTCGACA GAGAGTAGGA 360

CAAAAGCGAA AGCTTTTGAA TGTTGCGAAG CAACGGCCCG AAGGGTGAAT CACGAAGTGA 420

TTCATAAACT GCCAGGCATC AAATTGCAGG AAACCGGGAT GAAAATCCGG GTTGTGACAA 480

AGAAATTCGG TGGAGCGG 498

>(242)X Corvallis CP027677

AATCCAGATC TTGGTGGTGA TGTACAGCTC GCTACGCGGT ACGCCGCTCT CAGCAATCGC 60

CTGTCCAACA GCCGCTTCAT TATCATAGAT TTGCGCAGTG TCGACAGCAC GGTAGCCCAG 120

TTCAAGCGCC GTTTTAACAG AAGCGATAAC CACGTCGTCT TTCAGGCGAA AAGTACCTAA 180

ACCGAATGCA GGGATAGTCA TAGAATTCCT CTTTTTAATC ACTATGTTCG TTAACTGAGG 240

GAATTATGAC CTTCAGCGCT TTGCAGAAGA AGAGCGAGAA ATGCAGAGGA TTTTTGCGAA 300

TTGAGCAATA ATTAAATGAC ATGCAAAGAA AAAGCCCTGA GCTAATGCTC AGGGCTCTTA 360

ATAAGTGGCG GAACGGACGG GACTCGAACC CGCGACCCCC TGCGTGACAG GCAGGTATTC 420

TAACCAACTG AACTACCGCT CCACCGAATT CTTTTACAAC CACTGACTTT TACATCAGTT 480

TACTGCTTAA TTTGATGCCT GGCAGTTCCC TACTCTCGCA TGGGGAGACC CCACACTACC 540

ATCGGCGCTA CGGCGTTTCA CTTCTGAGTT CGGCATGGGG TCAGGTGGGA CCACCGCGCT 600

AGTGCCGCCA GGCAAATTCT GTTATCAGAC CGTTTTTACG TTCTGATGTA ATCTGAATCA 660

GTGCTGAAAA TCATCTCTCA TCCGCCAAAA CATCTTCGGC GTTGTAAGGT TAAGCCTCAC 720

GGTTCATTAG TACCGGTTAG CTCAACGCAT CGCTGCGCTT ACACACCCGG CCTATCAACG 780

TCGTCGTCTT CAACGTTCCT TCAGGAGACT CTAAGTCTCA GGGAGAACTC ATCTCGGGGC 840

AAGTTTCGTG CTTAGATGCT TTCAGCACTT ATCTCTTCCG CATTTAGCTA CCGG 894

>(243)C Unique_NoNCBImatch

ATGTTTTGGC GGATGAGAGA TGATTTTCAG CACTGATTCC GGATTTTCGG GAACGAAAGA 60

TAAAAGATTC GTTTCCGGCA ACAGAATTTG CCTGGCGGCA CTAGCGCGGT GGTCCCACCT 120

GACCCCATGC CGAACTCAGA AGTGAAACGC CGTAGCGCCG ATGGTAGTGT GGGGTCTCCC 180

CATGCGAGAG TAGGGAACTG CCAGGCATCA AATTAGAAGT ATGGCATTAA CTTTCGGGGC 240

AGTTCACTAC TCGACAGAGA GTAGGACAAA AGCGAAAGCT TTTGAACGTT GCGAAGCAAC 300

GGCCCGAAGG GTGAATCACG AAGTGATTCA TAAACTGCCA GGCATCAAAT TAAGCAGTAA 360

GCCGGTCATA AACTGGTGGT TGTAAAAAAA TTCGGTGGAG CGGTAG 406

>(244)C Unique_NoNCBImatch

ATGTTTTGGC GGATGAGAGA CGATTTTCAG CACTGATTCC GGATTTTCGG GAACGAAAGA 60

TTTTACGCTG AGGCAAGGCG GCAAATGAAG GAAAGGAAGA AGCATACAGA AGTATGTGAC 120

TGACTTTACG AATGCAGCCA ACGCAGCATC GGTGTAAAAG ATTCGTTTCT GGCAACAGAA 180

TTTGCCTGGC GGCACTAGCG CGGTGGTCCC ACCTGACCCC ATGCCGAACT CAGAAGTGAA 240

ACGCCGTAGC GCCGATGGTA GTGTGGGGTC TCCCCATGCG AGAGTAGGGA ACTGCCAGGC 300

ATCAAATTAG AAGTATGGCA TTAACTTTCG GGGCAGTTCA CTACTCGACA GAGAGTAGGA 360

CAAAAGCGAA AGCTTTTGAA CGTTGCGAAG CAACGGCCCG AAGGGTGAAT CACGAAGTGA 420

TTCATAAACT GCCAGGCATC AAATTAAGCA GTAAACTGAT GTAAAAATCA GTGGTTGTAA 480

GAGAATTCGG TGGAGCGG 498

>(245)C Unique_NoNCBImatch

ATGTTTTGGC GGATGAGAGA CGATTTTCAG CAATGATTCC GGATTTTCGG GAACGAAAGA 60

TTTTACGCTG AGGCAAGGCG GCAAATGAAG GAAAGGAAGA AGCATACAGA AGTATGTGAC 120

TGACTTTACG AATGCAGCCA ACGCAGCATC GGTGTAAAAG ATTCGTTTCT GGCAACAGAA 180

TTTGCCTGGC GGCACTAGCG CGGTGGTCCC ACCTGACCCC ATGCCGAACT CAGAAGTGAA 240

ACGCCGTAGC GCCGATGGTA GTGTGGGGTC TCCCCATGCG AGAGTAGGGA ACTGCCAGGC 300

ATCAAATTAG AAGTATGGCA TTAACTTTCG GGGCAGTTCA CTACTCGACA GAGAGTAGGA 360

CAAAAGCGAA AGCTTTTGAA CGTTGCGAAG CAACGGCCCG AAGGGTGAAT CACGAAGTGA 420

TTCATAAACT GCCAGGCATC AAATTAAGCA GTAAACTGAT GTAAAAATCA GTGGTTGTAA 480

AAGAATTCGG TGGAGCGG 498

>(246)C Unique_NoNCBImatch

ATGTTTTGGC GGATTGAGAA GACGAACAAT TTTCAGCTCA GATTCAGAGT CCGAAGGATT 60

TTACGCTGAG ACAAGGCGGC AAACGCAGCG ACGGAAGGAG CATACAGAAG TATGTGACTG 120

ACGCTCGCAA GAGCAGCCAA CGCCGTATCA GTGTAAAAGA CACAGGACAG AGCACAAAGA 180

ATTTGCCTGG CGGCACTAGC GCGGTGGTCC CACCTGACCC CATGCCGAAC TCAGAAGTGA 240

AACGCCGTAG CGCCGATGGT AGTGTGGGGT CTCCCCATGC GAGAGTAGGG AACTGCCAGG 300

CATCAAATAG AATGAAAGGC CCTGTCGGAA GACTGGGCCT TTTGTTTTAT CTGTAGTCTG 360

CCGGTGAGCA CTCTCCTGAG GAGGAGAAAT CCGCCGGGAG CGGATTTGAA CGTTGCGCAG 420

CAACGGCCCG GAGGGGTGGC GGGCAGGACG CCCGCCATAA ACTGCCAGGC ATCAAATTAA 480

GCAGTAAGCC GGTCATAGAT CGGTGGTTGT AAAAGAATTC GGTGGAGCGG TAG 533

>(247)X_Dessau_Seftenberg_CP047424_CP038593

TCGGTACGCA GTTTTTTCAG GCTCTCTTTC AGGCTTGGGA TTAGCTTGTC TTTGCTAAGG 60

TTTTCAATCC AGATCTTGGT GGTGATGTAC AGCTCATTAC GCGGTACGCC GCTCTCAGCA 120

ATCGCCTGTC CAACAGCCGC TTCATTATCA TAGATTTGCG CAGTGTCGAC AGCACGGTAG 180

CCCAGTTCAA GCGCCGTTTT CACAGAAGCG ATAACCACGT CGTCTTTCAG GCGAAAAGTA 240

CCTAAACCGA ATGCAGGGAT AGTCATAGAA TTCCTCTTTT TAATCACTAT GTTCGTTAAC 300

TGAGGGGATT ATGACCTTCA GCGCTTTGCA GAAAAAGAGC GAGAAATGCA GAGGATTTTT 360

GCGAATTGAG CAATAATTAA ATGACAGGCA AAGAAAAAGC CCTGAGCTAA TGCTCAGGGC 420

TCTTAATAAG TGGCGGAACG GACGGGACTC GAACCCGCGA CCCCCTGCGT GACAGGCAGG 480

TATTCTAACC AACTGAACTA CCGCTCCACC GAATTCTTTT ACAACCACTG ATTTTTACAT 540

CAGTTTACTG CTTAATTTGA TGCCTGGCAG TTCCCTACTC TCGCATGGGG AGACCCCACA 600

CTACCATCGG CGCTACGGCG TTTCACTTCT GAGTTCGGCA TGGGGTCAGG TGGGACCACC 660

GCGCTACGGC CGCCAGGCAA ATTCTTTAT GCTCTGTACG CAATTCTTA TCGCATCAGC 720

GGCGTTGCCT GCGCTCGTAA ACTCAGTCAC ATACTTCTGT ATGCTCCTTC CTTTCCTTCG 780

CTTGCCGCCT TGCTGCTGCG CAAATTATTG CGTACTCAAC TCTGAATCAG TGCTGAAAAT 840

CATCTCTCAT CCGCCAAAAC ATCTTCGGCG TTGTAAGGTT AAGCCTCACG GTTCATTAGT 900

ACCGGTTAGC TCAACGCATC GCTGCGCTTA CACACCCGGC CTATCAACGT CGTCGTCTTC 960

AACGTTCCTT CAGGAGACTC TAAGTCTCAG GGAGAACTCA TCTCGGGGCA AGTTTCGTGC 1020

TTAGATGCTT TCAGCACTTA TCTCTTCCGC ATTTAGCTAC CGGGCAGTGC CATTGGCATG 1080

ACAACCCGAA CACCAGTGAT GCGTCCACTC CGGTCCTCTC GTACTAGGAG CAGCCCCCCT 1140

CAGTTCTCCA GCGCCCACGG CAGATAGGGA CCGAACTGTC TCACGACGTT CTAAACCCAG 1200

CTCGCGTACC ACTTTAAATG GCGAACAGCC ATACCCTTGG GACCTACTTC AGCCCCAGGA 1260

TGTGATGAGC CGACATCGAG GTGCCAAACA CCGCCGTCGA TATGAACTCT TGGGCGGTAT 1320

CAGCCTGTTA TCCCCGGAGT ACCTTTTATC CGTTGAGCGA TG 1362

>(248)X_Sundsvall_LS483457

TCGGTACGCA GTTTTTTCAG GCTCTCTTTC AGGCTTGGGA TCAGCTTGTC TTTGCTAAGG 60

TTTTCAATCC AGATCTTAGT GGTGATGTAC AGCTCATTAC GCGGTACGCC GCTCTCAGCA 120

ATCGCCTGTC CAACAGCAAC TTCATTATCA TAGATTTGCG CAGTGTCGAC AGCACGGTAG 180

CCCAGTTCAA GCGCCGTTTT CACAGAAGCG ATAACCACGT CGTCTTTCAG GCGAAAAGTA 240

CCTAAACCGA ATGCAGGGAT AGTCATAGAA TTCCTCTTTT TAATCACTAT GTTCGTTAAC 300

TGAGGGGATT ATGACCTTCA GCGCTTTGTA GAAAAAGAGC GAAAAATGCA GAGGATTTTT 360

GCGAATTGAG CAATAATTAA ATGACAGGCA AAGAAAAAGC CCTGAGCTAA TGCTCAGGGC 420

TCTTAATAAG TGGCGGAACG GACGGGACTC GAACCCGCGA CCCCCTGCGT GACAGGCAGG 480

TATTCTAACC AACTGAACTA CCGCTCCACC GAATTCTTTT ACAACCACTG ATTTTTACAT 540

CAGTTTACTG CTTAAATTGA TGCCTGGCAG TTCCCTACTC TCGCATGGGG AGACCCCACA 600

CTACCATCGG CGCTACGGCG TTTCACTTCT GAGTTCGGCA TGGGGTCAGG TGGGACCACC 660

GCGCTAGTGC CGCCAGGCAA ATTCTTTTAT GCTCAGTACG CAATTCTTTA TCGCATCAGC 720

GGCGTTGCCT GCGCTCGTAA ACTCAGTCAC ATACTTCTGT ATGCTCCTTC CTTTCCTTCG 780

CTTGCCGCCT TGCTGCTGCG CAAATTATTG CGTACTCAAC TCTGAATCAG TGCTGAAAAT 840

CGTCTCTCAT CCGCCAAAAC ATCTTCGGCG TTGTAAGGTT AAGCCTCACG GTTCATTAGT 900

ACCGGTTAGC TCAACGCATC GCTGCGCTTA CACACCCGGC CTATCAACGT CGTCGTCTTC 960

AACGTTCCTT CAGGAGACTC TAAGTCTCAG GGAGAACTCA TCTCGGGGCA AGTTTCGTGC 1020

TTAGATGCTT TCAGCACTTA TCTCTTCCGC ATTTAGCTAC CGGGCAGTGC CATTGGCATG 1080

ACAACCCGAA CACCAGTGAT GCGTCCACTC CGGTCCTCTC GTACTAGGAG CAGCCCCCCT 1140

CAGTTCTCCA GCGCCCACGG CAGATAGGGA CCGAACTGTC TCACGACGTT CTAAACCCAG 1200

CTCGCGTACC ACTTTAAATG GCGAACAGCC ATACCCTTGG GACCTACTTC AGCCCCAGGA 1260

TGTGATGAGC CGACATCGAG GTGCCAAACA CCGCCGTCGA TATGAACTCT TGGGCGGTAT 1320

CAGCCTGTTA TCCCCGGAGT ACCTTTTATC CGTTGAGCGA TG 1362

>(249)X Bredeney_Give-CP007533_CP019174

AAGAGATAAG TGCTGAAAGC ATCTAAGCAC GAAACTTGCC CCGAGATGAG TTCTCCCTGA 60

GACTTAGAGT CTCCTGAAGG AACGTTGAAG ACGACGACGT TGATAGGCCG GGTGTGTAAG 120

CGCAGCGATG CGTTGAGCTA ACCGGTACTA ATGAACCGTG AGGCTTAACC TTACAACGCC 180

GAAGATGTTT TGGCGGTTGA GAAGACAACA ATTTTCAGCT CAGATTCAGA GTTGAGTACG 240

CAATAATTTG CGCAGCAGCA AGGCGGCAAG CGAAGGAAAG GAAGGAGCAT ACAGAAGTAT 300

GTGACTGACT TTACGAGCGC AGGCAACGCC GCTGATGCGA TAAAGAATTG CGTACTGAGC 360

ATAAAAGAAT TTGCCTGGCG GCACTAGCGC GGTGGTCCCA CCTGACCCCA TGCCGAACTC 420

AGAAGTGAAA CGCCGTAGCG CCGATGGTAG TGTGGGGTCT CCCCATGCGA GAGTAGGGAA 480

CTGCCAGGCA TCAAATTAAG CAGTAAGCCG GTCATAAACC GGTGGTTGTA AAAGAATCCG 540

GTGGAGCGGT AGTTCAGTTG GTTAGAATAC CTGCCTGTCA CGCAGGGGGT CGCGGGTTCG 600

AGTCCCGTCC GTTCCGCCAC TTATTAAGAG CCCTGAGCAT TAGCTCAGGG CTTTTTCTTT 660

GCCTGTCATT TAATTATTGC TCAATTCGCA AAAATCCTCT GCATTTCTCG CTCTTTTTCT 720

ACAAAGCGCT GAAGGTCATA ATCCCCTCAG TTAACGAACA TAGTGATTAA AAAGAGGAAT 780

TCTATGACTA TCCCTGCATT CGGTTTAGGT ACTTTTCGCC TGAAAGACGA CGTGGTTATC 840

GCTTCTGTGA AAACGGCGCT TGAACTGGGC TACCGTGCTG TCGACACTGC GCAAATCTAT 900

GATAATGAAG TTGCTGTTGG ACAGGCGATT GCTGAGAGCG GCGTACCGCG TAATGAGCTG 960

TACATCACCA CTAAGATCTG GATTGAAAAC CTTAGCAAAG ACAAGCTGAT CCCAAGCCTG 1020

AAAGAGAGCC TGAAAAAACT GCGTACCGAT TATGTCGATC TGACGCTGAT CCACTGGCCA 1080

TCTCCGGGTG ATGCCGTGTC TGTCAAAAAT TATGCAGGCA TTGCTGGAAG CTAAAAAACA 1140

AGGGCTGACT CGCGAAATTG GCATTTCAAC TT 1172

>(250)X Chester_CP019178

TCGGTACGCA GTTTTTTCAG GCTCTCTTTC AGGCTTGGGA TTAGCTTGTC TTTGCTAAGG 60

TTTTCAATCC AGATCTTGGT GGTGATGTAC AGCTCATTAC GCGGTACGCC GCTCTCAGCA 120

ATCGCCTGTC CAACAGCCGC TTCATTATCA TAGATTTGCG CAGTGTCGAC AGCACGGTAG 180

CCTAGTTCAA GCGCCGCTTT AACAGAAGCG ATAACCACGT CGTCTTTCAG GCGAAAAGTA 240

CCTAAACCGA ATGCAGGGAT AGTCATAGAA TTCCTCTTTT TAATCACTAT GTTCGTTAAC 300

TGAGGGGATT ATGACCTTCA GCGCTTTGTA GAAAAAGAGC GAGAAATGCA GAGGATTTTT 360

GCGAATTGAG CAATAATTAA ATGACAGGCA AAGAAAAAGC CCTGAGCTAA TGCTCAGGGC 420

TCTTAATAAG TGGCGGAACG GACGGGACTC GAACCCGCGA CCCCCTGCGT GACAGGCAGG 480

TATTCTAACC AACTGAACTA CCGCTCCACC GAATTTTTTT ACAACCACTG ATTTTTACAT 540

CAGTTTACTG CTTAAATTGA TGCCTGGCAG TTCCCTACTC TCGCATGGGG AGACCCCACA 600

CTACCATCGG CGCTACGGCG TTTCACTTCT GAGTTCGGCA TGGGGTCAGG TGGGACCACC 660

GCGCTACGGC CGCCAGGCAA ATTCTGTTGC CGGAAACGAA TCTTTTACAC CGATGCTGCG 720

TTGGCTGCAT TCGTAAAGTC AGTCACATAC TTCTGTATGC TCCTTCCTTT CCTTCATTTG 780

CCGCCTTGCC TCAGCGTAAA ATCTTTCGTT CCCGAAAATC CGGAATCAGT GCTGAAAATC 840

ATCTCTCAAC CGCCAAAACA TCTTCGGCGT TGTAAGGTTA AGCCTCACGG TTCATTAGTA 900

CCGGTTAGCT CAACGCATCG CTGCGCTTAC ACACCCGGCC TATCAACGTC GTCGTCTTCA 960

ACGTTCCTTC AGGAGACTCT AAGTCTCAGG GAGAACTCAT CTCGGGGCAA GTTTCGTGCT 1020

TAGATGCTTT CAGCACTTAT CTCTTCCGCA TTTAGCTACC GGGCAGTGCC ATTGGCATGA 1080

CAACCCGAAC ACCAGTGATG CGTCCACTCC GGTCCTCTCG TACTAGGAGC AGCCCCCCTC 1140

AGTTCTCCAG CGCCCACGGC AGATAGGGAC CGAACTGTCT CACGACGTTC TAAACCCAGC 1200

TCGCGTACCA CTTTAAATGG CGAACAGCCA TACCCTTGGG ACCTACTTCA GCCCCAGGAT 1260

GTGATGAGCC GACATCGAGG TGCCAAACAC CGCCGTCGAT ATGAACTCTT GGGCGGTATC 1320

AGCCTGTTAT CCCCGGAGTA CCTTTTATCC GTTGAGCGAT G 1361

>(251)X Poona_CP019189

TCGGTACGCA GTTTTTTCAG GCTCTCTTTC AGGCTTGGGA TCAGCTTGTC TTTGCTAAGG 60

TTTTCAATCC AGATCTTAGT GGTGATGTAC AGCTCATTAC GCGGTACGCC GCTCTCAGCA 120

ATCGCCTGTC CAACAGCAAC TTCATTATCA TAGATTTGCG CAGTGTCGAC AGCACGGTAG 180

CCCAGTTCAA GCGCCGTTTT CACAGAAGCG ATAACCACGT CGTCTTTCAG GCGAAAAGTA 240

CCTAAACCGA ATGCAGGGAT AGTCATAGAA TTCCTCTTTT TAATCACTAT GTTCGTTAAC 300

TGAGGGGATT ATGACCTTCA GCGCTTTGTA GAAAAAGAGC GAGAAATGCA GAGGATTTTT 360

GCGAATTGAG CAATAATTAA ATGACAGGCA AAGAAAAAGC CCTGAGCTAA TGCTCAGGGC 420

TCTTAATAAG TGGCGGAACG GACGGGACTC GAACCCGCGA CCCCCTGCGT GACAGGCAGG 480

TATTCTAACC AACTGAACTA CCGCTCCACC GAATTTCTTT GTCACAACCC GGATTTTCAT 540

CCCGGTTTAC TGCAATTTGA TGCCTGGCAG TTCCCTACTC TCGCATGGGG AGACCCCACA 600

CTACCATCGG CGCTACGGCG TTTCACTTCT GAGTTCGGCA TGGGGTCAGG TGGGACCACC 660

GCGCTAGTGC CGCCAGGCAA ATTCTTTTAT GCTCAGTACG CAATTCTTTA TCGCATCAGC 720

GGCGTTGCCT GCGCTCGTAA ACTCAGTCAC ATACTTCTGT ATGCTCCTTC CTTTCCTTCG 780

CTTGCCGCCT TGCTGCTGCG CAAATTATTG CGTACTCAAC TCTGAATCAG TGCTGAAAAT 840

CGTCTCTCAT CCGCCAAAAC ATCTTCGGCG TTGTAAGGTT AAGCCTCACG GTTCATTAGT 900

ACCGGTTAGC TCAACGCATC GCTGCGCTTA CACACCCGGC CTATCAACGT CGTCGTCTTC 960

AACGTTCCTT CAGGAGACCT AAAGTCTCAG GGAGAACTCA TCTCGGGGCA AGTTTCGTGC 1020

TTAGATGCTT TCAGCACTTA TCTCTTCCGC ATTTAGCTAC CGGGCAGTGC CATTGGCATG 1080

ACAACCCGAA CACCAGTGAT GCGTCCACTC CGGTCCTCTC GTACTAGGAG CAGCCCCCCT 1140

CAGTTCTCCA GCGCCCACGG CAGATAGGGA CCGAACTGTC TCACGACGTT CTAAACCCAG 1200

CTCGCGTACC ACTTTAAATG GCGAACAGCC ATACCCTTGG GACCTACTTC AGCCCCAGGA 1260

TGTGATGAGC CGACATCGAG GTGCCAAACA CCGCCGTCGA TATGAACTCT TGGGCGGTAT 1320

CAGCCTGTTA TCCCCGGAGT ACCTTTTATC CGTTGAGCGA TG 1362

>(252)X Heidelberg_CP051358

TCGGTACGCA GTTTTTTCAG GCTCTCTTTC AGGCTTGGGA TTAGCTTGTC TTTGCTAAGG 60

TTTTCAATCC AGATCTTGGT GGTGATGTAC AGCTCGCTAC GTGGTACGCC GCTCTCAGTA 120

ATCGCCTGTC CAACAGCCGC TTCATTATCA TAGATTTGCG CAGTGTCGAC AGCACGGTAG 180

CCCAGTTCAA GCGCCGTTTT CACAGAAGCG ATAACCACGT CGTCTTTCAG GCGAAAAGTA 240

CCTAAACCGA ATGCAGGGAT AGTCATAGAA TTCCTCTTTT TAATCACTAT GTTCGTTAAC 300

TGAGGGGATT ATGACCTTCA GCGCTTTGTA GAAAAAGAGC GAGAAATGCA GAGGATTTTT 360

GCGAATTGAG CAATAATTAA ATCGCAGGCA AAGAAAAAGC CCTGAGCTAA TGCTCAGGGC 420

TCTTAATAAG TGGCGGAACG GACGGGACTC GAACCCGCGA CCCCCTGCGT GACAGGCAGG 480

TATTCTAACC AACTGAACTA CCGCTCCACC GAATTTCTTT GTCACAACCC GGATTTTCAT 540

CCCGGTTTAC TGCAATTTGA TGCCTGGCAG TTTATGAATC ACTTCGTGAT TCACCCTTCG 600

GGCCGTTGCT TCGCAACGTT CAAAAGCTTT CGCTTTTGTC CTACTCTCTG TCGAGTAGTG 660

AACTGCCCCG AAAGTTAATG CCATACTTCT AATTTGATGC CTGGCAGTTC CCTACTCTCG 720

CATGGGGAGA CCCCACACTA CCATCGGCGC TACGGCGTTT CACTTCTGAG TTCGGCATGG 780

GGTCAGGTGG GACCACCGCG CTAGTGCCGC CAGGCAAATT CTGTTATCAG ACCGTTTTTA 840

CGTTCTGATG TAATCTGAAT CAGTGCTGAA AATCGTCTCT CAACCGCCAA AACATCTTCG 900

GCGTTGTAAG GTTAAGCCTC ACGGTTCATT AGTACCGGTT AGCTCAACGC ATCGCTGCGC 960

TTACACACCC GGCCTATCAA CGTCGTCGTC TTCAACGTTC CTTCAGGAGA CTCTAAGTCT 1020

CAGGGAGAAC TCATCTCGGG GCAAGTTTCG TGCTTAGATG CTTTCAGCAC TTATCTCTTC 1080

CGCATTTAGC TACCGGGCAG TGCCATTGGC ATGACAACCC GAACACCAGT GATGCGTCCA 1140

CTCCGGTCCT CTCGTACTAG GAGCAGCCCC CCTCAGTTCT CCAGCGCCCA CGGCAGATAG 1200

GGACCGAACT GTCTCACGAC GTTCTAAACC CAGCTCGCGT ACCACTTTAA ATGGCGAACA 1260

GCCATACCCT TGGGACCTAC TTCAGCCCCA GGATGTGATG AGCCGACATC GAGGTGCCAA 1320

ACACCGCCGT CGATATGAAC TCTTGGGCGG TATCAGCCTG TTATCCCCGG AGTACCTTTT 1380

ATCCGTTGAG CGATG 1395

>(253)X Bergen_CP019405

TCGGTACGCA GTTTTTTCAG GCTCTCTTTC AGGCTTGGGA TTAGTTTGTC TTTGCTAAGG 60

TTTTCAATCC AGATCTTGGT GGTGATGTAC AGCTCATTAC GTGGTACACC GCTTTCAGCA 120

ATCGCCTGTC CAACAGCCGC TTCATTATCA TAGATCTGTG CAGTGTCGAC AGCACGGTAG 180

CCTAGTTCAA GCGCCGTTTT AACAGAAGCG ATAACCACGT CGTCTTTCAG GCGAAAAGTA 240

CCTAAACCGA ATGCAGGGAT AGTCATAGAA TTCCTCTTTT TAATCACTAT GTTCGTTAAC 300

TGAGGGGATT ATGACCTTCA GCGCTTTGCA GAAAAAGAGC GAGAAATGCA GAGGATTTTT 360

GCGAATTGAG CAATAATTAA ATGACATGCA AAGAAAAAGC CCTGAGCTAA TGCTCAGGGC 420

TCTTAATAAG TGGCGGAACG GACGGGACTC GAACCCGCGA CCCCCTGCGT GACAGGCAGG 480

TATTCTAACC AACTGAACTA CCGCTCCACC GAATTCTTTT ACAACCACTG ATTTTTACAT 540

CAGTTTACTG CTTAATTTGA TGCCTGGCAG TTTATGAATC ACTTCGTGAT TCACCCTTCG 600

GGCCGTTGCT TCGCAACGTT CAAAAGCTTT CGCTTTTGTC CTACTCTCTG TCGAGTAGTG 660

AACTGCCCCG AAAGTTAATG CCATACTTCT AATTTGATGC CTGGCAGTTC CCTACTCTCG 720

CATGGGGAGA CCCCACACTA CCATCGGCGC TACGGCGTTT CACTTCTGAG TTCGGCATGG 780

GGTCAGGTGG GACCACCGCG CTAGTGCCGC CAGGCAAATT CTTTGTGCTC TGTCCTGTGT 840

CTTTTACACT GATACGGCGT TGGCTGCTCT TGCGAGCGTC AGTCACATAC TTCTGTATGC 900

TCCTTCCGTC GCTGCGTTTG CCGCCTTGTC TCAGCGTAAA ATCCTTCGGA CTCTGAATCT 960

GAGCTGAAAA TTGTTGTCTT CTCAATCCGC CAAAACATCT TCGGCGTTGT AAGGTTAAGC 1020

CTCACGGTTC ATTAGTACCG GTTAGCTCAA CGCATCGCTG CGCTTACACA CCCGGCCTAT 1080

CAACGTCGTC GTCTTCAACG TTCCTTCAGG AGACTCTAAG TCTCAGGGAG AACTCATCTC 1140

GGGGCAAGTT TCGTGCTTAG ATGCTTTCAG CACTTATCTC TTCCGCATTT AGCTACCGGG 1200

CAGTGCCATT GGCATGACAA CCCGAACACC AGTGATGCGT CCACTCCGGT CCTCTCGTAC 1260

TAGGAGCAGC CCCCCTCAGT TCTCCAGCGC CCACGGCAGA TAGGGACCGA ACTGTCTCAC 1320

GACGTTCTAA ACCCAGCTCG CGTACCACTT TAAATGGCGA ACAGCCATAC CCTTGGGACC 1380

TACTTCAGCC CCAGGATGTG ATGAGCCGAC ATCGAGGTGC CAAACACCGC CGTCGATATG 1440

AACTCTTGGG CGGTATCAGC CTGTTATCCC CGGAGTACCT TTTATCCGTT GAGCGATG 1498

>(254)X Manhattan_CP022497

CATCGCTCAA CGGATAAAAG GTACTCCGGG GATAACAGGC TGATACCGCC CAAGAGTTCA 60

TATCGACGGC GGTGTTTGGC ACCTCGATGT CGGCTCATCA CATCCTGGGG CTGAAGTAGG 120

TCCCAAGGGT ATGGCTGTTC GCCATTTAAA GTGGTACGCG AGCTGGGTTT AGAACGTCGT 180

GAGACAGTTC GGTCCCTATC TGCCGTGGGC GCTGGAGAAC TGAGGGGGGC TGCTCCTAGT 240

ACGAGAGGAC CGGAGTGGAC GCATCACTGG TGTTCGGGTT GTCATGCCAA TGGCACTGCC 300

CGGTAGCTAA ATGCGGAAGA GATAAGTGCT GAAAGCATCT AAGCACGAAA CTTGCCCCGA 360

GATGAGTTCT CCCTGAGACT TAGAGTCTCC TGAAGGAACG TTGAAGACGA CGACGTTGAT 420

AGGCCGGGTG TGTAAGCGCA GCGATGCGTT GAGCTAACCG GTACTAATGA ACCGTGAGGC 480

TTAACCTTAC AACGCCGAAG ATGTTTTGGC GGATTGAGAA GACAACAATT TTCAGCTCAG 540

ATTCAGAGTC CGAAGGATTT TACGCTGAGA CAAGGCGGCA AACGCAGCGA CGGAAGGAGC 600

ATACAGAAGT ATGTGACTGA CGCTCGCAAG AGCAGCCAAC GCCGTATCAG TGTAAAAGAC 660

ACAGGACAGA GCACAAAGAA TTTGCCTGGC GGCACTAGCG CGGTGGTCCC ACCTGACCCC 720

ATGCCGAACT CAGAAGTGAA ACGCCGTAGC GCCGATGGTA GTGTGGGGTC TCCCCATGCG 780

AGAGTAGGGA ACTGCCAGGC ATCAAATTAG AAGTATGGCA TTAACTTTCG GGGCAGTTCA 840

CTACTCGACA GAGAGTAGGA CAAAAGCGAA AGCTTTTGAA CGTTGCGAAG CAACGGCCCG 900

AAGGGTGAAT CACGAAGTGA TTCATAAACT GCCAGGCATC AAATTAAGCA GTAAACTGAT 960

GTAAAAATCA GTGGTTGTAA AAGAATTCGG TGGAGCGGTA GTTCAGTTGG TTAGAATACC 1020

TGCCTGTCAC GCAGGGGGTC GCGGGTTCGA GTCCCGTCCG TTCCGCCACT TATTAAGAGC 1080

CCTGAGCATT AGCTCAGGGC TTTTTCTTTT CCTGTCATTT AATTATTGCT CAATTCGCAA 1140

AAATCCTCTG CATTTCTCGC TCTTTTTCTG CAAAGCGCTG AAGGTCATAA TTCCCTCAGT 1200

TAACGAACAT AGTGATTAAA AAGAGGAATT CTATGACTAT CCCTGCATTC GGTTTAGGTA 1260

CTTTTCGCCT GAAAGACGAC GTGGTTATCG CTTCTGTTAA AACGGCGCTT GAACTGGGCT 1320

ACCGTGCTGT CGACACTGCG CAAATCTATG ATAATGAAGC GGCTGTTGGA CAGGCGATTG 1380

CTGAGAGCGG CGTACCGCGT AGCGAGCTGT ACATCACCAC CAAGATCTGG ATTGAAAACC 1440

TTAGCAAAGA CAAGCTAATC CCAAGCCTGA AAGAGAGCCT GAAAAAACTG CGTACCGA 1498

>(255)X Inverness_CP075132

TGATCGCAGC AGATGAAGTG CCGGTATTAA CAACCAGCTC AGCCGAACTC GCCCGACAGC 60

AGCTTAGCGC ATTAAATGGC TGTAGCTGGC TTCCTGTAAA CTGGGCAAAT GAAAAGGGCG 120

GATTACATAC CGTTGCCGAC AGCGCAACGC TTTCACGGCC ACTATACGCT ATTTGGCTGC 180

AAAACAGCGA TAAATACTCG CTGATCTGCG ATCTGTTAAA AACGGATGTG CTGGATGAAC 240

AATGAAAATG ATGAAAAGAA AAAGAAGGGA ACTTACGATT ACCTGATCAG ATAATCAGGT 300

AATTTTAGGC AAAAAAAAAT CCTTAGCAGA TGCTAAGGAT TATTTCTGGC AGGGGCGGAG 360

AGACTCGAAC TCCCAACACC CGGTTTTGGA GACCGGTGCT CTACCAATTG AACTACGCCC 420

CTAATTAGGG TGGCGGAACG GACGGGACTC GAACCCGCGA CCCCCTGCGT GACAGGCAGG 480

TATTCTAACC AACTGAACTA CCGCTCCACC GAATTCTTTT ACAACCACTG ATTTTTACAT 540

CAGTTTACTG CTTAATTTGA TGCCTGGCAG TTCCCTACTC TCGCATGGGG AGACCCCACA 600

CTACCATCGG CGCTACGGCG TTTCACTTCT GAGTTCGGCA TGGGGTCAGG TGGGACCACC 660

GCGCTAGTGC CGCCAGGCAA ATTCTGTTAC CGGAAACGAA TCTTTTACAC CGATGCTGCG 720

TTGGCTGCAT TCGCAAAGTC AGTCACATAC TTCTGTATGC TCCTTCCTTT CCTTCATTTG 780

CCGCCTTGCC TCAGCGTAAA ATCTTTCGTT CCCGAAAATC CGGAATCAGT GCTGAAAATC 840

GTCTCTCATC CGCCAAAACA TCTTCGGCGT TGTAAGGTTA AGCCTCACGG TTCATTAGTA 900

CCGGTTAGCT CAACGCATCG CTGCGCTTAC ACACCCGGCC TATCAACGTC GTCGTCTTCA 960

ACGTTCCTTC AGGAGACTCT AAGTCTCAGG GAGAACTCAT CTCGGGGCAA GTTTCGTGCT 1020

TAGATGCTTT CAGCACTTAT CTCTTCCGCA TTTAGCTACC GGGCAGTGCC ATTGGCATGA 1080

CAACCCGAAC ACCAGTGATG CGTCCACTCC GGTCCTCTCG TACTAGGAGC AGCCCCCCTC 1140

AGTTCTCCAG CGCCCACGGC AGATAGGGAC CGAACTGTCT CACGACGTTC TAAACCCAGC 1200

TCGCGTACCA CTTTAAATGG CGAACAGCCA TACCCTTGGG ACCTACTTCA GCCCCAGGAT 1260

GTGATGAGCC GACATCGAGG TGCCAAACAC CGCCGTCGAT ATGAACTCTT GGGCGGTATC 1320

AGCCTGTTAT CCCCGGAGTA CCTTTTATCC GTTGAGCGAT 1360

>(256)X Othmarschen_CP066260

TCGGTACGCA GTTTTTTCAG GCTCTCTTTC AGGCTTGGGA TTAGCTTGTC TTTGCTAAGG 60

TTTTCAATCC AGATCTTGGT GGTGATGTAC AGCTCGCTAC GCGGTACGCC GCTCTCAGCA 120

ATCGCCTGTC CAACAGCCGC TTCATTATCA TAGATTTGCG CAGTGTCGAC AGCACGGTAG 180

CCCAGTTCAA GCGCCGTTTT CACAGAAGCG ATAACCACGT CGTCTTTCAG GCGAAAAGTA 240

CCTAAACCGA ATGCAGGGAT AGTCATAGAA TTCCTCTTTT TAATCACTAT GTTCGTTAAC 300

TGAGGGGATT ATGACCTTCA GCGCTTTGCA GAAAAAGAGC GAGAAATGCA GAGGATTTTT 360

GCGAATTGAG CAATAATTAA ATGACATGCA AAGAAAAAGC CCTGAGCTAA TGCTCAGGGC 420

TCTTAATAAG TGGCGGAACG GACGGGACTC GAACCCGCGA CCCCCTGCGT GACAGGCAGG 480

TATTCTAACC AACTGAACTA CCGCTCCACC GAATTCTTTT ACAACCACTG ATTTTTACAT 540

CAGTTTACTG CTTAATTTGA TGCCTGGCAG TTTATGAATC ACTTCGTGAT TCACCCTTCG 600

GGCCGTTGCT TCGCAACGTT CAAAAGCTTT CGCTTTTGTC CTACTCTCTG TCGAGTAGTG 660

AACTGCCCCG AAAGTTAATG CCATACTTCT AATTTGATGC CTGGCAGTTC CCTACTCTCG 720

CATGGGGAGA CCCCACACTA CCATCGGCGC TACGGCGTTT CACTTCTGAG TTCGGCATGG 780

GGTCAGGTGG GACCACCGCG CTAGTGCCGC CAGGCAAATT CTTTGTGCTC TGTACGCAAT 840

TCTTTATCGC ATCAGCGGCG TTGCCTGCGC TCGTAAACTC AGTCACATAC TTCTGTATGC 900

TCCTTCCTTT CCTTCGCTTG CCGCCTTGCT GCTGCGCAAA TTATTGCGTA CTCAACTCTG 960

AATCAGTGCT GAAAATCATC TCTCATCCGC CAAAACATCT TCGGCGTTGT AAGGTTAAGC 1020

CTCACGGTTC ATTAGTACCG GTTAGCTCAA CGCATCGCTG CGCTTACACA CCCGGCCTAT 1080

CAACGTCGTC GTCTTCAACG TTCCTTCAGG AGACTCTAAG TCTCAGGGAG AACTCATCTC 1140

GGGGCAAGTT TCGTGCTTAG ATGCTTTCAG CACTTATCTC TTCCGCATTT AGCTACCGGG 1200

CAGTGCCATT GGCATGACAA CCCGAACACC AGTGATGCGT CCACTCCGGT CCTCTCGTAC 1260

TAGGAGCAGC CCCCCTCAGT TCTCCAGCGC CCACGGCAGA TAGGGACCGA ACTGTCTCAC 1320

GACGTTCTAA ACCCAGCTCG CGTACCACTT TAAATGGCGA ACAGCCATAC CCTTGGGACC 1380

TACTTCAGCC CCAGGATGTG ATGAGCCGAC ATCGAGGTGC CAAACACCGC CGTCGATATG 1440

AACTCTTGGG CGGTATCAGC CTGTTATCCC CGGAGTACCT TTTATCCGTT GAGCGATG 1498

>(257)X Choleraesuis-var-Kunzendorf_CP075031

TCGGTACGCA GTTTTTTCAG GCTCTCTTTC AGGCTTGGGA TTAGTTTGTC TTTGCTAAGG 60

TTTTCAATCC AGATCTTGGT GGTGATGTAC AGCTCATTAC GTGGTACACC GCTTTCAGCA 120

ATCGCCTGTC CAACAGCCGC TTCATTATCA TAGATCTGTG CAGTGTCGAC AGCACGGTAG 180

CCCAGTTCAA GCGCCGTTTT AACAGAAGCG ATAACCACGT CGTCTTTCAG GCGAAAAGTA 240

CCTAAACCGA ATGCAGGGAT AGTCATAGAA TTCCTCTTTT TAATCACTAT GTTCGTTAAC 300

TGAGGGAATT ATGACCTTCA GCGCTTTGTA GAAAAAGAGC GAGAAATGCA GAGGATTTTT 360

GCGAATTGAG CAATAATTAA ATGACAGGCA AAGAAAAAGC CCTGAGCTAA TGCTCAGGGC 420

TCTTAATAAG TGGCGGAACG GACGGGACTC GAACCCGCGA CCCCCTGCGT GACAGGCAGG 480

TATTCTAACC AACTGAACTA CCGCTCCACC GAATTTCTTT GTCACAATCC GGATTTTCAT 540

CCCGGTTTAC TGCAATTTGA TGCCTGGCAG TTTATGAATC ACTTCGTGAT TCACCCTTCG 600

GGCCGTTGCT TCGCAACGTT CAAAAGCTTT CGCTTTTGTC CTACTCTCTG TCGAGTAGTG 660

AACTGCCCCG AAAGTTAATA CCATACTTCT AATTTGATGC CTGGCAGTTC CCTACTCTCG 720

CATGGGGAGA CCCCACACTA CCATCGGCGC TACGGCGTTT CACTTCTGAG TTCGGCATGG 780

GGTCAGGTGG GACCACCGCG CTAGTGCCGC CAGGCAAATT CTTTTGTGCT CAGTACGCAA 840

TTCTTTATCG CATCAGCGGC GTTGCCCGCG CTCGTAAAGT CAGTCACATA CTTCTGTATG 900

CTCCTTCCTT TCCTTCGCTT GCCGCCTTGC TGCTGCGCAA ATTATTGCGT ACTCAACTCT 960

GAATCAGTGC TGAAAATTGT CTCTCATCCG CCAAAACATC TTCGGCGTTG TAAGGTTAAG 1020

CCTCACGGTT CATTAGTACC GGTTAGCTCA ACGCATCGCT GCGCTTACAC ACCCGGCCTA 1080

TCAACGTCGT CGTCTTCAAC GTTCCTTCAG GAGACTCTAA GTCTCAGGGA GAACTCATCT 1140

CGGGGCAAGT TTCGTGCTTA GATGCTTTCA GCACTTATCT CTTCCGCATT TAGCTACCGG 1200

GCAGTGCCAT TGGCATGACA ACCCGAACAC CAGTGATGCG TCCACTCCGG TCCTCTCGTA 1260

CTAGGAGCAG CCCCCCTCAG TTCTCCAGCG CCCACGGCAG ATAGGGACCG AACTGTCTCA 1320

CGACGTTCTA AACCCAGCTC GCGTACCACT TTAAATGGCG AACAGCCATA CCCTTGGGAC 1380

CTACTTCAGC CCCAGGATGT GATGAGCCGA CATCGAGGTG CCAAACACCG CCGTCGATAT 1440

GAACTCTTGG GCGGTATCAG CCTGTTATCC CCGGAGTACC TTTTATCCGT TGAGCGATG 1499

>(258)X Nitra_CP019416

CATCGCTCAA CGGATAAAAG GTACTCCGGG GATAACAGGC TGATACCGCC CAAGAGTTCA 60

TATCGACGGC GGTGTTTGGC ACCTCGATGT CGGCTCATCA CATCCTGGGG CTGAAGTAGG 120

TCCCAAGGGT ATGGCTGTTC GCCATTTAAA GTGGTACGCG AGCTGGGTTT AGAACGTCGT 180

GAGACAGTTC GGTCCCTATC TGCCGTGGGC GCTGGAGAAC TGAGGGGGGC TGCTCCTAGT 240

ACGAGAGGAC CGGAGTGGAC GCATCACTGG TGTTCGGGTT GTCATGCCAA TGGCACTGCC 300

CGGTAGCTAA ATGCGGAAGA GATAAGTGCT GAAAGCATCT AAGCACGAAA CTTGCCCCGA 360

GATGAGTTCT CCCTGAGACT TAGAGTCTCC TGAAGGAACG TTGAAGACGA CGACGTTGAT 420

AGGCCGGGTG TGTAAGCGCA GCGATGCGTT GAGCTAACCG GTACTAATGA ACCGTGAGGC 480

TTAACCTTAC AACGCCGAAG ATGTTTTGGC GGATGAGAAG ACGATTTTCA GCAGTGATTC 540

AGAGTTGAGT ACGCAATACT TTGCGCAGCA GCAAGGCGGC AAGCGAAGGA AAGGAAGGAG 600

CATACAGAAG TATGTGACTG AGTTTACGAG CGCAGGCAAC GCCGCTGATG CGATAAAGAA 660

TTGCGTACAG AGCACAAAGA ATTTGCCTGG CGGCACTAGC GCGGTGGTCC CACCTGACCC 720

CATGCCGAAC TCAGAAGTGA AACGCCGTAG CGCCGATGGT AGTGTGGGGT CTCCCCATGC 780

GAGAGTAGGG AACTGCCAGG CATCAAATTA GAAGTATGGC ATTAACTTTC GGGGCAGTTC 840

ACTACTCGAC AGAGAGTAGG ACAAAAGCGA AAGCTTTTGA ACGTTGCGAA GCAACGGCCC 900

GAAGGGTGAA TCACGAAGTG ATTCATAAAC TGCCAGGCAT CAAATTGCAG TAAACCGGGA 960

TGAAAATCCG GGTTGTGACA AAGAAATTCG GTGGAGCGGT AGTTCAGTTG GTTAGAATAC 1020

CTGCCTGTCA CGCAGGGGGT CGCGGGTTCG AGTCCCGTCC GTTCCGCCAC TTATTAAGAG 1080

CCCTGAGCAT TAGCTCAGGG CTTTTTCTTT GCCTGCGATT TAATTATTGC TCAATTCGCA 1140

AAAATCCTCT GCATTTCTCG CTCTTTTTCT ACAAAGCGCT GAAGGTCATA ATCCCCTCAG 1200

TTAACGAACA TAGTGATTAA AAAGAGGAAT TCTATGACTA TCCCTGCATT CGGTTTAGGT 1260

ACTTTTCGCC TGAAAGACGA CGTGGTTATC GCTTCTGTGA AAACGGCGCT TGAACTGGGC 1320

TACCGTGCTG TCGACACTGC GCAAATCTAT GATAATGAAG CGGCTGTTGG ACAGGCGATT 1380

ACTGAGAGCG GCGTACCACG TAGCGAGCTG TACATCACCA CCAAGATCTG GATTGAAAAC 1440

CTTAGCAAAG ACAAGCTAAT CCCAAGCCTG AAAGAGAGCC TGAAAAAACT GCGTACCGA 1499

>(259)X Mikawasima_CP034713

TCGGTACGCA GTTTTTTCAG GCTCTCTTTC AGGCTTGGGA TTAGCTTGTC TTTGCTAAGG 60

TTTTCAATCC AGATCTTGGT GGTGATGTAC AGCTCATTAC GCGGTACGCC GCTCTCAGCA 120

ATCGCCTGTC CAACAGCCGC TTCATTATCA TAGATTTGCG CAGTGTCGAC AGCACGGTAG 180

CCTAGTTCAA GCGCCGTTTT AACAGAAGCG ATAACCACGT CGTCTTTCAG GCGAAAAGTA 240

CCTAAACCGA ATGCAGGGAT AGTCATAGAA TTCCTCTTTT TAATCACTAT GTTCGTTAAC 300

TGAGGGGATT ATGACCTTCA GCGCTTTGTA GAAAAAGAGC GAGAAATGCA GAGGATTTTT 360

GCGAATTGAG CAATAATTAA ATCGCAGGCA AAGAAAAAGC CCTGAGCTAA TGCTCAGGGC 420

TCTTAATAAG TGGCGGAACG GACGGGACTC GAACCCGCGA CCCCCTGCGT GACAGGCAGG 480

TATTCTAACC AACTGAACTA CCGCTCCACC GAATTCTTTT ACAACCACTG ATTTTTACAT 540

CAGTTTACTG CTTAATTTGA TGCCTGGCAG TTTATGAATC ACTTCGTGAT TCACCCTTCG 600

GGCCGTTGCT TCGCAACGTT CAAAAGCTTT CGCTTTTGTC CTACTCTCTG TCGAGTAGTG 660

AACTGCCCCG AAAGTTAATG CCATACTTCT AATTTGATGC CTGGCAGTTC CCTACTCTCG 720

CATGGGGAGA CCCCACACTA CCATCGGCGC TACGGCGTTT CACTTCTGAG TTCGGCATGG 780

GGTCAGGTGG GACCACCGCG CTAGTGCCGC CAGGCAAATT CTGTTACCGG AAACGAATCT 840

TTTACACCGA TGCTGCGTTG GCTGCATTCG CAAAGTCAGT CACATACTTC TGTATGCTCC 900

TTCCTTTCCT TCATTTGCCG CCTTGCCTCA GCGTAAAATC TTTCGTTCCC GAAAATCCGG 960

AATCAGTGCT GAAAATCGTC TCTCATCCGC CAAAACATCT TCGGCGTTGT AAGGTTAAGC 1020

CTCACGGTTC ATTAGTACCG GTTAGCTCAA CGCATCGCTG CGCTTACACA CCCGGCCTAT 1080

CAACGTCGTC GTCTTCAACG TTCCTTCAGG AGACCTAAAG TCTCAGGGAG AACTCATCTC 1140

GGGGCAAGTT TCGTGCTTAG ATGCTTTCAG CACTTATCTC TTCCGCATTT AGCTACCGGG 1200

CAGTGCCATT GGCATGACAA CCCGAACACC AGTGATGCGT CCACTCCGGT CCTCTCGTAC 1260

TAGGAGCAGC CCCCCTCAGT TCTCCAGCGC CCACGGCAGA TAGGGACCGA ACTGTCTCAC 1320

GACGTTCTAA ACCCAGCTCG CGTACCACTT TAAATGGCGA ACAGCCATAC CCTTGGGACC 1380

TACTTCAGCC CCAGGATGTG ATGAGCCGAC ATCGAGGTGC CAAACACCGC CGTCGATATG 1440

AACTCTTGGG CGGTATCAGC CTGTTATCCC CGGAGTACCT TTTATCCGTT GAGCGATG 1498

>(260)X Sandiego_CP075040

TCGGTACGCA GTTTTTTCAG GCTCTCTTTC AGGCTTGGGA TCAGCTTGTC TTTGCTAAGG 60

TTTTCAATCC AGATCTTAGT GGTGATGTAC AGCTCATTAC GCGGTACGCC GCTCTCAGCA 120

ATCGCCTGTC CAACAGCAAC TTCATTATCA TAGATTTGCG CAGTGTCGAC AGCACGGTAG 180

CCCAGTTCAA GCGCCGTTTT CACAGAAGCG ATAACCACGT CGTCTTTCAG GCGAAAAGTA 240

CCTAAACCGA ATGCAGGGAT AGTCATAGAA TTCCTCTTTT TAATCACTAT GTTCGTTAAC 300

TGAGGGGATT ATGACCTTCA GCGCTTTGTA GAAAAAGAGC GAGAAATGCA GAGGATTTTT 360

GCGAATTGAG CAATAATTAA ATGACAGGCA AAGAAAAAGC CCTGAGCTAA TGCTCAGGGC 420

TCTTAATAAG TGGCGGAACG GACGGGACTC GAACCCGCGA CCCCCTGCGT GACAGGCAGG 480

TATTCTAACC AACTGAACTA CCGCTCCACC GAATTTTTTT ACAACCACCA GTTTATGACC 540

GGCTTACTGC TTAATTTGAT GCCTGGCAGT TTATCAATCA CTTCGTGATT CACCCTTCGG 600

GCCGTTGCTT CGCAACGTTC AAAAGCTTTC GCTTTTGTCC TACTCTCTGT CGAGTAGTGA 660

ACTGCCCCGA AAGTTAATGC CATACTTCTA ATTTGATGCC TGGCAGTTCC CTACTCTCGC 720

ATGGGGAGAC CCCACACTAC CATCGGCGCT ACGGCGTTTC ACTTCTGAGT TCGGCATGGG 780

GTCAGGTGGG ACCACCGCGC TAGTGCCGCC AGGCAAATTC TGTTGCCAGA AACGAATCTC 840

TTATCTTTCG TTCCCGAAAA TCCGGAATCA GTGCTGAAAA TCGTCTCTCA ATCCGCCAAA 900

ACATCTTCGG CGTTGTAAGG TTAAGCCTCA CGGTTCATTA GTACCGGTTA GCTCAACGCA 960

TCGCTGCGCT TACACACCCG GCCTATCAAC GTCGTCGTCT TCAACGTTCC TTCAGGAGAC 1020

CTAAAGTCTC AGGGAGAACT CATCTCGGGG CAAGTTTCGT GCTTAGATGC TTTCAGCACT 1080

TATCTCTTCC GCATTTAGCT ACCGGGCAGT GCCATTGGCA TGACAACCCG AACACCAGTG 1140

ATGCGTCCAC TCCGGTCCTC TCGTACTAGG AGCAGCCCCC CTCAGTTCTC CAGCGCCCAC 1200

GGCAGATAGG GACCGAACTG TCTCACGACG TTCTAAACCC AGCTCGCGTA CCACTTTAAA 1260

TGGCGAACAG CCATACCCTT GGGACCTACT TCAGCCCCAG GATGTGATGA GCCGACATCG 1320

AGGTGCCAAA CACCGCCGTC GATATGAACT CTTGGGCGGT ATCAGCCTGT TATCCCCGGA 1380

GTACCTTTTA TCCGTTGAGC GATG 1404

>(261)X Mbandaka_CP019183

TCGGTACGCA GTTTTTTCAG GCTCTCTTGC AGGCTTGGGA TTAGTTTGTC TTTGCTAAGG 60

TTTTCAATCC AGATCTTGGT GGTGATGTAC AGCTCGCTAC GTGGTACGCC GCTCTCAGCA 120

ATCGCCTGTC CAACAGCCGC TTCATTATCA TAGATTTGCG CAGTGTCGAC AGCACGGTAG 180

CCCAGTTCAA GCGCCGTTTT CACAGAAGCG ATAACCACGT CGTCTTTCAG GCGAAAAGTA 240

CCTAAACCGA ATGCAGGGAT AGTCATAGAA TTCCTCTTTT TAATCACTAT GTTCGTTAAC 300

TGAGGGGATT ATGACCTTCA GCGCTTTGTA GAAAAAGAGC GAGAAATGCA GAGGATTTTT 360

GCGAATTGAG CAATAATTAA ATGACAGGCA AAGAAAAAGC CCTGAGCTAA TGCTCAGGGC 420

TCTTAATAAG TGGCGGAACG GACGGGACTC GAACCCGCGA CCCCCTGCGT GACAGGCAGG 480

TATTCTAACC AACTGAACTA CCGCTCCACC GAATTCTTTT ACAACCACTG ATTTTTACAT 540

CAGTTTACTG CTTAATTTGA TGCCTGGCAG TTTATGAATC ACTTCGTGAT TCACCCTTCG 600

GGCCGTTGCT TCGCAACGTT CAAAAGCTTT CGCTTTTGTC CTACTCTCTG TCGAGTAGTG 660

AACTGCCCCG AAAGTTAATG CCATACTTCT AATTTGATGC CTGGCAGTTC CCTACTCTCG 720

CATGGGGAGA CCCCACACTA CCATCGGCGC TACGGCGTTT CACTTCTGAG TTCGGCATGG 780

GGTCAGGTGG GACCACCGCG CTAGTGCCGC CAGGCAAATT CTGTTACCGG AAACGAATCT 840

TTTACACCGA TGCTGCGTTG GCTGCATTCG CAAAGTCAGT CACATACTTC TGTATGCTCC 900

TTCCTTTCCT TCATTTGCCG CCTTGCCTCA GCGTAAAATC TTTCGTTCCC GAAAATCCGG 960

AATCAGTGCT GAAAATCGTC TCTCATCCGC CAAAACAGCT TCGGCGTTGT AAGGTTAAGC 1020

CTCACGGTTC ATTAGTACCG GTTAGCTCAA CGCATCGCTG CGCTTACACA CCCGGCCTAT 1080

CAACGTCGTC GTCTTCAACG TTCCTTCAGG AGACTCTAAG TCTCAGGGAG AACTCATCTC 1140

GGGGCAAGTT TCGTGCTTAG ATGCTTTCAG CACTTATCTC TTCCGCATTT AGCTACCGGG 1200

CAGTGCCATT GGCATGACAA CCCGAACACC AGTGATGCGT CCACTCCGGT CCTCTCGTAC 1260

TAGGAGCAGC CCCCCTCAGT TCTCCAGCGC CCACGGCAGA TAGGGACCGA ACTGTCTCAC 1320

GACGTTCTAA ACCCAGCTCG CGTACCACTT TAAATGGCGA ACAGCCATAC CCTTGGGACC 1380

TACTTCAGCC CCAGGATGTG ATGAGCCGAC ATCGAGGTGC CAAACACCGC CGTCGATATG 1440

AACTCTTGGG CGGTATCAGC CTGTTATCCC CGGAGTACCT TTTATCCGTT GAGCGAT 1497

>(262)X Reading_CP051307

CATCGCTCAA CGGATAAAAG GTACTCCGGG GATAACAGGC TGATACCGCC CAAGAGTTCA 60

TATCGACGGC GGTGTTTGGC ACCTCGATGT CGGCTCATCA CATCCTGGGG CTGAAGTAGG 120

TCCCAAGGGT ATGGCTGTTC GCCATTTAAA GTGGTACGCG AGCTGGGTTT AGAACGTCGT 180

GAGACAGTTC GGTCCCTATC TGCCGTGGGC GCTGGAGAAC TGAGGGGGGC TGCTCCTAGT 240

ACGAGAGGAC CGGAGTGGAC GCATCACTGG TGTTCGGGTT GTCATGCCAA TGGCACTGCC 300

CGGTAGCTAA ATGCGGAAGA GATAAGTGCT GAAAGCATCT AAGCACGAAA CTTGCCCCGA 360

GATGAGTTCT CCCTGAGACT TAGAGTCTCC TGAAGGAACG TTGAAGACGA CGACGTTGAT 420

AGGCCGGGTG TGTAAGCGCA GCGATGCGTT GAGCTAACCG GTACTAATGA ACCGTGAGGC 480

TTAACCTTAC AACGCCGAAG ATGTTTTGGC GGATTGAGAA GACAACAATT TTCAGCTCAG 540

ATTCAGAGTC CGAAGGATTT TACGCTGAGA CAAGGCGGCA AACGCAGCGA CGGAAGGAGC 600

ATACAGAAGT ATGTGACTGA CGCTCGCAAG AGCAGCCAAC GCCGTATCAG TGTAAAAGAC 660

ACAGGACGGA GCACAAAGAA TTTGCCTGGC GGCACTAGCG CGGTGGTCCC ACCTGACCCC 720

ATGCCGAACT CAGAAGTGAA ACGCCGTAGC GCCGATGGTA GTGTGGGGTC TCCCCATGCG 780

AGAGTAGGGA ACTGCCAGGC ATCAAATTAG AAGTATGGCA TTAACTTTCG GGGCAGTTCA 840

CTACTCGACA GAGAGTAGGA CAAAAGCGAA AGCTTTTGAA CGTTGCGAAG CAACGGCCCG 900

AAGGGTGAAT CACGAAGTGA TTCATAAACT GCCAGGCATC AAATTAAGCA GTAAACTGAT 960

GTAAAAATCA GTGGTTGTAA AAGAATTCGG TGGAGCGGTA GTTCAGTTGG TTAGAATACC 1020

TGCCTGTCAC GCAGGGGGTC GCGGGTTCGA GTCCCGTCCG TTCCGCCACT TATTAAGAGC 1080

CCTGAGCATT AGCTCAGGGC TTTTTCTTTT CCTGTCATTT AATTATTGCT CAATTCGCAA 1140

AAATCCTCTG CATTTCTCGC TCTTTTTCTG CAAAGCGCTG AAGGTCATAA TTCCCTCAGT 1200

TAACGAACAT AGTGATTAAA AAGAGGAATT CTATGACTAT CCCTGCATTC GGTTTAGGTA 1260

CTTTTCGCCT GAAAGACGAC GTGGTTATCG CTTCTGTTAA AACGGCGCTT GAACTGGGCT 1320

ACCGTGCTGT CGACACTGCG CAAATCTATG ATAATGAAGC GGCTGTTGGA CAGGCGATTG 1380

CTGAGAGCGG CGTACCGCGT AGCGAGCTGT ACATCACCAC CAAGATCTGG ATTGAAAACC 1440

TTAGCAAAGA CAAGCTAATC CCAAGCCTGA AAGAGAGCCT GAAAAAACTG CGTACCGA 1498

>(263)X Tennessee_Gaminara_CP075010_CP024165

TCGGTACGCA GTTTTTTCAG GCTCTCTTTC AGGCTTGGGA TCAGCTTGTC TTTGCTAAGG 60

TTTTCAATCC AGATCTTGGT GGTGATGTAC AGCTCATTAC GCGGTACGCC GCTCTCAGCA 120

ATCGCCTGTC CAACAGCCGC TTCATTATCA TAGATTTGCG CAGTGTCGAC AGCACGGTAG 180

CCCAGTTCAA GCGCCGTTTT CACAGAAGCG ATAACCACGT CGTCTTTCAG GCGAAAAGTA 240

CCTAAACCGA ATGCAGGGAT AGTCATAGAA TTCCTCTTTT TAATCACTAT GTTCGTTAAC 300

TGAGGGGATT ATGACCTTCA GCGCTTTGTA GAAAAAGAGC GAAAAATGCA GAGGATTTTT 360

GCGAATTGAG CAATAATTAA ATGACAGGCA AAGAAAAAGC CCTGAGCTAA TGCTCAGGGC 420

TCTTAATAAG TGGCGGAACG GACGGGACTC GAACCCGCGA CCCCCTGCGT GACAGGCAGG 480

TATTCTAACC AACTGAACTA CCGCTCCACC GAATTTTTTT ACAACCACCA GTCTATGACC 540

GGCTTACTGC TTAATTTGAT GCCTGGCAGT TTATGAATCA CTTCGTGATT CACCCTTCGG 600

GCCGTTGCTT CGCAACGTTC AAAAGCTTTC GCTTTTGTCC TACTCTCTGT CGAGTAGTGA 660

ACTGCCCCGA AAGTTAATGC CATACTTCTA ATTTGATGCC TGGCAGTTCC CTACTCTCGC 720

ATGGGGAGAC CCCACACTAC CATCGGCGCT ACGGCGTTTC ACTTCTGAGT TCGGCATGGG 780

GTCAGGTGGG ACCACCGCGC TAGTGCCGCC AGGCAAATTC TTTGTGCTCT GTCCTGTGTC 840

TTTTACACTG ATACGGCGTT GGCTGCTCTT GCGAACGTCA GTCACATACT TCTGTATGCT 900

CCTTCCGTCG CTGCGTTTGC CGCCTTGTCT CAGCGTAAAA TCCTTCGGAC TCTGAATCTG 960

AGCTGAAAAT CTTCTCTCAA TCCGCCAAAA CATCTTCGGC GTTGTAAGGT TAAGCCTCAC 1020

GGTTCATTAG TACCGGTTAG CTCAACGCAT CGCTGCGCTT ACACACCCGG CCTATCAACG 1080

TCGTCGTCTT CAACGTTCCT TCAGGAGACT CTAAGTCTCA GGGAGAACTC ATCTCGGGGC 1140

AAGTTTCGTG CTTAGATGCT TTCAGCACTT ATCTCTTCCG CATTTAGCTA CCGGGCAGTG 1200

CCATTGGCAT GACAACCCGA ACACCAGTGA TGCGTCCACT CCGGTCCTCT CGTACTAGGA 1260

GCAGCCCCCC TCAGTTCTCC AGCGCCCACG GCAGATAGGG ACCGAACTGT CTCACGACGT 1320

TCTAAACCCA GCTCGCGTAC CACTTTAAAT GGCGAACAGC CATACCCTTG GGACCTACTT 1380

CAGCCCCAGG ATGTGATGAG CCGACATCGA GGTGCCAAAC ACCGCCGTCG ATATGAACTC 1440

TTGGGCGGTA TCAGCCTGTT ATCCCCGGAG TACCTTTTAT CCGTTGAGCG ATG 1493

>(264)X Milwaukee_CP030175

TCGGTACGCA GTTTTTTCAG GCTCTCTTTC AGGCTTGGGA TTAGCTTGTC TTTGCTAAGG 60

TTTTCAATCC AGATCTTGGT GGTGATGTAC AGCTCGCTAC GTGGTACGCC GCTCTCAGTA 120

ATCGCCTGTC CAACAGCCGC TTCATTATCA TAGATTTGCG CAGTGTCGAC AGCACGGTAG 180

CCCAGTTCAA GCGCCGTTTT CACAGAAGCG ATAACCACGT CGTCTTTCAG GCGAAAAGTA 240

CCTAAACCGA ATGCAGGGAT AGTCATAGAA TTCCTCTTTT TAATCACTAT GTTCGTTAAC 300

TGAGGGGATT ATGACCTTCA GCGCTTTGCA GAAAAAGAGC GAGAAATGCA GAGGATTTTT 360

GCGAATTGAG CAATAATTAA ATGACATGCA AAGAAAAAGC CCTGAGCTAA TGCTCAGGGC 420

TCTTAATAAG TGGCGGAACG GACGGGACTC GAACCCGCGA CCCCCTGCGT GACAGGCAGG 480

TATTCTAACC AACTGAACTA CCGCTCCACC GAATTTCTTT GTCACAACCC GGATTTTCAT 540

CCCGGTTTAC TGCAATTTGA TGCCTGGCAG TTTATGAATC ACTTCGTGAT TCACCCTTCG 600

GGCCGTTGCT TCGCAACGTT CAAAAGCTTT CGCTTTTGTC CTACTCTCTG TCGAGTAGTG 660

AACTGCCCCG AAAGTTAATG CCATACTTCT AATTTGATGC CTGGCAGTTC CCTACTCTCG 720

CATGGGGAGA CCCCACACTA CCATCGGCGC TACGGCGTTT CACTTCTGAG TTCGGCATGG 780

GGTCAGGTGG GACCACCGCG CTAGTGCCGC CAGGCAAATT CTTTGTGCTC TGTCCTGTGT 840

CTTTTACACT GATACGGCGT TGGCTGCTCT TGCGAGCGTC AGTCACATAC TTCTGTATGC 900

TCCTTCCGTC GCTGCGTTTG CCGCCTTGTC TCAGCGTAAA ATCCTTCGGA CTCTGAATCT 960

GAGCTGAAAA TTGTTGTCTT CTCAATCCGC CAAAACATCT TCGGCGTTGT AAGGTTAAGC 1020

CTCACGGTTC ATTAGTACCG GTTAGCTCAA CGCATCGCTG CGCTTACACA CCCGGCCTAT 1080

CAACGTCGTC GTCTTCAACG TTCCTTCAGG AGACTTTAAG TCTCAGGGAG AACTCATCTC 1140

GGGGCAAGTT TCGTGCTTAG ATGCTTTCAG CACTTATCTC TTCCGCATTT AGCTACCGGG 1200

CAGTGCCATT GGCATGACAA CCCGAACACC AGTGATGCGT CCACTCCGGT CCTCTCGTAC 1260

TAGGAGCAGC CCCCCTCAGT TCTCCAGCGC CCACGGCAGA TAGGGACCGA ACTGTCTCAC 1320

GACGTTCTAA ACCCAGCTCG CGTACCACTT TAAATGGCGA ACAGCCATAC CCTTGGGACC 1380

TACTTCAGCC CCAGGATGTG ATGAGCCGAC ATCGAGGTGC CAAACACCGC CGTCGATATG 1440

AACTCTTGGG CGGTATCAGC CTGTTATCCC CGGAGTACCT TTTATCCGTT GAGCGATG 1498

>(265)X Paratyphi-B_Typhimurium_CP024619_NC_010102

TCGGTACGCA GTTTTTTCAG GCTCTCTTTC AGGCTTGGGA TTAGCTTGTC TTTGCTAAGG 60

TTTTCAATCC AGATCTTGGT GGTGATGTAC AGCTCGCTAC GCGGTACGCC GCTCTCAGCA 120

ATCGCCTGTC CAACAGCCGC TTCATTATCA TAGATTTGCG CAGTGTCGAC AGCACGGTAG 180

CCCAGTTCAA GCGCCGTTTT AACAGAAGCG ATAACCACGT CGTCTTTCAG GCGAAAAGTA 240

CCTAAACCGA ATGCAGGGAT AGTCATAGAA TTCCTCTTTT TAATCACTAT GTTCGTTAAC 300

TGAGGGAATT ATGACCTTCA GCGCTTTGCA GAAAAAGAGC GAGAAATGCA GAGGATTTTT 360

GCGAATTGAG CAATAATTAA ATGACAGGAA AAGAAAAAGC CCTGAGCTAA TGCTCAGGGC 420

TCTTAATAAG TGGCGGAACG GACGGGACTC GAACCCGCGA CCCCCTGCGT GACAGGCAGG 480

TATTCTAACC AACTGAACTA CCGCTCCACC GAATTTCTTT GTCACAACCC GGATTTTCAT 540

CCCGGTTTAC TGCAATTTGA TGCCTGGCAG TTTATGAATC ACTTCGTGAT TCACCCTTCG 600

GGCCGTTGCT TCGCAACGTT CAAAAGCTTT CGCTTTTGTC CTACTCTCTG TCGAGTAGTG 660

AACTGCCCCG AAAGTTAATG CCATACTTCT AATTTGATGC CTGGCAGTTC CCTACTCTCG 720

CATGGGGAGA CCCCACACTA CCATCGGCGC TACGGCGTTT CACTTCTGAG TTCGGCATGG 780

GGTCAGGTGG GACCACCGCG CTAGTGCCGC CAGGCAAATT CTTTGTGCTC TGTACGCAAT 840

TCTTTATCGC ATCAGCGGCG TTGCCTGCGC TCGTAAACTC AGTCACATAC TTCTGTATGC 900

TCCTTCCTTT CCTTCGCTTG CCGCCTTGCT GCTGCGCAAA TTATTGCGTA CTCAACTCTG 960

AATCAGTGCT GAAAATCGTC TCTCATCCGC CAAAACATCT TCGGCGTTGT AAGGTTAAGC 1020

CTCACGGTTC ATTAGTACCG GTTAGCTCAA CGCATCGCTG CGCTTACACA CCCGGCCTAT 1080

CAACGTCGTC GTCTTCAACG TTCCTTCAGG AGACTCTAAG TCTCAGGGAG AACTCATCTC 1140

GGGGCAAGTT TCGTGCTTAG ATGCTTTCAG CACTTATCTC TTCCGCATTT AGCTACCGGG 1200

CAGTGCCATT GGCATGACAA CCCGAACACC AGTGATGCGT CCACTCCGGT CCTCTCGTAC 1260

TAGGAGCAGC CCCCCTCAGT TCTCCAGCGC CCACGGCAGA TAGGGACCGA ACTGTCTCAC 1320

GACGTTCTAA ACCCAGCTCG CGTACCACTT TAAATGGCGA ACAGCCATAC CCTTGGGACC 1380

TACTTCAGCC CCAGGATGTG ATGAGCCGAC ATCGAGGTGC CAAACACCGC CGTCGATATG 1440

AACTCTTGGG CGGTATCAGC CTGTTATCCC CGGAGTACCT TTTATCCGTT GAGCGATG 1498

>(266)X Java_LT571437

TCGGTACGCA GTTTTTTCAG GCTCTCTTTC AGGCTTGGGA TTAGCTTGTC TTTGCTAAGG 60

TTTTCAATCC AGATCTTGGT GGTGATGTAC AGCTCGCTAC GCGGTACGCC GCTCTCAGCA 120

ATCGCCTGTC CAACAGCCGC TTCATTATCA TAGATTTGCG CAGTGTCGAC AGCACGGTAG 180

CCCAGTTCAA GCGCCGTTTT AACAGAAGCG ATAACCACGT CGTCTTTCAG GCGAAAAGTA 240

CCTAAACCGA ATGCAGGGAT AGTCATAGAA TTCCTCTTTT TAATCACTAT GTTCGTTAAC 300

TGAGGGAATT ATGACCTTCA GCGCTTTGCA GAAAAAGAGC GAGAAATGCA GAGGATTTTT 360

GCGAATTGAG CAATAATTAA ATGACAGGAA AAGAAAAAGC CCTGAGCTAA TGCTCAGGGC 420

TCTTAATAAG TGGCGGAACG GACGGGACTC GAACCCGCGA CCCCCTGCGT GACAGGCAGG 480

TATTCTAACC AACTGAACTA CCGCTCCACC GAATTTCTTT GTCACAACCC GGATTTTCAT 540

CCCGGTTTAC TGCAATTTGA TGCCTGGCAG TTTATGAATC ACTTCGTGAT TCACCCTTCG 600

GGCCGTTGCT TCGCAACGTT CAAAAGCTTT CGCTTTTGTC CTACTCTCTG TCGAGTAGTG 660

AACTGCCCCG AAAGTTAATG CCATACTTCT AATTTGATGC CTGGCAGTTC CCTACTCTCG 720

CATGGGGAGA CCCCACACTA CCATCGGCGC TACGGCGTTT CACTTCTGAG TTCGGCATGG 780

GGTCAGGTGG GACCACCGCG CTAGTGCCGC CAGGCAAATT CTTTGTGCTC TGTACGCAAT 840

TCTTTATCGC ATCAGCGGCG TTGCCTGCGC TCGTAAACTC AGTCACATAC TTCTGTATGC 900

TCCTTCCTTT CCTTCGCTTG CCGCCTTGCT GCTGCGCAAA TTATTGCGTA CTCAACTCTG 960

AATCACTGCT GAAAATCGTC TCTCATCCGC CAAAACATCT TCGGCGTTGT AAGGTTAAGC 1020

CTCACGGTTC ATTAGTACCG GTTAGCTCAA CGCATCGCTG CGCTTACACA CCCGGCCTAT 1080

CAACGTCGTC GTCTTCAACG TTCCTTCAGG AGACCTAAAG TCTCAGGGAG AACTCATCTC 1140

GGGGCAAGTT TCGTGCTTAG ATGCTTTCAG CACTTATCTC TTCCGCATTT AGCTACCGGG 1200

CAGTGCCATT GGCATGACAA CCCGAACACC AGTGATGCGT CCACTCCGGT CCTCTCGTAC 1260

TAGGAGCAGC CCCCCTCAGT TCTCCAGCGC CCACGGCAGA TAGGGACCGA ACTGTCTCAC 1320

GACGTTCTAA ACCCAGCTCG CGTACCACTT TAAATGGCGA ACAGCCATAC CCTTGGGACC 1380

TACTTCAGCC CCAGGATGTG ATGAGCCGAC ATCGAGGTGC CAAACACCGC CGTCGATATG 1440

AACTCTTGGG CGGTATCAGC CTGTTATCCC CGGAGTACCT TTTATCCGTT GAGCGATG 1498

>(267)X Koessen_CP019412

CATCGCTCAA CGGATAAAAG GTACTCCGGG GATAACAGGC TGATACCGCC CAAGAGTTCA 60

TATCGACGGC GGTGTTTGGC ACCTCGATGT CGGCTCATCA CATCCTGGGG CTGAAGTAGG 120

TCCCAAGGGT ATGGCTGTTC GCCATTTAAA GTGGTACGCG AGCTGGGTTT AGAACGTCGT 180

GAGACAGTTC GGTCCCTATC TGCCGTGGGC GCTGGAGAAC TGAGGGGGGC TGCTCCTAGT 240

ACGAGAGGAC CGGAGTGGAC GCATCACTGG TGTTCGGGTT GTCATGCCAA TGGCACTGCC 300

CGGTAGCTAA ATGCGGAAGA GATAAGTGCT GAAAGCATCT AAGCACGAAA CTTGCCCCGA 360

GATGAGTTCT CCCTGAGACT TTAGGTCTCC TGAAGGAACG TTGAAGACGA CGACGTTGAT 420

AGGCCGGGTG TGTAAGCGCA GCGATGCGTT GAGCTAACCG GTACTAATGA ACCGTGAGGC 480

TTAACCTTAC AACGCCGAAG ATGTTTTGGC GGATGAGAGA CGATTTTCAG CACTGATTCA 540

GAGTTGAGTA CGCAATAATT TGCGCAGCAG CAAGGCGGCA AGCGAAGGAA AGGAAGGAGC 600

ATACAGAAGT ATGTGACTGA GTTTACGAGC GCAGGCAACG CCGCTGATGC GATAAAGAAT 660

TGCGTACTGA GCATAAAAGA ATTTGCCTGG CGGCACTAGC GCGGTGGTCC CACCTGACCC 720

CATGCCGAAC TCAGAAGTGA AACGCCGTAG CGCCGATGGT AGTGTGGGGT CTCCCCATGC 780

GAGAGTAGGG AACTGCCAGG CATCAATTTA AGCAGTAAAC TGATGTAAAA ATCAGTGGTT 840

GTAAAAGAAT TCGGTGGAGC GGTAGTTCAG TTGGTTAGAA TACCTGCCTG TCACGCAGGG 900

GGTCGCGGGT TCGAGTCCCG TCCGTTCCGC CACTTATTAA GAGCCCTGAG CATTAGCTCA 960

GGGCTTTTTC TTTGCCTGTC ATTTAATTAT TGCTCAATTC GCAAAAATCC TCTGCATTTT 1020

TCGCTCTTTT TCTACAAAGC GCTGAAGGTC ATAATCCCCT CAGTTAACGA ACATAGTGAT 1080

TAAAAAGAGG AATTCTATGA CTATCCCTGC ATTCGGTTTA GGTACTTTTC GCCTGAAAGA 1140

CGACGTGGTT ATCGCTTCTG TGAAAACGGC GCTTGAACTG GGCTACCGTG CTGTCGACAC 1200

TGCGCAAATC TATGATAATG AAGCGGCTGT TGGACAGGCG ATTGCTGAGA GCGGCGTACC 1260

ACGTAGCGAG CTGTACATCA CCACCAAGAT CTGGATTGAA AACCTTAGCA AAGACAAACT 1320

AATCCCAAGC CTGAAAGAGA GCCTGAAAAA ACTGCGTACC GA 1362

>(268)X Meleagridis_CP074321

AGAGATAAGT GCTGAAAGCA TCTAAGCACG AAACTTGCCC CGAGATGAGT TCTCCCTGAG 60

ACTTAGAGTC TCCTGAAGGA ACGTTGAAGA CGACGACGTT GATAGGCCGG GTGTGTAAGC 120

GTAGCGATGC GTTGAGCTAA CCGGTACTAA TGAACCGTGA GGCTTAACCT TACAACGCCG 180

AAGATGTTTT GGCGGATGAG AGACGATTTT CAGCAGTGAT TCAGAGTTGA GTACGCAATA 240

ATTTGCGCAG CAGCAAGGCG GCAAGCGAAG GAAAGGAAGG AGCATACAGA AGTATGTGAC 300

TGAGTTTACG AGCGCAGGCA ACGCCGCTGA TGCGATAAAG AATTGCGTAC AGAGCACAAA 360

GAATTTGCCT GGCGGCACTA GCGCGGTGGT CCCACCTGAC CCCATGCCGA ACTCAGAAGT 420

GAAACGCCGT AGCGCCGATG GTAGTGTGGG GTCTCCCCAT GCGAGAGTAG GGAACTGCCA 480

GGCATCAAAT TAGAAGTATG GCATTAACTT TCGGGGCAGT TCACTACTCG ACAGAGAGTA 540

GGACAAAAGC GAAAGCTTTT GAACGTTGCG AAGCAACGGC CCGAAGGGTG AATCACGAAG 600

TGATTCATAA ACTGCCAGGC ATCAAATTGC AGTAAACCGG GATGAAAATC CGGGTTGTGA 660

CAAAGAAATT CGGTGGAGCG GTAGTTCAGT TGGTTAGAAT ACCTGCCTGT CACGCAGGGG 720

GTCGCGGGTT CGAGTCCCGT CCGTTCCGCC ACTTATTAAG AGCCCTGAGC ATTAGCTCAG 780

GGCTTTTTCT TTGCATGTCA TTTAATTATT GCTCAATTCG CAAAAATCCT CTGCATTTCT 840

CGCTCTTTTT CTGCAAAGCG CTGAAGGTCA TAATCCCCTC AGTTAACGAA CATAGTGATT 900

AAAAAGAGGA ATTCTATGAC TATCCCTGCA TTCGGTTTAG GTACTTTTCG CCTGAAAGAC 960

GACGTGGTTA TCGCTTCTGT TAAAACGGCG CTTGAACTGG GCTACCGTGC TGTCGACACT 1020

GCACAGATCT ATGATAATGA AGCGGCTGTT GGACAGGCGA TTGCTGAAAG CGGTGTACCA 1080

CGTAATGAGC TGTACATCAC CACCAAGATC TGGATTGAAA ACCTTAGCAA GACAGCTAAT 1140

CCCAGCCTGA AAGAGAGCCT GAAAAACTGC GTACCGATAT GTCGATCTGA CGCTGATCAC 1200

TGGCATCTCC GGTGATGCCT GTCTGTCGAG AATTATGCAG GCTTGCTGGA AGCTAAAAAC 1260

AAGGCTGACC GC 1272
